# Base-catalyzed synthesis of aryl amides from aryl azides and aldehydes†
†Electronic supplementary information (ESI) available: Descriptions of experiments and characterization data. See DOI: 10.1039/c5sc03510d


**DOI:** 10.1039/c5sc03510d

**Published:** 2015-10-22

**Authors:** Sheng Xie, Yang Zhang, Olof Ramström, Mingdi Yan

**Affiliations:** a Department of Chemistry , KTH – Royal Institute of Technology , Teknikringen 36 , Stockholm , Sweden . Email: ramstrom@kth.se ; Email: mingdi_yan@uml.edu; b Department of Chemistry , University of Massachusetts Lowell , Lowell , MA 01854 , USA

## Abstract

Aryl amides have been used as important compounds in pharmaceuticals, materials and in molecular catalysis. The methods reported to prepare aryl amides generally require very specific reagents, and the most popular carboxyl–amine coupling reactions demand stoichiometric activators. Herein, we report that aryl azides react with aldehydes under base-catalyzed conditions to yield aryl amides efficiently. Mechanistic investigations support the formation of triazoline intermediates *via* azide–enolate cycloaddition, which subsequently undergo rearrangement to give amides by either thermal decomposition (20–140 °C) or aqueous acid work-up at room temperature. The strategy does not require nucleophilic anilines and is especially efficient for highly electron-deficient aryl amides, including perfluoroaryl amides, which are otherwise challenging to synthesize.

## Abstract 2

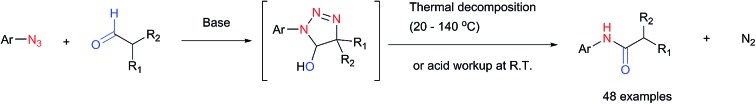
Aryl amides are efficiently synthesized from the rearrangement of triazolines, which formed in the base-catalyzed azide–aldehyde cycloaddtion.

## Introduction

**Scheme 1:**
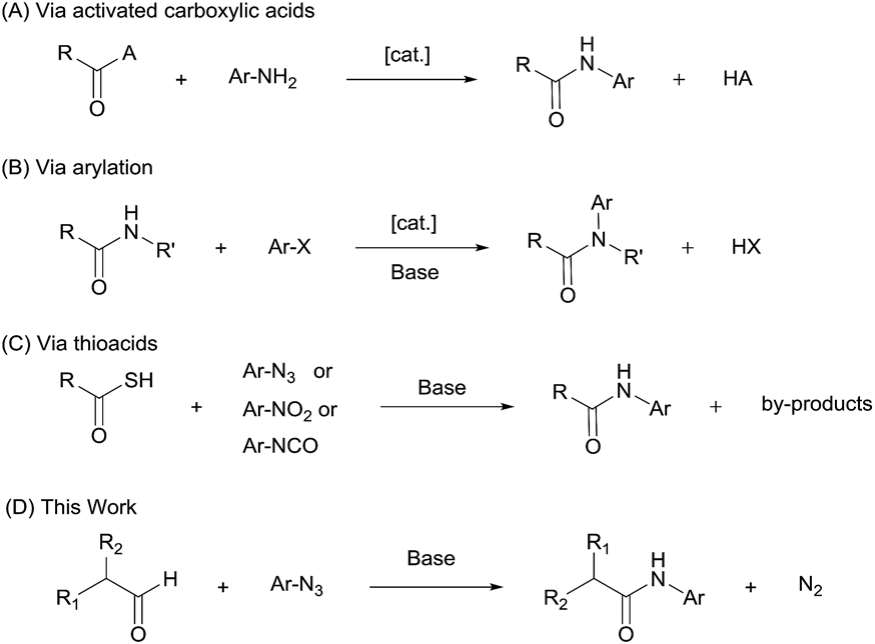
Synthetic approaches to aryl amides.

Aryl amides are an important class of organic compounds, existing ubiquitously in for example pharmaceuticals,^1^–^3^ high performance materials,^4^,^5^ synthetic auxiliaries,^6^–^10^ and supramolecular assemblies.^11^ Traditional aryl amide synthesis generally follows the same approach as for aliphatic amides, *i.e.*, by coupling a nucleophilic aniline with an activated carboxylic acid (Scheme 1A).^12^,^13^ Popular methods that involve refluxing anilines with acyl chlorides or anhydrides, or the use of a coupling reagent often show poor atom economy.^14^–^16^ Thermally driven condensation of carboxylic acids and anilines can also be envisaged.^17^ However, many mild amidation protocols that work excellently for aliphatic amides perform sluggishly for aryl amides.^18^–^21^ In addition, for electron-deficient anilines, which are weak nucleophiles, these strategies suffer from harsh conditions, low yields, and wasteful work-up. Alternative aryl amide syntheses include arylation of aliphatic amides (Scheme 1B),^22^–^26^ aminocarboxylation of aryl halides^27^ or ketene precursors,^28^,^29^ N-heterocyclic carbene-mediated^30^–^33^ or Ni-catalyzed dehydrogenative coupling of aldehydes,^34^ and direct oxidative amidation of aldehydes^35^ or alcohols.^36^–^38^ These protocols require special reagents, are generally conducted at elevated temperatures (80 °C–200 °C), and can rarely be applied to highly electron-deficient aryl amides. To overcome the limitation of the weak nucleophilicity of anilines, a different approach involving N-centered radicals has also been developed,^39^ however suffering from limited substrate scope.^40^–^42^ Thus, new and improved methods for anilide formation are needed.

Synthetic methods using thiocarboxylic acids with aryl azides,^43^–^45^ isocyanates^46^ or nitroarenes^47^ have been reported to produce aryl amides efficiently under relatively mild conditions (Scheme 1C). These reactions are generally initiated by nucleophilic addition of the thioacetate anion to the respective nitrogen-containing species, followed by rearrangement and elimination. In thioacid/azide reaction, for example, the aryl amide is formed *via* a thiotriazoline intermediate and subsequent loss of sulfur and molecular nitrogen. This ‘tethering-rearrangement’ strategy eliminates the requirement of a nucleophilic aniline in the amidation reaction. However, it has met with limited success due to the difficulty in accessing thiocarboxylic acid reagents and issues with their stability.

The azide–aldehyde reaction to yield the amide while releasing N_2_ gas as the only byproduct is an attractive strategy, however, it has limitation regarding the azide substrate.^48^ The Boyer reaction requires stoichiometric amount of a strong acid and has shown only limited success and moderate yields with aliphatic azides in the intermolecular reactions.^49^,^50^ Radical mediated azide–aldehyde reactions all needed specific directing groups on substrates.^40^–^42^

**Scheme 2:**
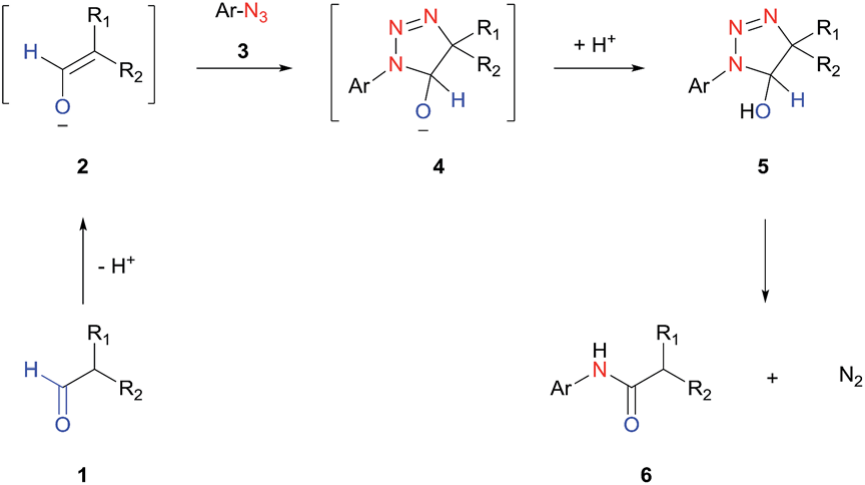
Proposed base-catalyzed amidation.

In this work, we report a general strategy for the synthesis of aryl amides from aryl azides and aldehydes (Scheme 1D). We hypothesize that the enolate anion (**2**) formed from aldehyde (**1**) under basic conditions would undergo 1,3-dipolar cycloaddition with azide (**3**) to form 5-hydroxytriazoline (**5**), which would then rearrange to aryl amide (**6**) with extrusion of molecular nitrogen (Scheme 2). The strategy of intermolecular tethering *via* cycloaddition would partially obviate the requirement of a strongly nucleophilic *N*–Ar species and would offer a straightforward synthesis route. Moreover, the availability of a wide variety of aldehydes and azides is of high practical advantage. Although the triazoline formation under basic condition is expected to proceed efficiently,^51^,^52^ the control over the rearrangement of 5-hydroxytriazoline (**5**) to amide presents a challenge. Depending on the substituents and reaction conditions, 5-hydroxytriazoline can undergo different reactions, primarily leading to triazoles.^53^–^56^ Herein, we report the discovery of strategies to facilitate the conversion of the triazolines to aryl amides. Thus, a wide range of aryl azides and aldehydes react under mild conditions to give aryl amides in high yields.

## Results and discussion

Initial studies were carried out using cyclohexanecarbaldehyde (**1a**) and phenyl azide (**3a**) in DMSO-*d*_6_ together with 10 mol% cesium carbonate, and the reaction was monitored by NMR (Table 1, entry 1). Complete conversion to triazoline **5a** was observed within six hours but no amide (**6a**) was detected at 30 °C (Fig. S1†). After workup with 1 M HCl, aryl amide **6a** was obtained in 90% yield (entry 1). Alternatively, microwave heating in DMSO at 100 °C gave aryl amide **6a** within one hour in 96% yield (entry 3). Conventional heating showed identical efficiency. Increasing the temperature accelerated both the cycloaddition to triazoline and the rearrangement to aryl amide. Further screening of the base catalyst showed a positive correlation between the conversion and the strength of the base, in agreement with the degree of enolate formation. The use of KOH in DMSO, an inexpensive and stronger base,^57^ resulted in completion of the reaction within 30 minutes while maintaining an excellent yield (entry 4). Potassium carbonate gave the product in 82% yield together with the un-reacted azide starting material (entry 5). For weaker bases such as triethylamine, no amide product was observed under the same conditions (entry 6). Compared to DMF, acetonitrile and ethanol, DMSO was the most efficient solvent (entries 4, 7–9), likely owing to its ability to enhance basicity.^57^

**Table 1:** Optimization of the reaction conditions^*a*^

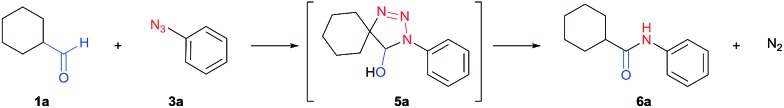				
#	Base (0.1 eq.)	Solvent	Temp./time	Yield (%)
1	Cs_2_CO_3_	DMSO	30 °C/6 h	90^*b*^
2	Cs_2_CO_3_	DMSO	80 °C/1 h	87
3	Cs_2_CO_3_	DMSO	100 °C/1 h	96
4	KOH	DMSO	100 °C/0.5 h	96 (92^*c*^)
5	K_2_CO_3_	DMSO	100 °C/1 h	82
6	NEt_3_	DMSO	100 °C/1 h	0
7	KOH	DMF	100 °C/0.5 h	60
8	KOH	MeCN	100 °C/0.5 h	55
9	KOH	EtOH	100 °C/0.5 h	35
10	Cs_2_CO_3_	EtOH	100 °C/0.5 h	0

^*a*^Conditions: **1a** (1.15–1.25 mmol), **3a** (1 mmol), solvent (2 mL); microwave heating, sealed tube; isolated yield.

^*b*^Workup with aq. HCl (1 M).

^*c*^ **3a** (5 mmol).

**Fig. 1:**
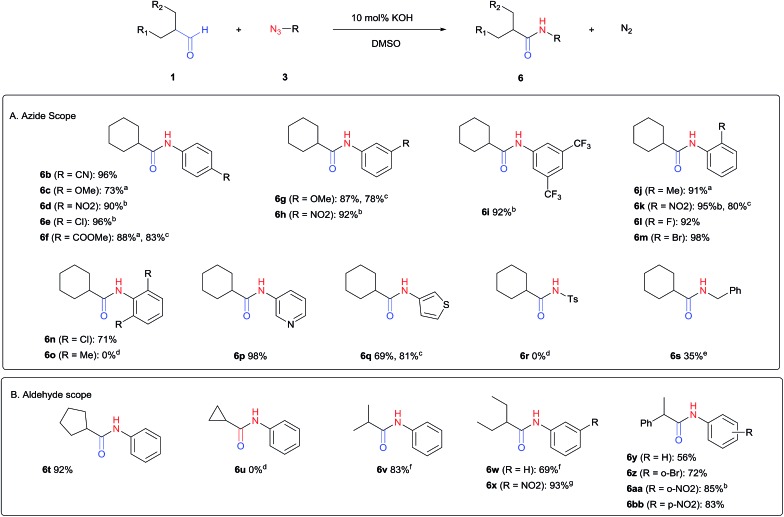
Substrate scope using α-monosubstituted aldehydes. Conditions: aldehyde **1** (1.15–1.25 mmol), azide **3** (1 mmol), DMSO (2 mL), microwave heating at 100 °C, 0.5 h, isolated yield. ^a^ 1 h. ^b^ 80 °C. ^c^ 30 °C, 6–24 h, quenched by 0.5 M aq. AcOH (2 mL). ^d^ 80–160 °C, 2–10 h. ^e^ 120 °C, 1 h. ^f^ 140 °C, 2 h. ^g^ 60 °C, 12 h.

The scope of the azide was next investigated under base catalysis and thermal conditions using cyclohexanecarbaldehyde (**1a**) (Fig. 1A). In general, the reactions were carried out at least 20 °C below the decomposition temperature of the azide (Fig. S2†). The aryl azide showed high tolerance to various substituents including cyano (**6b**, 96%), methoxy (**6c**, 73%; **6g**, 87%), trifluoromethyl (**6i**, 92%), methyl (**6j**, 91%) as well as base-labile nitro (**6d**, 90%; **6h**, 92%; **6k**, 95%), carboxylic ester (**6f**, 88%) and halo (F, **6l**, 92%; Cl, **6d**, 96%; Br, **6k**, 98%) groups.

Reactions with phenyl azides having electron-withdrawing groups generally proceeded faster, and the amidation was completed at a lower temperature (80 °C) compared to phenyl azide (100 °C). An exception was *p*-cyanophenyl azide, which gave the partial amide product at 80 °C, albeit the reaction could be completed neatly at 100 °C (**6b**, 96%). On the other hand, aryl azides having electron-donating groups generally required longer reaction times compared to phenyl azide, and gave slightly lower but still good isolated yields (**6c**, 73%; **6g**, 87%). In these cases, a small amount of aldol products were observed which contributed to the decreased amide yields. The steric effect of the *ortho*-group on the aryl azide was also examined. Monosubstitution at the *ortho* position had no impact; for example, 2-methylphenyl azide gave amide **6j** in 91% isolated yield. While 2,6-dichlorophenyl azide gave good isolated yield (**6n**, 71%), no product was formed from 2,6-dimethylphenyl azide (**6o**) after extensive optimization, likely owing to the high steric hindrance of the two *ortho* methyl groups. Heterocyclic aryl azides also performed well in the reaction. 3-Azidopyridine resulted in an excellent yield (**6p**, 98%), and 3-azidothiophene gave the corresponding amide in good yield (**6q**, 69%) together with noticeable black tar indicating thermal decomposition at 100 °C. Benzyl azide could also undergo amidation (**6s**, 35%), whereas, surprisingly, electrophilic tosyl azide did not give any amide product.

The reaction could furthermore be carried out at room temperature (entry 1, Table 1). In this case, the cycloaddition was completed within 6–24 hours to give the triazoline, after which the amide was obtained in high yield following acid workup (**6g**, 78%; **6q**, 81%). For highly electron-deficient azides, small amounts of aniline were observed; however, the aryl amides could still be isolated in good yields (**6f**, 83%; **6k**, 80%; **6i**, 78%). These results thus demonstrate a room temperature process for aryl amide synthesis.

**Scheme 3:**
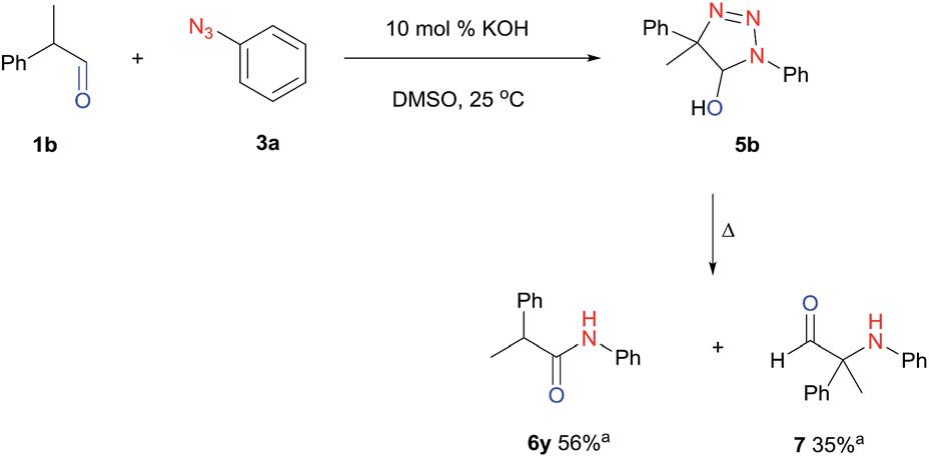
Thermal rearrangement of triazoline **5b**.

Fig. 1B displays the aldehyde scope. Cyclopentyl aldehyde proved to be an excellent substrate (**6t**, 92%), whereas cyclopropane carbaldehyde did not give any product even at 160 °C, likely due to unfavorable enolate formation (**6u**).^58^ Acyclic isobutyraldehyde gave amide **6v** in 83% isolated yield; however requiring a temperature of 140 °C for 2 h to go to completion, likely due to the lower accessibility of the acyclic enolate compared to the cyclic counterparts. Similarly, 2-ethylbutanal gave the aryl amide within 2 h at 140 °C (**6w**, 69%), however being unreactive at 100 °C. The lower reactivity of acyclic aldehydes could be largely overcome by using an electron-deficient azide, where, for example, *m*-nitrophenyl azide reacted with 2-ethylbutanal neatly at 60 °C (**6x**, 93%). In addition, acyclic 2-phenylacetaldehyde, having an activated α-proton, reacted readily with all aryl azides (**6y**, **6z**, **6aa**, **6bb**). The moderate yield of **6y** (56%) was associated with the α-amino-ketone by-product **7** (35% yield, Scheme 3) formed from the triazoline intermediate.^54^ However, much less α-amino-ketone by-product was observed for electron-deficient azides, and the yields for the isolated amides increased significantly (**6z**, 72%; **6aa**, 85%; **6bb**, 83%). Non-enolizable aldehydes such as benzaldehyde showed no product formation as expected.

We next investigated the reaction of α-unsubstituted aldehydes. When the same conditions (0.1 equiv. KOH/DMSO) were applied to 3-phenylpropanal, amide **6cc** was not detected but the triazole was instead formed together with a large amount of unreacted azide (Table S2†). The low azide conversion (40–50%) was also associated with substantial aldol self-condensation. To promote azide–enolate cycloaddition, the protocol was modified by premixing the azide with *t*-BuOK followed by dropwise addition of excess aldehyde (Table S2†). The use of THF/*t*-BuOH as solvent further inhibited the dehydration/aromatization, and amide **6** was isolated after acid workup in up to 68% yield (Table 2, entry 1).^54^ This strategy could be applied to various α-unsubstituted aldehydes and azides, showing good substrate scope to yield aryl amides in 50–73% yields at room temperature (Table 2). Electron-deficient azides (entries 4–5) underwent cycloaddition much faster than phenyl azide, but required additional optimization to maximize the amide yield.

**Table 2:** Amidation of α-unsubstituted aldehydes^*a*^

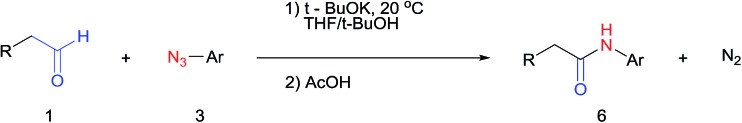				
#	**1**	**3**	Product	Yield^*b*^ (%)
1	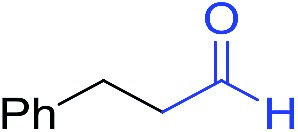	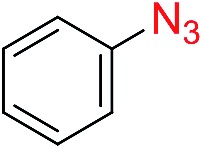	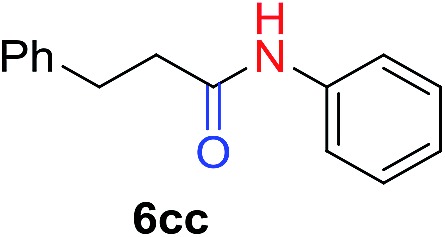	68
2	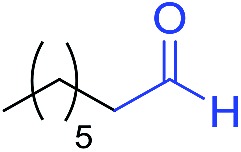	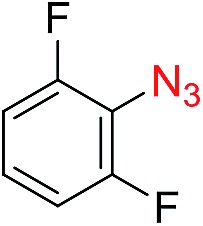	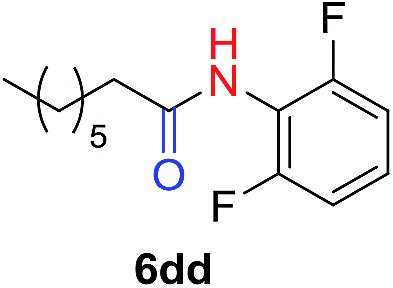	67
3	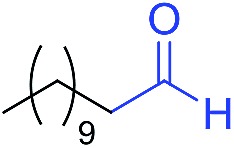	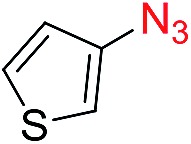	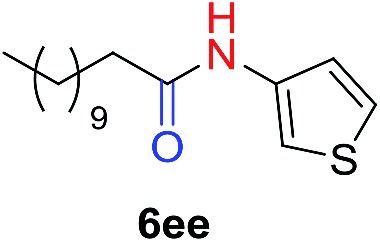	73
4^*c*^	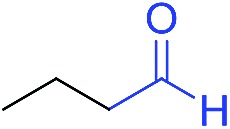	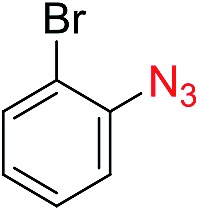	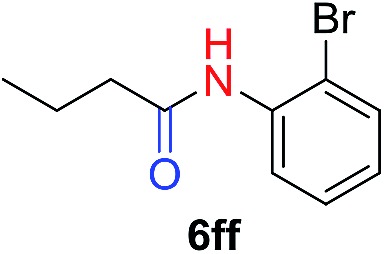	57
5^*c*^	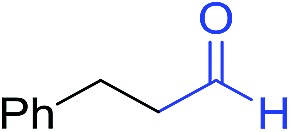	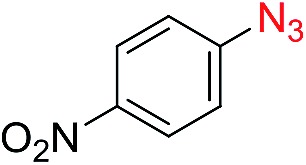	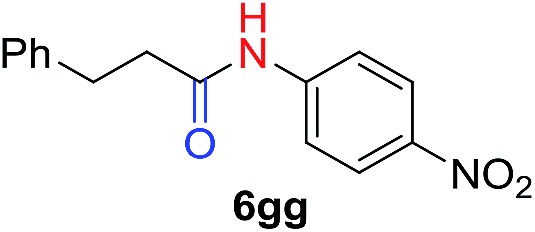	50

^*a*^Protocol: to a solution of azide **3** (1 mmol) and *t*-BuOK (2 equiv.) in THF/*t*-BuOH (1 mL/0.5 mL) under vigorous stirring, aldehyde **1** (4 mmol) in THF (0.5 mL) was added dropwise. After the reaction was completed (1–5 minutes), the solution was quenched with 1.5 M aq. AcOH (2 mL).

^*b*^Isolated yield.

^*c*^ *t*-BuOK (1.2 equiv.), the volume of solvent was doubled.

**Fig. 2:**
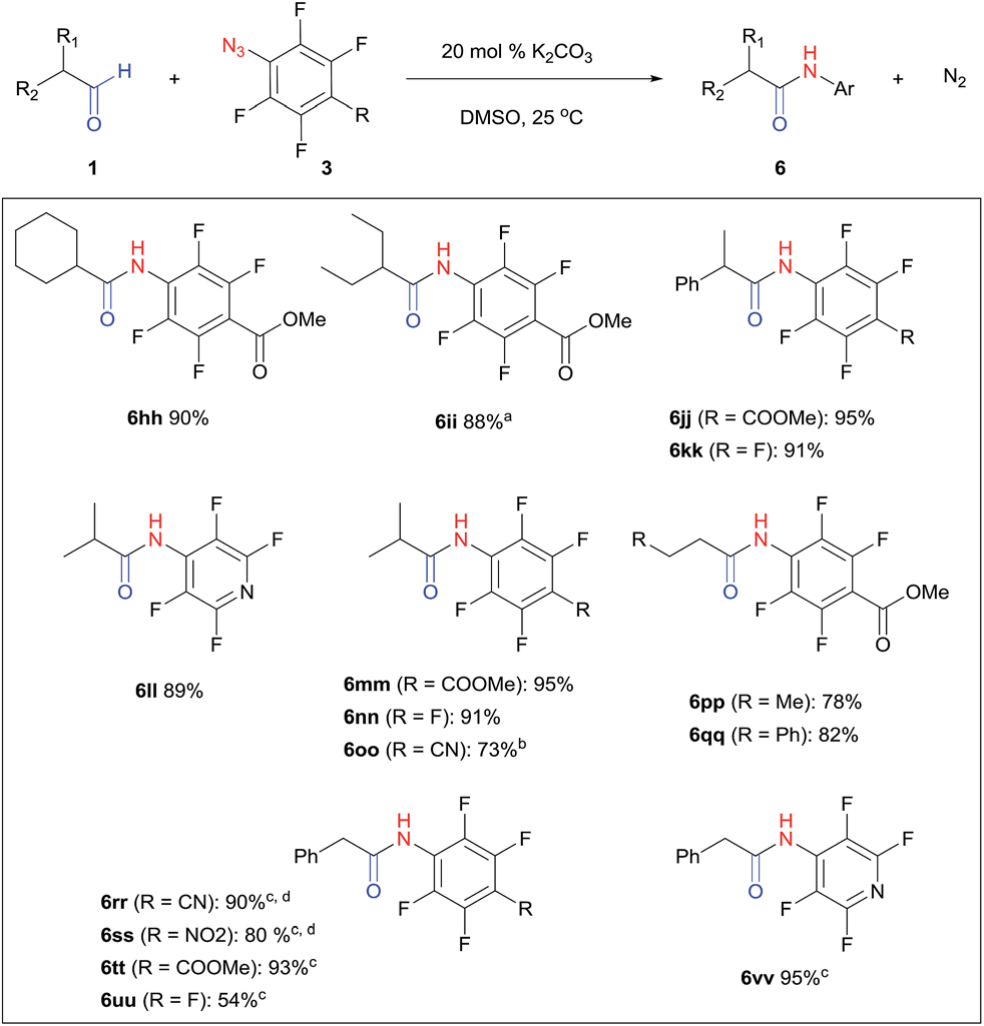
Synthesis of perfluoroaryl amides. Conditions: aldehyde **1** (1.15–1.25 mmol), azide **3** (1 mmol), DMSO (2 mL), 1–12 h, isolated yield. ^a^ 60 °C. ^b^ 50 °C, 4 d. ^c^*p*-Dimethylaminopyridine (DMAP) was used as base. ^d^ 40 °C, 16 h.

Perfluoroaryl amides constitute an important subclass of amides. For example, they are used as auxiliaries in C–H activations, the synthesis of which is not trivial owing to the significant deactivation by the electronegative F atoms.^9^,^59^–^63^ The protocol developed in this study could be readily applied to perfluoroaryl azides giving perfluoroaryl amides in high yields. Generally, this reaction proceeded within 1–12 hours under mild conditions at room temperature in the presence of K_2_CO_3_ in DMSO (Fig. 2). *p*-Cyano (**6oo**, **6rr**) and *p*-nitro (**6ss**) perfluoroaryl azides required slightly higher temperature (40–60 °C), likely due to the highly electron-withdrawing group that slowed down the formation of triazoline. Interestingly, α-unsubstituted aldehydes, which resulted in substantial triazole formation with other aryl azides, showed high selectivity with perfluorophenyl azides to yield aryl amides exclusively (**6pp**, 78%; **6qq**, 82%). For the activated phenylacetaldehyde, a weak base such as DMAP or imidazole was sufficient to promote the reaction to form the perfluoroaryl amides neatly (**6rr–6vv**, 54–95%). In addition, acid workup was not needed, which further simplifies the synthetic protocol.

The triazolines formed from perfluoroaryl azides rearranged directly into aryl amides and could not be detected by NMR. The preference of rearrangement over dehydration is likely due to the highly electron-deficient perfluoroaryl group, which facilitates nitrogen extrusion. This is similar to the previously studied perfluoroaryl azide–enamine cycloaddition that yields an amidine from the corresponding 5-amino-triazoline intermediate.^64^

## Conclusions

We have developed a general approach for the synthesis of aryl amides from aryl azides and aldehydes by a base-catalyzed cycloaddition/rearrangement strategy. This process involves triazoline formation between enolates and aryl azides, eliminating the requirement of a nucleophilic aniline or organometallic catalyst in the amidation reaction. The triazoline intermediate readily undergoes thermally induced or acid-mediated rearrangement into aryl amide accompanied by the extrusion of N_2_. This base-catalyzed protocol uses mild conditions to give aryl amides, including highly electron-deficient perfluoroaryl amides, in high yields. The straightforward protocol and mild conditions, coupled with high availability of starting materials, makes this method a valuable approach to access a wide range of aryl amides.

## Supplementary Material

Supplementary information
