# Supplementary material for: Base-catalyzed synthesis of aryl amides from aryl azides and aldehydes
Source: Chem Sci. 2015 Oct 22;7(1):713–8. doi: 10.1039/c5sc03510d (PMC5952891; doi:10.1039/c5sc03510d)
Supplement: Supplementary file 1 [file SC-007-C5SC03510D-s001.pdf]

Electronic Supplementary Material (ESI) for Chemical Science.
This journal is © The Royal Society of Chemistry 2015

**SUPPORTING INFORMATION**

# Base-Catalyzed Synthesis of Aryl Amides from Aryl Azides and Aldehydes

Sheng Xie,[a] Yang Zhang,[a] Olof Ramström,*[,a] and Mingdi Yan*[,a,b]

[a]Department of Chemistry, KTH - Royal Institute of Technology, Teknikringen 36, S-10044 Stockholm, Sweden
[b]Department of Chemistry, University of Massachusetts Lowell, 1 University Ave., Lowell, MA 01854, USA

*E-mail: ramstrom@kth.se, Mingdi_Yan@uml.edu

## Table of Contents

General procedures ..... S1
Figure S1. $^{1}$H NMR spectra of model reaction ..... S2
Figure S2. Azide decomposition temperatures ..... S2
Table S1. Optimization of conditions for reaction of α-unsubstituted aldehyde with phenyl azide. ..... S3
Synthesis of azides ..... S3
Synthesis of products ..... S7
References ..... S16
Characterization Spectra ..... S18

## General procedures

All reagents and solvents were used as received from Sigma Aldrich, Alfa Aesar, Fluka, and Merck. Thin-layer chromatography was conducted using TLC silica gel 60 $F_{254}$ (Merck Co.), visualized with ultraviolet light. $^{1}$H-, $^{13}$C- and $^{19}$F-NMR data were recorded on a Bruker Ascend$^{TM}$ 400 instrument or a Bruker DMX 500 instrument. Chemical shifts are reported as δ values (ppm) with $CDCl_3$ ($^{1}$H: δ = 7.26, $^{13}$C: δ = 77.16), DMSO-$d_6$ ($^{1}$H: δ = 2.50, $^{13}$C: δ = 39.52) or acetone-$d_6$ ($^{1}$H: δ = 2.05, $^{13}$C: δ = 29.84) as the internal standard. $^{19}$F NMR signals were referenced to hexafluorobenzene (δ = -161.75 in $CDCl_3$ or -162.65 in DMSO-$d_6$) unless noted otherwise. High resolution electrospray ionization (HRMS-ESI) mass spectrometry data were obtained from the Mass Spectrometry Lab at the University of Illinois at Urbana–Champaign. IR spectra were recorded on ReactIR$^{TM}$ IC10 (Mettler Toledo Co.) for liquid samples, or SPECTRUM 2000 (Perkin Elmer) for solid samples in the ATR mode.

**Figure S1.$^{1}$H NMR spectra of model reaction**

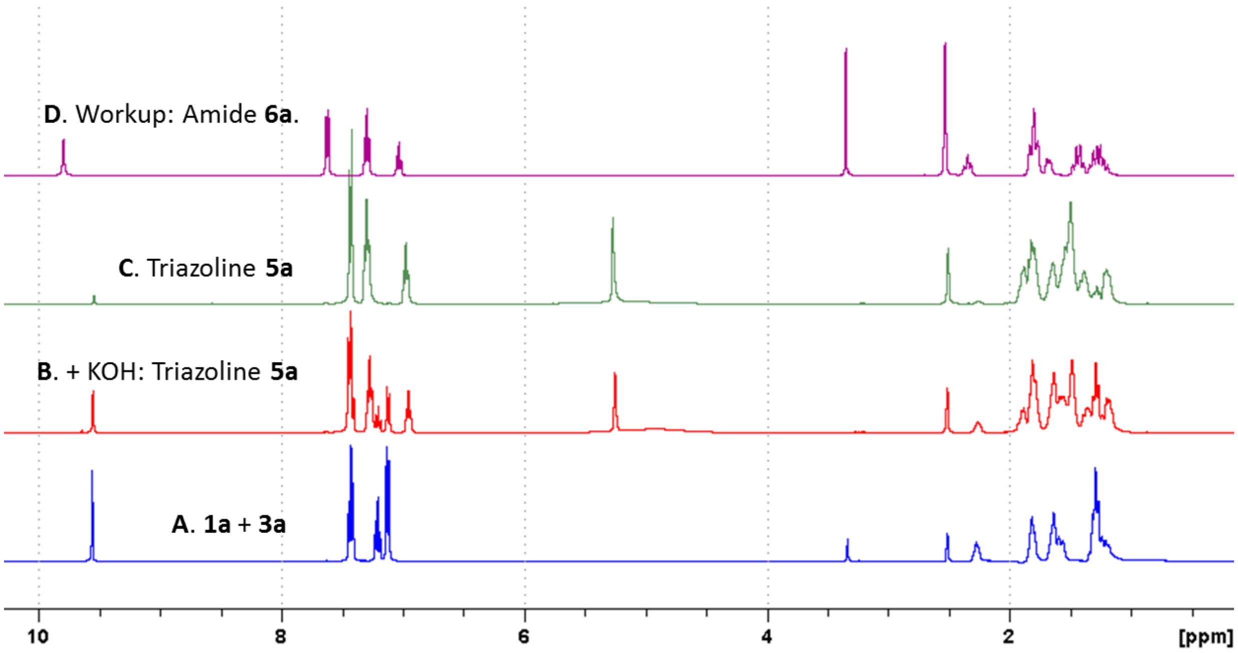


A). Azide and aldehyde in DMSO-$d_6$; B).Triazoline**5a** formed after addition of KOH;C).Triazoline**5a** formed exclusively(6 h); D). Aqueous acidicworkup yieldingamide **6a**.

**Figure S2. Azide decomposition temperatures***

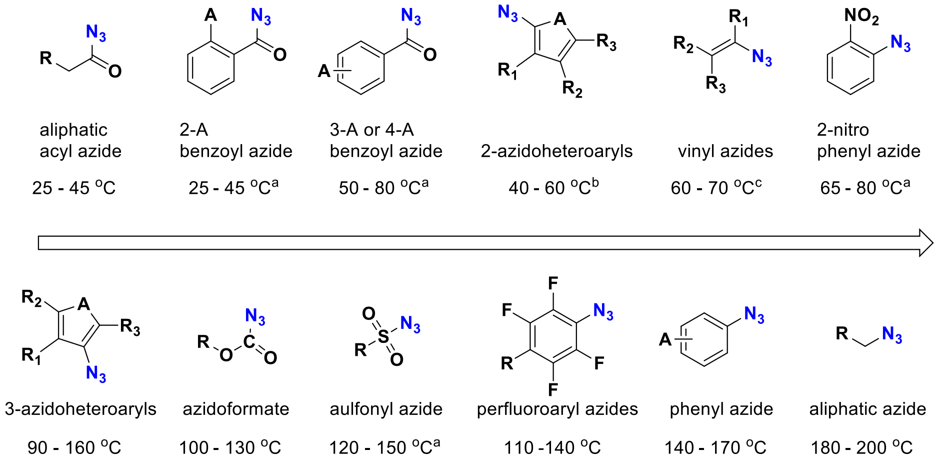


*The temperature rangewasdetermined where the rate constant for the decomposition of azide was $10^{-5}$ to $10^{-4}s^{-1}$ ($t_{0.5}$ = 2 – 24h), following the suggestion in ref.1. [a]Ref. 1 and references therein; [b]ref. 2; [c]ref. 3; [d]ref. 4.

**Table S1. Optimization of conditions for reaction of α-unsubstituted aldehyde with phenyl azide.**[a]

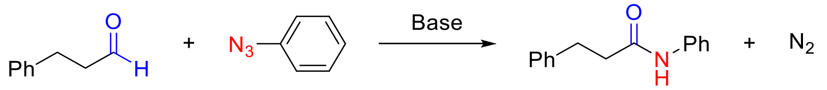


**1c** **3a** **6cc**

| Entry | Base (eq.) | Solvent (v:v) | **1c** (eq.) | Temp. /Time. | Conv.[b] of **3a** (%) | Yield[c] of **6cc** (%) |
|---|---|---|---|---|---|---|
| 1 | t-BuOK (0.5) | THF/*t*-BuOH (3:1) | 3.0 | 20 °C/10 min | 10 | 8 |
| 2 | t-BuOK (1.1) | THF/*t*-BuOH (3:1) | 3.0 | 20 °C/6 min | 70 | 48 (66[d]) |
| 3 | t-BuOK (1.5) | THF/*t*-BuOH (3:1) | 4.0 | 20 °C/5 min | 71 | 57 (86[d]) |
| 4 | t-BuOK (2.0) | THF/*t*-BuOH (3:1) | 4.0 | 20 °C/5 min | > 95 | 68 |

[a]Protocol: to a solution of **3a** (1 mmol) and base in THF/*t*-BuOH(1 mL/0.5 mL) under vigorous stirring, aldehyde **1c** in THF (0.5 mL) was added dropwise during 0.5 min. After the reaction was completed, the solution was quenched by aq. AcOH (1.5 M, 2 mL). [b]Determined by $^1$H NMR. [c]Isolated yield.[d]Yield based on recycled azide.

## Synthesis of azides

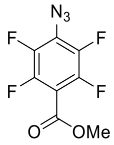


**Methyl 4-azido-2,3,5,6-tetrafluorobenzoate**.[5]**General procedure A**: Methyl pentafluorobenzoate (9.5 g, 40 mmol) was dissolved in a 2:1 (v/v) mixture of acetone and water (90 mL). Sodium azide (3.40 g, 52 mmol, 1.3 equiv.) was added to the flask and the mixture was refluxed at 85 °C for 6 h. The mixture was subsequently cooled to r.t., diluted with water (150 mL), and extracted with diethyl ether (3 x 150 mL). The extract was dried over $MgSO_4$ and the solvent evaporated under reduced pressure, yielding the product as colorless crystals (9.5 g, 95%). Further purification was performed by flash column chromatography using EtOAc:hexanes (1:40) as the eluent. The compounds were stored in the dark to prevent extensive light exposure. $^1$H NMR (400 MHz, $CDCl_3$): δ 3.97 (s, 3H); $^{13}$C NMR (125 MHz, $CDCl_3$): δ 160.0, 145.5 (dm, $J_{C\text{-}F}$ = 260.1 Hz), 140.6 (dm, $J_{C\text{-}F}$ = 250.1 Hz), 123.5, 107.8, 53.4; $^{19}$F NMR (376 MHz, $CDCl_3$): δ -138.62 (m, 2F), -150.91 (m, 2F).

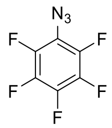


**1-Azido-2,3,4,5,6-pentafluorobenzene**.[6]**General procedure B**: Aniline (20 mmol) was dissolved in TFA (25 mL) and cooled to -2 °C using an immersion cooler. After ~15 min, $NaNO_2$ (12 mmol) was added in portions while stirring. After stirred at 0 °C for 1h, sodium azide (30 mmol) was added and the mixture was stirred at -2-0 °C for 1h. The mixture was diluted with

$Et_2O$ (50 mL) and washed with water and then saturated $NaHCO_3$ and dried over $MgSO_4$. Afterremovalofsolvent,theresiduewaspurifiedbyflashcolumnusing pentane as eluent to give a pale brown liquid in 82% yield. $^{13}$C NMR (125 MHz, $CDCl_3$): δ 115.89 (dt, J = 4.63, 12.5 Hz), 138.14 (dm, J = 261.30 Hz), 141.02 (dm, J = 250.0 Hz); $^{19}$F NMR (376 MHz, $CDCl_3$): -151.48 (m, 2F), -159.62 (m, 1F), -161.11 (m, 2F).

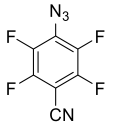


**4-Azido-2,3,5,6-tetrafluorobenzonitrile**. Synthesized according to general procedure A. The product was purified by column chromatography using hexanes:EtOAc mixture as eluent to give a light yellow liquid in 65% yield. $^{13}$C NMR (125 MHz, $CDCl_3$): δ 89.1 (t, 1C, CN, J = 17.5 Hz), 107.2 (t, 1C, J = 3.6 Hz), 126.7 (m, 1C), 140.3 (dm, 2C, $J_{C\text{-}F}$ = 252.4 Hz), 147.5 (dm, 2C, $J_{C\text{-}F}$ = 261.5 Hz); $^{19}$F NMR (376 MHz, $CDCl_3$): δ -132.05 (m, 2F), -148.68 (m, 2F).

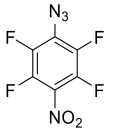


**1-Azido-2,3,5,6-tetrafluoro-4-nitrobenzene**. Synthesized according to general procedure A. The product was purified by column chromatography using hexanes:EtOAc (70:1) mixture as eluent to give a pale yellow liquid in 67% yield. $^{13}$C NMR (100 MHz, $CDCl_3$): δ 125.4 (t, 1C, J = 3.6 Hz), 126.6 (m, 1C), 140.7 (dm, 2C, $J_{C\text{-}F}$ = 252.0 Hz), 141.4 (dm, 2C, $J_{C\text{-}F}$ = 264.0 Hz);$^{19}$F NMR (376 MHz, $CDCl_3$): δ -145.65 (m, 2F), -148.76 (m, 2F).

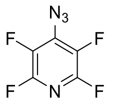


**4-Azido-2,3,5,6-tetrafluoropyridine**.[7] The product was purified by distillation using a Büchi Kügelrohr apparatus to give a colorless liquid in 68% yield. $^{13}$C NMR (125 MHz, $CDCl_3$): *δ* 132.2 (m, $C_{(4)}$-$N_3$), 135.4 (dm, 2C, $J_{C\text{-}F}$= 261.8 Hz), 143.6 (dm, 2C, $J_{C\text{-}F}$= 244.1 Hz). $^{19}$F NMR (376 MHz, CDCl3): δ -89.59 (m, 2F), -152.91 (m, 2F).

**Phenyl Azide (6)**.[8]**General procedure C**: To a 250 mL round-bottom flask,charged with aniline (0.02 mol) and water(40 mL), was added concentratedHCl(20.0 mL,0.21 mol) under vigorousstirring while cooling using an immersion cooler. After stirring at -2 °C for 20-30 min, afreshly prepared, ice-cold solution of sodium nitrite (1.9 g, 0.03 mol) in water (10 mL) wasadded dropwise and the mixture was stirred for an additional 10-20 min. A freshly prepared solution of sodium azide (2.5 g, 0.03 mol) in water (20 mL) was then added dropwise to the reaction mixture while maintaining the temperature below 5 °C, after which the reaction mixture was stirred for an additional 20–30 min at 0 °C, followed by stirring at rt for 1 h. Afterwards, the solution was extracted with EtOAc (50mL x 3), washed with saturated $NaHCO_3$ solution and brine (75mL), and dried over $Na_2SO_4$. After removal of solvent, the crudemixture was purified by column chromatography using hexanes as eluent to give an orange oil in 63% yield. $^{1}$H NMR (400 MHz, $CDCl_3$): δ 7.04 (d, 2H, J=8.2 Hz), 7.15 (t, 1H, J=8.2 Hz), 7.36 (t, 2H, J=8.2 Hz); $^{13}$C NMR (125 MHz, $CDCl_3$): δ 119.1 (2C), 124.9, 129.8 (2C), 140.0.

**Benzyl azide**.[9] The product was purified by column chromatography using pentane to give a colorless oil in 90% yield. $^{1}$H NMR (400 MHz, $CDCl_3$): δ 7.35 (m, 5H, Ar), 4.35 (s, 2H, $CH_2$); $^{13}$C NMR (125 MHz, $CDCl_3$): δ 146.3, 135.6, 130.4, 127.6, 21.8.

**Tosyl Azide**.[10]Colorless oil in 98% yield.$^{1}$H NMR (500 MHz, $CDCl_3$): δ 2.48 (s, 3H, $CH_3$), 7.41(d, 2H, J=8.4 Hz), 7.84(d, 2H, J=8.4 Hz). $^{13}$C NMR (125 MHz, $CDCl_3$): δ 21.8, 127.6 (2C), 130.3 (2C), 135.6, 146.4.

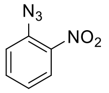

**1-Azido-2-nitrobenzene**.[11] A mixture of *o*-nitroaniline (2.8 g, 0.02 mol), water (80 ml) and concentrated hydrochloric acid (45 mL, 0.54 mmol) was placed in a 500 mL flask. The flask was cooled to 0°C and sodium nitrite (1.45 g, 0.021 mol ) in water (10 mL) was added dropwise. After 30 min stirring at 0°C, sodium azide (13.0 g, 0.20 mol) in water (10 mL)was added dropwise, during which the product precipitatedout as anoff-white solid. After the nitrogen evolution had ceased, the product was collected on a Büchner funnel and washed twice with ice-cold water and then dried in air overnight to give the product as pale yellowneedles (2.1 g, 88 %). $^{13}$C NMR (125 MHz, $CDCl_3$): δ140.9, 134.8,134.1, 126.1, 125.1, 120.8;$^{1}$H NMR (400 MHz, $CDCl_3$): δ 7.92 (dd, 1H, J= 8.1, 1.5 Hz), 7.60 (dt, 1H, J= 7.9, 1.4 Hz), 7.31 (dd, 1H, J= 8.4, 1.0 Hz), 7.23 (dt, 1H, J= 7.7, 1.1 Hz).

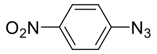

**1-Azido-4-nitrobenzene**.[12](0.02 mol scale) Synthesized according to general procedureC. The product was purified by column chromatography using hexanes: DCM 1:1 mixture as eluent to give a yellow whitesolid in 48% yield (1.5 g). $^{13}$C NMR (125 MHz, $CDCl_3$): δ 146.9, 144.6,125.6, 119.4;$^{1}$H NMR (400 MHz, $CDCl_3$): δ 8.24 (dm, 2H, J= 8.9 Hz), 7.14 (dm, 2H, J= 9.0 Hz).

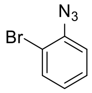

**1-Azido-2-bromobenzene**.[12](0.02 mol scale) Synthesized according to general procedure B. The product was purified by column chromatography using hexanes as eluent to give a light yellow liquid in 90 % yield. $^{13}$C NMR (125 MHz, $CDCl_3$): δ 138.7, 134.0, 128.7, 126.1, 119.5, 114.0; $^{1}$H NMR (400 MHz, $CDCl_3$): δ 7.55 (dd, 1H, J= 8.0, 1.1 Hz), 7.34 (app t, 1H, J= 7.6 Hz), 7.17 (dd, 1H, J= 8.0, 1.1 Hz), 7.01 (app t, 1H, J= 7.6 Hz).

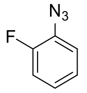

**1-Azido-2-fluorobenzene**.[13](0.02 mol scale) Synthesized according to general procedure B.The product was purified by column chromatography using hexanes as eluent to give a light yellow liquid in 68% yield.$^{1}$H NMR (400 MHz, $CDCl_3$): δ 7.10 (m, 4H, J= 8.0); $^{13}$C NMR (100 MHz, $CDCl_3$): δ 154.9 (d, J = 249 Hz), 127.9 (d, J = 11 Hz), 125.8 (d, J = 7 Hz), 124.9 (d, J = 4 Hz), 121.0, 116.7 (d, J = 19 Hz); $^{19}$F NMR (376 MHz, $CDCl_3$): δ -126.25 (s).

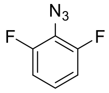

**1-azido-2,6-difluorobenzene**.[13](0.02 mol scale) Synthesized according to general procedure B.The product was purified by column chromatography using hexanes as eluent to give white crystals in 33% yield. $^{1}$H NMR (400 MHz, $CDCl_3$): δ 7.02 (m, 1 H), 6.89 (m, 2 H); $^{13}$C NMR (100 MHz, $CDCl_3$): δδ 155.8 (dd, J = 4, 250 Hz), 124.8 (t, J = 9 Hz), 117.5 (t, J = 14 Hz), 112.1 (dd, J = 5, 18 Hz); $^{19}$F NMR (376 MHz, $CDCl_3$): δ-122.80 (s, 2F).

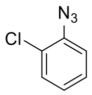

**1-Azido-2-chlorobenzene**.[14](0.02 mol scale) Synthesized according to general procedure C.The product was purified by column chromatography using pentane as eluent to give a light yellow liquid in 88% yield. $^{1}$H NMR (400 MHz, $CDCl_3$): δ 7.31 (d, 2H, J = 8.7 Hz), 6.96 (d, 2H, J = 8.7 Hz).$^{13}$C NMR (100 MHz, $CDCl_3$): δ 138.7, 130.2, 129.8, 120.3.

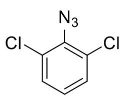

**1-Azido-2,6-dichlorobenzene**.[15](0.02 mol scale) Synthesized according to general procedure B. The product was purified by column chromatography using pentane as eluent to give a light yellow oil in 67% yield. $^{1}$H NMR (400 MHz, $CDCl_3$): δ 7.30 (d, 2H, J = 8.2 Hz), 7.05 (t, 1H, J = 8.2 Hz); $^{13}$C NMR (125 MHz, $CDCl_3$): δ 134.2, 129.6, 129.0, 126.4.

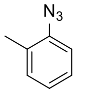


**1-Azido-2-methylbenzene**.[16](0.02 mol scale) Synthesized according to general procedure C. The product was purified by column chromatography using pentane as eluent to give a light yellow liquid in 81% yield.$^1$H NMR (400 MHz, $CDCl_3$): δ 7.26 (t, 1H, J = 7.4 Hz), 7.19 (d, 1H, J = 7.3 Hz), 7.15 (d, 1H, J = 7.3 Hz),7.07 (t, 1H, J = 7.4 Hz);$^{13}$C NMR (100 MHz, $CDCl_3$): δ 138.4, 131.1, 129.6, 127.1, 124.6, 117.9, 17.2.

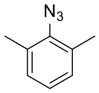


**1-Azido-2,6-dimethylbenzene**.[15](0.02 mol scale) Synthesized according to general procedure C. The product was purified by column chromatography using hexanes as eluent to give a light yellow liquid in 86% yield. $^1$H NMR (400 MHz, $CDCl_3$): δ 7.02 (m, 3H), 2.38 (s, 6H); $^{13}$C NMR (100 MHz, $CDCl_3$): δ 137.0, 132.2, 128.9, 125.7, 18.2.

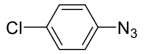


**1-Azido-4-chlorobenzene**.[15](0.02 mol scale) Synthesized according to general procedure C. The product was purified by column chromatography using pentane as eluent to give a light yellow liquid in 88% yield. $^1$H NMR (400 MHz, $CDCl_3$): δ 7.31 (app d, 2H, J = 8.6 Hz), 6.98(app d, 2H, J = 8.6 Hz); $^{13}$C NMR (100 MHz, $CDCl_3$): δ 138.7, 130.2, 129.9, 120.3.

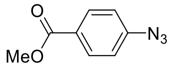


**Methyl 4-azidobenzoate**.[17](0.02 mol scale) Synthesized according to general procedure C. The product was purified by column chromatography using hexanes:EtOAc as eluent to give a pale solid in 62 % yield. $^1$H NMR (400 MHz, $CDCl_3$): δ8.03 (app d, 2H, J = 8.6 Hz), 7.05 (app d,2H, J = 8.6 Hz), 3.90 (s, 3 H); $^{13}$C NMR (100 MHz, $CDCl_3$): δ 166.3, 144.7, 131.4, 126.7, 118.9, 52.2.

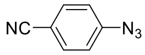


**4-Azidobenzonitrile**.[18](0.02 mol scale) Synthesized according to general procedure C. The product was purified by column chromatography using hexanes:EtOAc (9:1) as eluent to give a pale yellow solid in 80% yield. $^1$H NMR (400 MHz, $CDCl_3$): δ 7.02 (m, 3H), 2.38 (s, 6H); $^{13}$C NMR (100 MHz, $CDCl_3$): δ 137.0, 132.2, 128.9, 125.7, 18.2.

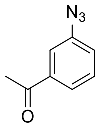


**3-Azido-acetophenone**.[19](0.02 mol scale) Synthesized according to general procedure C.The product was purified by column chromatography using hexanes:EtOAc 9:1 as eluent to give anorange liquid in 70 % yield. $^1$H NMR (400 MHz, $CDCl_3$): δ 7.70 (app d, 1H, J = 7.7 Hz), 7.60 (app t, 1H, J = 1.9 Hz), 7.44 (t, 1H, J = 7.7 Hz), 7.23 (app dd, 1H, J = 7.7, 1.9 Hz), 2.62 (s, 3H); $^{13}$C NMR (100 MHz, $CDCl_3$): δ 197.1, 140.9, 138.7, 130.0, 124.9, 123.5, 118.5, 26.7.

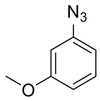


**3-Azido-acetophenone**.[14](0.02 mol scale) Synthesized according to general procedure C. Extracted with diethyl ether instead of EtOAc. The product was purified by column chromatography using pentane as eluent to give a light yellow oil in 80% yield. $^1$H NMR (400 MHz, $CDCl_3$): δ 7.26 (t, 1H, J = 8.2 Hz), 6.72 (dd, 1H, J = 8.2, 2.0 Hz), 6.65 (dd, 1H, J = 8.2, 2.0 Hz), 6.56 (t, 1H, J= 2.0 Hz), 3.81 (s, 3H); $^{13}$C NMR (100 MHz, $CDCl_3$): δ 160.8, 141.3, 130.4, 111.3, 110.7, 104.9, 55.4.

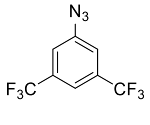


**1-Azido-3,5-bis(trifluoromethyl)benzene**.[20](0.02 mol scale) Synthesized according to general procedure C.The product was purified by column chromatography using pentane

as eluent to give a pale yellow liquid in 65% yield. $^{1}$H NMR (400 MHz, $CDCl_3$): δ 7.65 (s, 1H), 7.45 (s, 2H); $^{13}$C NMR (100 MHz, $CDCl_3$): δ 142.4 (1C), 133.1 (2C, q, J= 34 Hz), 122.8 (2C*F*$_3$, q, J= 271 Hz), 119.1 (2C, m), 118.3 (C, m); $^{19}$F NMR (376 MHz, $CDCl_3$): δ -63.17 (m, 6F).

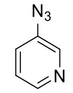

**3-Azidopyridine**.[21](0.02 mol scale) Synthesized according to **general procedure C**. The product was purified by column chromatography using hexanes:EtOAc 1:4 as eluent to give an amber liquid in 69% yield. $^{1}$H NMR (400 MHz, $CDCl_3$): δ 8.35(app d, 1H, J = 4.5 Hz), 8.31(d, 1H, J = 2.1 Hz), 7.26(dm, 1H, J = 8.1 Hz), 7.22 (m, 1H); $^{13}$C NMR (100 MHz, $CDCl_3$): δ 146.0, 141.3, 137.1, 125.9, 124.1.

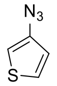

**3-Azidothiophene**.[22]Synthesized using a modified version of a reported procedure.[23] To a solution of 3-bromothiophene (0.03 mmol) in anhydrous ether (80 mL) at -70°C under nitrogen, was added *n*-butyl lithium (24 mL, 1.6 M solution in hexane)while stirring. The mixture was stirred for 30 min at -70°C, to which was added *p*-toluenesulfonyl azide (0.07 mol) dropwise. The reaction mixture was stirred for 3 h at -70°C, after which the temperature was allowed to increase to -40°C over 1 h.A solution of ethylenediaminetetraacetic acid disodium salt (11.4 g, 0.05 mol) in water (100 mL), was added, whilemaintaining the temperature below 0 °C. After stirring for 15 min at 0°C, the reaction mixture was allowed to reach rt, and stirred for another 12 h. The mixture was extracted with ether and washed with water, dried over $MgSO_4$, filtered, and evaporated. The red-brown residue was purified by column chromatography using pentaneas eluent to give anamber liquid in 22% yield. $^{1}$H NMR (400 MHz, $CDCl_3$): δ 7.30 (dd, 1H, J = 3.2, 5.2 Hz), 6.82 (dd, 1H, J = 5.2, 1.5 Hz), 6.80 (dd, 1H, J= 3.2, 1.5 Hz); $^{13}$C NMR (100 MHz, $CDCl_3$): δ 137.7, 126.7, 120.6, 109.8.

**Synthesis of products**

**General procedure A** (Table 1, Figure 1).In amicrowave vial (2 mL) charged with azide (1.0 mmol) and aldehyde (1.1 mmol) in DMSO (2.0 mL), was added base (0.1 mmol) and the reaction mixture was stirred at elevated temperatureunder microwave irradiation for 0.5-2 h. The crude reaction mixture was added to aqueous $NH_4Cl$solution (20 mL)andtheaqueouslayerwasextractedwithEtOAc (3x30mL).The combined organic layers were washed with water and then dried over $Na_2SO_4$, filtered and concentrated. The products were obtained by column chromatography (silica gel, hexanes/EtOAc).

**General procedure B** (Figure 1). In a flask (10 mL) charged with azide (1.0 mmol) and aldehyde (1.2 – 1.5 mmol) in DMSO (2.0 mL), was added base (0.1 mmol) and the reaction mixture was stirred at rt. When NMR analysis (DMSO-*d*$_6$) indicated complete conversion, an aq. AcOH solution (2 mL, 1.5 M) was added dropwise and the reaction mixture was stirred for 1 h.The resulting solution was extracted with EtOAc (3 x 30 mL), and the combined organic layers were washed with 0.5 M aq. HCl (10 mL), dried over $Na_2SO_4$, filtered and concentrated. The products were obtained by column chromatography (silica gel, hexanes/EtOAc).

**General procedure C** (Table 2 and S1). In avial (10 mL) charged with azide (1.0 mmol) in THF (1 mL) and*t*-BuOH (0.5 mL), *t*-BuOK (1.2 – 2.0 mmol) was added and the reaction mixture was stirred vigorously at room temperature.Afterwards, aldehyde (4 mmol) in THF (0.5 mL) was added dropwise within 0.5 - 1 minute. After the reaction time (1-5 minutes), the mixture wasquickly quenched by 2mL

aq. AcOH (1.5 M) and stirred for another 1 hours.The mixture was then extracted with ethyl acetate (3 x 30 mL).The combined organic layers were then dried over $Na_2SO_4$, filtered and concentrated. Amides were obtained by column chromatography (silica gel, hexanes/EtOAc).

**General procedure D** (Figure 2). In aflask (5 mL) charged with azide (1.0 mmol) and aldehyde (1.1 mmol) in DMSO (2.0 mL), was added base (0.1 mmol) and the reaction mixture was stirred at room temperature while the flask remained open. After complete conversion, as indidcated by $^{19}$F-NMR spectroscopy, the reaction mixture was added to an aq.$NH_4Cl$ solution (20 mL), andextracted with ethylacetate (3 x 30 mL).The combined organic layers were washed with water and concentrated. The products were obtained by column chromatography (silica gel, hexanes/EtOAc).

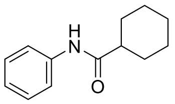

*N*-Phenylcyclohexanecarboxamide**(6a).**[24] White solid. $R_f$= 0.28 (hexanes/EtOAc= 5:1). $^1$H NMR (400 MHz, $CDCl_3$): $\delta_H$ 1.24 – 1.35 (m, 3H), 1.54 (quartet, 2H, C*H*, J = 12.3 Hz), 1.70 (m, 1H, C*H*), 1.83 (m, 2H), 1.96 (d, 2H, C*H*, J = 13.2 Hz), 2.23 (tt, 1H, C*H,* J = 11.6, 3.6 Hz), 7.09 (t, 1H, J = 7.5 Hz), 7.10 (br. s, 1H, N*H*), 7.31 (t, 2H, J = 7.5 Hz), 7.52 (d, 2H, J= 7.5 Hz ); $^{13}$C NMR (100 MHz, $CDCl_3$): δ25.8 (3C), 29.7 (2C), 46.7, 119.8, 119.9, 124.2, 129.1, 138.2, 174.4.

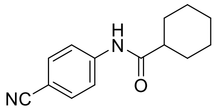

*N*-(4-Cyanophenyl)cyclohexanecarboxamide(**6b**). White powder. $R_f$ = 0.20 (hexanes/EtOAc= 4:1). $^1$H NMR (400 MHz, $CDCl_3$): $\delta_H$ 1.21 – 1.39 (m, 3H), 1.52 (quartet, 2H, C*H*, J = 12.8 Hz), 1.72 (m, 1H, C*H*), 1.84 (m, 2H, C*H₂*), 1.96 (d, 2H, C*H*, J = 13.0 Hz), 2.26 (tt, 1H, C*H,* J = 3.5, 11.8 Hz), 7.34 (br. s, 1H, N*H*), 7.60 (d, 1H, J = 8.6 Hz), 7.67 (d, 1H, J = 8.6 Hz); $^{13}$C NMR (100 MHz, $CDCl_3$): δ25.7 (3C), 29.7 (2C), 46.8, 107.1, 119.0, 119.6, 133.4, 142.3, 174.8. ESI-HRMS: Calcd. for $C_{14}H_{17}N_2O$ $[M+H]^+$: 229.1335, found 229.1340. IR(ATR), see attached spectrum.

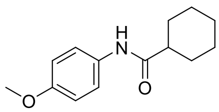

*N*-(4-Methoxyphenyl)cyclohexanecarboxamide(**6c**).[35] White solid. $R_f$ = 0.21 (hexanes/EtOAc= 4:1). $^1$H NMR (400 MHz, $CDCl_3$): $\delta_H$ 1.23 – 1.33 (m, 3H), 1.53 (quartet, 2H, C*H*, J = 12.8 Hz), 1.72 (m, 1H, C*H*), 1.84 (m, 2H), 1.95 (d, 2H, C*H*, J = 13.2 Hz), 2.20 (tt, 1H, C*H,* J = 3.4, 12.0 Hz), 3.78 (s, 3H, OC*H₃*),6.85 (d, 2H, J = 8.2 Hz), 7.06 (br. s, 1H, N*H*), 7.42 (d, 2H, J = 8.2 Hz); $^{13}$C NMR (100 MHz, $CDCl_3$): δ25.9 (3C), 29.9 (2C), 46.6, 55.6, 114.3, 121.8, 131.3, 156.4, 174.2.

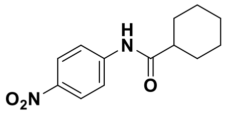

*N*-(4-Nitrophenyl)cyclohexanecarboxamide(**6d**).[30]Yellow powder. $R_f$ = 0.36 (hexanes/EtOAc= 3:1). $^1$H NMR (400 MHz, $CDCl_3$): $\delta_H$ 1.31 (m, 3H), 1.54 (quartet, 2H, C*H*, J = 12.8 Hz), 1.71 (m, 1H, C*H*), 1.85 (m, 2H, C*H₂*), 1.98 (d, 2H, C*H*, J = 13.2 Hz), 2.29 (tt, 1H, C*H,* J = 3.4, 12.0 Hz), 7.54 (br. s, 1H, N*H*), 7.72 (d, 1H, J = 9.0 Hz), 8.20 (d, 1H, J = 9.0 Hz); $^{13}$C NMR (100 MHz, $CDCl_3$): δ25.7 (3C), 29.7 (2C), 46.8, 119.1, 125.2, 143.5, 144.1, 174.9.

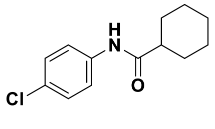

*N*-(4-Chlorophenyl)cyclohexanecarboxamide(**6e**).[31]White powder. $R_f$ = 0.43 (hexanes/EtOAc= 9:1). $^1$H NMR (400 MHz, $CDCl_3$): $\delta_H$ 1.22 – 1.35 (m, 3H), 1.52

(quartet, 2H, C*H*, J = 12.8 Hz), 1.70 (m, 1H, C*H*), 1.83 (m, 2H, $CH_2$), 1.94 (d, 2H, C*H*, J = 13.0 Hz), 2.22 (tt, 1H, C*H,* J = 3.5, 11.6 Hz), 7.26 (br. s, 1H, N*H*), 7.26 (d, 1H, J = 9.0 Hz), 7.47 (d, 1H, J = 9.0 Hz); $^{13}$C NMR (100 MHz, $CDCl_3$): δ25.8 (3C), 29.8 (2C), 46.6, 121.2, 129.1, 129.2, 136.8, 174.5.

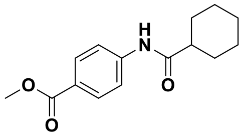


Methyl 4-(cyclohexanecarboxamido)benzoate(**6f**).[32] White powder. $R_f$ = 0.20 (hexanes/EtOAc= 4:1). $^1$H NMR (400 MHz, $CDCl_3$): $\delta_H$ 1.22 – 1.33 (m, 3H), 1.54 (quartet, 2H, C*H*, J = 12.8 Hz), 1.71 (m, 1H, C*H*), 1.83 (m, 2H, $CH_2$), 1.96 (d, 2H, C*H*, J = 13.0 Hz), 2.25 (tt, 1H, C*H,* J = 3.5, 11.6 Hz), 3.89 (s, 3H, $OCH_3$), 7.38 (br. s, 1H, N*H*), 7.61 (d, 1H, J = 8.7 Hz), 7.99 (d, 1H, J = 8.7 Hz); $^{13}$C NMR (100 MHz, $CDCl_3$): δ25.8 (3C), 29.7 (2C), 46.8, 52.1, 118.9, 125.6, 131.0, 142.4, 166.8, 174.7.

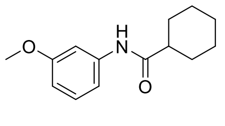


*N*-(3-Methoxyphenyl)cyclohexanecarboxamide(**6g**). White solid. $R_f$ = 0.47 (hexanes/EtOAc= 3:1). $^1$H NMR (400 MHz, $CDCl_3$): $\delta_H$ 1.22 – 1.40 (m, 3H), 1.54 (app quartet, 2H, C*H*, J = 12.8 Hz), 1.71 (m, 1H, C*H*), 1.84 (m, 2H), 1.96 (app d, 2H, C*H*, J = 13.2 Hz), 2.22 (tt, 1H, C*H,* J = 3.4, 11.8 Hz), 3.80 (s, 3H, $OCH_3$),6.65 (app d, 1H, J = 8.3 Hz), 6.94 (app d, 1H, J = 7.8 Hz), 7.14 (br. s, 1H, N*H*), 7.19 (t, 1H, J = 8.1 Hz), 7.37 (s, 1H); $^{13}$C NMR (100 MHz, $CDCl_3$): δ25.8 (3C), 29.8 (2C), 46.7, 55.4, 105.4, 110.2, 111.9, 129.7, 139.6, 160.2, 174.7; ESI-HRMS: Calcd. for $C_{14}H_{20}NO_2$ $[M+H]^+$: 234.1494, found 234.1502. IR(ATR), see attached spectrum.

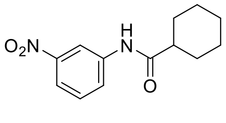


*N*-(3-Nitrophenyl)cyclohexanecarboxamide(**6h**).[33]Yellow powder. $R_f$ = 0.50 (hexanes/EtOAc= 4:1). $^1$H NMR (400 MHz, $CDCl_3$): $\delta_H$ 1.21 – 1.40 (m, 3H), 1.55 (quartet, 2H, C*H*, J = 12.8 Hz), 1.72 (m, 1H, C*H*), 1.85 (dm, 2H, C*H*, J = 12.8 Hz), 1.97 (d, 2H, C*H*, J = 13.2 Hz), 2.29 (tt, 1H, C*H,* J = 3.4, 11.8 Hz), 7.47 (t, 1H, J = 8.1 Hz), 7.54 (br. s, 1H, N*H*), 7.93 (dd, 1H, J = 8.2, 1.5 Hz), 7.96 (dd, 1H, J = 8.2, 1.5 Hz), 8.38 (t, 1H, J = 2.0 Hz) ; $^{13}$C NMR (100 MHz, $CDCl_3$): δ25.7 (3C), 29.7 (2C), 46.6, 114.6, 118.8, 125.6, 130.0, 139.4, 148.7, 174.9.

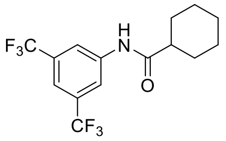


*N*-(3,5-Bis(trifluoromethyl)phenyl)cyclohexanecarboxamide(**6i**).[34] White solid. $R_f$ = 0.57 (hexanes/EtOAc= 4:1). $^1$H NMR (400 MHz, $CDCl_3$): $\delta_H$ 1.21 – 1.40 (m, 3H), 1.55 (quartet, 2H, C*H*, J = 12.8 Hz), 1.72 (m, 1H, C*H*), 1.86 (m, 2H), 1.96 (d, 2H, C*H*, J = 13.2 Hz), 2.27 (tt, 1H, C*H,* J = 3.4, 11.8 Hz), 7.49 (br. s, 1H, N*H*), 7.58 (s, 1H), 8.05 (s, 1H); $^{13}$C NMR (100 MHz, $CDCl_3$): δ25.7 (3C), 29.7 (2C), 46.6, 117.5 (m), 119.5 (m), 123.2 (quartet, 1C, $J_{CF}$ = 272 Hz, *C*$F_3$),132.5 (quartet, 1C, $J_{CF}$ = 33 Hz),139.6, 174.9; $^{19}$F NMR (376 MHz, $CDCl_3$): δ-63.07 (m, 6F); ESI-HRMS: Calcd. for $C_{15}H_{16}F_6NO$ $[M+H]^+$: 340.1136, found 340.1134. IR(ATR), see attached spectrum.

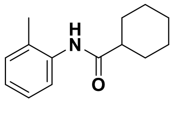


*N*-(*o*-Tolyl)cyclohexanecarboxamide**(6j).**[27] White powder.$R_f$ = 0.50 (hexanes/EtOAc= 3:1). $^1$H NMR (400 MHz, $CDCl_3$): $\delta_H$ 1.22 – 1.34 (m, 3H), 1.55 (quartet, 2H, C*H*, J = 12.8 Hz), 1.70 (m, 1H, C*H*), 1.82 (m, 2H), 1.99 (d, 2H, C*H*, J = 13.2 Hz), 2.22 – 2.30 (m, 4H, C*H* &$CH_3$), 6.95 (br. s, 1H, N*H*), 7.04 (t, 1H, J = 7.5 Hz), 7.14 – 7.20 (m, 2H), 7.82 (d, 1H, J = 7.9 Hz); $^{13}$C NMR (100 MHz, $CDCl_3$): δ 17.9, 25.9, 30.0, 46.6, 123.2, 125.1, 126.9, 128.9, 130.5, 135.9, 174.3.

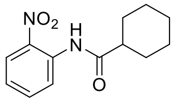

*N*-(2-Nitrophenyl)cyclohexanecarboxamide **(6k).**[25]Yellow powder. $R_f$ = 0.42 (hexanes/EtOAc= 4:1). $^1$H NMR (400 MHz, $CDCl_3$): $\delta_H$ 1.25 – 1.42 (m, 3H), 1.55 ( quartet, 2H, C*H*, J = 12.8 Hz), 1.72 (m, 1H), 1.85 (dm, 2H, C*H*, J = 12.8 Hz), 2.03 (d, 2H, C*H*, J = 13.2 Hz), 2.36 (tt, 1H, C*H,* J = 3.4, 11.6 Hz), 7.16 (t, 1H, J = 7.7 Hz), 7.63 (t, 1H, J = 7.7 Hz), 8.21 (dd, 1H, J = 8.4, 1.3 Hz),8.82 (d, 1H, J = 8.4 Hz), 10.44 (br. s, 1H, N*H*); $^{13}$C NMR (100 MHz, $CDCl_3$): δ25.7, 25.8, 29.7, 47.3, 122.4, 123.1, 125.9, 135.4, 136.1, 136.4, 175.4.

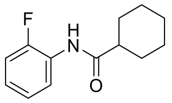

*N*-(2-Fluorophenyl)cyclohexanecarboxamide(**6l**).[28] White powder. $R_f$ = 0.28 (hexanes/EtOAc= 9:1). $^1$H NMR (400 MHz, $CDCl_3$): $\delta_H$ 1.24 – 1.36 (m, 3H), 1.55 (quartet, 2H, C*H*, J = 12.8 Hz), 1.72 (m, 1H, C*H*), 1.84 (dm, 2H, C*H*, J = 12.8 Hz), 1.97 (d, 2H, C*H*, J = 13.2 Hz), 2.28 (tt, 1H, C*H,* J = 3.5, 12.0 Hz), 7.05 (m, 3H), 7.38 (br. s, 1H, N*H*), 8.35 (t, 1H, J = 9.5 Hz) ; $^{13}$C NMR (100 MHz, $CDCl_3$): δ25.76, 25.79, 29.8, 46.7, 114.8 (d, $J_{CF}$ = 19 Hz), 121.8, 124.1 (d, $J_{CF}$ = 8 Hz), 124.7 (d, $J_{CF}$ = 4 Hz), 126.7 (d, $J_{CF}$ = 10 Hz), 152.4 (d, $J_{CF}$ = 242 Hz), 174.5. $^{19}$F NMR (376 MHz, $CDCl_3$): δ-131.96 (m).

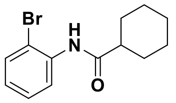

*N*-(2-Bromophenyl)cyclohexanecarboxamide**(6m).**[26]White powder. $R_f$ = 0.35 (hexanes/EtOAc= 12:1). $^1$H NMR (400 MHz, $CDCl_3$): $\delta_H$ 1.22 – 1.36 (m, 3H), 1.55 (app quartet, 2H, C*H*, J = 12.8 Hz), 1.72 (m, 1H, C*H*), 1.86 (app dm, 2H, C*H*, J = 12.8 Hz), 2.03 (d, 2H, C*H*, J = 13.2 Hz), 2.32 (tt, 1H, C*H,* J = 3.4, 12.0 Hz), 6.96 (td, 1H, J = 1.5, 7.8 Hz), 7.31 (t, 1H, J = 7.8 Hz), 7.52 (dd, 1H, J = 8.1, 1.2 Hz),7.70 (br. s, 1H, N*H*), 8.38 (d, 1H, J = 8.2 Hz) ; $^{13}$C NMR (100 MHz, $CDCl_3$): δ25.8, 25.9, 29.8, 46.8, 122.0, 125.1, 128.6, 132.3, 136.0, 174.4.

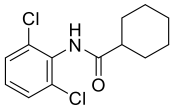

*N*-(2,6-Dichlorophenyl)cyclohexanecarboxamide(**6n**).[29] White powder. $R_f$ = 0.16 (hexanes/EtOAc= 9:1). $^1$H NMR (400 MHz, $CDCl_3$): $\delta_H$ 1.22 – 1.36 (m, 3H), 1.55 (m, 2H), 1.72 (m, 1H, C*H*), 1.86 (dm, 2H, C*H*, J = 12.8 Hz), 2.04 (d, 2H, C*H*, J = 13.2 Hz), 2.34 (t, 1H, C*H,* J = 12.0 Hz), 6.93 (br. s, 1H, N*H*), 7.16 (t, 1H, J = 8.0 Hz), 7.36 (d, 2H, J = 8.0 Hz); $^{13}$C NMR (100 MHz, $CDCl_3$): δ25.8, 25.9, 29.8, 45.7, 128.4, 128.5, 132.3, 133.8, 174.1.

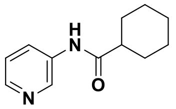

*N*-(Pyridin-3-yl)cyclohexanecarboxamide(**6p**).[36]White solid. Yield > 95%. $R_f$ = 0.17 (hexanes/EtOAc= 1:1). $^1$H NMR (400 MHz, $CDCl_3$): $\delta_H$ 1.21 – 1.39 (m, 3H), 1.55 (quartet, 2H, C*H*, J = 12.8 Hz), 1.71 (m, 1H, C*H*), 1.83 (m, 2H), 1.95 (d, 2H, C*H*, J = 13.2 Hz), 2.29 (tt, 1H, C*H,* J = 11.6, 3.3 Hz), 7.27 (dd, 1H, J = 8.0, 1.4 Hz), 7.74 (br. s, 1H, N*H*), 8.24 (dd, 1H, Ar-H, J = 8.0, 1.4 Hz), 8.32 (s, 1H), 8.57 (s, 1H); $^{13}$C NMR (100 MHz, $CDCl_3$): δ25.7 (3C), 29.7 (2C), 46.5, 123.9, 127.5, 135.4, 140.9, 144.8, 175.2.

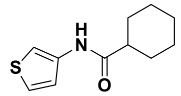

*N*-(Thiophen-3-yl)cyclohexanecarboxamide(**6q**).White solid. $R_f$ = 0.33 (hexanes/EtOAc= 4:1). $^1$H NMR (400 MHz, $CDCl_3$): $\delta_H$ 1.22 – 1.39 (m, 3H), 1.52 (quartet, 2H, C*H*, J = 12.8 Hz), 1.71 (m, 1H, C*H*), 1.83 (m, 2H), 1.94 (d, 2H, C*H*, J = 13.2 Hz), 2.22 (tt, 1H, C*H,* J = 3.4, 11.8 Hz), 7.49 (br. s, 1H, N*H*), 6.99 (dd, 1H, Ar-H, J = 5.2, 1.2 Hz), 7.21 (dd, 1H, Ar-H, J = 5.2, 3.2 Hz), 7.58 (br.s, 1H, N*H*), 7.59 (m, 1H, N*H*); $^{13}$C NMR (100 MHz, $CDCl_3$): δ25.8 (3C), 29.8 (2C), 46.1, 110.1, 121.1, 124.6, 135.8, 173.7; ESI-HRMS: Calcd. for $C_{11}H_{16}NOS$ $[M+H]^+$: 210.0953, found 210.0955. IR(ATR), see attached spectrum.

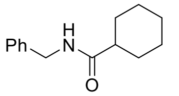

*N*-Benzylcyclohexanecarboxamide (**6s**).[37] White powder. $R_f$= 0.2 (hexanes/EtOAc= 4:1). $^1$H NMR (400 MHz, $CDCl_3$): $\delta_H$ 1.18 – 1.30 (m, 3H), 1.47 (m, 2H), 1.67 (m, 1H, C*H*), 1.78 (dm, 2H, C*H*, J = 12.8 Hz), 1.87 (d, 2H, C*H*, J = 13.2 Hz), 2.11 (tt, 1H, C*H*, J = 3.6, 12.0 Hz), 5.85 (br. s, 1H, N*H*), 7.30 (m, 5H); $^{13}$C NMR (100 MHz, $CDCl_3$): δ25.9, 29.8, 43.5, 45.7, 127.5, 127.8, 128.8, 138.7, 176.1.

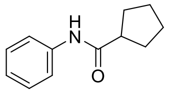

*N*-Phenylcyclopentanecarboxamide (**6t**).[40] White powder. $R_f$ = 0.17 (hexanes/EtOAc= 9:1). $^1$H NMR (400 MHz, $CDCl_3$): $\delta_H$ 1.61 (m, 2H), 1.79 (m, 2H), 1.92 (m, 4H), 2.68 (quintet, 1H, C*H*, J = 8.3 Hz), 7.09 (t, 1H, J = 7.5 Hz), 7.16 (br. s, 1H, N*H*), 7.31 (t, 2H, J = 7.5 Hz), 7.52 (d, 2H, J = 7.5 Hz) ; $^{13}$C NMR (100 MHz, $CDCl_3$): δ26.2, 30.7, 47.1, 119.8, 124.2, 129.1, 138.3, 174.7.

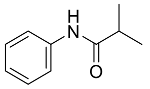

*N*-Phenylisobutyramide(**6v**).[38] White powder. $R_f$= 0.33 (hexanes/EtOAc= 3:1). $^1$H NMR (400 MHz, $CDCl_3$): $\delta_H$ 1.25 (d, 6H, C*H*$_3$, J = 7.4 Hz), 2.51 (m, 1H, C*H*), 7.09 (t, 1H, J = 7.9 Hz), 7.23 (br. s, 1H, N*H*), 7.31 (t, 2H, J = 7.9 Hz), 7.53 (d, 2H, J = 7.9 Hz); $^{13}$C NMR (100 MHz, $CDCl_3$): δ 19.8, 36.9, 119.9, 124.3, 129.1, 138.2, 175.4.

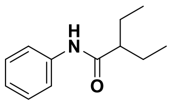

2-Ethyl-*N*-phenylbutanamide(**6w**).[39] White powder. $R_f$ = 0.26 (hexanes/EtOAc= 20:3). $^1$H NMR (400 MHz, $CDCl_3$): $\delta_H$ 0.96 (t, 6H, $CH_3$, J = 7.4 Hz), 1.57 (m, 2H, C*H*$_2$), 1.72 (m, 2H, C*H*$_2$), 2.03 (septet, 1H, C*H*, J = 4.9 Hz), 7.10 (t, 1H, J = 7.8 Hz), 7.15 (br. s, 1H, N*H*), 7.32 (t, 2H, J = 7.8 Hz), 7.55 (d, 2H, J = 8.1 Hz); $^{13}$C NMR (100 MHz, $CDCl_3$): δ12.3, 26.0, 52.7,120.0, 124.4, 129.1, 138.1, 174.3.

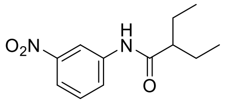

2-Ethyl-*N*-(3-nitrophenyl)butanamide(**6x**).[25a] White powder. $R_f$ = 0.42 (hexanes/EtOAc= 3:1). $^1$H NMR (400 MHz, $CDCl_3$): $\delta_H$ 0.97 (t, 6H, J = 7.4 Hz), 1.60 (m, 2H, C*H*$_2$), 1.73 (m, 2H, C*H*$_2$), 2.09 (septet, 1H, C*H*, J = 4.9 Hz), 7.41 (br. s, 1H, N*H*), 7.49 (t, 1H, J = 8.0 Hz), 7.96 (dd, 1H, J = 8.2, 1.6 Hz), 7.99 (d, 1H, J = 8.0 Hz), 8.40 (t, 1H, J = 1.6 Hz); $^{13}$C NMR (100 MHz, $CDCl_3$): δ12.2, 25.9, 52.6, 114.7, 119.0, 125.7, 130.0, 139.1, 148.7, 174.8; ESI-HRMS: Calcd. for $C_{12}H_{17}N_2O_3$ $[M+H]^+$: 237.1239, found 237.1247. IR(ATR), see attached spectrum.

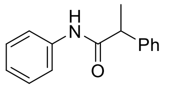

*N*,2-Diphenylpropanamide(**6y**).[41] White solid. $R_f$= 0.55 (hexanes/EtOAc= 3:1). $^1$H NMR (400 MHz, $CDCl_3$): $\delta_H$ 1.60 (d, 3H, C*H*$_3$, J = 7.1 Hz), 3.23 (quartet, 1H, C*H*), 7.07 (t, 1H, J = 7.5 Hz), 7.12 (br. s, 1H, N*H*), 7.25 – 7.28 (m, 2H), 7.30 – 7.33 (m, 1H), 7.36 – 7.42 (m, 6H); $^{13}$C NMR (100 MHz, $CDCl_3$): δ 18.7, 48.3, 119.8, 124.4, 127.7, 128.4, 129.0, 129.3, 138.0, 141.1, 172.4.

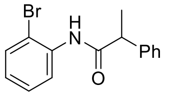

*N*-(2-Bromophenyl)-2-phenylpropanamide(**6z**).[42] pale yellow solid. $R_f$ = 0.25 (hexanes/EtOAc= 4:1). $^1$H NMR (400 MHz, $CDCl_3$): $\delta_H$ 1.65 (d, 3H, C*H*$_3$, J = 7.2 Hz), 3.80 (quartet, 1H, Ar-C*H*, J = 7.2 Hz), 6.92 (app t, 1H, Ar-*H*, J = 7.8 Hz), 7.28 (t, 1H, J = 7.8 Hz), 7.33 (m, 1H), 7.38-7.44 (m, 5H, Ar-*H*), 7.61 (br, 1H, N-*H*), 8.36 (d, 1H, J = 8.1 Hz); $^{13}$C NMR (100 MHz, $CDCl_3$): δ18.1, 48.6, 113.3, 121.5, 125.1, 128.0, 128.1, 128.4, 129.4, 132.3, 135.9, 140.4, 172.6.

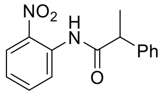

*N*-(2-Nitrophenyl)-2-phenylpropanamide(**6aa**). Yellowish solid. $R_f$ = 0.30 (hexanes/EtOAc = 6:1). $^1$H NMR (400 MHz, $CDCl_3$): $\delta_H$ 1.65 (d, 3H, C*H*$_3$, J = 7.1 Hz), 3.82 (quartet, 1H, Ar-C*H,* J = 7.1 Hz), 7.13 (t, 1H, Ar-*H,* J = 7.8 Hz), 7.30-7.46 (m, 5H, Ar-*H*), 7.61 (t, 1H, Ar-*H,* J = 7.8 Hz), 8.15 (dd, 1H, J = 8.6, 1.4 Hz), 8.80 (d, 1H, J = 8.6 Hz), 10.31 (s, 1H, N-*H*); $^{13}$C NMR (100 MHz, $CDCl_3$): δ18.1, 49.3, 122.1, 123.2, 125.8, 127.9, 128.1, 129.4, 135.2, 136.0, 136.3, 140.0, 173.6. ESI-HRMS: Calcd. for $C_{15}H_{15}N_2O_3$ $[M+H]^+$: 271.1077, found 271.1076. IR(ATR), see attached spectrum.

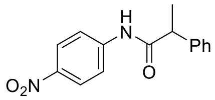

*N*-(4-Nitrophenyl)-2-phenylpropanamide(**6bb**).[30]Yellowish solid. $R_f$ = 0.31 (hexanes/EtOAc = 3:1). $^1$H NMR (400 MHz, $CDCl_3$): $\delta_H$ 1.60 (d, 3H, C*H*$_3$, J = 7.1 Hz), 3.76 (quartet, 1H, Ar-C*H,* J = 7.1 Hz), 7.33-7.40 (m, 5H, Ar-*H*), 7.49 (s, 1H, N-*H*), 7.61 (d, 1H, Ar-*H,* J = 9.1 Hz), 8.14 (d, 1H, J = 9.1 Hz); $^{13}$C NMR (100 MHz, $CDCl_3$): δ18.6, 48.4, 119.1, 125.1, 127.8, 128.1, 129.5, 140.3, 143.6, 143.8, 172.9.

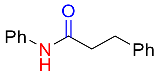

*N*,3-Diphenylpropanamide(**6cc**).[43] Colorless solid. $R_f$ = 0.23 (hexanes/EtOAc= 6:1). $^1$H NMR (400 MHz, $CDCl_3$): $\delta_H$ 2.66 (t, 2H, J = 7.7 Hz), 3.06 (t, 2H, J = 7.7 Hz), 7.08 (br. s, 1H, N*H*), 7.10 (t, 1H, J = 7.2 Hz), 7.24 (m, 3H), 7.30 (m, 4H), 7.43 (d, 2H, J = 7.2 Hz); $^{13}$C NMR (100 MHz, $CDCl_3$): δ31.7, 39.7, 120.0, 124.5, 126.6, 128.6, 128.8, 129.1, 137.9, 140.8, 170.5.

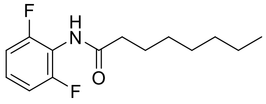

*N*-(2,6-Difluorophenyl)octanamide(**6dd**). Colorless solid. $R_f$ = 0.32 (hexanes/EtOAc= 4:1). $^1$H NMR (400 MHz, $CDCl_3$): $\delta_H$ 0.88 (t, 3H, J = 6.3 Hz), 1.31 (m, 8H), 1.73 (m, 2H), 2.4 (m, 2H), 6.80 (br. s, 1H, N*H*), 6.94 (t, 2H, J = 8.1 Hz), 7.19 (m, 1H); $^{13}$C NMR (100 MHz, $CDCl_3$): δ14.2, 22.7, 25.7, 29.1, 29.2, 31.7, 31.8, 36.6, 111.7, 111.9, 127.7, 158.1(d, 1C, $J_{CF}$ = 250 Hz), 171.7; $^{19}$F NMR (376 MHz, $CDCl_3$): δ-118.00 (m); ESI-HRMS: Calcd. for $C_{14}H_{20}NOF_2$ $[M+H]^+$: 256.1507, found 256.1509. IR(ATR), see attached spectrum.

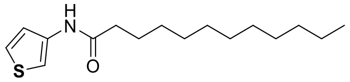

*N*-(Thiophen-3-yl)dodecanamide(**6ee**). White solid. $R_f$ = 0.47 (hexanes/EtOAc= 4:1). $^1$H NMR (400 MHz, $CDCl_3$): $\delta_H$ 0.88 (t, 3H, J = 6.3 Hz), 1.25 (m, 16H), 1.70 (quintet, 2H, J = 7.2 Hz), 2.33 (t, 2H, J = 7.2 Hz), 6.98 (dd, 1H, Ar-H, J = 5.1, 1.1 Hz), 7.22 (dd, 1H, Ar-H, J = 5.2, 3.2 Hz), 7.42 (br.s, 1H, N*H*), 7.57 (d, 1H, J = 2.2 Hz); $^{13}$C NMR (100 MHz, $CDCl_3$): δ14.3, 22.8, 25.8, 29.4, 29.5(2C), 29.6, 29.8(2C), 30.1, 37.4, 110.2, 131.0, 124.6, 135.7, 170.7; ESI-HRMS: Calcd. for $C_{16}H_{28}NOS$ $[M+H]^+$: 282.1866, found 282.1866. IR(ATR), see attached spectrum.

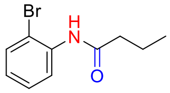

*N*-(2-Bromophenyl)butyramide(**6ff**).[44]Colorless solid. $R_f$ = 0.51 (hexanes/EtOAc= 4:1). $^1$H NMR (400 MHz, $CDCl_3$): $\delta_H$ 1.04 (t, 2H, J = 7.3 Hz), 1.79 (sextet, 2H, J = 7.3 Hz),2.41 (t, 2H, J = 7.3 Hz), 6.97 (t, 1H, J = 7.5 Hz), 7.31 (t, 1H, J = 7.5 Hz), 7.53 (d, 1H, J = 7.5 Hz), 7.62 (br. s, 1H, N*H*), 8.36 (d, 1H, J = 7.5 Hz); $^{13}$C NMR (100 MHz, $CDCl_3$):δ13.9, 19.2, 40.1, 113.3, 122.0, 125.2, 128.6, 132.3, 135.9, 171.3.

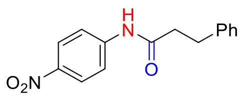

*N*-(4-Nitrophenyl)-3-phenylpropanamide(**6gg**).[45] Yellowish solid. $R_f$ = 0.19 (hexanes/EtOAc= 6:1). $^1$H NMR (400 MHz, $CDCl_3$): $\delta_H$ 2.73 (t, 2H, J = 7.7 Hz), 3.06 (t, 2H, J = 7.7 Hz), 7.23 (tm, 3H, J = 7.7 Hz), 7.30 (t, 1H, J = 7.7 Hz), 7.61 (br. s, 1H, N*H*), 7.62 (d, 2H, J = 9.3 Hz), 8.16 (d, 2H, J = 9.3 Hz); $^{13}$C NMR (100 MHz, $CDCl_3$):δ31.4, 39.6, 119.2, 125.2, 126.7, 128.4, 128.8, 140.2, 143.5, 143.8, 171.1.

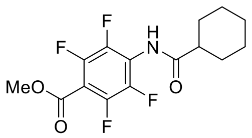

Methyl 4-(cyclohexanecarboxamido)-2,3,5,6-tetrafluorobenzoate(**6hh**).White solid. $R_f$ = 0.47 (hexanes/EtOAc= 3:1). $^1$H NMR (400 MHz, $CDCl_3$): $\delta_H$ 1.21 – 1.39 (m, 3H), 1.56 (quartet, 2H, C*H*, J = 12.8 Hz), 1.72 (m, 1H, C*H*), 1.89 (d, 2H, C*H*, J = 12.8 Hz), 1.99 (d, 2H, C*H*, J = 12.8 Hz), 2.40 (tt, 1H, C*H,* J = 3.5, 11.5 Hz), 3.97 (s, 3H, O$CH_3$), 7.09 (br, 1H, N*H*); $^{13}$C NMR (100 MHz, $CDCl_3$): δ 25.6, 25.7, 29.7, 45.4, 53.4, 109.9 (m), 119.7 (m), 142.1 (dm, 2C, $J_{CF}$ = 250 Hz), 145.1 (dm, 2C, $J_{CF}$ = 258 Hz), 160.2, 174.0; $^{19}$F NMR (376 MHz, $CDCl_3$): δ-139.60 (m, 2F), -144.32 (m, 2F).ESI-HRMS: Calcd. for $C_{15}H_{16}F_4NO_3$ $[M+H]^+$: 334.1066, found 334.1068. IR(ATR), see attached spectrum.

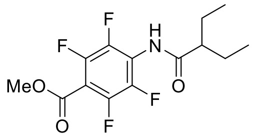

Methyl 4-(2-ethylbutanamido)-2,3,5,6-tetrafluorobenzoate(**6ii**). White powder. $R_f$ = 0.2 (hexanes/EtOAc= 6:1). $^1$H NMR (400 MHz, $CDCl_3$): $\delta_H$ 0.96 (t, 6H, J = 7.4 Hz), 1.60 (m, 2H, $CH_2$), 1.72 (m, 2H, $CH_2$), 2.23 (septet, 1H, C*H*, J = 5.0 Hz), 3.97 (s, 3H, OMe), 7.17 (br. s, 1H, N*H*); $^{13}$C NMR (100 MHz, $CDCl_3$): δ11.9, 25.9, 51.4, 53.4, 110.0 (m), 119.5 (m), 142.0 (dm, 2C, $J_{CF}$ = 250 Hz), 145.0 (dm, 2C, $J_{CF}$ = 250 Hz), 160.4, 174.2; $^{19}$F NMR (376 MHz, $CDCl_3$): δ-139.63 (m, 2F), -144.00 (m, 2F); ESI-HRMS: Calcd. for $C_{14}H_{16}F_4NO_3$ $[M+H]^+$: 322.1066, found 322.1071.IR (ATR), see attached spectrum.

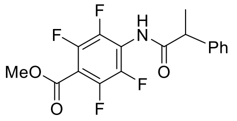

Methyl 2,3,5,6-tetrafluoro-4-(2-phenylpropanamido)benzoate**(6jj)**. White solid. $R_f$= 0.33 (3:1 hexanes/EtOAc). $^1$H NMR (400 MHz, $CDCl_3$): $\delta_H$ 1.59 (d, 3H, $CH_3$, J = 7.2 Hz), 3.84 (quartet, 1H, Ar-C*H*, J = 7.2 Hz), 3.95 (s, 3H, O$CH_3$), 7.03 (br, 1H, N*H*), 7.31 - 7.41 (m, 5H, Ar-*H*); $^{13}$C NMR (100 MHz, $CDCl_3$): δ 18.6, 47.3, 53.4, 109.9 (m), 119.5 (m), 127.8, 128.0, 129.5, 140.2, 142.0 (dm, 2C, $J_{CF}$ = 243 Hz), 144.7 (dm, 2C, $J_{CF}$ = 256 Hz), 159.8, 169.4; $^{19}$F NMR (376 MHz, $CDCl_3$): δ-139.54 (m, 2F), -144.20 (m, 2F);ESI-HRMS: Calcd. for $C_{17}H_{14}F_4NO_3$ $[M+H]^+$: 356.0901, found 356.0910.IR(ATR), see attached spectrum.

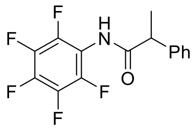

*N*-(Perfluorophenyl)-2-phenylpropanamide(**6kk**).[46]White solid. $R_f$ = 0.30 (10:1 hexanes/EtOAc). $^1$H NMR (400 MHz, $CDCl_3$): $\delta_H$ 1.60 (d, 3H, $CH_3$, J = 7.1 Hz), 3.82 (quartet, 1H, Ar-C*H,* J = 7.1 Hz), 6.77 (br, 1H, N*H*), 7.27-7.42 (m, 5H, Ar-*H*); $^{13}$C NMR (100 MHz, $CDCl_3$): δ 18.6, 47.2, 112.0 (m), 127.8, 128.0, 129.4, 140.2, 137.8 (dm, 2C, $J_{CF}$ = 247 Hz), 140.3 (dm, 1C, $J_{CF}$ = 232 Hz), 140.4, 143.1 (dm, 2C, $J_{CF}$ = 252 Hz), 173.0; $^{19}$F NMR (376 MHz, $CDCl_3$): δ-145.00 (m, 2F), -156.54 (m, 1F),-156.54 (m, 2F). IR(ATR), see attached spectrum.

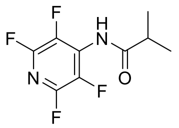

*N*-(Perfluoropyridin-4-yl)isobutyramide (**6ll**). White solid. $R_f$ = 0.25 (hexanes/EtOAc= 5:1). $^1$H NMR (400 MHz, $CDCl_3$): $\delta_H$ 1.30 (d, 6H, $CH_3$, J = 6.9 Hz), 2.70 (septet, 1H, C*H,* J = 6.9 Hz), 7.19 (s, 1H, N*H*); $^{13}$C NMR (100 MHz, DMSO): δ19.2, 34.0, 130.0 (m), 136.7 (dm, 2C, $J_{CF}$ = 258 Hz), 142.9 (dm, 2C, $J_{CF}$ = 240 Hz), 174.7; $^{19}$F NMR (376 MHz, DMSO):

δ-92.67 (m, 2F), -145.58 (m, 2F).ESI-HRMS: Calcd. for $C_9H_9F_4N_2O$ $[M+H]^+$: 237.0651, found 237.0648. IR(ATR), see attached spectrum.

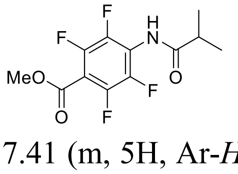

Methyl 2,3,5,6-tetrafluoro-4-isobutyramidobenzoate(**6mm**).White solid. $R_f$ = 0.24 (hexanes/EtOAc= 3:1). $^1$H NMR (400 MHz, $CDCl_3$): $δ_H$ 1.28 (d, 6H, C*H*3, J = 6.9 Hz), 2.67 (septet, 1H, C*H,* J = 6.9 Hz), 3.95 (s, 3H, OC*H*3), 7.03 (br, 1H, N*H*), 7.31 - 7.41 (m, 5H, Ar-*H*); $^{13}$C NMR (100 MHz, $CDCl_3$): δ 19.5, 35.9, 53.4, 110.0 (m), 119.7 (m), 142.1 (dm, 2C, $J_{CF}$ = 252 Hz), 145.1 (dm, 2C, $J_{CF}$ = 256 Hz), 159.8, 169.4; $^{19}$F NMR (376 MHz, $CDCl_3$): δ-139.60 (m, 2F), -144.42 (m, 2F);ESI-HRMS: Calcd. for $C_{12}H_{12}F_4NO_3$ $[M+H]^+$: 294.0753, found 294.0744.IR(ATR), see attached spectrum.

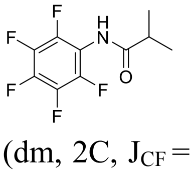

*N*-(Perfluorophenyl)isobutyramide(**6nn**). White solid. $R_f$= 0.44 (hexanes/EtOAc= 3:1). $^1$H NMR (400 MHz, $CDCl_3$): $δ_H$ 1.29 (d, 6H, C*H*3, J = 6.9 Hz), 2.65 (septet, 1H, C*H,* J = 6.9 Hz), 6.77 (br, 1H, N*H*); $^{13}$C NMR (100 MHz, $CDCl_3$): δ 19.6, 35.7, 112.1 (m), 137.8 (dm, 2C, $J_{CF}$ = 248 Hz), 140.3 (dm, 1C, $J_{CF}$ = 241 Hz), 143.2 (dm, 2C, $J_{CF}$ = 250 Hz), 175.7; $^{19}$F NMR (376 MHz, $CDCl_3$): δ-145.21 (m, 2F), -156.63 (m, 1F),-162.46 (m, 2F). ESI-HRMS: Calcd. for $C_{15}H_{16}NO$ $[M+H]^+$: 226.1232, found 226.1236. IR(ATR), see attached spectrum.

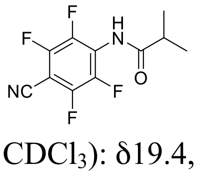

*N*-(4-Cyano-2,3,5,6-tetrafluorophenyl)isobutyramide(**6oo**).White solid. $R_f$ = 0.26 (hexanes/EtOAc= 3:1). $^1$H NMR (400 MHz, $CDCl_3$): $δ_H$ 1.28 (d, 6H, C*H*3, J = 6.9 Hz), 2.69 (septet, 1H, C*H,* J = 6.9 Hz), 7.43 (br, 1H, N*H*); $^{13}$C NMR (100 MHz, $CDCl_3$): δ19.4, 35.9, 91.2 (m, CN), 107.5 (m), 123.1 (m), 141.8 (dm, 2C, $J_{CF}$ = 254 Hz), 147.5 (dm, 2C, $J_{CF}$ = 261 Hz), 175.0; $^{19}$F NMR (376 MHz, $CDCl_3$): δ-132.78 (m, 2F), -141.80 (m, 2F);ESI-HRMS: Calcd. for $C_{11}H_9F_4N_2O$ $[M+H]^+$: 261.0651, found 261.0652. IR (ATR), see attached spectrum.

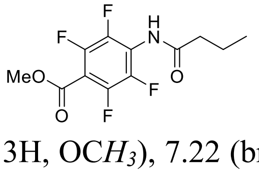

Methyl 4-butyramido-2,3,5,6-tetrafluorobenzoate (**6pp**).White solid. $R_f$ = 0.23 (hexanes/EtOAc= 9:1). $^1$H NMR (400 MHz, $CDCl_3$): $δ_H$ 1.02 (t, 3H, C*H*3, J = 7.4 Hz), 1.77 (sextet, 2H, C*H*2CH3, J = 7.4 Hz), 2.44 (t, 3H, C*H*3, J = 7.4 Hz), 3.97 (s, 3H, OC*H*3), 7.22 (br, 1H, N*H*); $^{13}$C NMR (100 MHz, $CDCl_3$): δ 13.6, 19.0, 38.3, 53.4, 110.1 (m), 119.5 (m), 142.1 (dm, 2C, $J_{CF}$ = 250 Hz), 145.1 (dm, 2C, $J_{CF}$ = 256 Hz), 160.2, 171.1; $^{19}$F NMR (376 MHz, $CDCl_3$): δ-139.52 (m, 2F), -144.15 (m, 2F).ESI-HRMS: Calcd. for $C_{12}H_{12}F_4NO_3$ $[M+H]^+$: 294.0753, found 294.0745. IR (ATR), see attached spectrum.

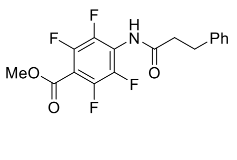

Methyl 2,3,5,6-tetrafluoro-4-(3-phenylpropanamido)benzoate (**6qq**).White solid. $R_f$ = 0.29 (hexanes/EtOAc = 3:1). $^1$H NMR (400 MHz, $CDCl_3$): $δ_H$ 2.76 (t, 2H, C*H*2, J = 7.5 Hz), 3.03 (t, 2H, Ar-C*H*2,J = 7.5 Hz), 3.96 (s, 3H, OC*H*3), 7.49 (br, 1H, N*H*), 7.19-7.26 (m, 3H, Ar-*H*), 7.27- 7.31 (m, 2H, Ar-*H*); $^{13}$C NMR (100 MHz, $CDCl_3$): δ 31.3, 38.0, 53.5, 109.9 (m), 119.3 (m), 126.7, 128.4, 128.8, 140.0, 142.0 (dm, 2C, $J_{CF}$ = 251 Hz), 145.0 (dm, 2C, $J_{CF}$ = 257 Hz), 160.2, 170.6; $^{19}$F NMR (376 MHz, $CDCl_3$): δ-139.46 (m, 2F), -143.80 (m, 2F);ESI-HRMS: Calcd. for $C_{17}H_{14}F_4NO_3$ $[M+H]^+$: 356.0910, found 356.0913. IR (ATR), see attached spectrum.

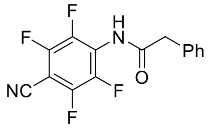

*N*-(4-Cyano-2,3,5,6-tetrafluorophenyl)-2-phenylacetamide**(6rr)**.White solid. $R_f = 0.23$ (3:1 hexanes/EtOAc). $^1$H NMR (400 MHz, $CDCl_3$): $\delta_H$ 3.80 (s, 2H, Ar-C*H₂*), 7.26-7.35 (m, 5H, Ar-*H*), 10.81 (s, 1H, N*H*); $^{13}$C NMR (100 MHz, $CDCl_3$): δ41.8, 90.2 (CN), 108.2 (m), 123.5 (m), 126.8, 128.4, 129.1, 134.9, 141.4 (dm, 2C, $J_{CF} = 250$ Hz), 145.0 (dm, 2C, $J_{CF} = 258$ Hz), 169.0; $^{19}$F NMR (376 MHz, $CDCl_3$): δ-130.25 (m, 2F), -137.63 (m, 2F);ESI-HRMS: Calcd. for $C_{15}H_9F_4N_2O$ $[M+H]^+$: 309.0651, found 309.0652. IR (ATR), see attached spectrum.

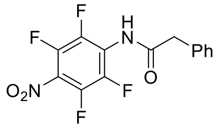

2-Phenyl-*N*-(2,3,5,6-tetrafluoro-4-nitrophenyl)acetamide**(6ss).** White powder. $R_f$ = 0.36 (3:1 hexanes/EtOAc). $^1$H NMR (400 MHz, DMSO): $\delta_H$ 3.80 (s, 1H, Ar-C*H₂*), 10.8 (br, 1H, N*H*), 7.27-7.35 (m, 5H, Ar-*H*); $^{13}$C NMR (100 MHz, DMSO): δ 41.7, 111.3 (m), 122.0 (m), 126.8, 128.4, 129.1, 134.9, 141.1 (dm, 2C, $J_{CF} = 257$ Hz), 141.7 (dm, 2C, $J_{CF}$ = 253 Hz), 169.0; $^{19}$F NMR (376 MHz, DMSO): δ-142.67 (m, 2F), -147.08(m, 2F). ESI-HRMS: Calcd. for $C_{14}H_9F_4N_2O_3$ $[M+H]^+$: 329.0549, found 329.0549. IR (ATR), see attached spectrum.

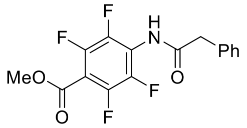

Methyl 2,3,5,6-tetrafluoro-4-(2-phenylacetamido)benzoate(**6tt**). White solid. $R_f$ = 0.30 (7:2 hexanes/EtOAc). $^1$H NMR (400 MHz, $CDCl_3$): $\delta_H$ 3.82 (s, 2H, Ar-C*H₂*), 3.96 (s, 3H, OC*H₃*), 7.10 (br, 1H, N*H*), 7.34 (m, 3H, Ar-*H*), 7.40 (m, 2H, Ar-*H*); $^{13}$C NMR (100 MHz, $CDCl_3$ &DMSO-$d_6$): δ42.6, 53.0, 109.0 (m), 120.2 (m), 126.8, 128.1, 128.4, 128.9, 134.4, 142.1 (dm, 2C, $J_{CF} = 240$ Hz), 145.1 (dm, 2C, $J_{CF} = 255$ Hz), 160.1, 172.3; $^{19}$F NMR (376 MHz, $CDCl_3$): δ-139.37(m, 2F), -143.99 (m, 2F);ESI-HRMS: Calcd. for $C_{16}H_{12}F_4NO_3$ $[M+H]^+$: 342.0748, found 342.0743. IR (ATR), see attached spectrum.

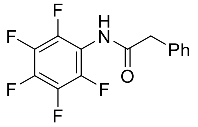

*N*-(Perfluorophenyl)-2-phenylacetamide(**6uu**).[47]White solid. $R_f$ = 0.42 (3:1 hexanes/EtOAc). $^1$H NMR (400 MHz, DMSO): $\delta_H$ 3.82 (s, 1H, Ar-C*H₂*), 10.3 (br, 1H, N*H*), 7.26-7.34 (m, 5H, Ar-*H*); $^{13}$C NMR (100 MHz, DMSO): δ 41.7, 112.9 (m), 126.0 (m), 126.7, 128.4, 129.0, 135.2, 137.3 (dm, 2C, $J_{CF} = 248$ Hz), 139.0 (dm, 1C, $J_{CF} = 243$ Hz), 142.6 (dm, 2C, $J_{CF} = 246$ Hz), 173.0; $^{19}$F NMR (376 MHz, $CDCl_3$): δ-145.40(m, 2F), -158.19 (m, 1F), -163.55 (m, 2F). IR (ATR), see attached spectrum.

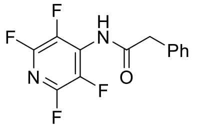

*N*-(Perfluoropyridin-4-yl)-2-phenylacetamide(**6vv**). White solid. $R_f$ = 0.24 (5:1 hexanes/EtOAc). $^1$H NMR (400 MHz, DMSO): $\delta_H$ 3.82 (s, 2H, Ar-C*H*), 7.26-7.35 (m, 5H, Ar-*H*), 10.99 (s, 1H, N*H*); $^{13}$C NMR (100 MHz, DMSO): δ41.8, 126.8, 128.4,129.1, 129.6 (m), 134.8, 136.6 (dm, 2C, $J_{CF} = 257$ Hz), 142.9 (dm, 2C, $J_{CF} = 240$ Hz), 168.7; $^{19}$F NMR (376 MHz, DMSO): δ-95.25 (m, 2F), -145.17 (m, 2F);ESI-HRMS: Calcd. for $C_{13}H_9F_4N_2O$ $[M+H]^+$: 285.0651, found 285.0646. IR (ATR), see attached spectrum.

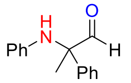

2-Phenyl-2-(phenylamino)propanal(**7**). Gray solid. $R_f$ = 0.68 (hexanes/EtOAc= 3:1). $^1$H NMR (400 MHz, $CDCl_3$): δ 1.85 (s, 3H), 5.22 (br. s, 1H, N*H*), 6.44 (d, 2H, J), 6.44 (d, 2H, J = 8.0 Hz), 6.67 (t, 1H, J = 7.3 Hz), 7.06 (t, 2H, J = 8.0 Hz), 7.36 (t, 1H, J = 7.3 Hz), 7.42 (t, 2H, J = 8.0 Hz), 7.50 (d, 2H, J = 7.3 Hz), 9.30 (s, 1H); $^{13}$C NMR (100 MHz, $CDCl_3$): δ19.7, 66.4, 115.3, 117.8, 127.4, 128.3, 129.1, 129.4, 137.8, 144.4, 196.8;ESI-HRMS: Calcd. for $C_{15}H_{16}NO$ $[M+H]^+$: 226.1226, found 226.1230. IR (ATR), see attached spectrum.

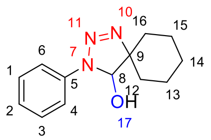

3-Phenyl-1,2,3-triazaspiro[4.5]dec-1-en-4-ol (5**a**). Triazoline **6a** decomposed during $SiO_2$-gel column chromatography separation and was characterized from the crude product. $^1$H NMR (400 MHz, DMSO): $\delta_H$ 1.19 – 1.88 (m, 10H), 5.26 (s, 1H, $C_{(8)}$H), 4.10 – 6.50 (br. s, 1H, OH), 6.97 (t, 1H, $C_{(2)}$H, J = 7.5 Hz), 7.29 (t, 2H, $C_{(1,3)}$H, J = 7.5 Hz), 7.43 (d, 2H, $C_{(4,6)}$H, J = 8 Hz); $^{13}$C NMR (100 MHz, DMSO): δ22.7, 23.4, 25.5, 27.8, 32.4, 82.7 ($C_{(9)}$), 83.1($C_{(8)}$), 115.5, 122.2, 129.5, 140.8.

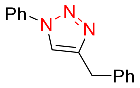

4-Benzyl-1-phenyl-1*H*-1,2,3-triazole(**8**).[48]White solid. $R_f$ = 0.25 (6:1 hexanes/EtOAc). $^1$H NMR (400 MHz, $CDCl_3$): δ 7.68 (m, 2H), 7.60 (s, 1H), 7.50 (m, 2H), 7.42 (m, 1H), 7.33 (m, 4H), 7.26 (m, 1H), 4.18 (s, 2H); $^{13}$C NMR (100 MHz, $CDCl_3$): δ32.5, 119.8, 120.6, 126.8, 128.7, 128.9, 129.0, 129.8, 137.3, 139.0, 148.7.

## References


(1) L'Abbe, G., *Chem. Rev.* **1969**, *69*, 345.

(2) Zanirato, P., *Arkivoc* **2009**, 97.

(3) L'Abbe, G.; Mathys, G., *J. Org. Chem.* **1974**, *39*, 1778.

(4) Xie, S.; Lopez, S. A.; Ramstrom, O.; Yan, M.; Houk, K. N., *J. Am. Chem. Soc.* **2015**, *137*, 2958.

(5) Keana, J. F. W.; Cai, S. X., *J. Org. Chem.* **1990**, *55*, 3640.

(6) Jin, L. M.; Xu, X.; Lu, H.; Cui, X.; Wojtas, L.; Zhang, X. P., *Angew. Chem. Int. Ed.* **2013**, *52*, 5309.

(7) Chapyshev, S. V., *Chem. Heterocycl. Compd.* **2001**, *37*.

(8) Berger, O.; Kaniti, A.; van Ba, C.; Vial, H.; Ward, S.; Biagini, G.; Bray, P.; O'Neill, P., *ChemMedChem* **2011**, *6*, 2094.

(9) Alonso, F.; Moglie, Y.; Radivoy, G.; Yus, M., *Eur. J. Org. Chem.* **2010**, *2010*, 1875.

(10) Serwinski, P.; Esat, B.; Lahti, P.; Liao, Y.; Walton, R.; Lan, J., *J. Org. Chem.* **2004**, *69*, 5247.

(11) Ueno, M.; Hori, C.; Suzawa, K.; Ebisawa, M.; Kondo, Y., *Eur. J. Org. Chem.* **2005**, *2005*, 1965.

(12) Kwok, S. W.; Fotsing, J. R.; Fraser, R. J.; Rodionov, V. O.; Fokin, V. V., *Org. Lett.* **2010**, *12*, 4217.

(13) Jin, L. M.; Xu, X.; Lu, H.; Cui, X.; Wojtas, L.; Zhang, X. P., *Angew. Chem. Int. Ed.* **2013**, *52*, 5309.

(14) Hu, M.; Li, J.; Yao, S. Q., *Org. Lett.* **2008**, *10*, 5529.

(15) Kitamura, M.; Kato, S.; Yano, M.; Tashiro, N.; Shiratake, Y.; Sando, M.; Okauchi, T., *Org. Biomol. Chem.* **2014**, *12*, 4397.

(16) Morawietz, J.; Sander, W.; Traeubel, M., *J. Org. Chem.* **1995**, *60*, 6368.

(17) Chitre, K. P.; Guillén, E.; Yoon, A. S.; Galoppini, E., *Eur. J. Inorg. Chem.* **2012**, *2012*, 5461.

(18) Nicolaides, A.; Enyo, T.; Miura, D.; Tomioka, H., *J. Am. Chem. Soc.* **2001**, *123*, 2628.

(19) Smith, C. D.; Greaney, M. F., *Org. Lett.* **2013**, *15*, 4826.

(20) Laitar, D. S.; Mathison, C. J.; Davis, W. M.; Sadighi, J. P., *Inorg. Chem.* **2003**, *42*, 7354.

(21) Tran, A. T.; Cergol, K. M.; Britton, W. J.; Imran Bokhari, S. A.; Ibrahim, M.; Lapthorn, A. J.; Payne, R. J., *MedChemComm* **2010**, *1*, 271.

(22) Potratz, S.; Mishra, A.; Bauerle, P., *Beilstein. J. Org. Chem.* **2012**, *8*, 683.

(23) Yarovenko, V. N.; Smirnova, N. G.; Bulgakova, V. N.; Zavarzin, I. V.; Krayushkin, M. M., *RUSS. J. ORG. CHEM.* **2003**, *39*, 1161.

(24) Liu, H.; Yan, N.; Dyson, P. J., *Chem. Commun.* **2014**, *50*, 7848.

(25) (a) Herbst, R. M.; Roberts, C. W.; Givens, H. T. F.; Harvill, E. K., *J. Org. Chem.* **1952,***17*, 262. (b) Suzuki, Y. U., Jpn. Kokai Tokkyo Koho (**2011**), JP 2011128469 A 20110630.

(26) Park, Y.-T.; Kim, M.-S.; Kwak, Y.-W.; Lee, J.-K.; Yoh, S.-D.; Kim, W.-S., *J. Photosci.* **2000,***7*, 135.

(27) La Regina, G.; Bai, R.; Rensen, W. M.; Di Cesare, E.; Coluccia, A.; Piscitelli, F.; Famiglini, V.; Reggio, A.; Nalli, M.; Pelliccia, S.; Da Pozzo, E.; Costa, B.; Granata, I.; Porta, A.; Maresca, B.; Soriani, A.; Iannitto, M. L.; Santoni, A.; Li, J.; Miranda Cona, M.; Chen, F.; Ni, Y.; Brancale, A.; Dondio, G.; Vultaggio, S.; Varasi, M.; Mercurio, C.; Martini, C.; Hamel, E.; Lavia, P.; Novellino, E.; Silvestri, R., *J. med. chem.***2013,***56*, 123.

(28) Beyer, A.; Buendia, J.; Bolm, C., *Org. Lett.* **2012,***14*, 3948.

(29) Nakatsuji, H.; Morimoto, M.; Misaki, T.; Tanabe, Y., *Tetrahedron* **2007,***63*, 12071.

(30) Carbone, G.; Burnley, J.; Moses, J. E., *Chem. Commun.* **2013,***49*, 2759.

(31) (a) Hara, T.; Wada, N.; Iwamura, H., *J. Agric. Food Chem.* **1992,***40*, 1692. (b) Li, W.; Wu, X. F., *J. Org. Chem.* **2014,***79*, 10410.

(32) Coppo, F. T.; Maskell, E. S. L.; Redshaw, S., WO 2009071519 A1,**2009**.

(33) (a) Mohmeyer, N.; Schmidt, H. W., *Chemistry* **2007,***13*, 4499. (b) Flaherty, D. P.; Simpson, D. S.; Miller, M.; Maki, B. E.; Zou, B. Y.; Shi, J.; Wu, M.; McManus, O. B.; Aube, J.; Li, M.; Golden, J. E., *Bioorg. Med. Chem. Lett.***2014,***24*, 3968.

(34) (a) Takahashi, N. A., Osamu, Eur. Pat. Appl. (**1998**), EP 878741 A2 19981118. (b) Ono, H. T., Noriaki; Ando, Osamu; Takeuchi, Masako, Jpn. Kokai Tokkyo Koho (1997), JP 09160301 A 19970620.

(35) Fors, B. P.; Krattiger, P.; Strieter, E.; Buchwald, S. L., *Org. Lett.* **2008,***10*, 3505.

(36) Fors, B. P.; Dooleweerdt, K.; Zeng, Q.; Buchwald, S. L., *Tetrahedron* **2009,***65*, 6576.

(37) Morimoto, H.; Fujiwara, R.; Shimizu, Y.; Morisaki, K.; Ohshima, T., *Org. Lett.* **2014,***16*, 2018.

(38) Xiang, S. K.; Zhang, D. X.; Hu, H.; Shi, J. L.; Liao, L. G.; Feng, C.; Wang, B. Q.; Zhao, K. Q.; Hu, P.; Yang, H.; Yu, W. H., *Adv. Synth. Catal.***2013,***355*, 1495.

(39) (a) Baigrie, L. M.; Lenoir, D.; Seikaly, H. R.; Tidwell, T. T., *J. Org. Chem.* **1985,***50*, 2105. (B) Cooke, M. P.; Pollock, C. M., *J. Org. Chem.* **1993,***58*, 7474.

(40) Ye, W.; Mo, J.; Zhao, T.; Xu, B., *Chem. Commun.* **2009**, 3246.

(41) Dunetz, J. R.; Xiang, Y.; Baldwin, A.; Ringling, J., *Org. Lett.* **2011,***13*, 5048.

(42) Jia, Y. X.; Katayev, D.; Bernardinelli, G.; Seidel, T. M.; Kundig, E. P., *Chem. Eur. J.* **2010,***16*, 6300.

(43) Bures, J.; Martin, M.; Urpi, F.; Vilarrasa, J., *J. Org. Chem.* **2009,***74*, 2203.

(44) Muñoz, J. d. M.; Alcázar, J.; de la Hoz, A.; Díaz-Ortiz, Á.; Alonso de Diego, S.-A., *Green Chem.* **2012,***14*, 1335.

(45) Upadhayaya, R. S.; Kulkarni, G. M.; Vasireddy, N. R.; Vandavasi, J. K.; Dixit, S. S.; Sharma, V.; Chattopadhyaya, J., *Bioorg. Med. Chem.* **2009,***17*, 4681.

(46) Li, J.; Subramaniam, K.; Smith, D.; Qiao, J. X.; Li, J. J.; Qian-Cutrone, J.; Kadow, J. F.; Vite, G. D.; Chen, B. C., *Org. Lett.* **2012,***14*, 214.

(47) Chiang, Y.; Grant, A. S.; Guo, H. X.; Kresge, A. J.; Paine, S. W., *J. Org. Chem.* **1997,***62*, 5363.

(48) Macdonald, J. E.; Kelly, J. A.; Veinot, J. G., *Langmuir* **2007,***23*, 9543.

## Characterization Spectra

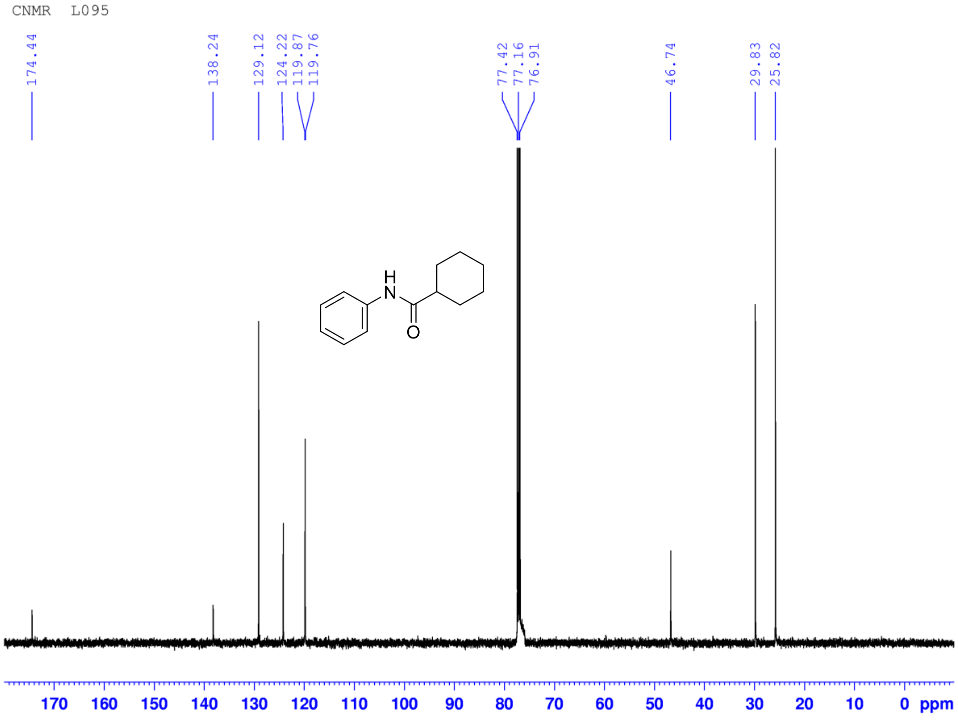


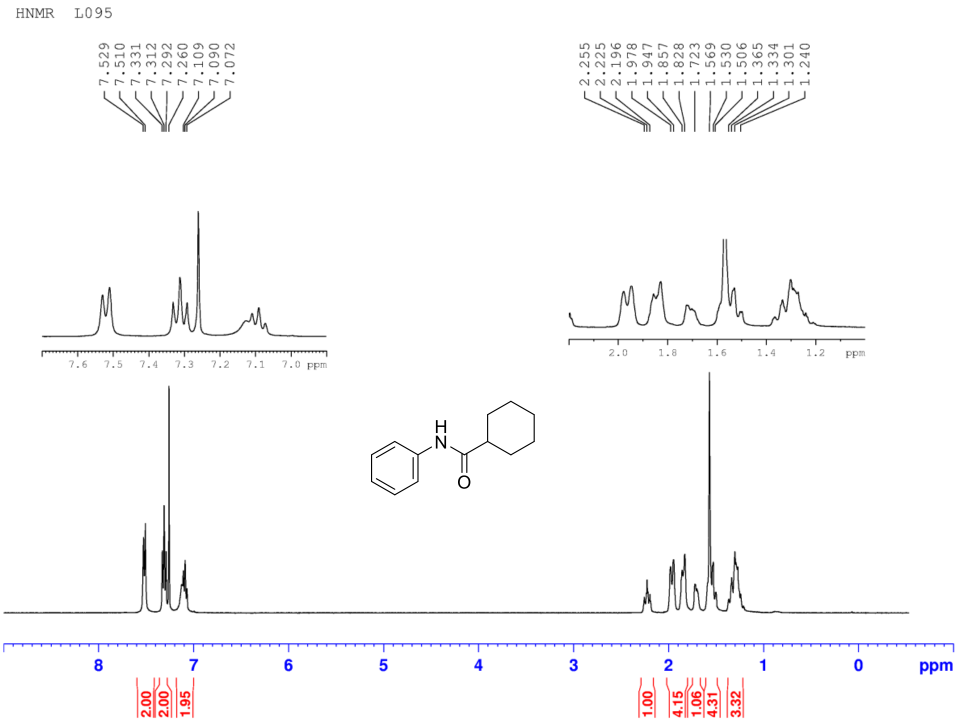

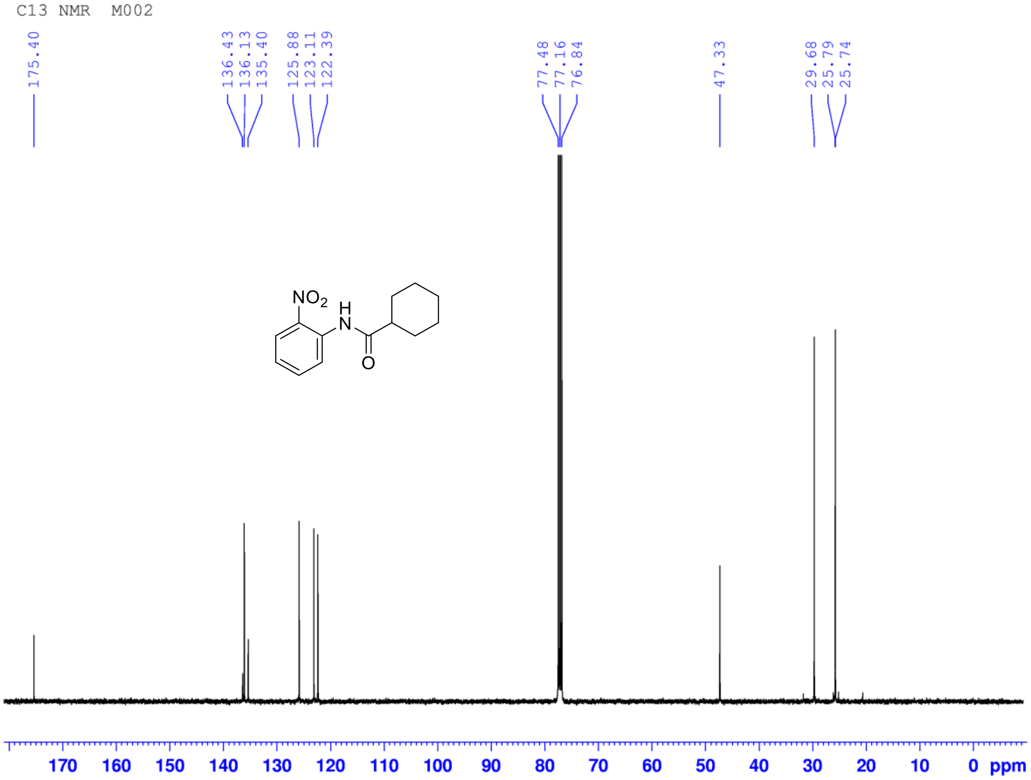
C13 NMR M002
175.40
136.43
136.13
135.40
125.88
123.11
122.39
77.48
77.16
76.84
47.33
29.68
25.79
25.74
NO2
H
N
O
170 160 150 140 130 120 110 100 90 80 70 60 50 40 30 20 10 0 ppm

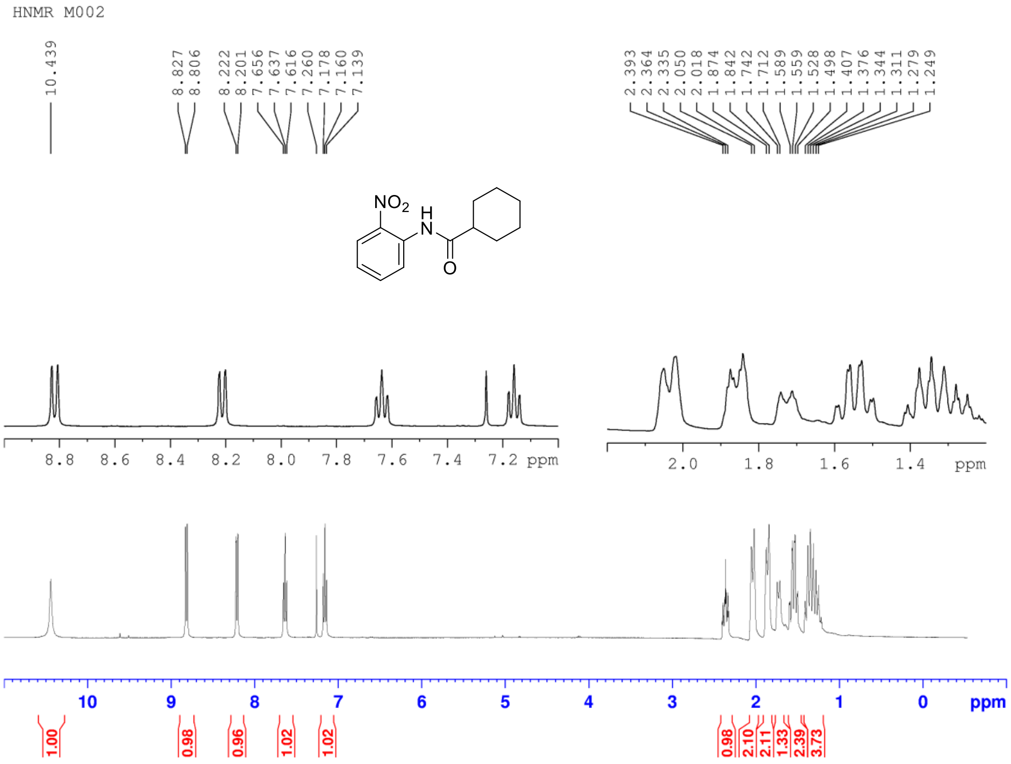
HNMR M002
10.439
8.827
8.806
8.222
8.201
7.656
7.637
7.616
7.260
7.178
7.160
7.139
2.393
2.364
2.335
2.050
2.018
1.874
1.842
1.742
1.712
1.589
1.559
1.528
1.498
1.407
1.376
1.344
1.311
1.279
1.249
NO2
H
N
O
8.8 8.6 8.4 8.2 8.0 7.8 7.6 7.4 7.2 ppm
2.0 1.8 1.6 1.4 ppm
10 9 8 7 6 5 4 3 2 1 0 ppm
1.00
0.98
0.96
1.02
1.02
0.98
2.10
2.11
1.33
2.39
3.73

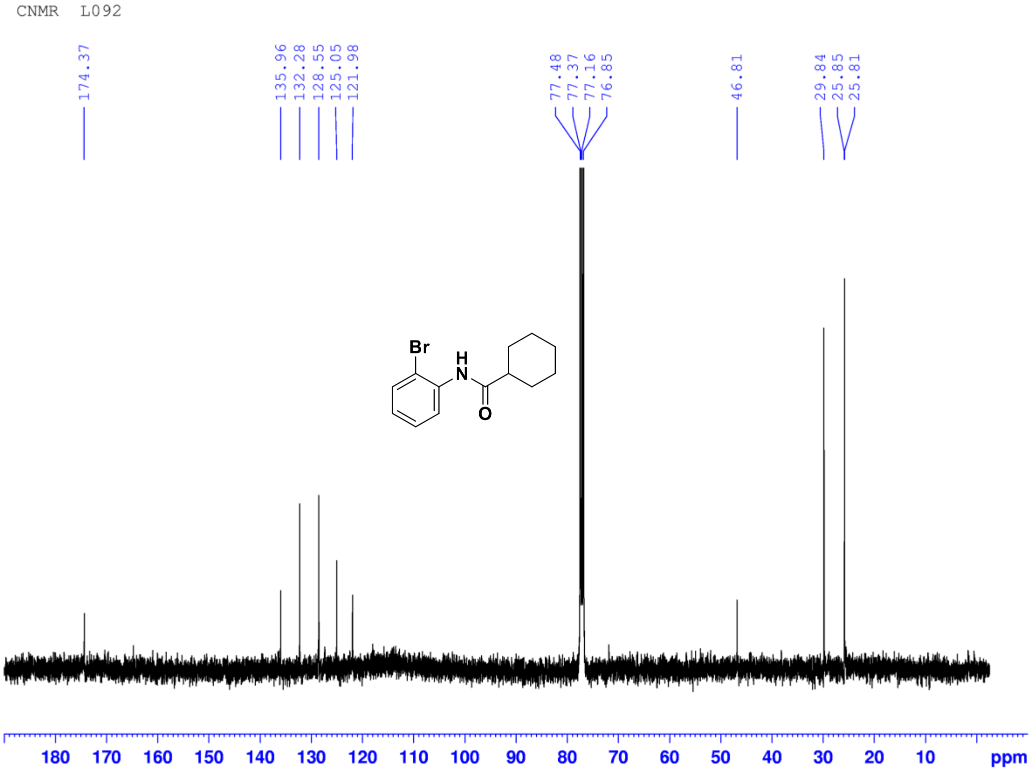
CNMR L092
174.37
135.96
132.28
128.55
125.05
121.98
77.48
77.37
77.16
76.85
46.81
29.84
25.85
25.81
Br
H
N
O
180 170 160 150 140 130 120 110 100 90 80 70 60 50 40 30 20 10 ppm

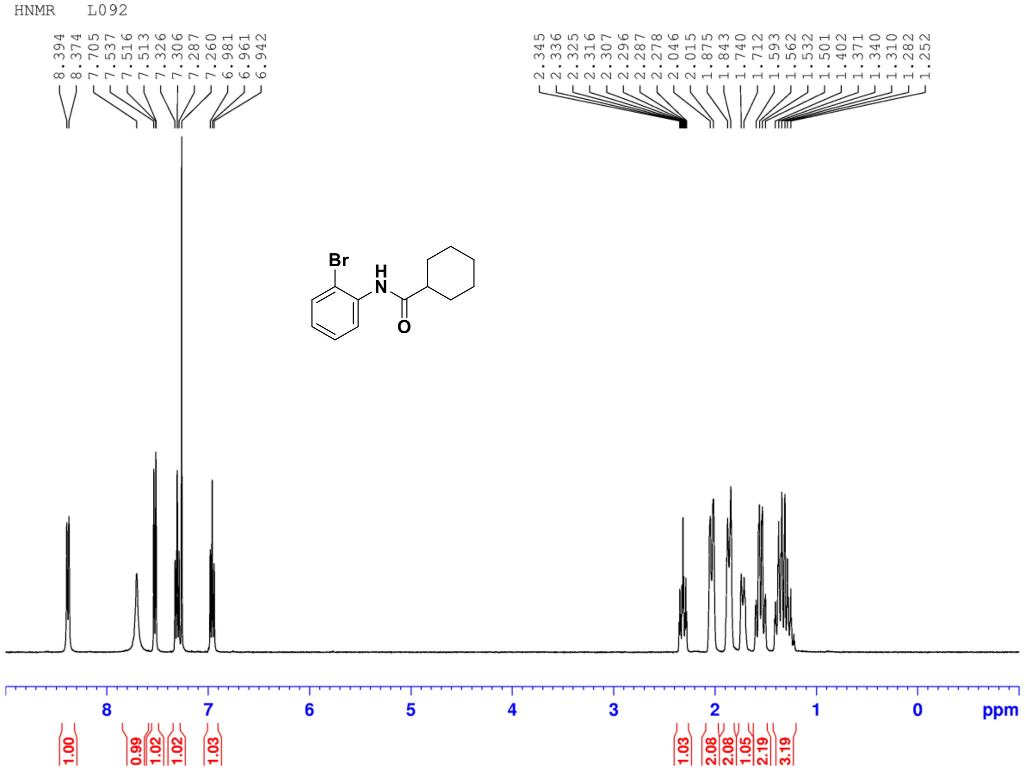
HNMR L092
8.394
8.374
7.705
7.537
7.516
7.513
7.326
7.306
7.287
7.260
6.981
6.961
6.942
2.345
2.336
2.325
2.316
2.307
2.296
2.287
2.278
2.046
2.015
1.875
1.843
1.740
1.712
1.593
1.562
1.532
1.501
1.402
1.371
1.340
1.310
1.282
1.252
Br
H
N
O
8 7 6 5 4 3 2 1 0 ppm
1.00
0.99
1.02
1.02
1.03
1.03
2.08
2.08
1.05
2.19
3.19

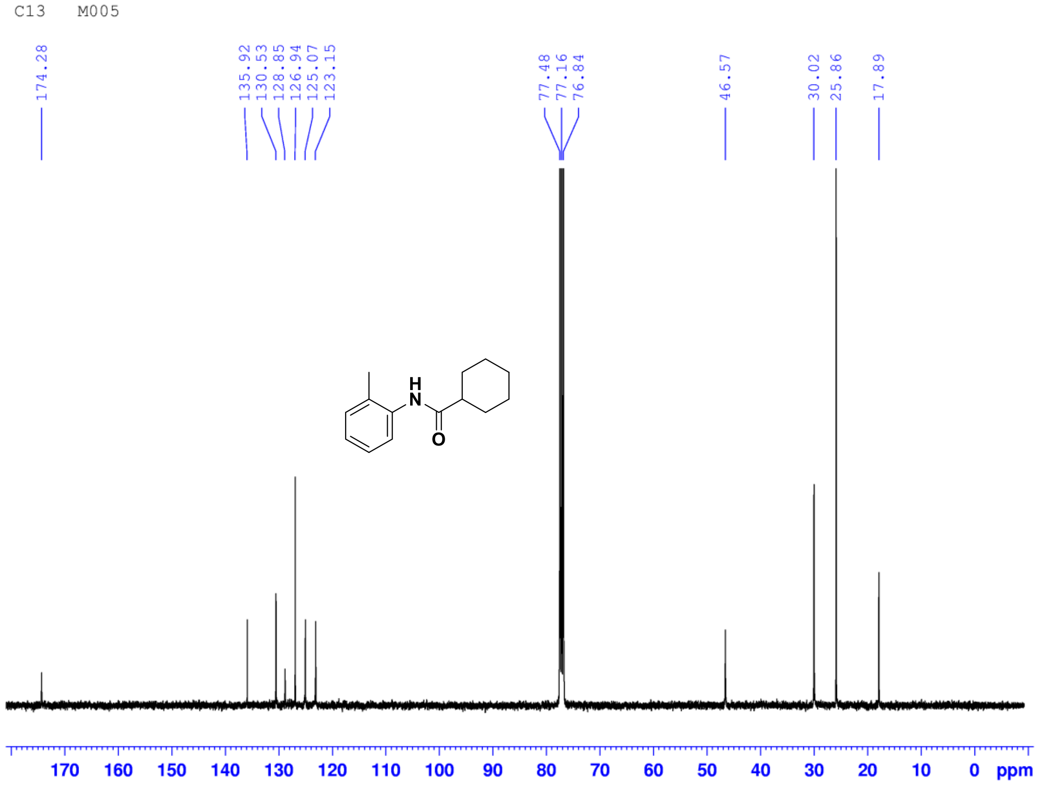
C13 M005
174.28
135.92
130.53
128.85
126.94
125.07
123.15
77.48
77.16
76.84
46.57
30.02
25.86
17.89
H
N
O
170 160 150 140 130 120 110 100 90 80 70 60 50 40 30 20 10 0 ppm

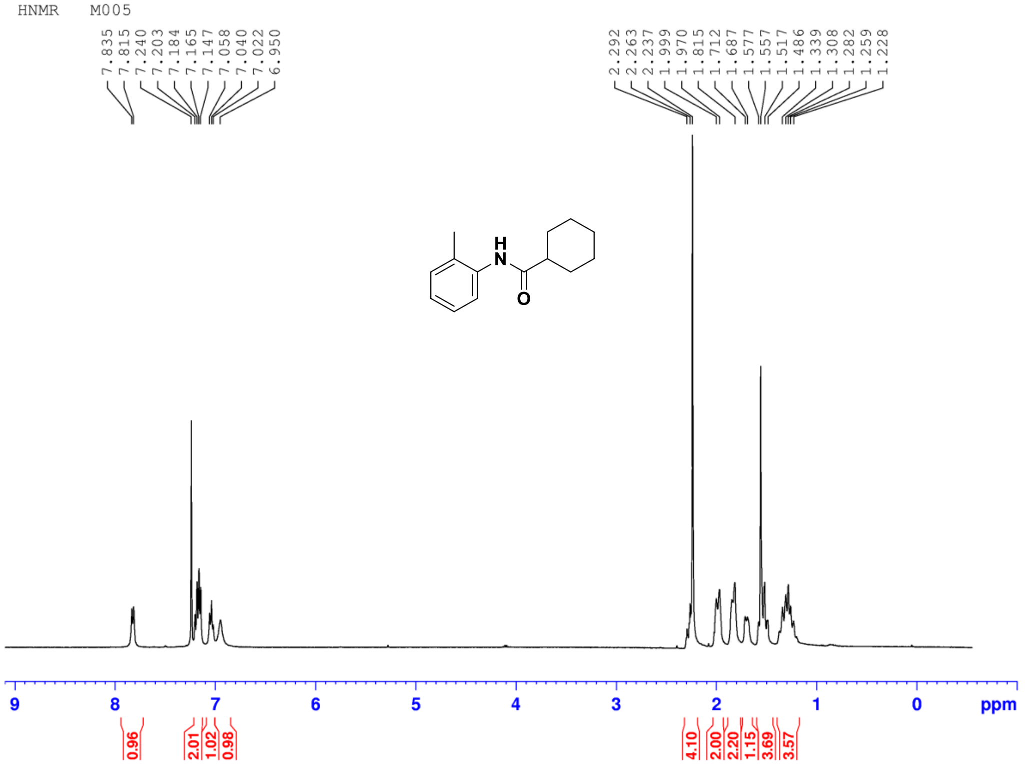
HNMR M005
7.835
7.815
7.240
7.203
7.184
7.165
7.147
7.058
7.040
7.022
6.950
2.292
2.263
2.237
1.999
1.970
1.815
1.712
1.687
1.577
1.557
1.517
1.486
1.339
1.308
1.282
1.259
1.228
H
N
O
9 8 7 6 5 4 3 2 1 0 ppm
0.96
2.01
1.02
0.98
4.10
2.00
2.20
1.15
3.69
3.57

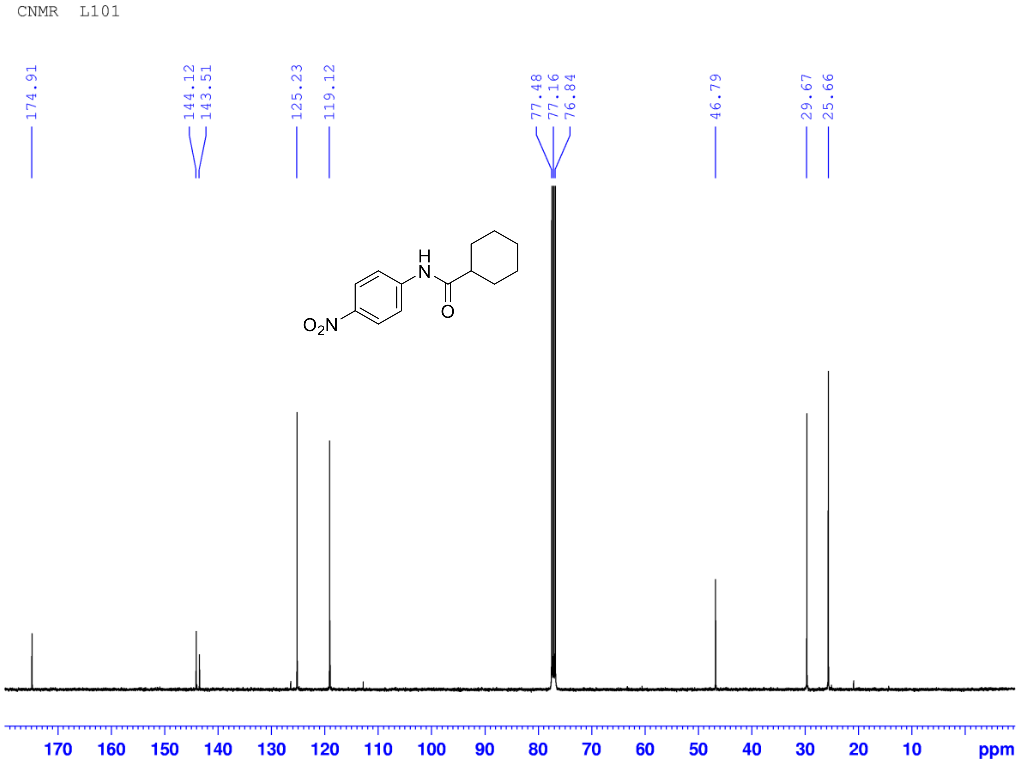
CNMR L101
174.91
144.12
143.51
125.23
119.12
77.48
77.16
76.84
46.79
29.67
25.66
$O_2N$
H
N
O
170 160 150 140 130 120 110 100 90 80 70 60 50 40 30 20 10 ppm

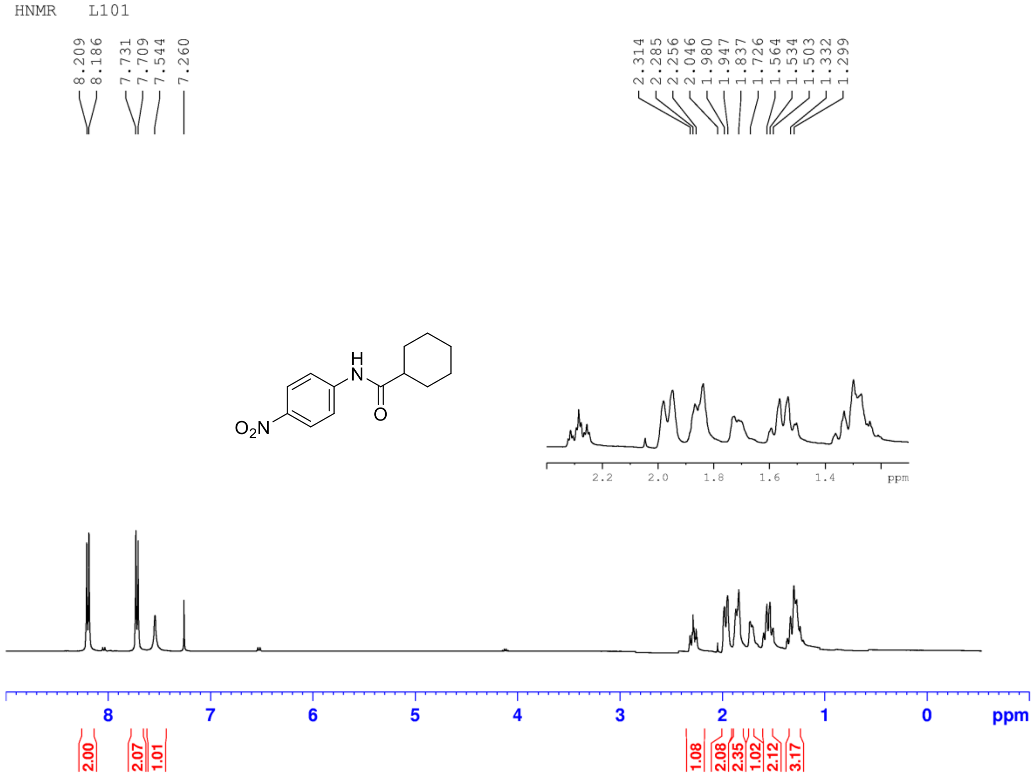
HNMR L101
8.209
8.186
7.731
7.709
7.544
7.260
2.314
2.285
2.256
2.046
1.980
1.947
1.837
1.726
1.564
1.534
1.503
1.332
1.299
$O_2N$
H
N
O
2.2 2.0 1.8 1.6 1.4 ppm
8 7 6 5 4 3 2 1 0 ppm
2.00
2.07
1.01
1.08
2.08
2.35
1.02
2.12
3.17

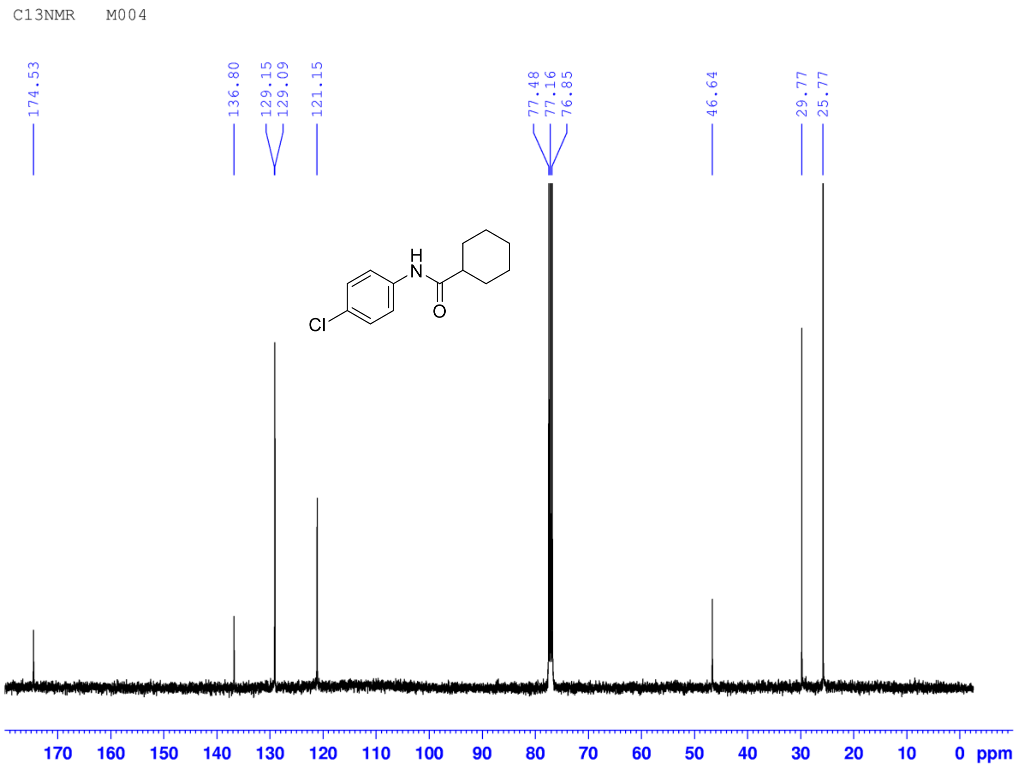
C13NMR M004
174.53
136.80
129.15
129.09
121.15
77.48
77.16
76.85
46.64
29.77
25.77
H
N
O
Cl
170 160 150 140 130 120 110 100 90 80 70 60 50 40 30 20 10 0 ppm

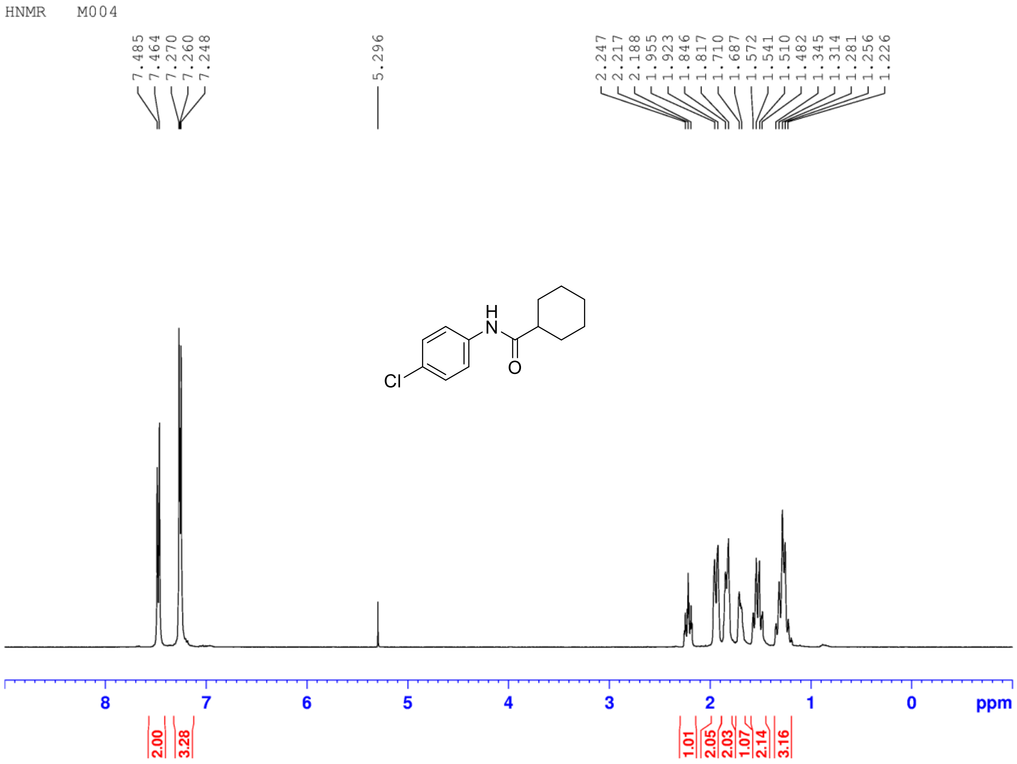
HNMR M004
7.485
7.464
7.270
7.260
7.248
5.296
2.247
2.217
2.188
1.955
1.923
1.846
1.817
1.710
1.687
1.572
1.541
1.510
1.482
1.345
1.314
1.281
1.256
1.226
H
N
O
Cl
8 7 6 5 4 3 2 1 0 ppm
2.00
3.28
1.01
2.05
2.03
1.07
2.14
3.16

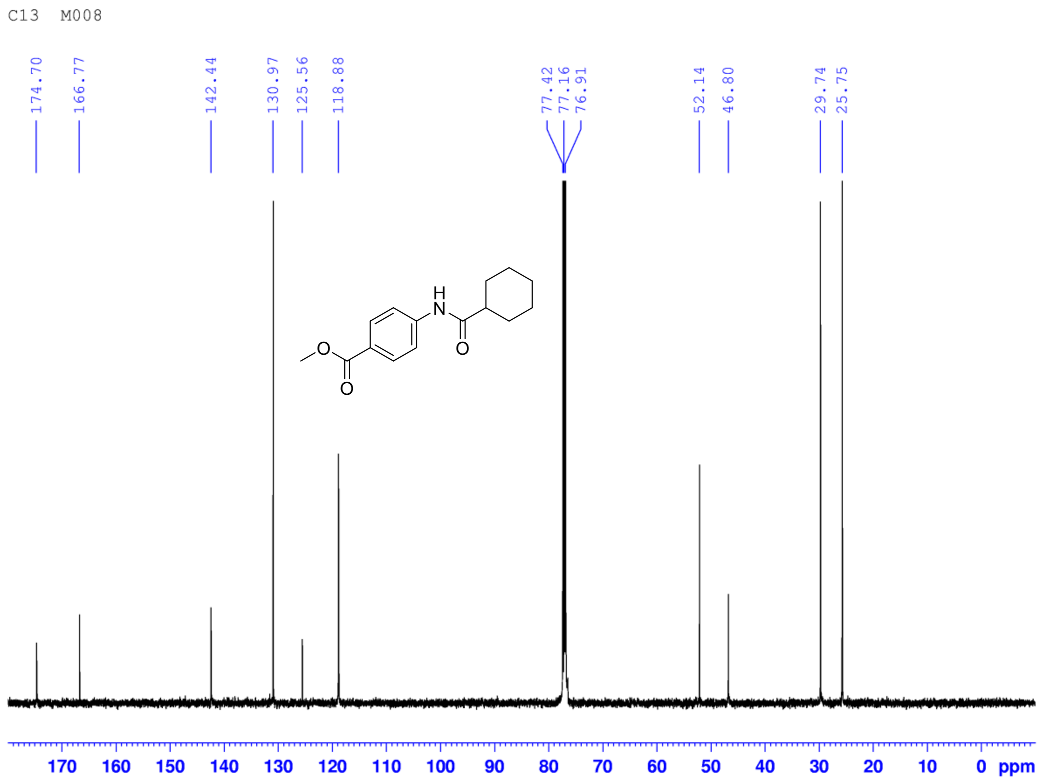
C13 M008
174.70
166.77
142.44
130.97
125.56
118.88
77.42
77.16
76.91
52.14
46.80
29.74
25.75
H
N
O
O
O
170 160 150 140 130 120 110 100 90 80 70 60 50 40 30 20 10 0 ppm

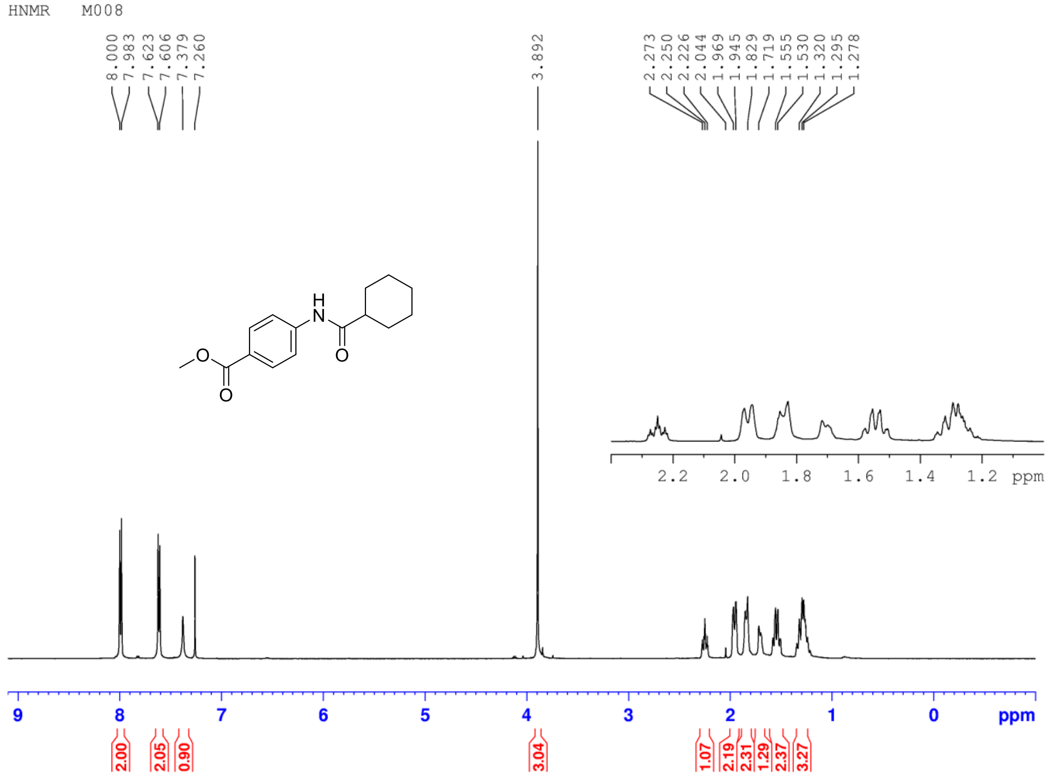
HNMR M008
8.000
7.983
7.623
7.606
7.379
7.260
3.892
2.273
2.250
2.226
2.044
1.969
1.945
1.829
1.719
1.555
1.530
1.320
1.295
1.278
H
N
O
O
O
2.2 2.0 1.8 1.6 1.4 1.2 ppm
9 8 7 6 5 4 3 2 1 0 ppm
2.00
2.05
0.90
3.04
1.07
2.19
2.31
1.29
2.37
3.27

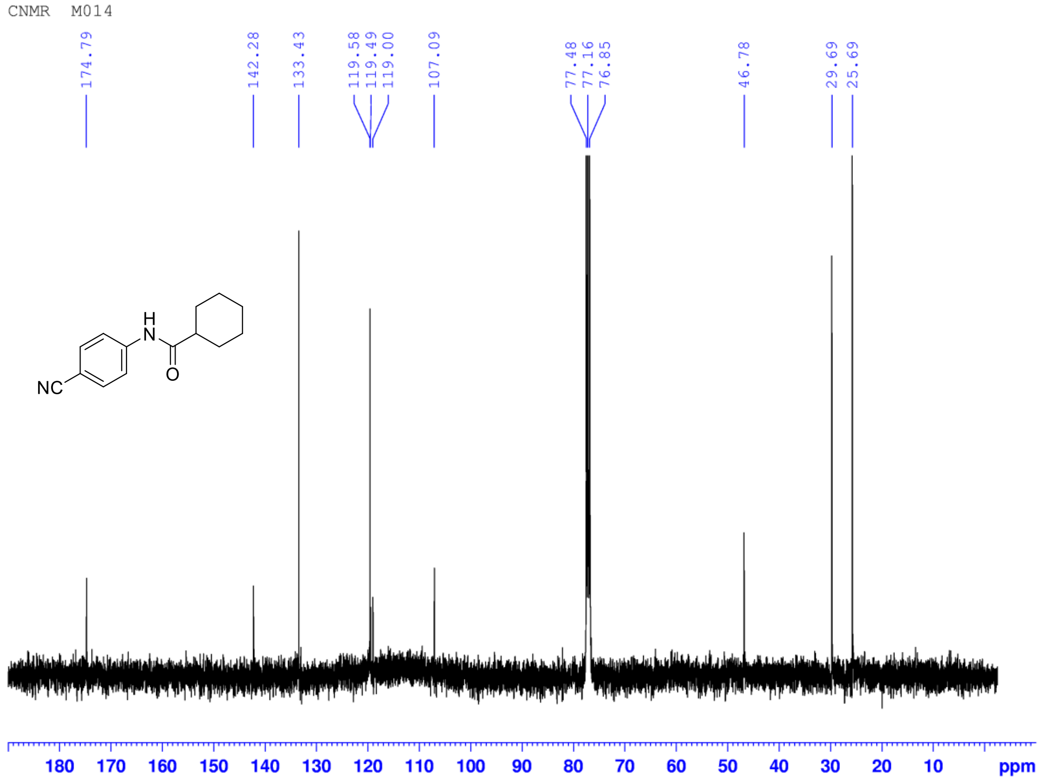
CNMR M014
174.79
142.28
133.43
119.58
119.49
119.00
107.09
77.48
77.16
76.85
46.78
29.69
25.69
NC
H
N
O
180 170 160 150 140 130 120 110 100 90 80 70 60 50 40 30 20 10 ppm

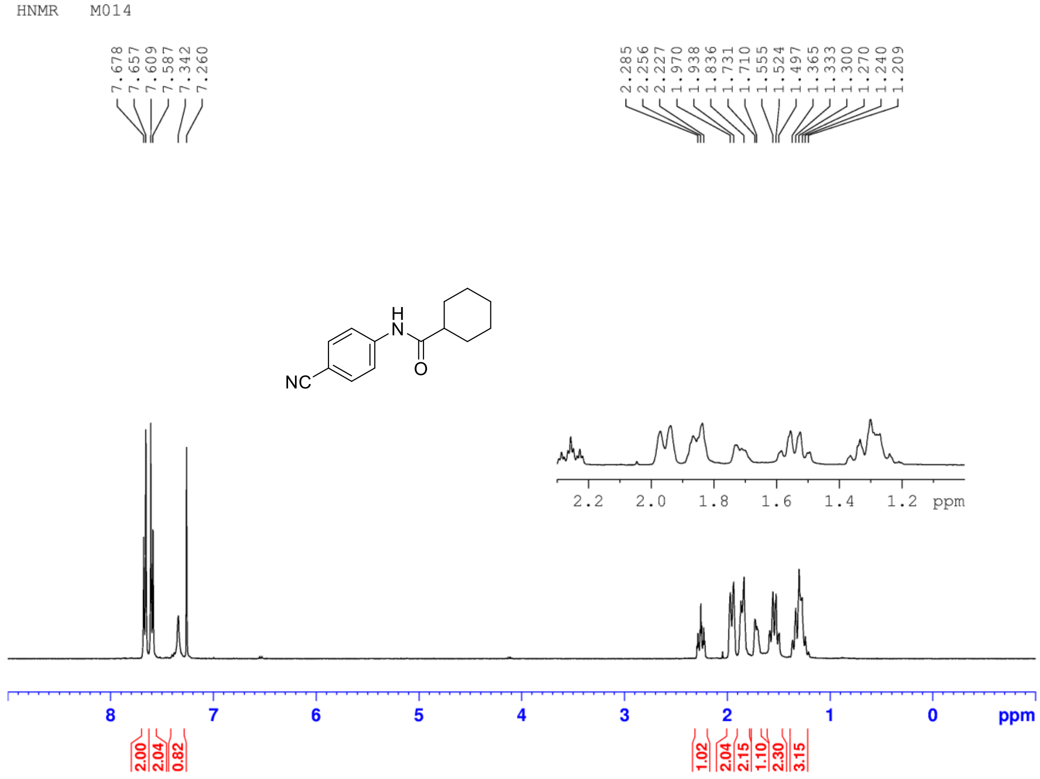
HNMR M014
7.678
7.657
7.609
7.587
7.342
7.260
2.285
2.256
2.227
1.970
1.938
1.836
1.710
1.731
1.555
1.524
1.497
1.365
1.333
1.300
1.270
1.240
1.209
NC
H
N
O
2.2 2.0 1.8 1.6 1.4 1.2 ppm
8 7 6 5 4 3 2 1 0 ppm
2.00
2.04
0.82
1.02
2.04
2.15
1.10
2.30
3.15

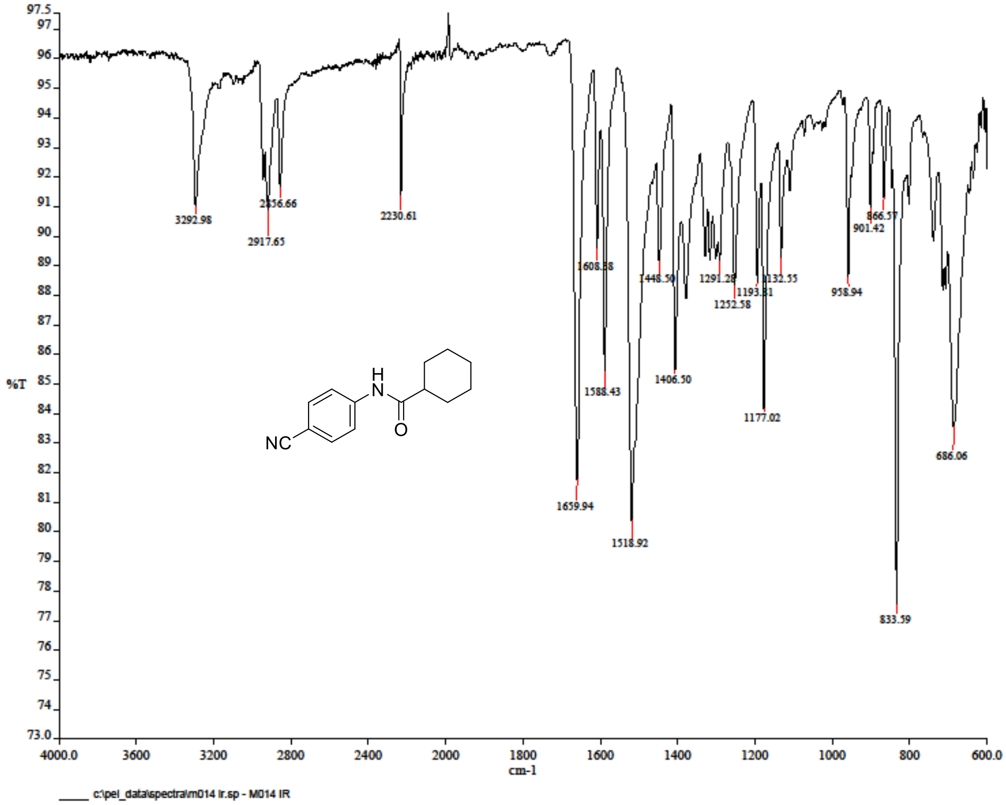
%T
cm-1
3292.98
2917.65
2856.66
2230.61
1659.94
1608.88
1588.43
1518.92
1448.50
1406.50
1291.28
1252.58
1193.81
1177.02
1132.55
958.94
901.42
866.57
833.59
686.06
NC
H
N
O
c:\pel_data\spectra\m014 ir.sp - M014 IR

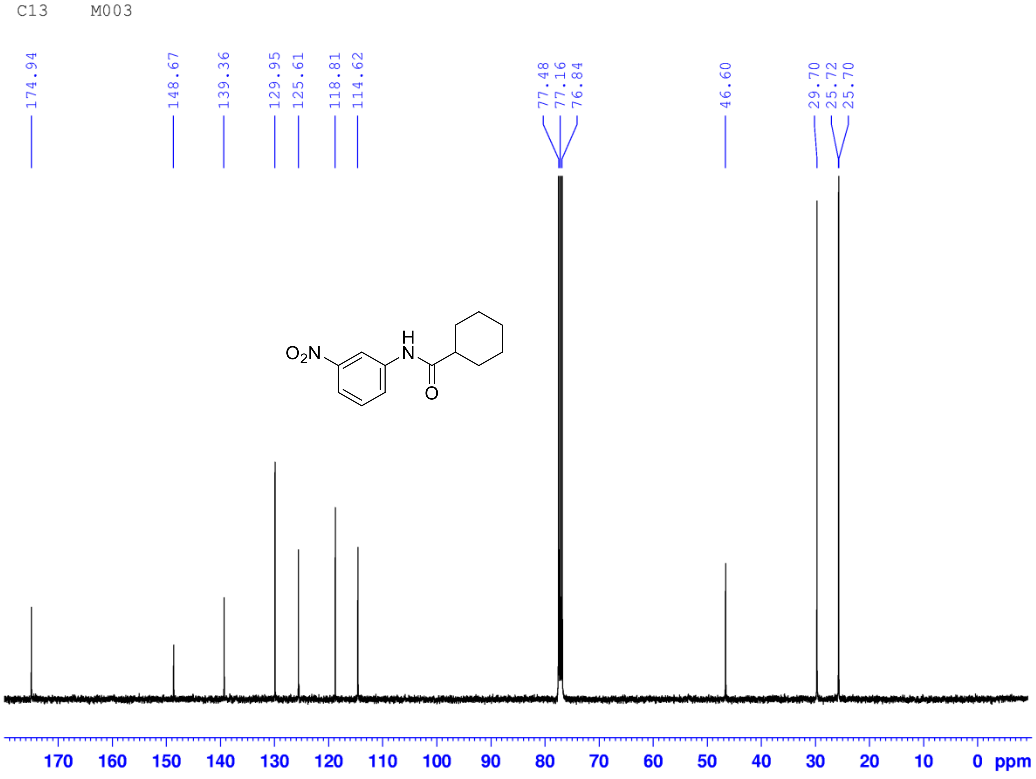
C13 M003
174.94
148.67
139.36
129.95
125.61
118.81
114.62
77.48
77.16
76.84
46.60
29.70
25.72
25.70
$O_2N$
H
N
O
170 160 150 140 130 120 110 100 90 80 70 60 50 40 30 20 10 0 ppm

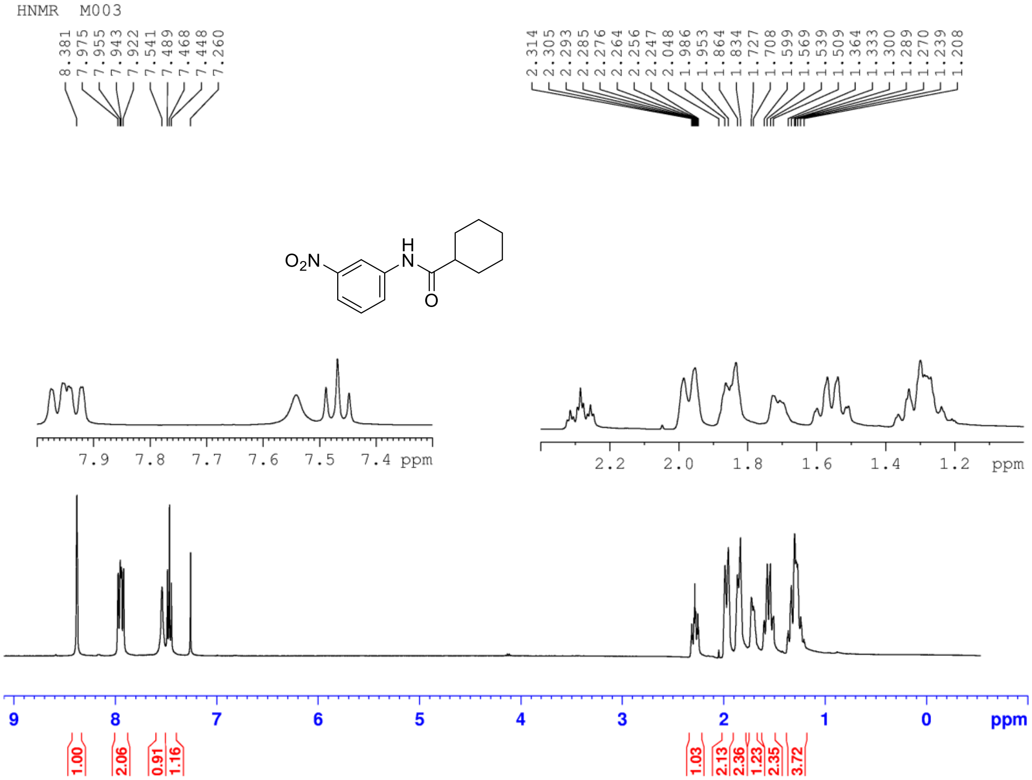
HNMR M003
8.381
7.975
7.955
7.943
7.922
7.541
7.489
7.468
7.448
7.260
2.314
2.305
2.293
2.285
2.276
2.264
2.256
2.247
2.048
1.986
1.953
1.864
1.834
1.727
1.708
1.599
1.569
1.539
1.509
1.364
1.333
1.300
1.289
1.270
1.239
1.208
$O_2N$
H
N
O
7.9 7.8 7.7 7.6 7.5 7.4 ppm
2.2 2.0 1.8 1.6 1.4 1.2 ppm
9 8 7 6 5 4 3 2 1 0 ppm
1.00
2.06
0.91
1.16
1.03
2.13
2.36
1.23
2.35
3.72

FNMR M013 CDCl3

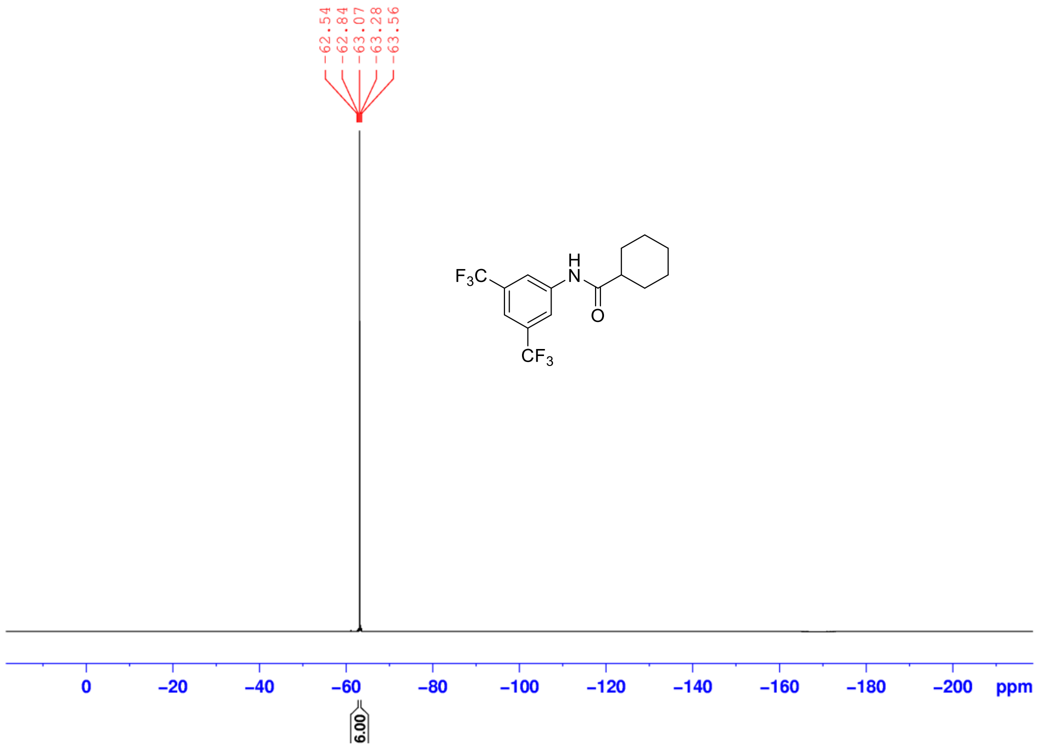


CNMR M013

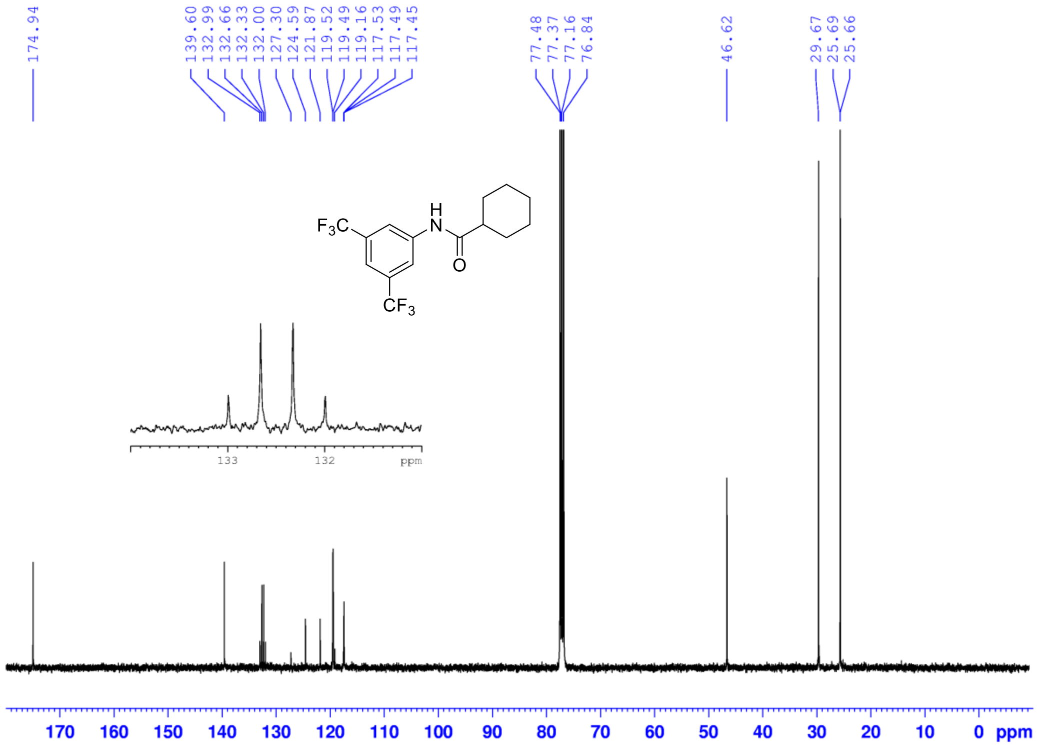

HNMR M013

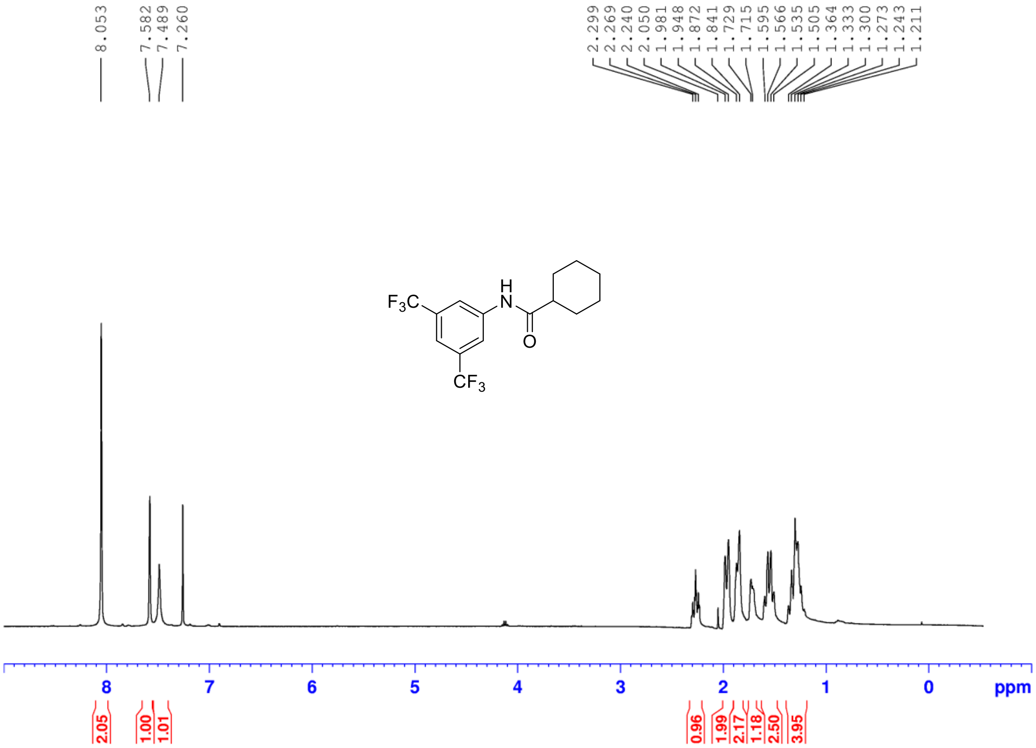


F19, CDCl3

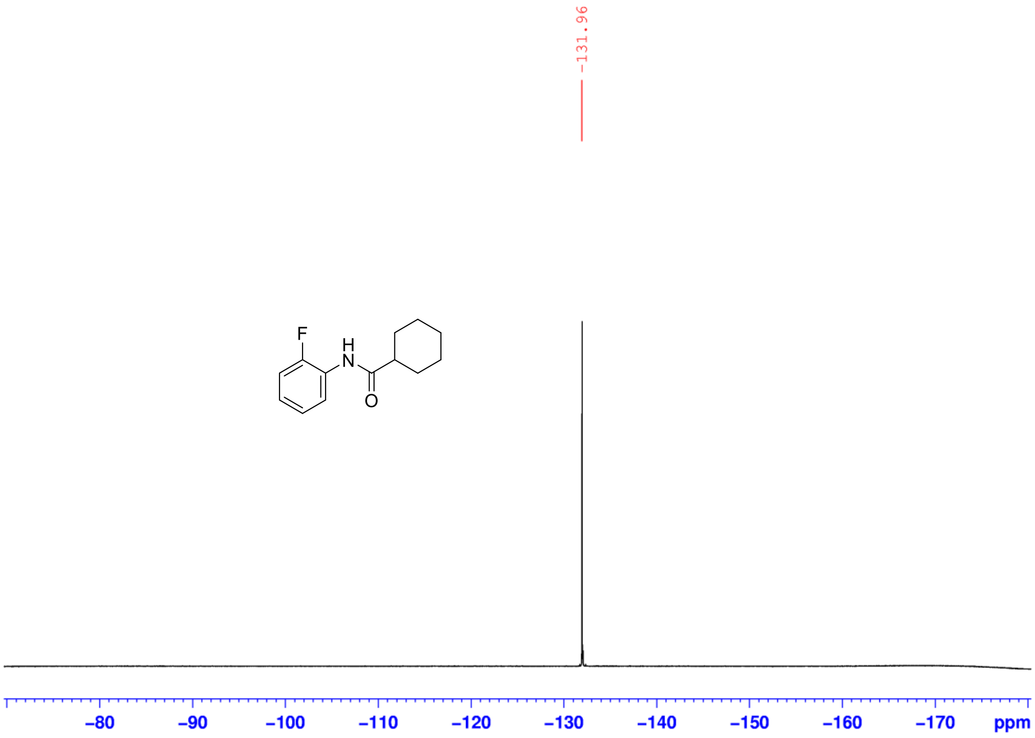

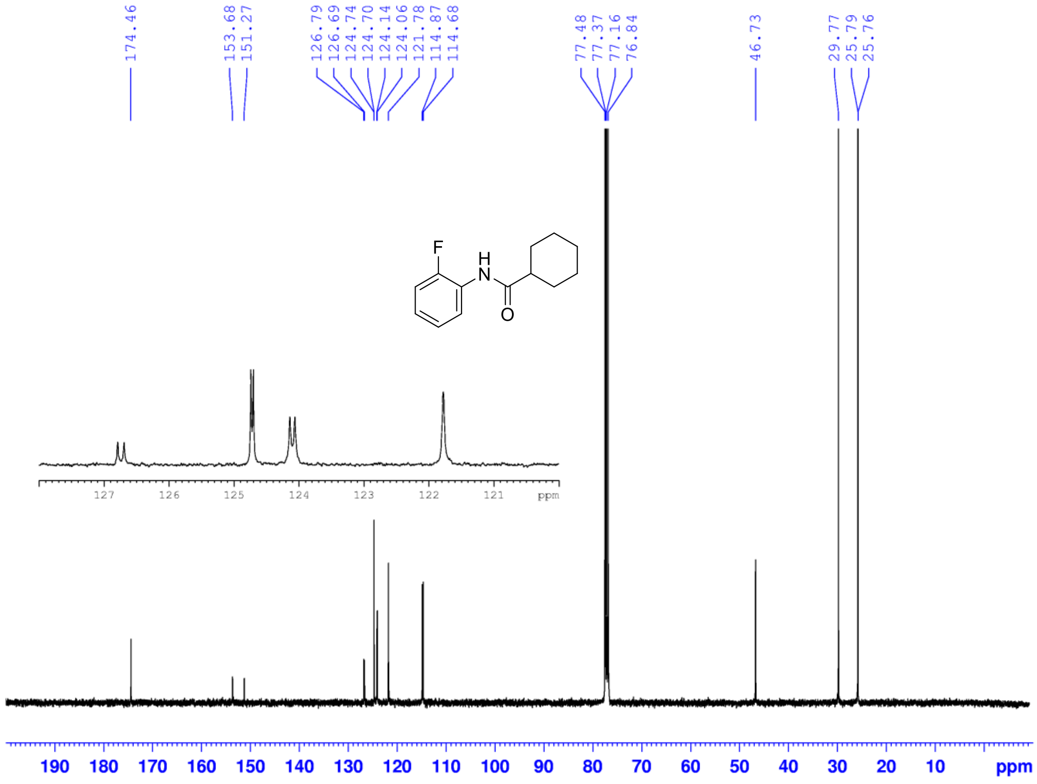
174.46
153.68
151.27
126.79
126.69
124.74
124.70
124.14
124.06
121.78
114.87
114.68
77.48
77.37
77.16
76.84
46.73
29.77
25.79
25.76
F
H
N
O
127 126 125 124 123 122 121 ppm
190 180 170 160 150 140 130 120 110 100 90 80 70 60 50 40 30 20 10 ppm

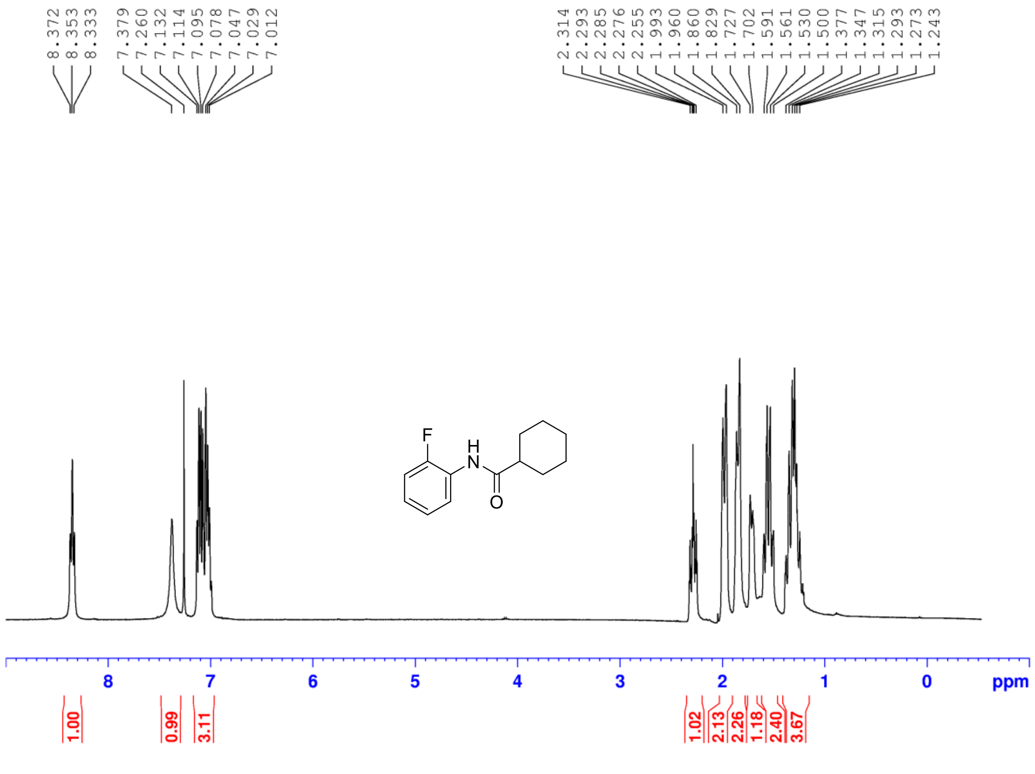
8.372
8.353
8.333
7.379
7.260
7.132
7.114
7.095
7.078
7.047
7.029
7.012
2.314
2.293
2.285
2.276
2.255
1.993
1.960
1.860
1.829
1.727
1.702
1.591
1.561
1.530
1.500
1.377
1.347
1.315
1.293
1.273
1.243
F
H
N
O
8 7 6 5 4 3 2 1 0 ppm
1.00
0.99
3.11
1.02
2.13
2.26
1.18
2.40
3.67

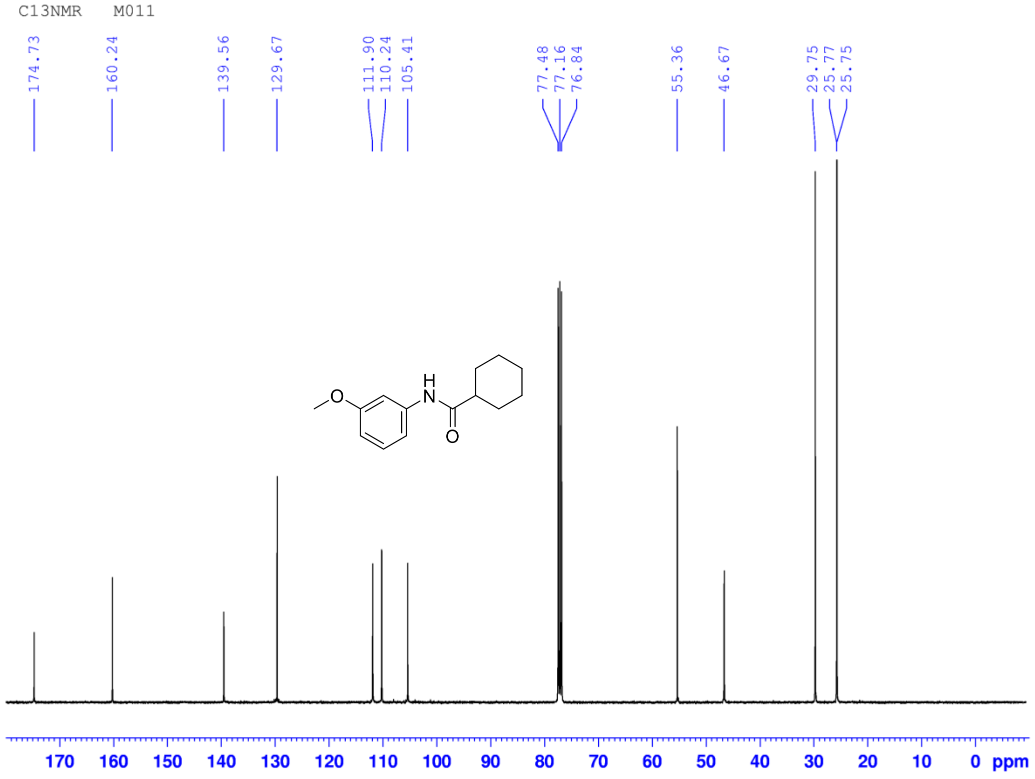
C13NMR M011
174.73
160.24
139.56
129.67
111.90
110.24
105.41
77.48
77.16
76.84
55.36
46.67
29.75
25.77
25.75
170 160 150 140 130 120 110 100 90 80 70 60 50 40 30 20 10 0 ppm

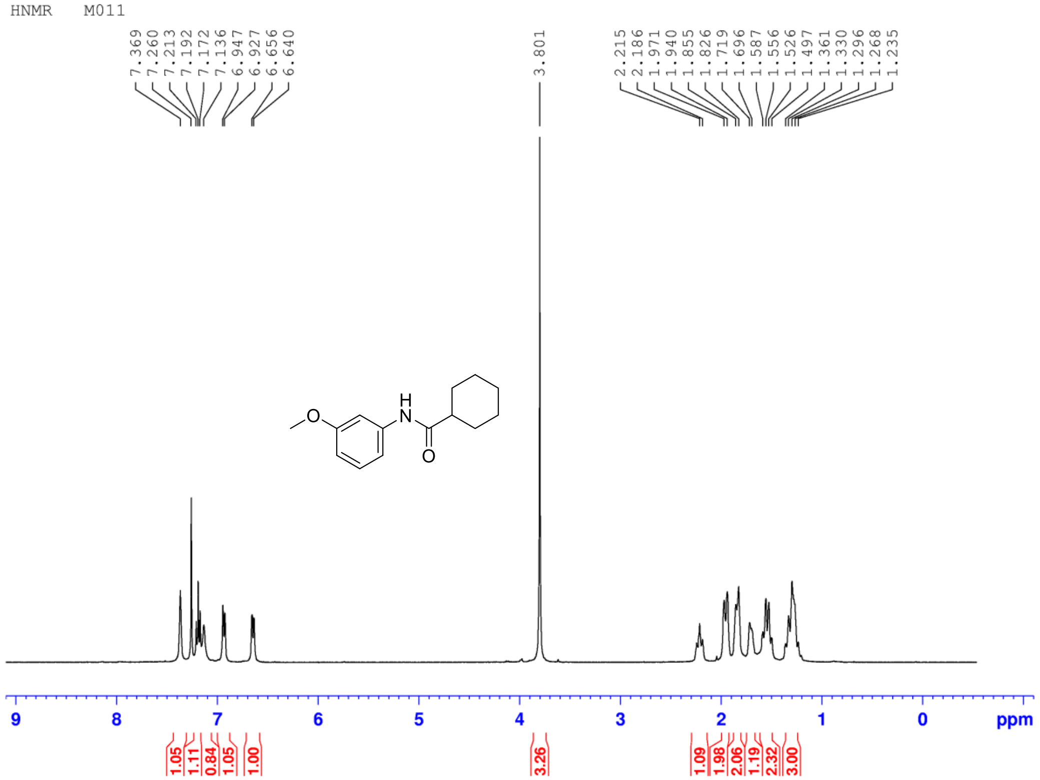
HNMR M011
7.369
7.260
7.213
7.192
7.172
7.136
6.947
6.927
6.656
6.640
3.801
2.215
2.186
1.971
1.940
1.855
1.826
1.719
1.696
1.587
1.556
1.526
1.497
1.361
1.330
1.296
1.268
1.235
9 8 7 6 5 4 3 2 1 0 ppm
1.05
1.11
0.84
1.05
1.00
3.26
1.09
1.98
2.06
1.19
2.32
3.00

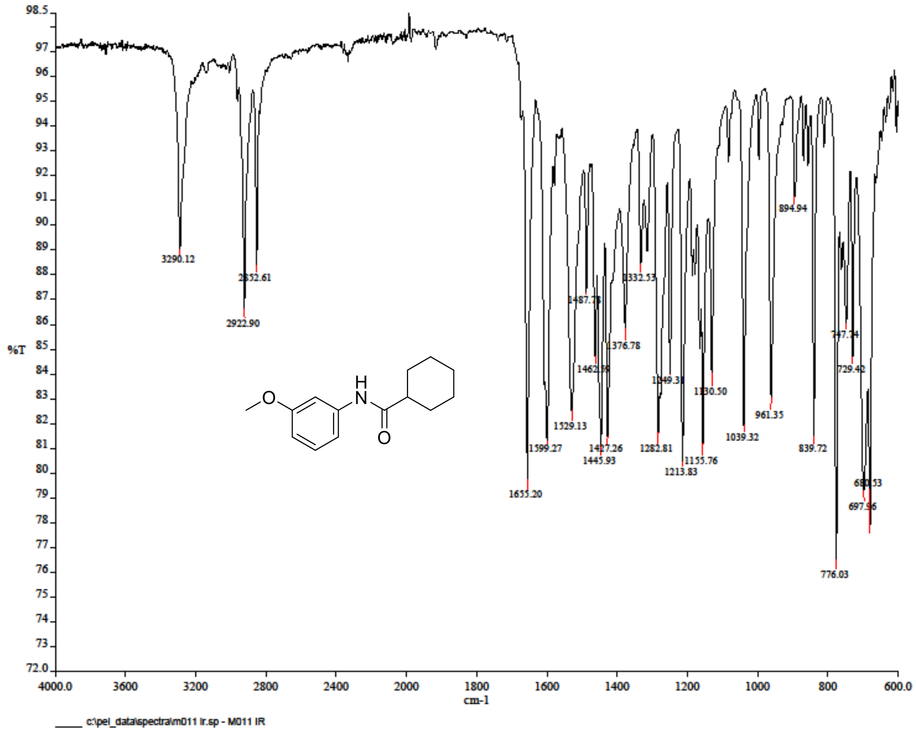
%T
cm-1
3290.12
2852.61
2922.90
1655.20
1599.27
1529.13
1445.93
1427.26
1376.78
1332.53
1282.81
1213.83
1155.76
1130.50
1039.32
961.35
894.94
839.72
776.03
747.74
729.42
697.96
c:\pel_data\spectra\m011 ir.sp - M011 IR

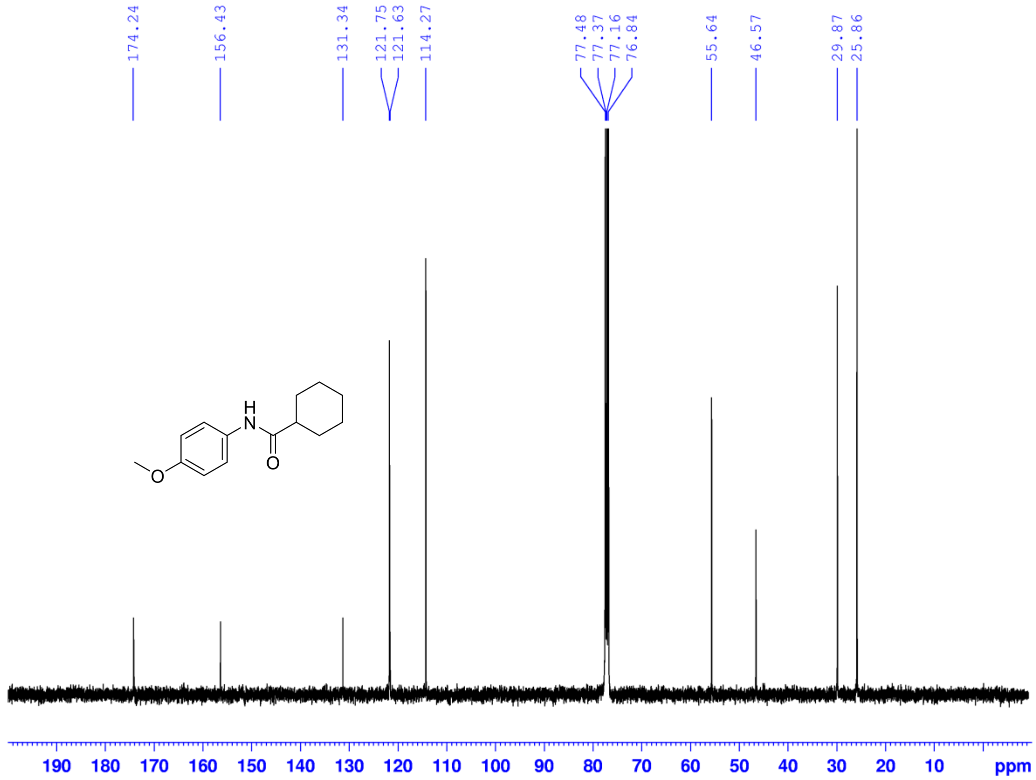

174.24
156.43
131.34
121.75
121.63
114.27
77.48
77.37
77.16
76.84
55.64
46.57
29.87
25.86
H
N
O
O
190 180 170 160 150 140 130 120 110 100 90 80 70 60 50 40 30 20 10 ppm


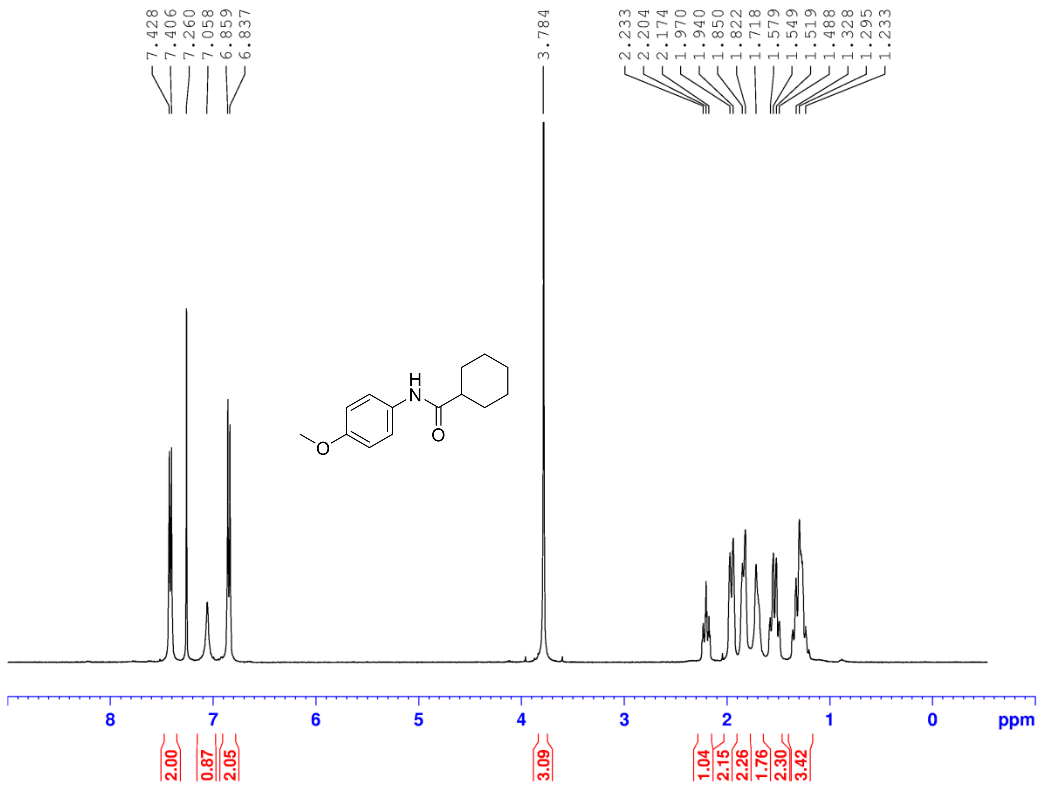

7.428
7.406
7.260
7.058
6.859
6.837
3.784
2.233
2.204
2.174
1.970
1.940
1.850
1.822
1.718
1.579
1.549
1.519
1.488
1.328
1.295
1.233
H
N
O
O
8 7 6 5 4 3 2 1 0 ppm
2.00
0.87
2.05
3.09
1.04
2.15
2.26
1.76
2.30
3.42

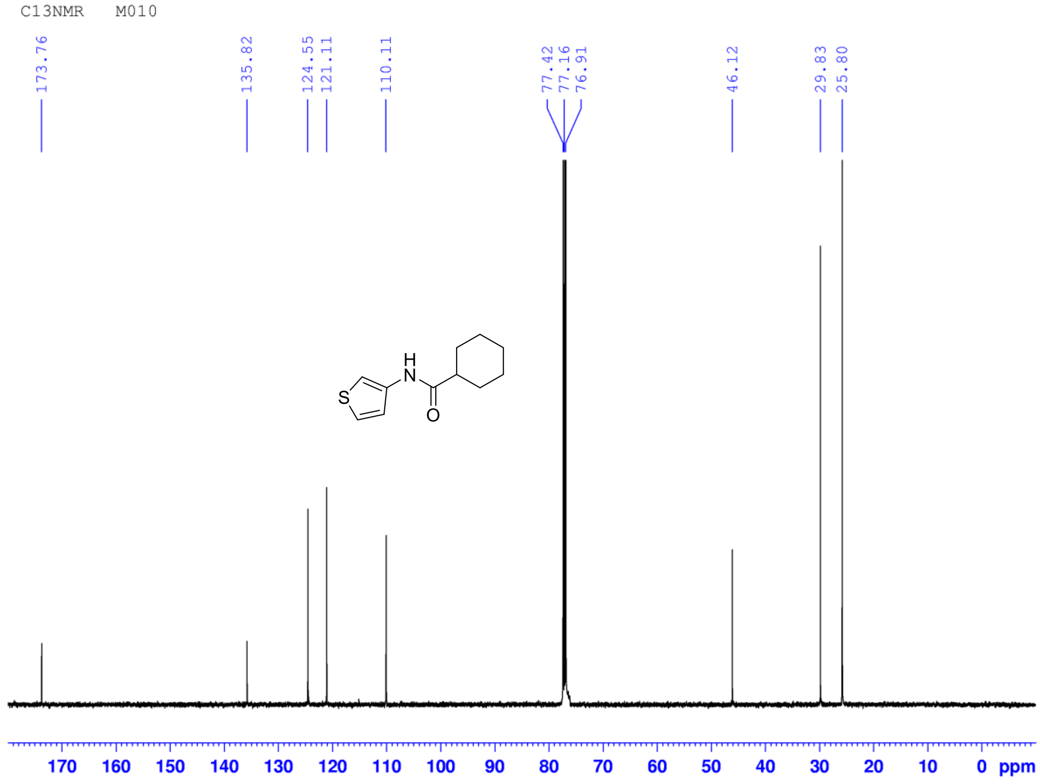
C13NMR M010
173.76
135.82
124.55
121.11
110.11
77.42
77.16
76.91
46.12
29.83
25.80
H
N
S
O
170 160 150 140 130 120 110 100 90 80 70 60 50 40 30 20 10 0 ppm

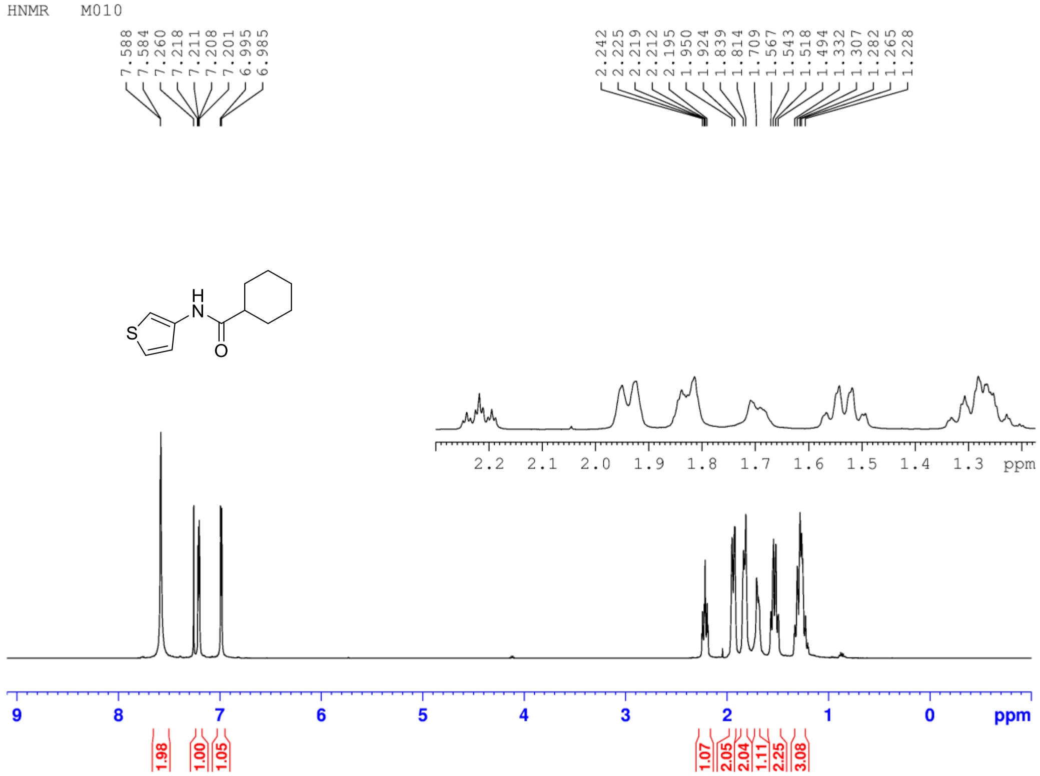
HNMR M010
7.588
7.584
7.260
7.218
7.211
7.208
7.201
6.995
6.985
2.242
2.225
2.219
2.212
2.195
1.950
1.924
1.839
1.814
1.709
1.567
1.543
1.518
1.494
1.332
1.307
1.282
1.265
1.228
H
N
S
O
2.2 2.1 2.0 1.9 1.8 1.7 1.6 1.5 1.4 1.3 ppm
9 8 7 6 5 4 3 2 1 0 ppm
1.98
1.00
1.05
1.07
2.05
2.04
1.11
2.25
3.08

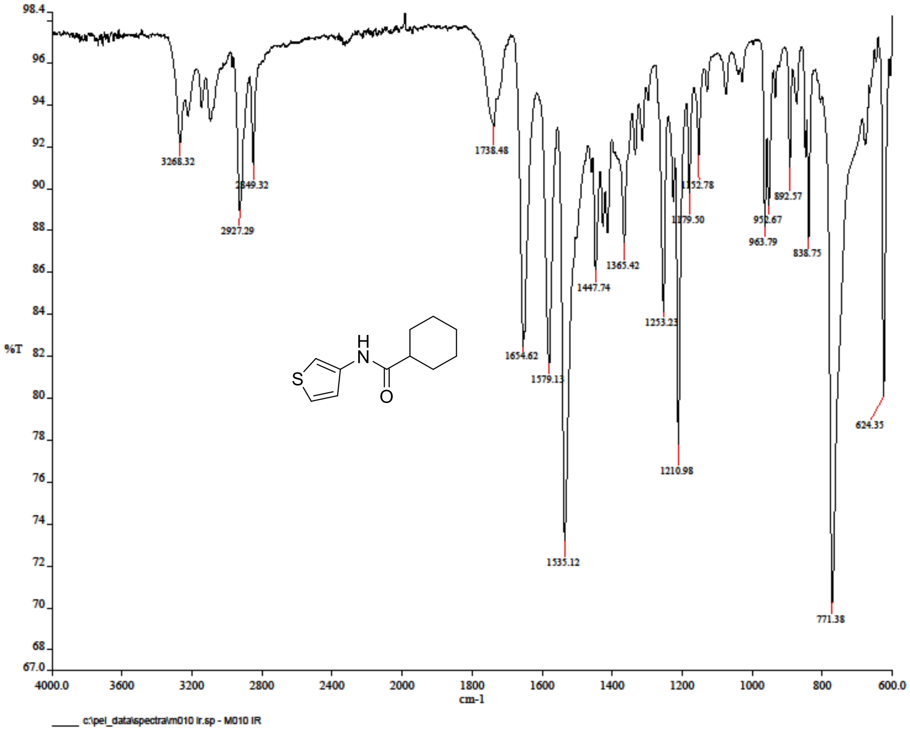

98.4
96
94
92
90
88
86
84
%T 82
80
78
76
74
72
70
68
67.0
4000.0
3600
3200
2800
2400
2000
1800
1600
1400
1200
1000
800
600.0
cm-1
3268.32
2927.29
2849.32
1738.48
1654.62
1579.13
1535.12
1447.74
1365.42
1253.23
1210.98
1179.50
1152.78
963.79
952.67
892.57
838.75
771.38
624.35
S
H
N
O
c:\pel_data\spectra\m010 ir.sp - M010 IR

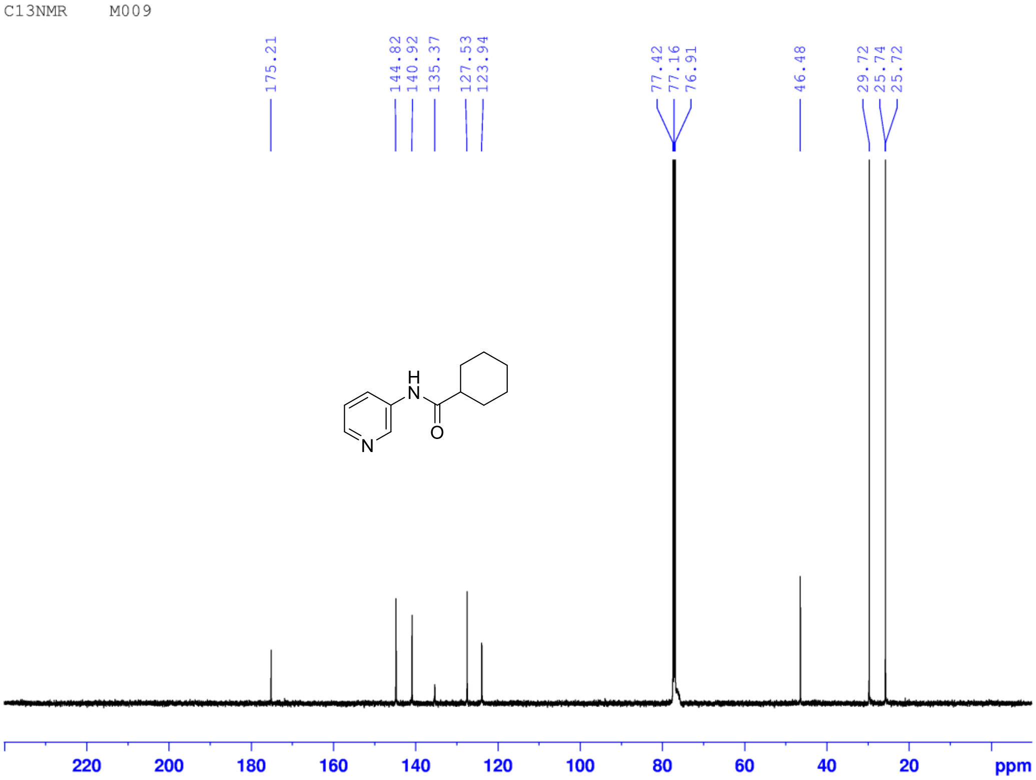
C13NMR M009
175.21
144.82
140.92
135.37
127.53
123.94
77.42
77.16
76.91
46.48
29.72
25.74
25.72
220
200
180
160
140
120
100
80
60
40
20
ppm

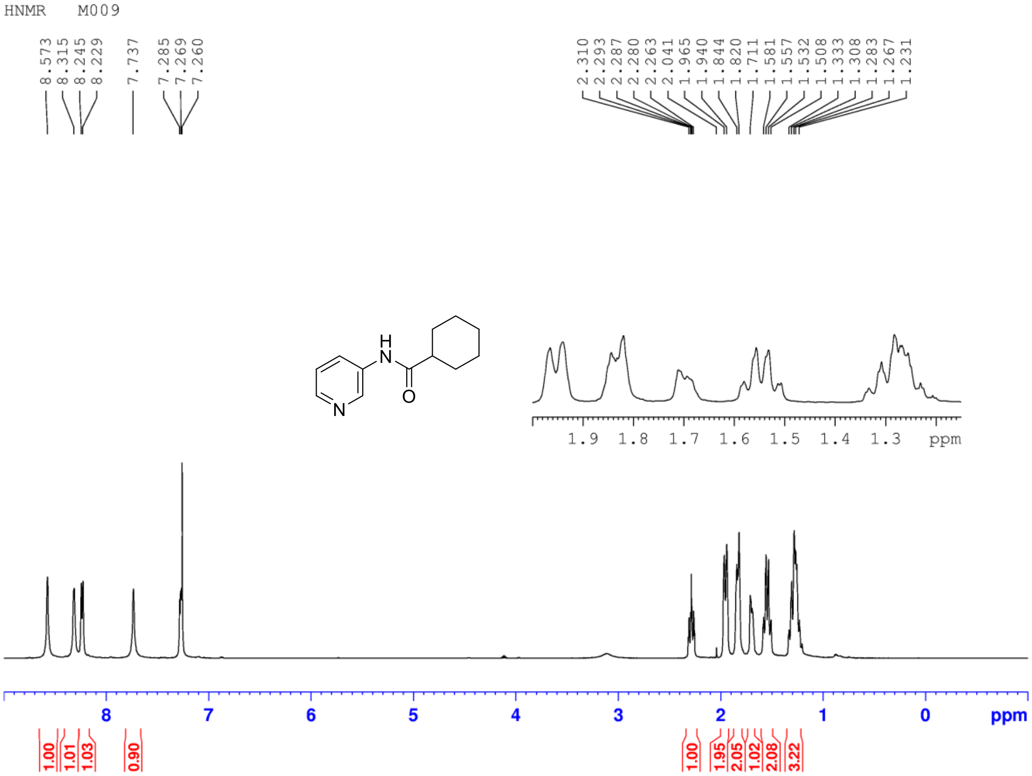
HNMR M009
8.573
8.315
8.245
8.229
7.737
7.285
7.269
7.260
2.310
2.293
2.287
2.280
2.263
2.041
1.965
1.940
1.844
1.820
1.711
1.581
1.557
1.532
1.508
1.333
1.308
1.283
1.267
1.231
1.9 1.8 1.7 1.6 1.5 1.4 1.3 ppm
8
7
6
5
4
3
2
1
0
ppm
1.00
1.01
1.03
0.90
1.00
1.95
2.05
1.02
2.08
3.22

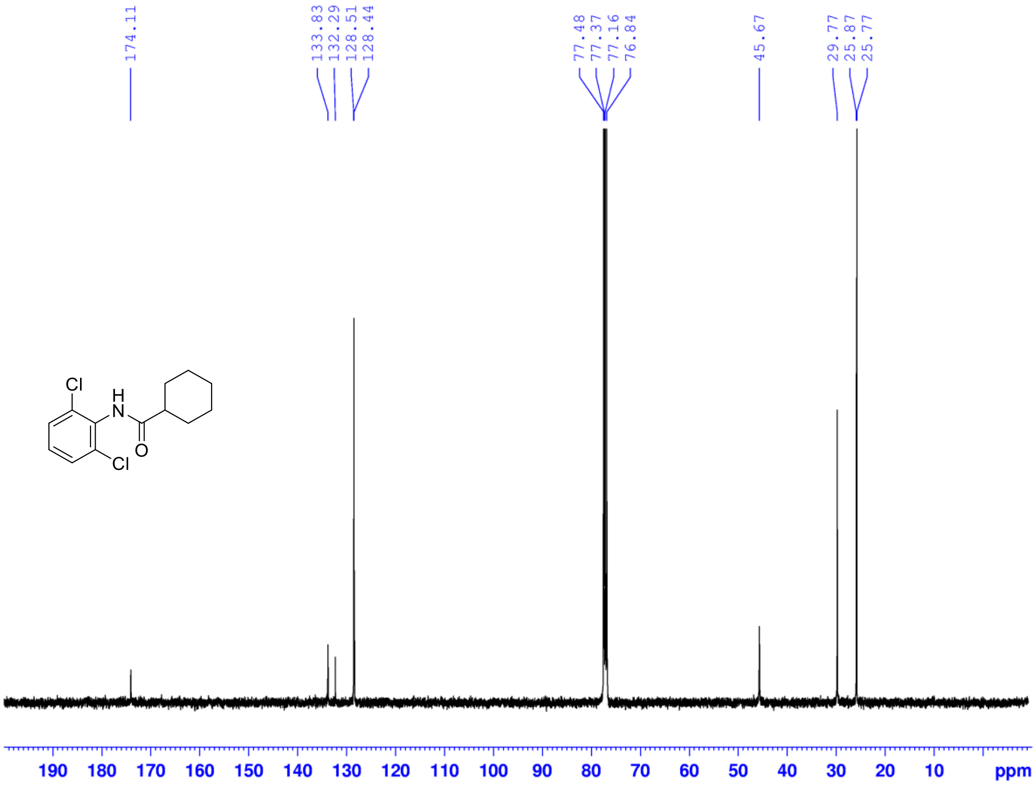
174.11
133.83
132.29
128.51
128.44
77.48
77.37
77.16
76.84
45.67
29.77
25.87
25.77
Cl
H
N
O
Cl
190 180 170 160 150 140 130 120 110 100 90 80 70 60 50 40 30 20 10 ppm

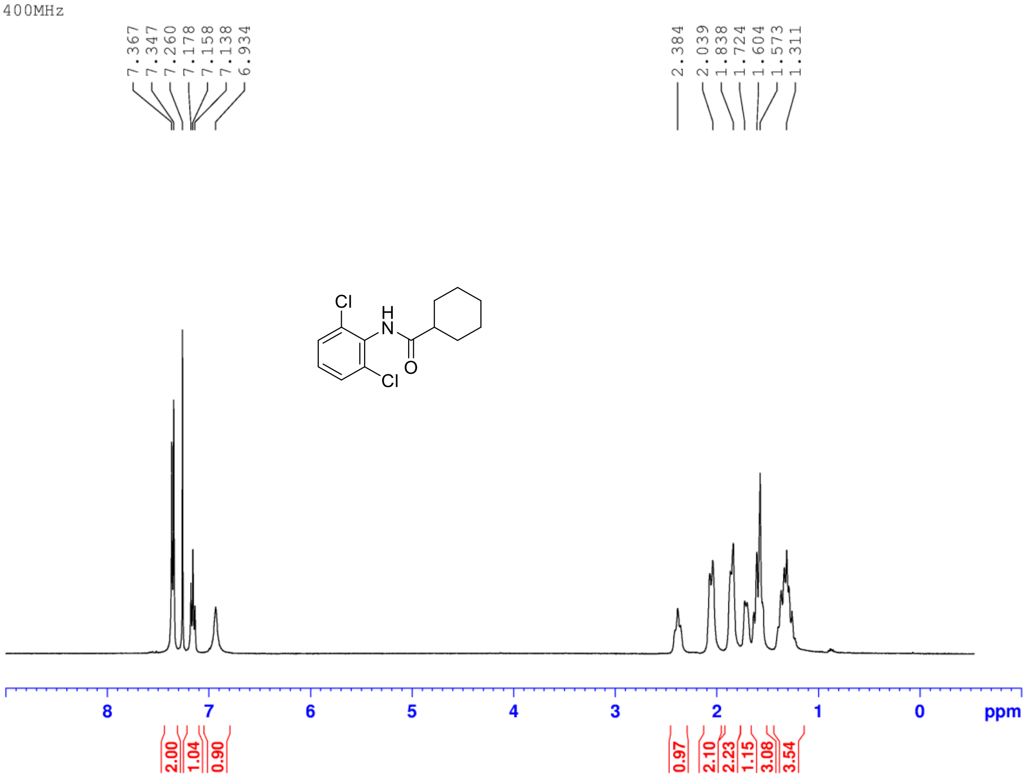
400MHz
7.367
7.347
7.260
7.178
7.158
7.138
6.934
2.384
2.039
1.838
1.724
1.604
1.573
1.311
Cl
H
N
O
Cl
8 7 6 5 4 3 2 1 0 ppm
2.00
1.04
0.90
0.97
2.10
2.23
1.15
3.08
3.54

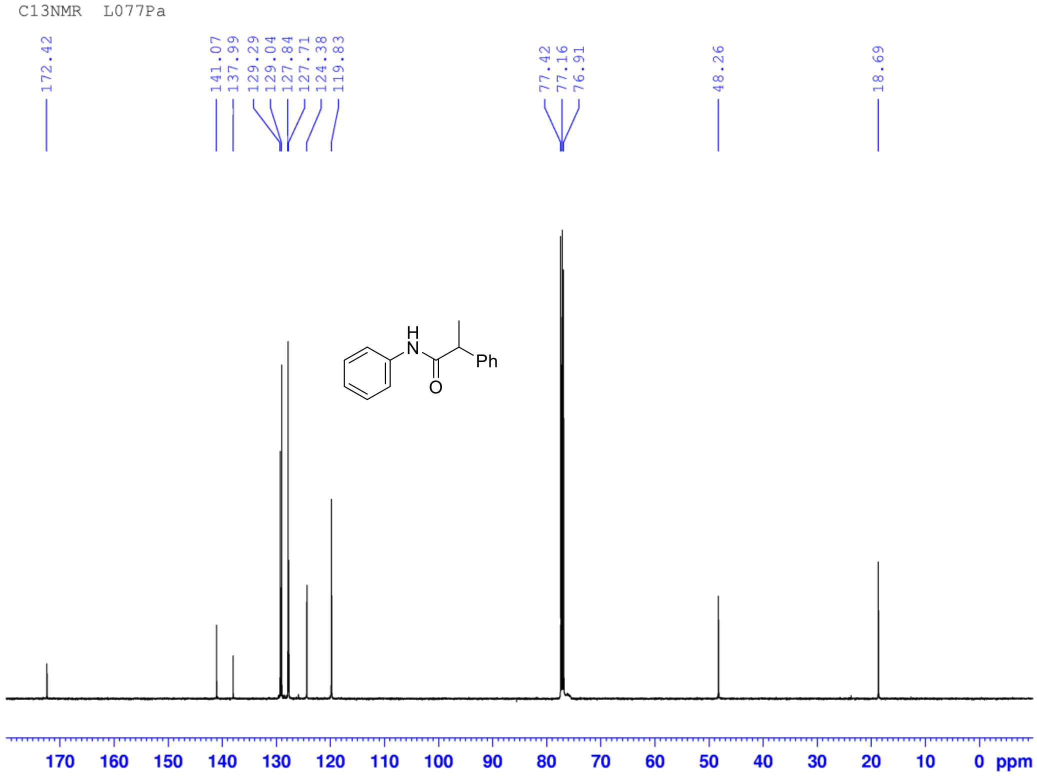
C13NMR L077Pa
172.42
141.07
137.99
129.29
129.04
127.84
127.71
124.38
119.83
77.42
77.16
76.91
48.26
18.69
H
N
Ph
O
170 160 150 140 130 120 110 100 90 80 70 60 50 40 30 20 10 0 ppm

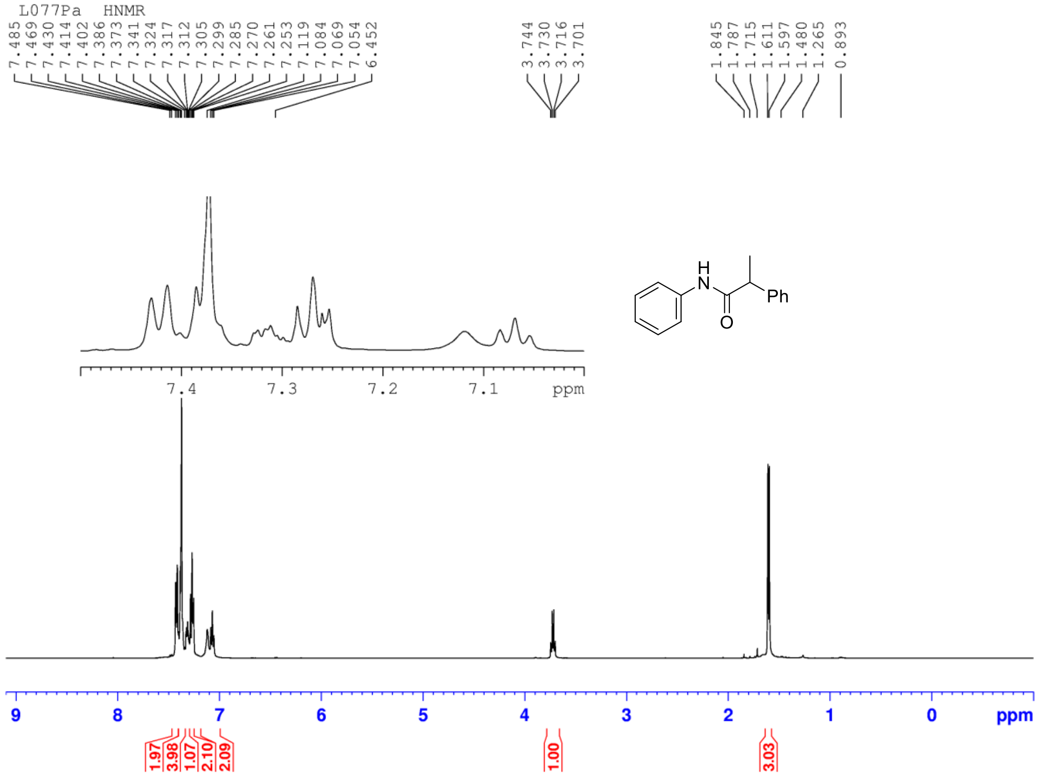
L077Pa HNMR
7.485
7.469
7.430
7.414
7.402
7.386
7.373
7.341
7.324
7.317
7.312
7.305
7.299
7.285
7.270
7.261
7.253
7.119
7.084
7.069
7.054
6.452
3.744
3.730
3.716
3.701
1.845
1.787
1.715
1.611
1.597
1.480
1.265
0.893
H
N
Ph
O
7.4 7.3 7.2 7.1 ppm
9 8 7 6 5 4 3 2 1 0 ppm
1.97
3.98
1.07
2.10
2.09
1.00
3.03

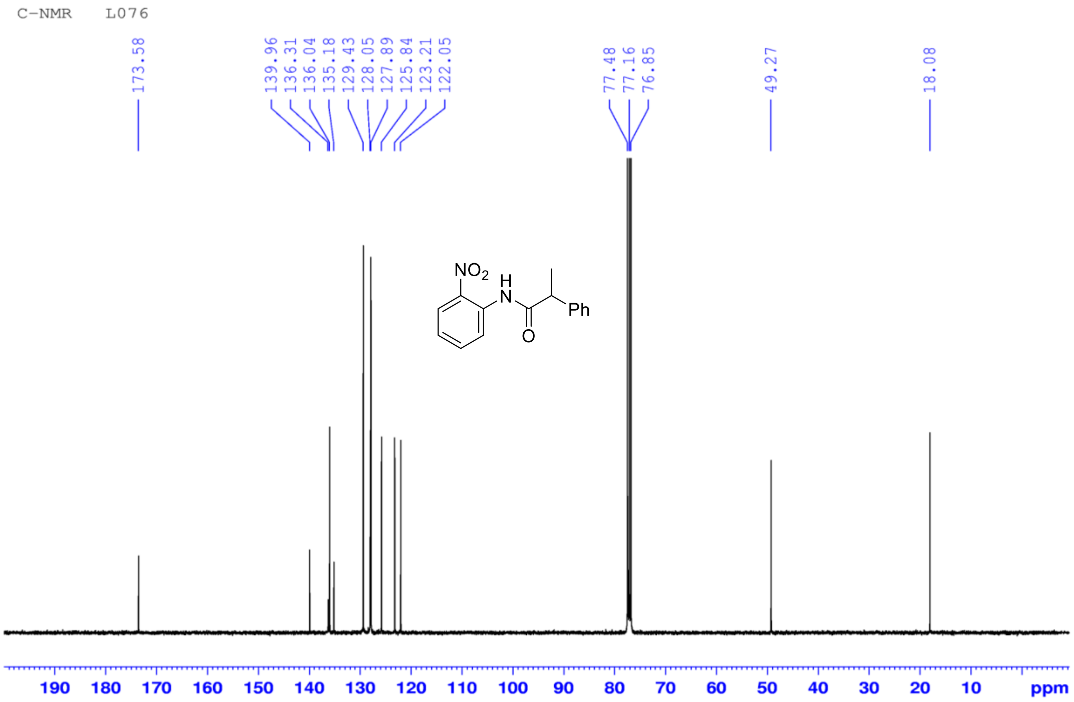
C-NMR L076
173.58
139.96
136.31
136.04
135.18
129.43
128.05
127.89
125.84
123.21
122.05
77.48
77.16
76.85
49.27
18.08
NO2
H
N
Ph
O
190 180 170 160 150 140 130 120 110 100 90 80 70 60 50 40 30 20 10 ppm

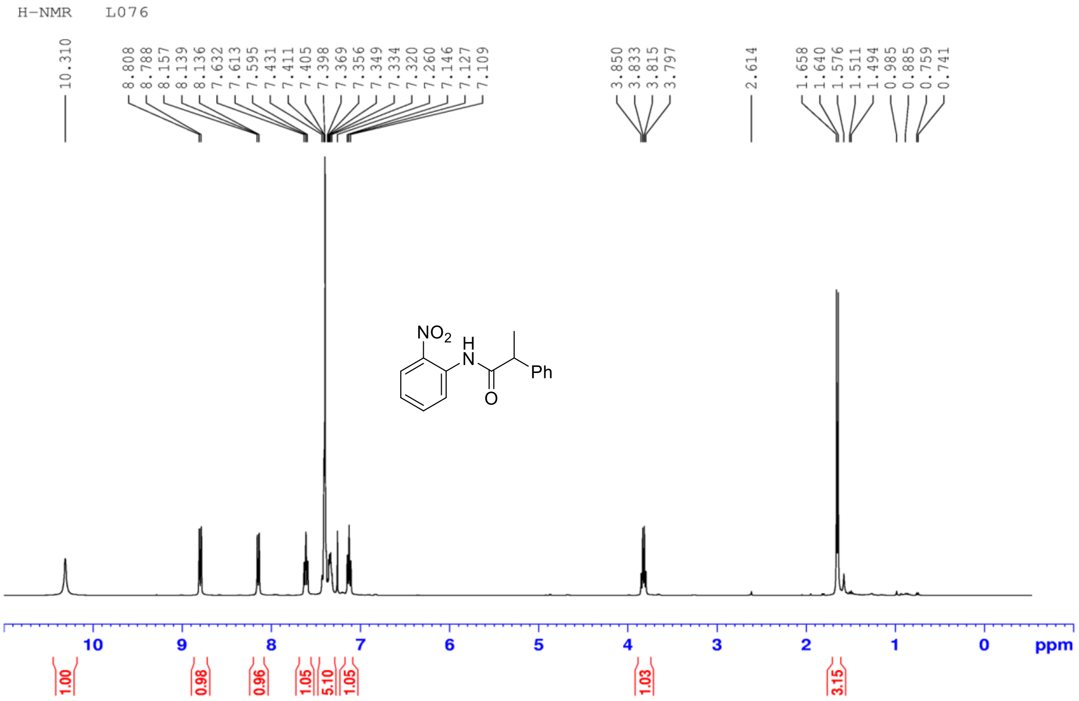
H-NMR L076
10.310
8.808
8.788
8.157
8.139
8.136
7.632
7.613
7.595
7.431
7.411
7.405
7.398
7.369
7.356
7.349
7.334
7.320
7.260
7.146
7.127
7.109
3.850
3.833
3.815
3.797
2.614
1.658
1.640
1.576
1.511
1.494
0.985
0.885
0.759
0.741
NO2
H
N
Ph
O
10 9 8 7 6 5 4 3 2 1 0 ppm
1.00
0.98
0.96
1.05
5.10
1.05
1.03
3.15

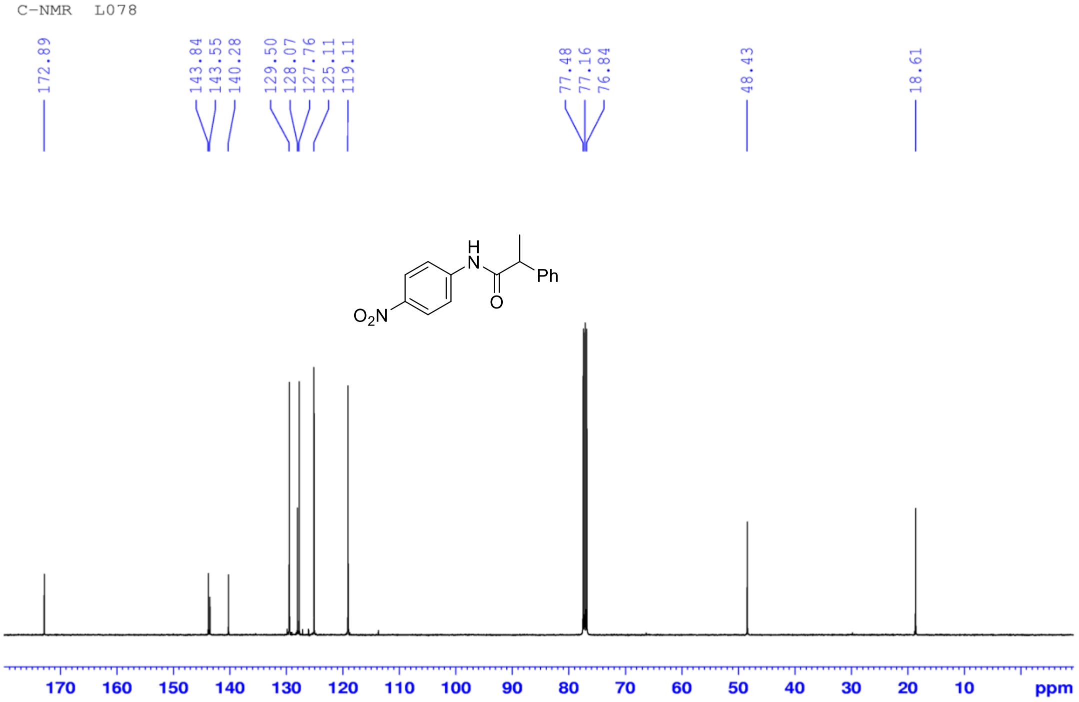
C-NMR L078
172.89
143.84
143.55
140.28
129.50
128.07
127.76
125.11
119.11
77.48
77.16
76.84
48.43
18.61
H
N
Ph
O
O2N
170
160
150
140
130
120
110
100
90
80
70
60
50
40
30
20
10
ppm

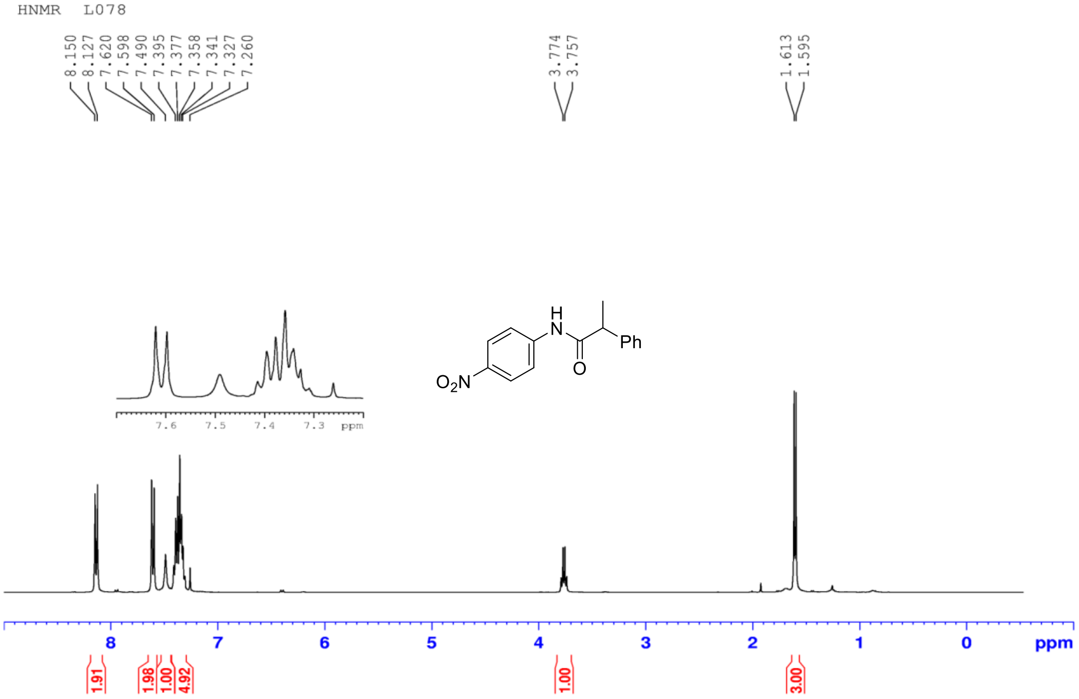
HNMR L078
8.150
8.127
7.620
7.598
7.490
7.395
7.377
7.358
7.341
7.327
7.260
3.774
3.757
1.613
1.595
H
N
Ph
O
O2N
7.6
7.5
7.4
7.3
ppm
8
7
6
5
4
3
2
1
0
ppm
1.91
1.98
1.00
4.92
1.00
3.00

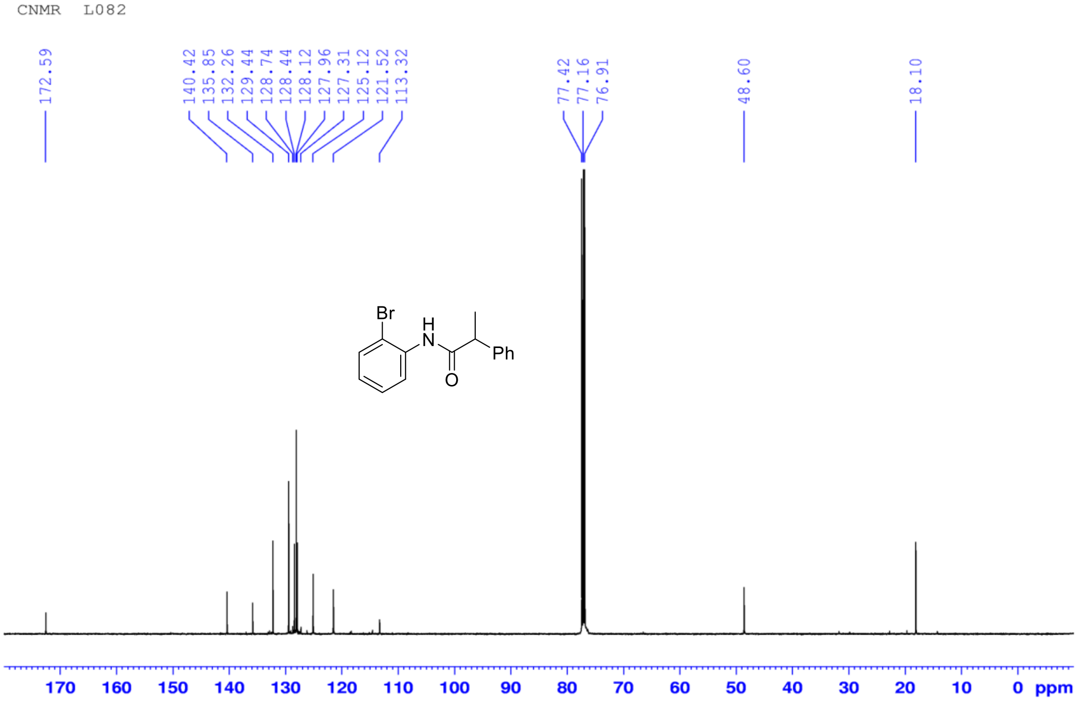
CNMR L082
172.59
140.42
135.85
132.26
129.44
128.74
128.44
128.12
127.96
127.31
125.12
121.52
113.32
77.42
77.16
76.91
48.60
18.10
Br
H
N
Ph
O
170
160
150
140
130
120
110
100
90
80
70
60
50
40
30
20
10
0 ppm

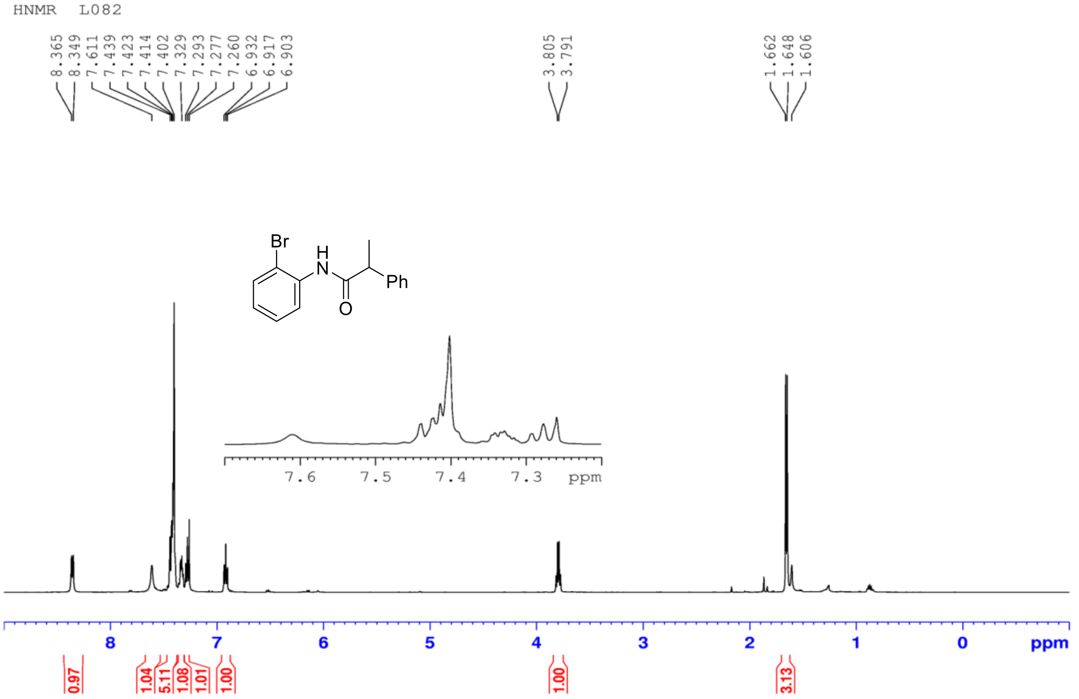
HNMR L082
8.365
8.349
7.611
7.439
7.423
7.414
7.402
7.329
7.293
7.277
7.260
6.932
6.917
6.903
3.805
3.791
1.662
1.648
1.606
Br
H
N
Ph
O
7.6
7.5
7.4
7.3
ppm
8
7
6
5
4
3
2
1
0
ppm
0.97
1.04
5.11
1.08
1.01
1.00
1.00
3.13

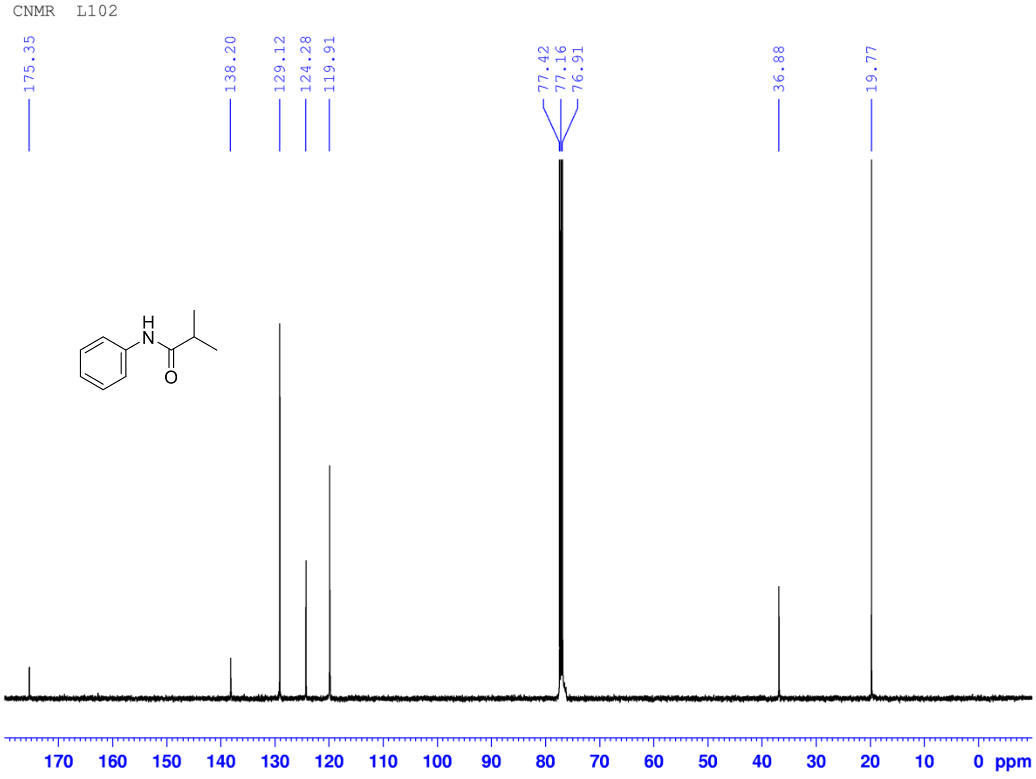
CNMR L102
175.35
138.20
129.12
124.28
119.91
77.42
77.16
76.91
36.88
19.77
H
N
O
170 160 150 140 130 120 110 100 90 80 70 60 50 40 30 20 10 0 ppm

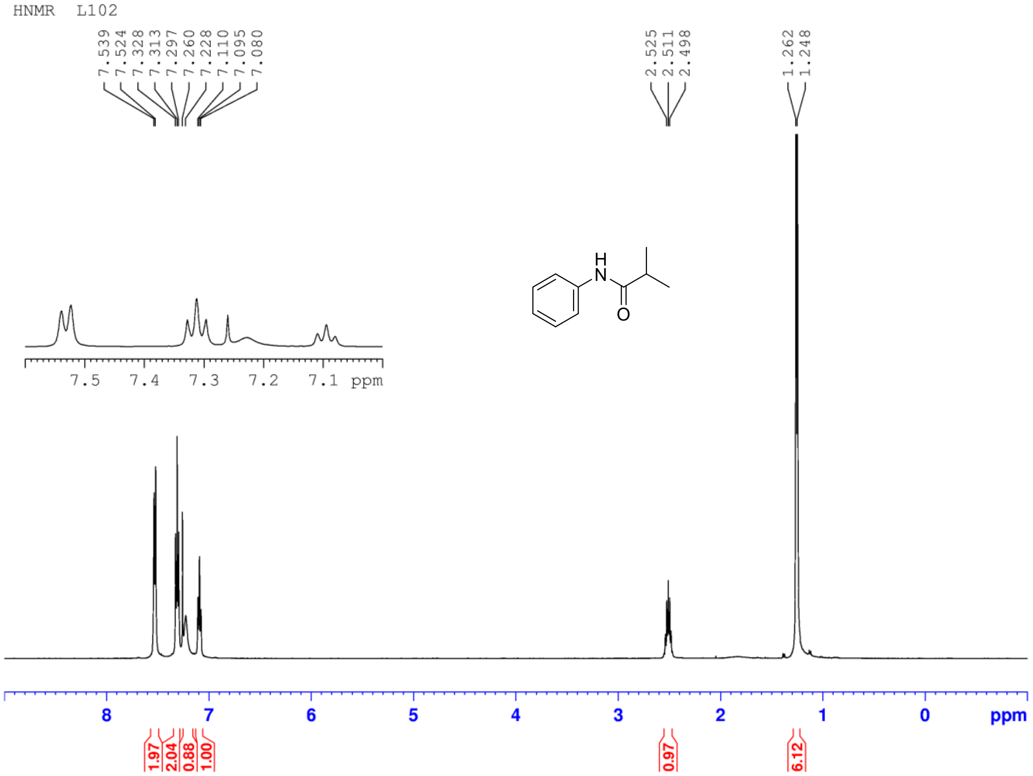
HNMR L102
7.539
7.524
7.328
7.313
7.297
7.260
7.228
7.110
7.095
7.080
2.525
2.511
2.498
1.262
1.248
H
N
O
7.5 7.4 7.3 7.2 7.1 ppm
8 7 6 5 4 3 2 1 0 ppm
1.97
2.04
0.88
1.00
0.97
6.12

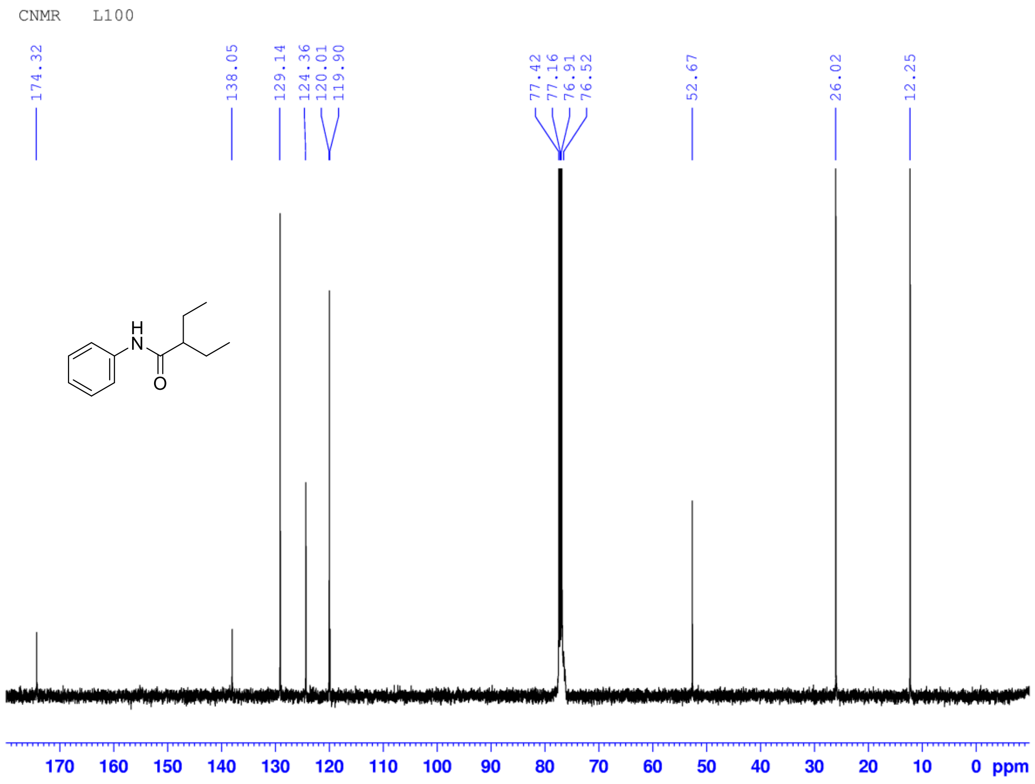
CNMR L100
174.32
138.05
129.14
124.36
120.01
119.90
77.42
77.16
76.91
76.52
52.67
26.02
12.25
H
N
O
170
160
150
140
130
120
110
100
90
80
70
60
50
40
30
20
10
0 ppm

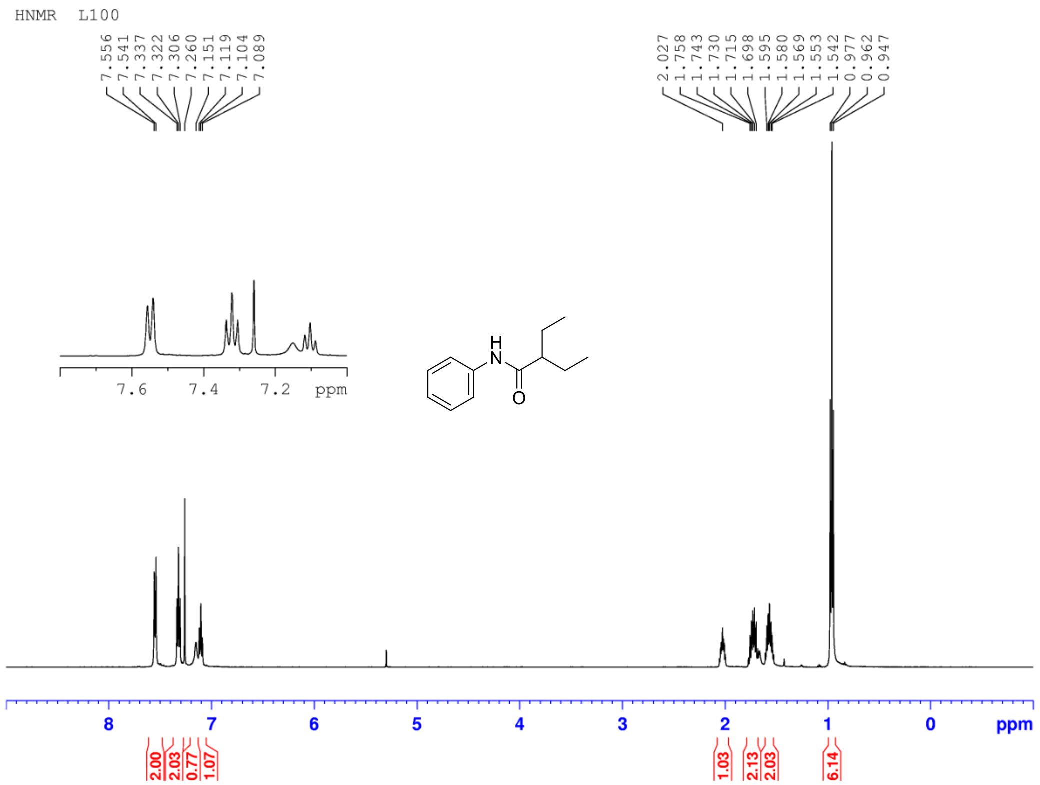
HNMR L100
7.556
7.541
7.337
7.322
7.306
7.260
7.151
7.119
7.104
7.089
2.027
1.758
1.743
1.730
1.715
1.698
1.595
1.580
1.569
1.553
1.542
0.977
0.962
0.947
7.6
7.4
7.2
ppm
H
N
O
8
7
6
5
4
3
2
1
0
ppm
2.00
2.03
0.77
1.07
1.03
2.13
2.03
6.14

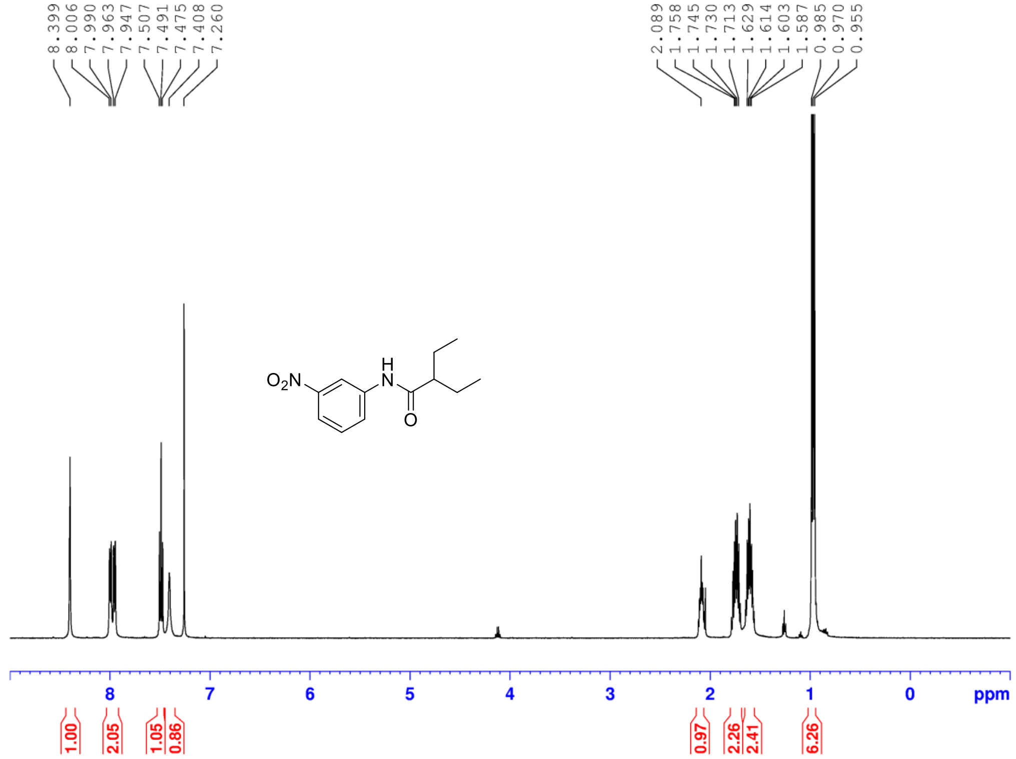
8.399
8.006
7.990
7.963
7.947
7.507
7.491
7.475
7.408
7.260
2.089
1.758
1.745
1.730
1.713
1.629
1.614
1.603
1.587
0.985
0.970
0.955
$O_2N$
H
N
O
8
7
6
5
4
3
2
1
0
ppm
1.00
2.05
1.05
0.86
0.97
2.26
2.41
6.26

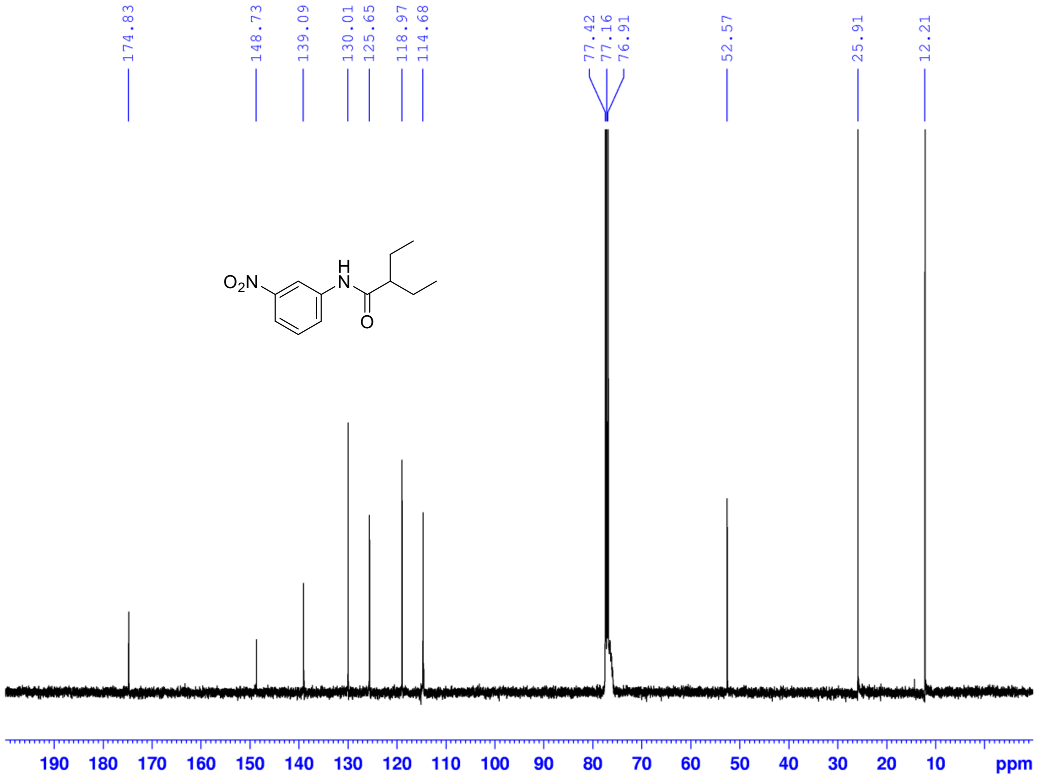
174.83
148.73
139.09
130.01
125.65
118.97
114.68
77.42
77.16
76.91
52.57
25.91
12.21
$O_2N$
H
N
O
190
180
170
160
150
140
130
120
110
100
90
80
70
60
50
40
30
20
10
ppm

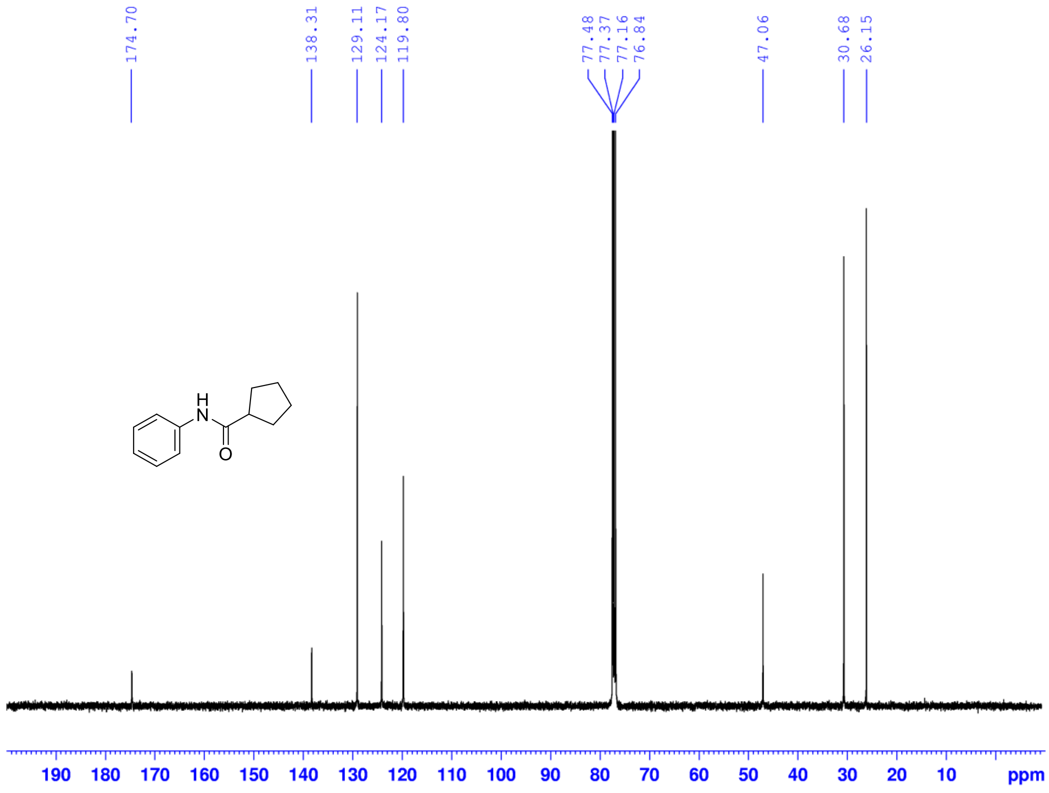
174.70
138.31
129.11
124.17
119.80
77.48
77.37
77.16
76.84
47.06
30.68
26.15
190 180 170 160 150 140 130 120 110 100 90 80 70 60 50 40 30 20 10 ppm

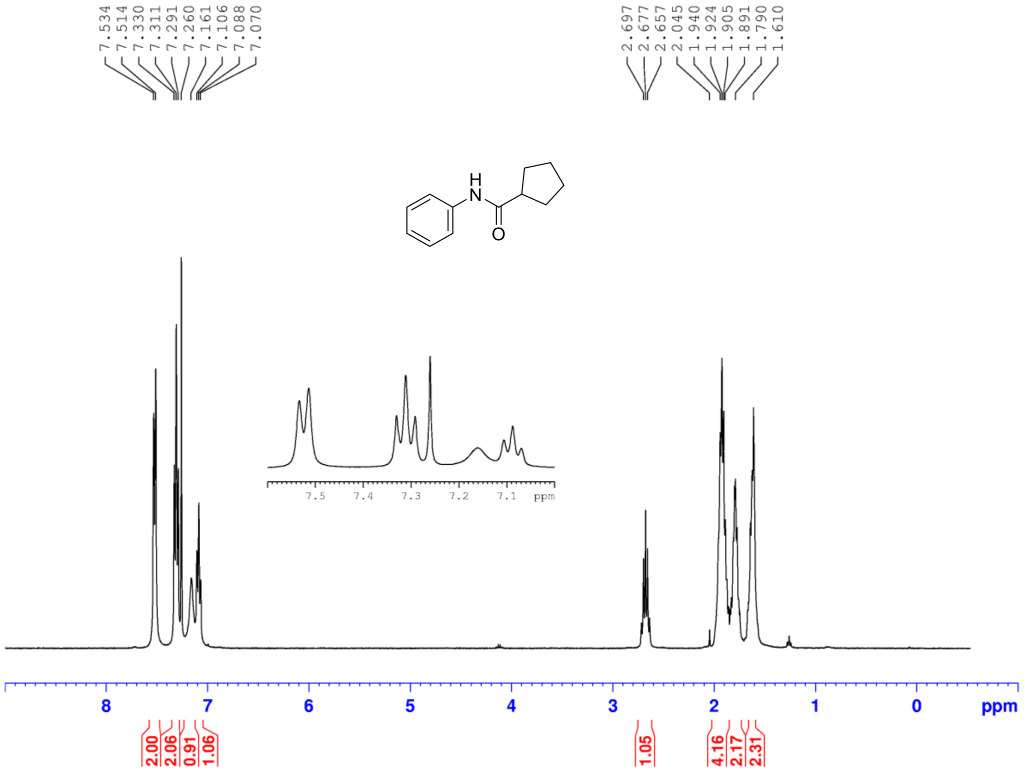
7.534
7.514
7.330
7.311
7.291
7.260
7.161
7.106
7.088
7.070
2.697
2.677
2.657
2.045
1.940
1.924
1.905
1.891
1.790
1.610
7.5 7.4 7.3 7.2 7.1 ppm
8 7 6 5 4 3 2 1 0 ppm
2.00
2.06
0.91
1.06
1.05
4.16
2.17
2.31

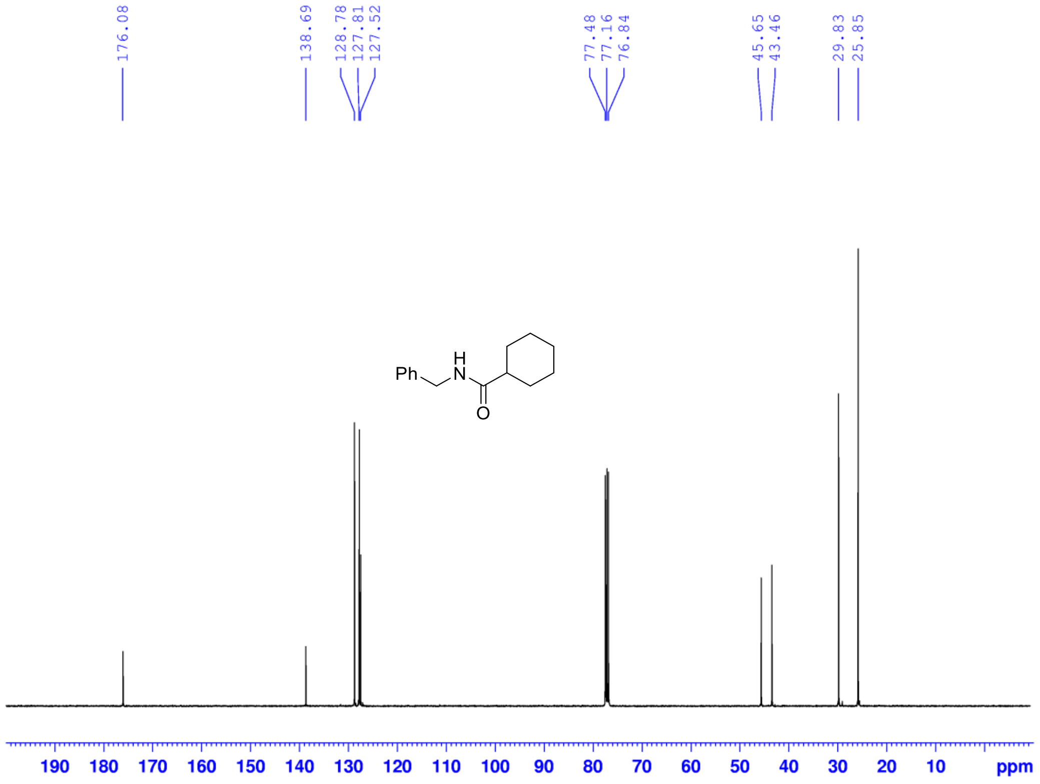
176.08
138.69
128.78
127.81
127.52
77.48
77.16
76.84
45.65
43.46
29.83
25.85
Ph
H
N
O
190 180 170 160 150 140 130 120 110 100 90 80 70 60 50 40 30 20 10 ppm

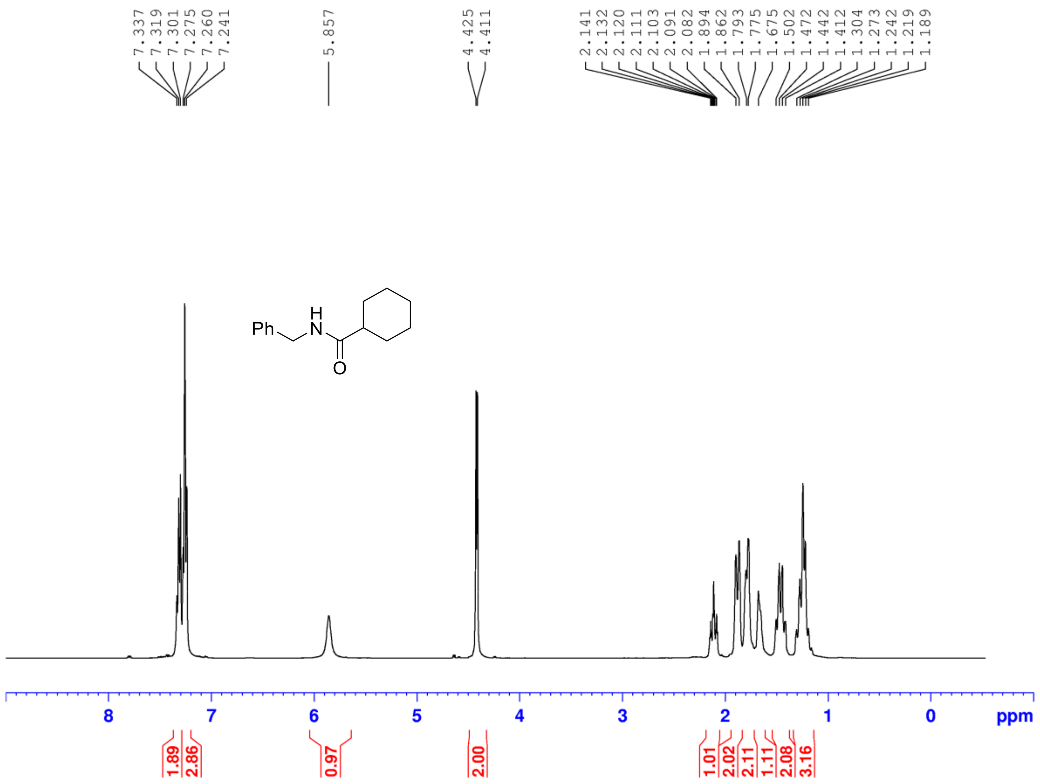
7.337
7.319
7.301
7.275
7.260
7.241
5.857
4.425
4.411
2.141
2.132
2.120
2.111
2.103
2.091
2.082
1.894
1.862
1.793
1.775
1.675
1.502
1.472
1.442
1.412
1.304
1.273
1.242
1.219
1.189
Ph
H
N
O
8 7 6 5 4 3 2 1 0 ppm
1.89
2.86
0.97
2.00
1.01
2.02
2.11
1.11
2.08
3.16

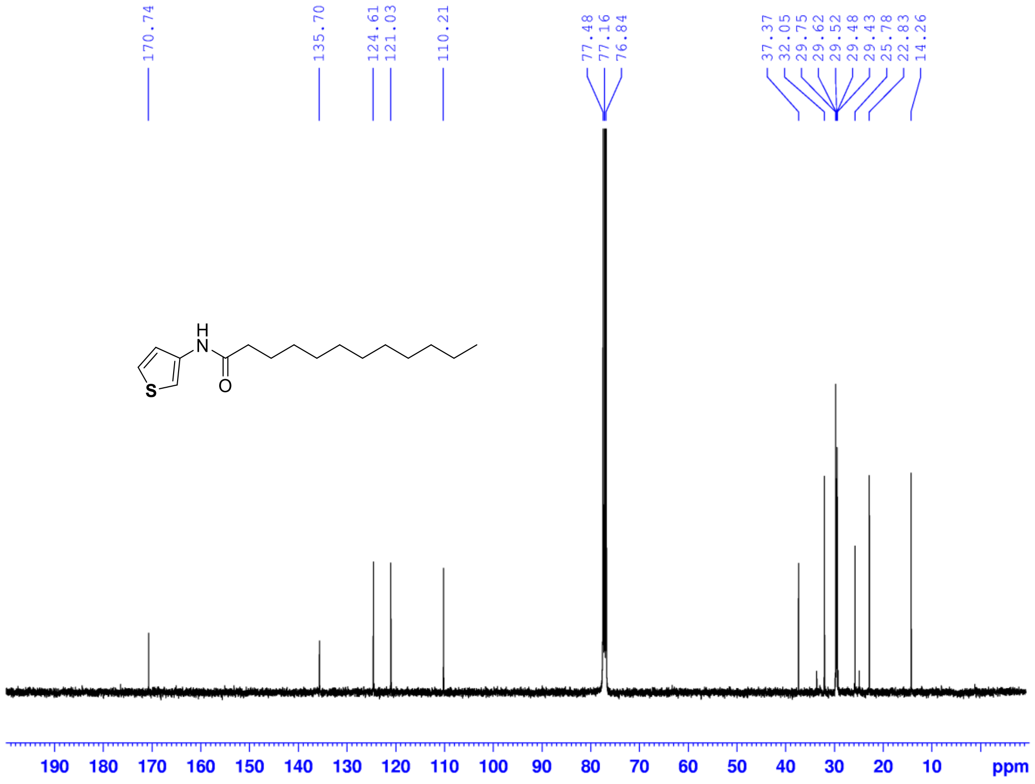

170.74
135.70
124.61
121.03
110.21
77.48
77.16
76.84
37.37
32.05
29.75
29.62
29.52
29.48
29.43
25.78
22.83
14.26
H
N
S
O
190 180 170 160 150 140 130 120 110 100 90 80 70 60 50 40 30 20 10 ppm


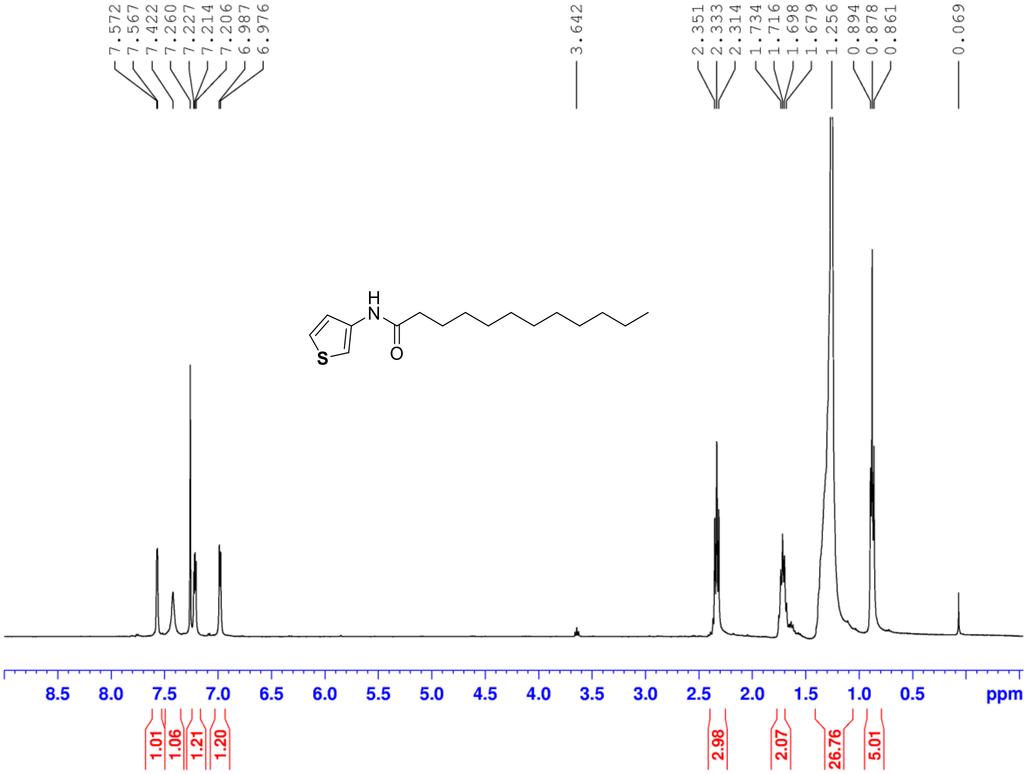

7.572
7.567
7.422
7.260
7.227
7.214
7.206
6.987
6.976
3.642
2.351
2.333
2.314
1.734
1.716
1.698
1.679
1.256
0.894
0.878
0.861
0.069
H
N
S
O
8.5 8.0 7.5 7.0 6.5 6.0 5.5 5.0 4.5 4.0 3.5 3.0 2.5 2.0 1.5 1.0 0.5 ppm
1.01
1.06
1.21
1.20
2.98
2.07
26.76
5.01

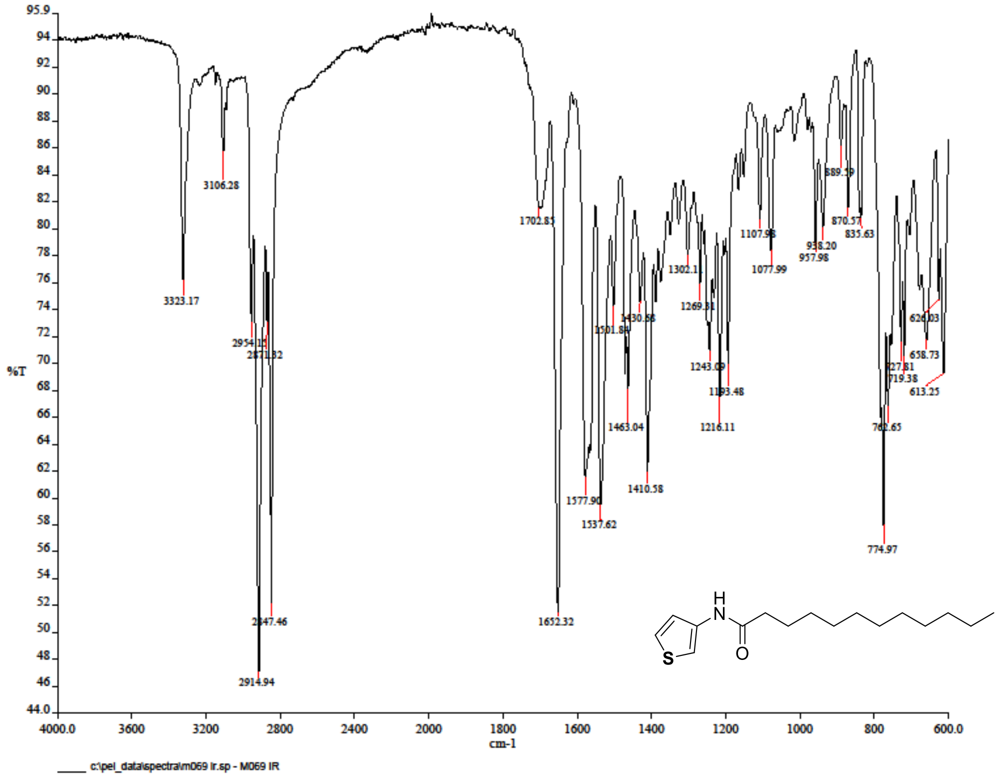

%T
cm-1
3323.17
3106.28
2954.15
2871.32
2914.94
2847.46
1702.85
1652.32
1577.90
1537.62
1501.84
1463.04
1430.58
1410.58
1302.1
1269.31
1243.09
1216.11
1193.48
1107.98
1077.99
958.20
957.98
889.19
870.57
835.63
774.97
762.65
727.81
719.38
658.73
626.03
613.25
H
N
O
S
c:\pel_data\spectra\m069 ir.sp - M069 IR

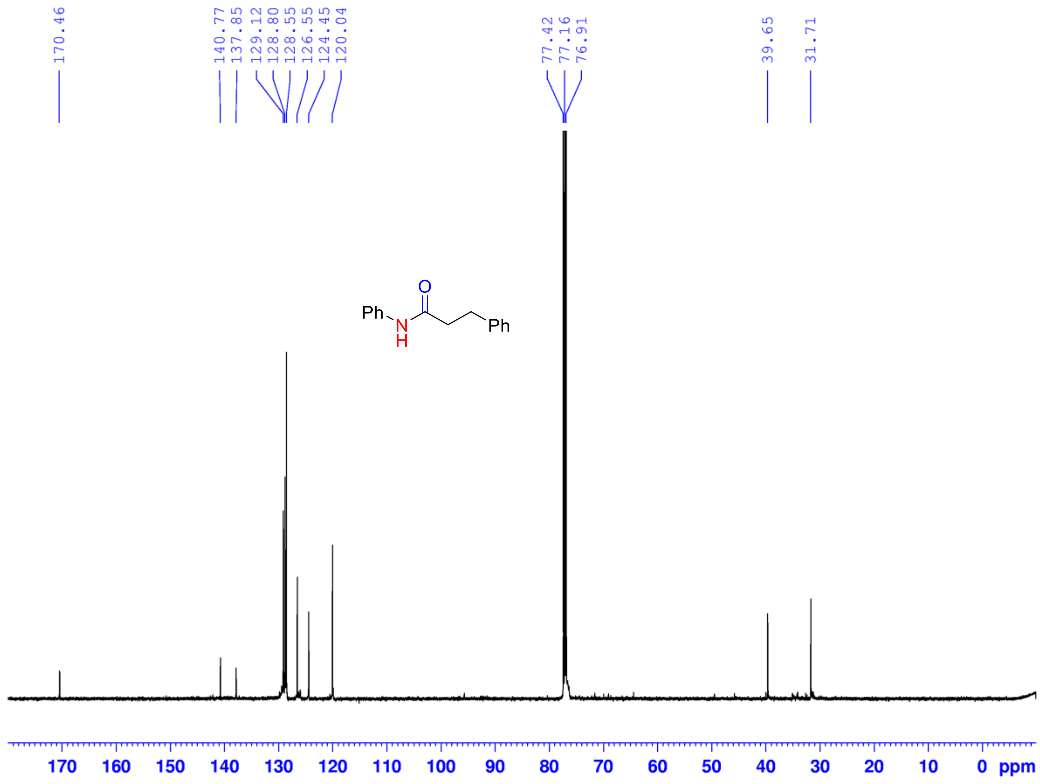
170.46
140.77
137.85
129.12
128.80
128.55
126.55
124.45
120.04
77.42
77.16
76.91
39.65
31.71
Ph
N
H
O
Ph
170
160
150
140
130
120
110
100
90
80
70
60
50
40
30
20
10
0
ppm

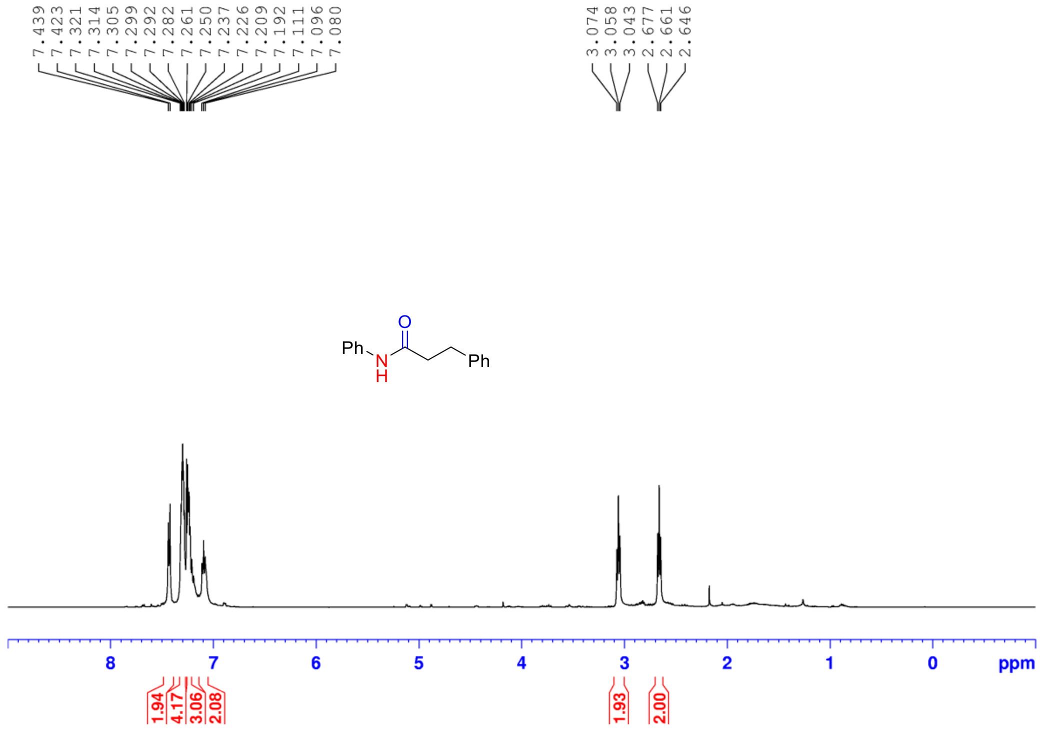
7.439
7.423
7.321
7.314
7.305
7.299
7.292
7.282
7.261
7.250
7.237
7.226
7.209
7.192
7.111
7.096
7.080
3.074
3.058
3.043
2.677
2.661
2.646
Ph
N
H
O
Ph
8
7
6
5
4
3
2
1
0
ppm
1.94
4.17
3.06
2.08
1.93
2.00

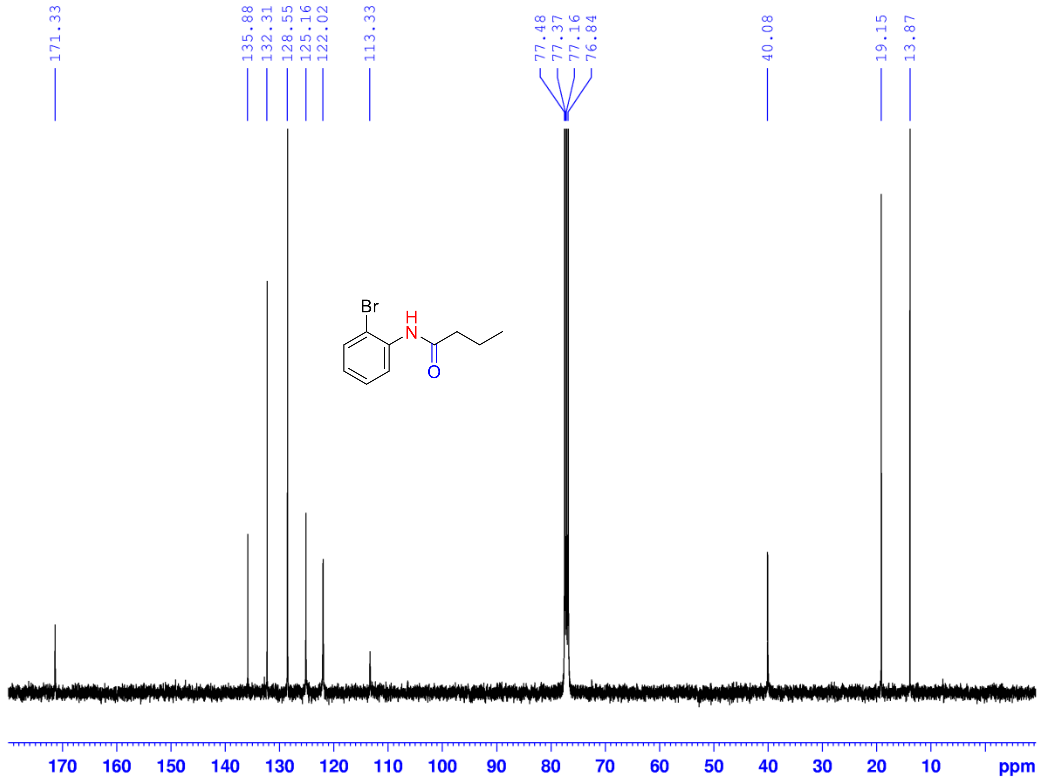
171.33
135.88
132.31
128.55
125.16
122.02
113.33
77.48
77.37
77.16
76.84
40.08
19.15
13.87
Br
H
N
O
170 160 150 140 130 120 110 100 90 80 70 60 50 40 30 20 10 ppm

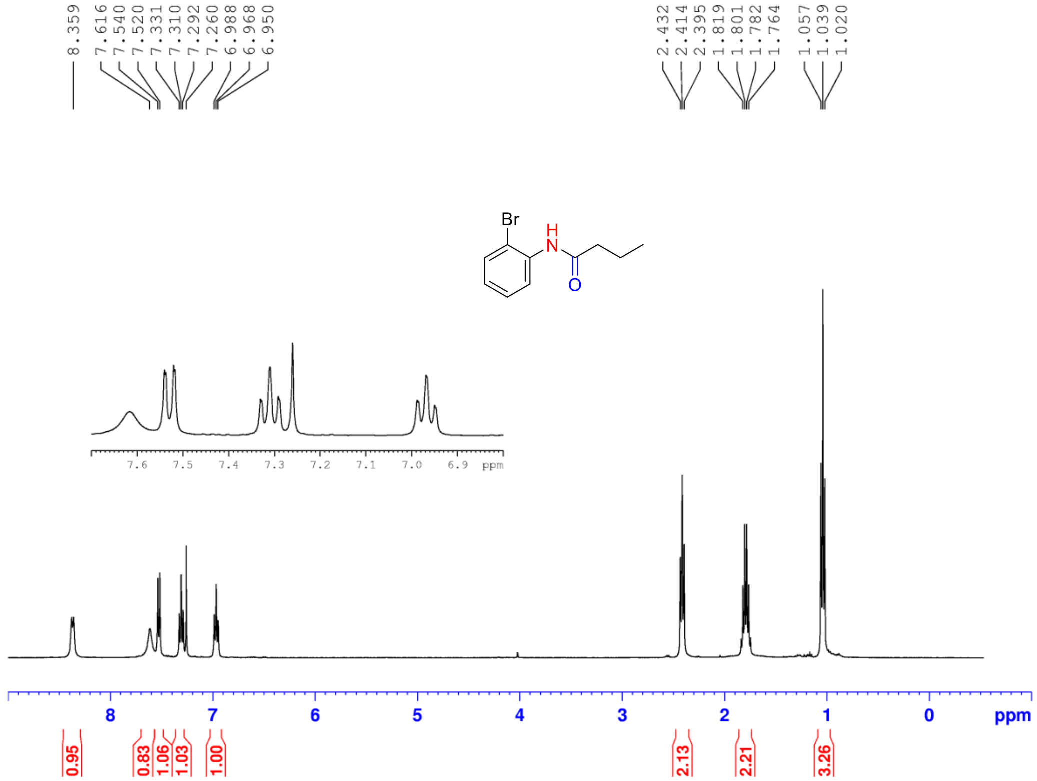
8.359
7.616
7.540
7.520
7.331
7.310
7.292
7.260
6.988
6.968
6.950
2.432
2.414
2.395
1.819
1.801
1.782
1.764
1.057
1.039
1.020
Br
H
N
O
7.6 7.5 7.4 7.3 7.2 7.1 7.0 6.9 ppm
8 7 6 5 4 3 2 1 0 ppm
0.95
0.83
1.06
1.03
1.00
2.13
2.21
3.26

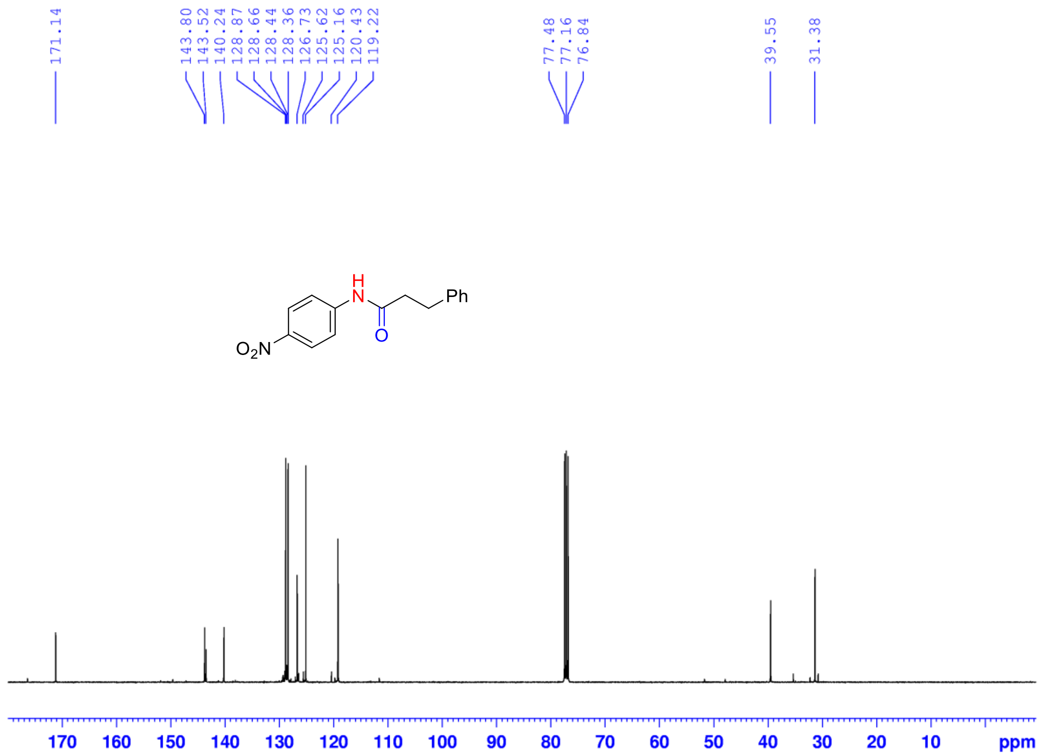
171.14
143.80
143.52
140.24
128.87
128.66
128.44
128.36
126.73
125.62
125.16
120.43
119.22
77.48
77.16
76.84
39.55
31.38
H
N
Ph
O
$O_2N$
170 160 150 140 130 120 110 100 90 80 70 60 50 40 30 20 10 ppm

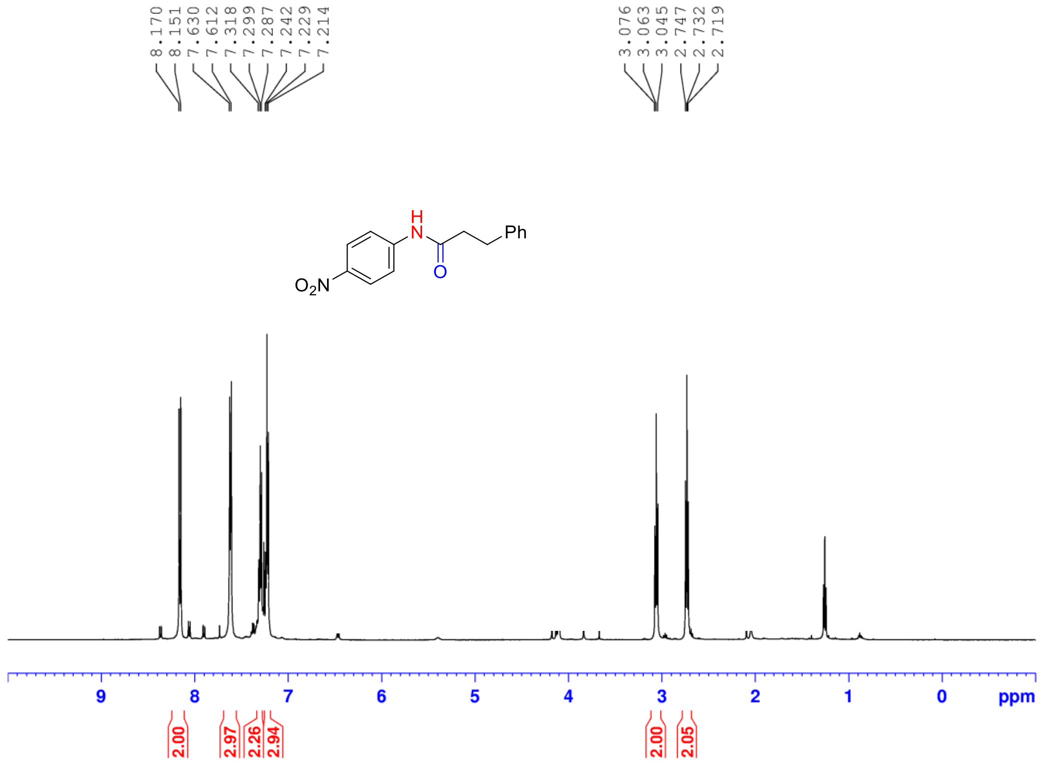
8.170
8.151
7.630
7.612
7.318
7.299
7.287
7.242
7.229
7.214
3.076
3.063
3.045
2.747
2.732
2.719
H
N
Ph
O
$O_2N$
9 8 7 6 5 4 3 2 1 0 ppm
2.00
2.97
2.26
2.94
2.00
2.05

F19 CDCl3

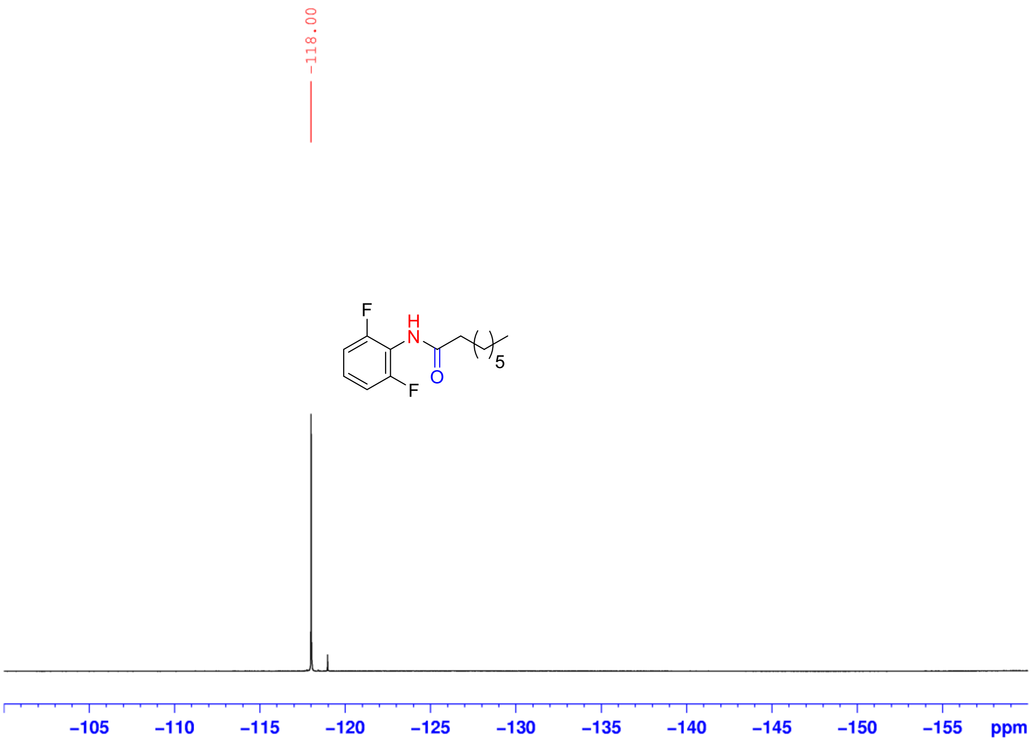
-118.00
F
H
N
O
5
F
-105
-110
-115
-120
-125
-130
-135
-140
-145
-150
-155
ppm

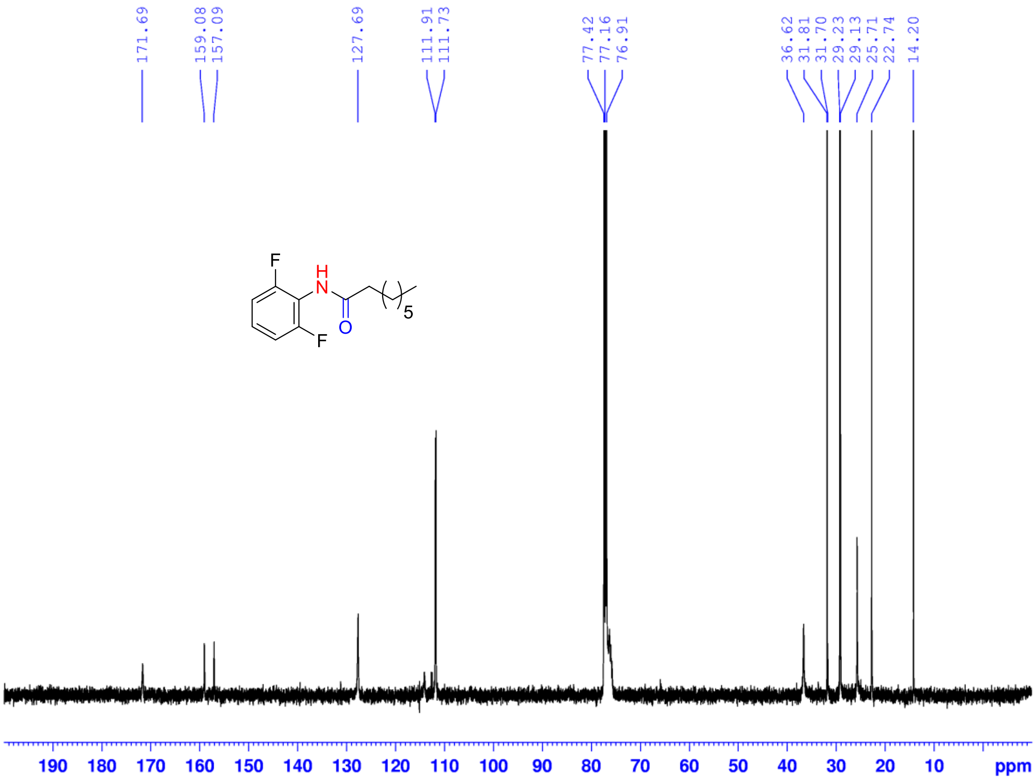
171.69
159.08
157.09
127.69
111.91
111.73
77.42
77.16
76.91
36.62
31.81
31.70
29.23
29.13
25.71
22.74
14.20
F
H
N
O
5
F
190
180
170
160
150
140
130
120
110
100
90
80
70
60
50
40
30
20
10
ppm

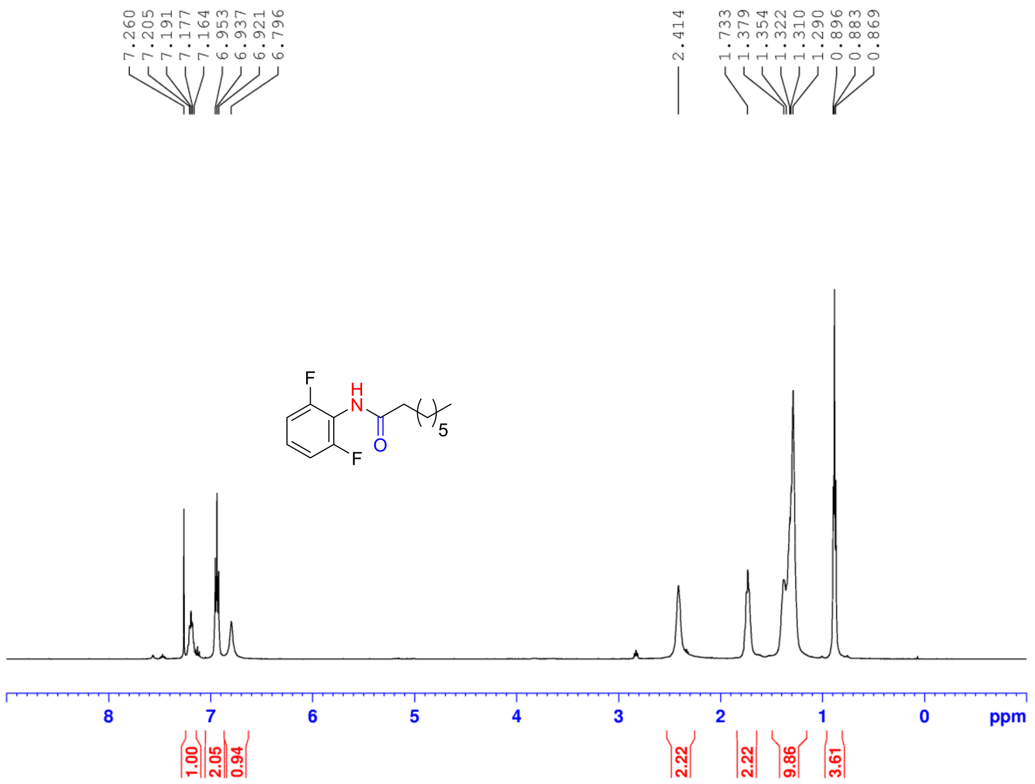
7.260
7.205
7.191
7.177
7.164
6.953
6.937
6.921
6.796
2.414
1.733
1.379
1.354
1.322
1.310
1.290
0.896
0.883
0.869
F
H
N
O
5
F
ppm
1.00
2.05
0.94
2.22
2.22
9.86
3.61

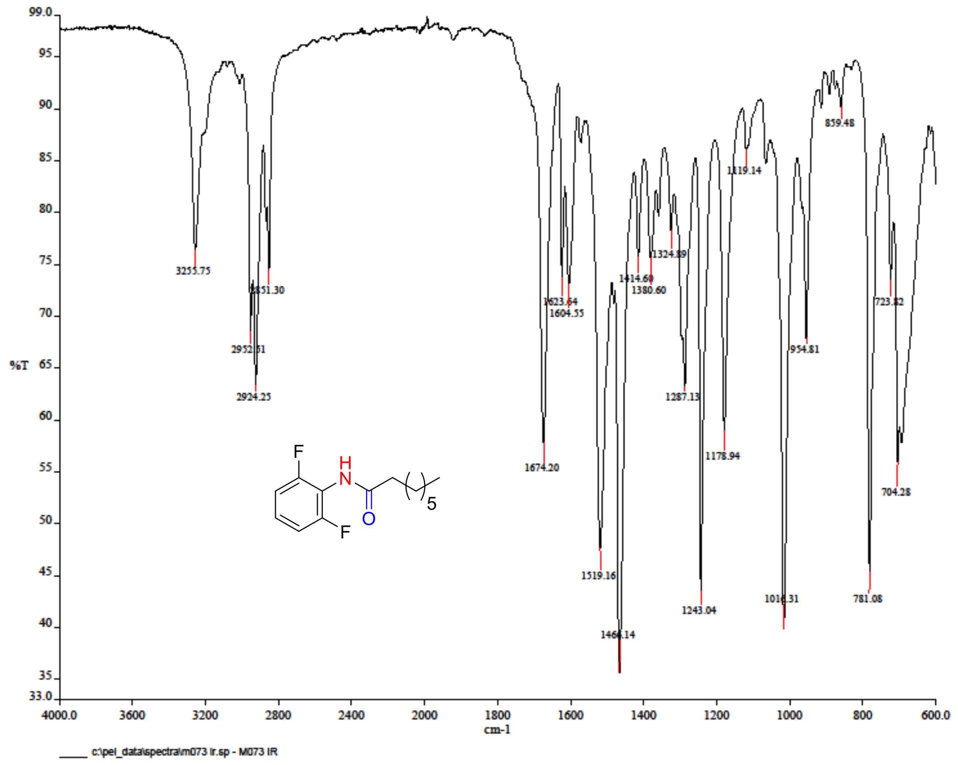
%T
cm-1
3255.75
2952.51
2924.25
2851.30
1674.20
1623.64
1604.55
1519.16
1464.14
1414.60
1380.60
1324.89
1287.13
1243.04
1178.94
1119.14
1018.31
954.81
859.48
781.08
723.82
704.28
c:\pel_data\spectra\m073 ir.sp - M073 IR

F-NMR L059

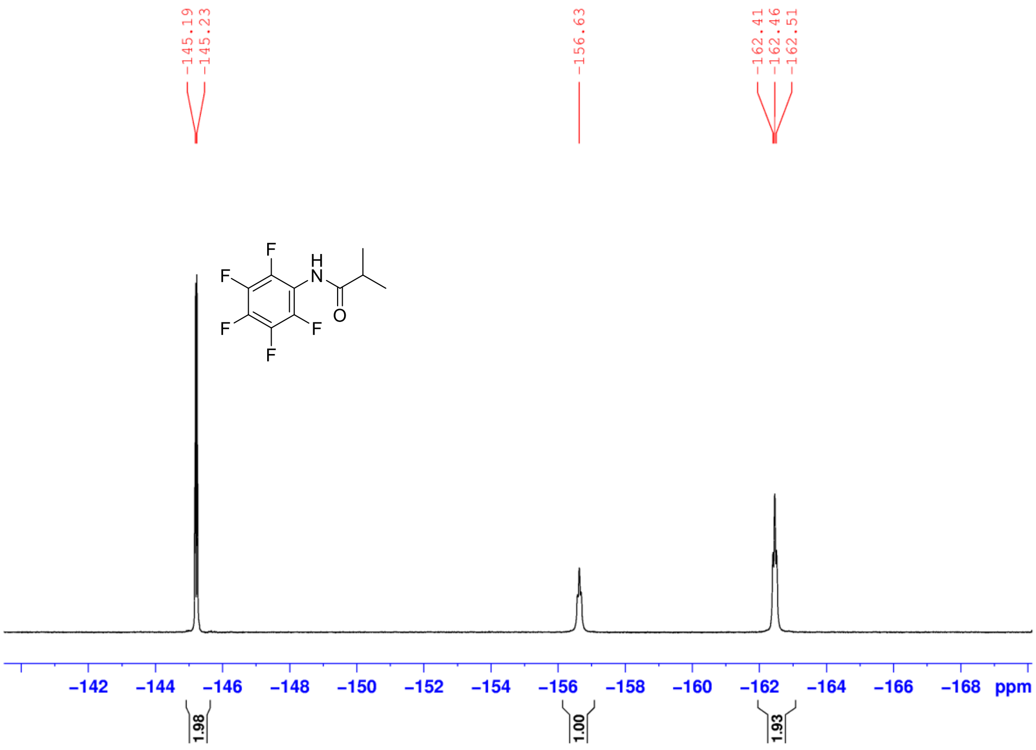


C-NMR L059

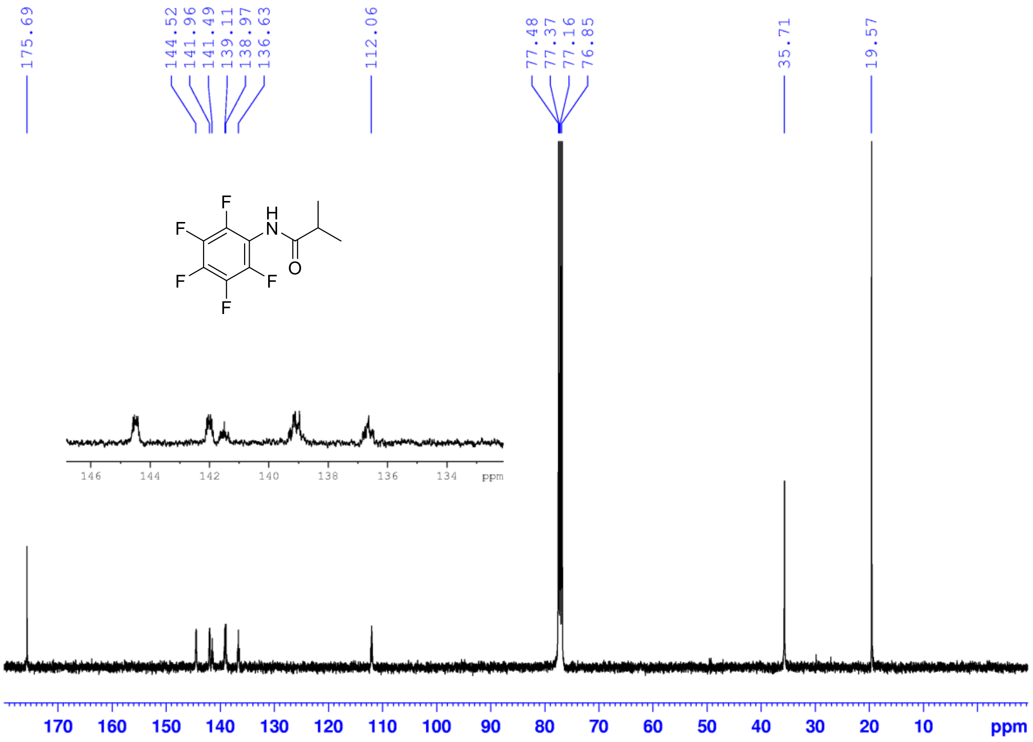

H-NMR L059

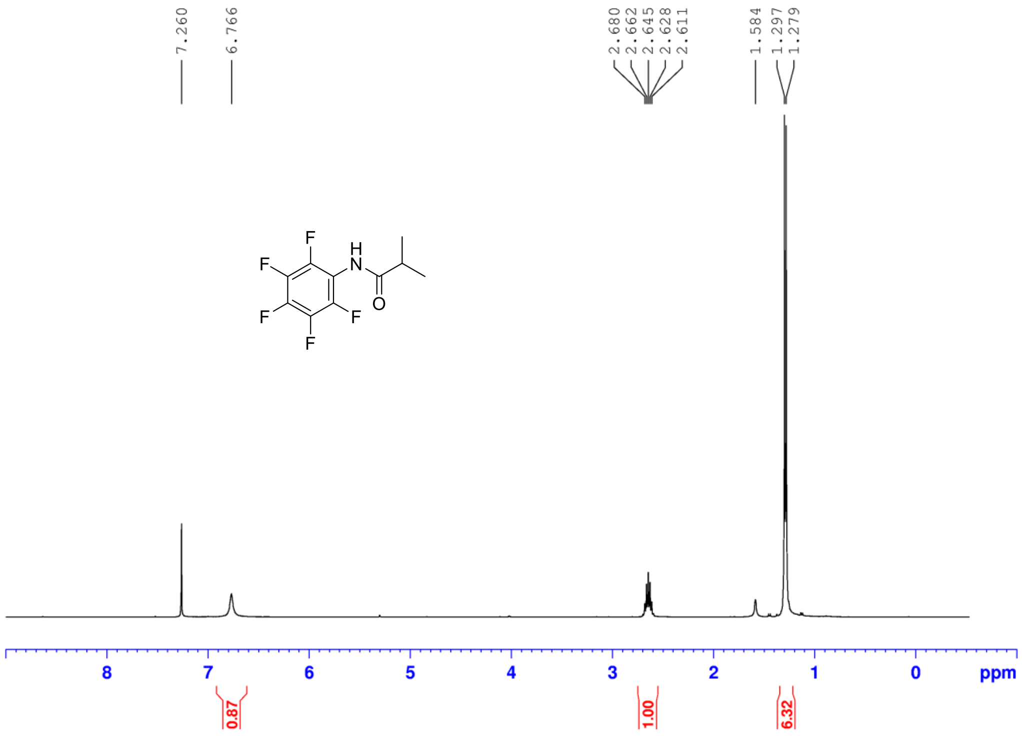
7.260
6.766
2.680
2.662
2.645
2.628
2.611
1.584
1.297
1.279
F
F
F
F
F
H
N
O
8
7
6
5
4
3
2
1
0
ppm
0.87
1.00
6.32

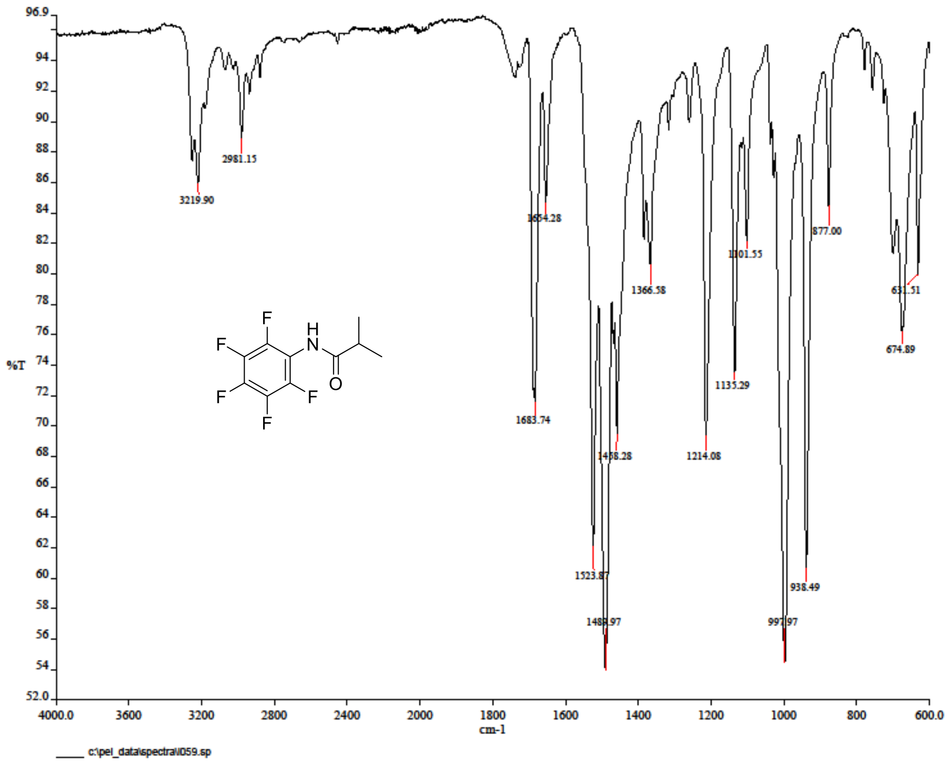
%T
cm-1
3219.90
2981.15
1654.28
1683.74
1523.87
1488.97
1458.28
1366.58
1214.08
1135.29
1101.55
997.97
938.49
877.00
674.89
631.51
c:\pel_data\spectra\l059.sp

FNMR L047 CDCl3 376MHz

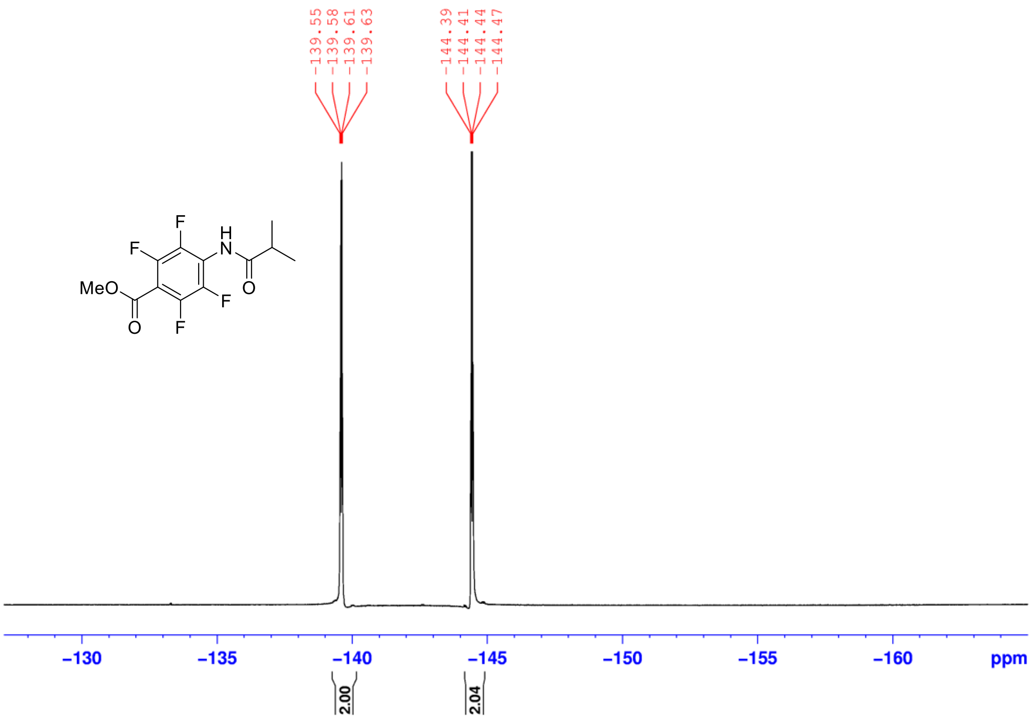


C13 L047 CDCl3 100MHz

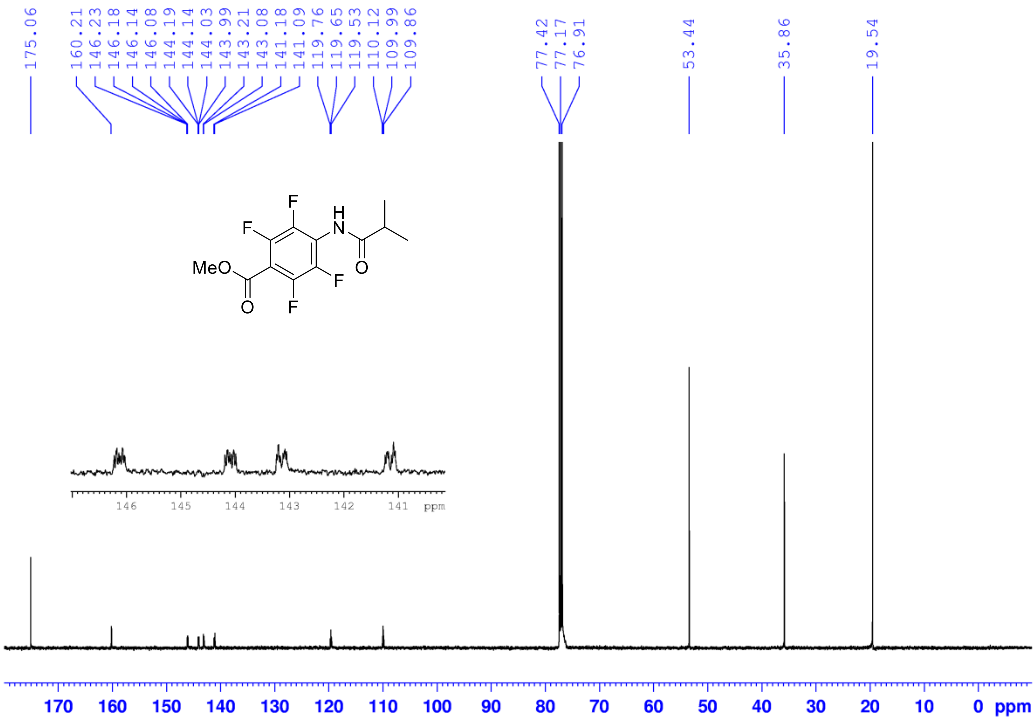

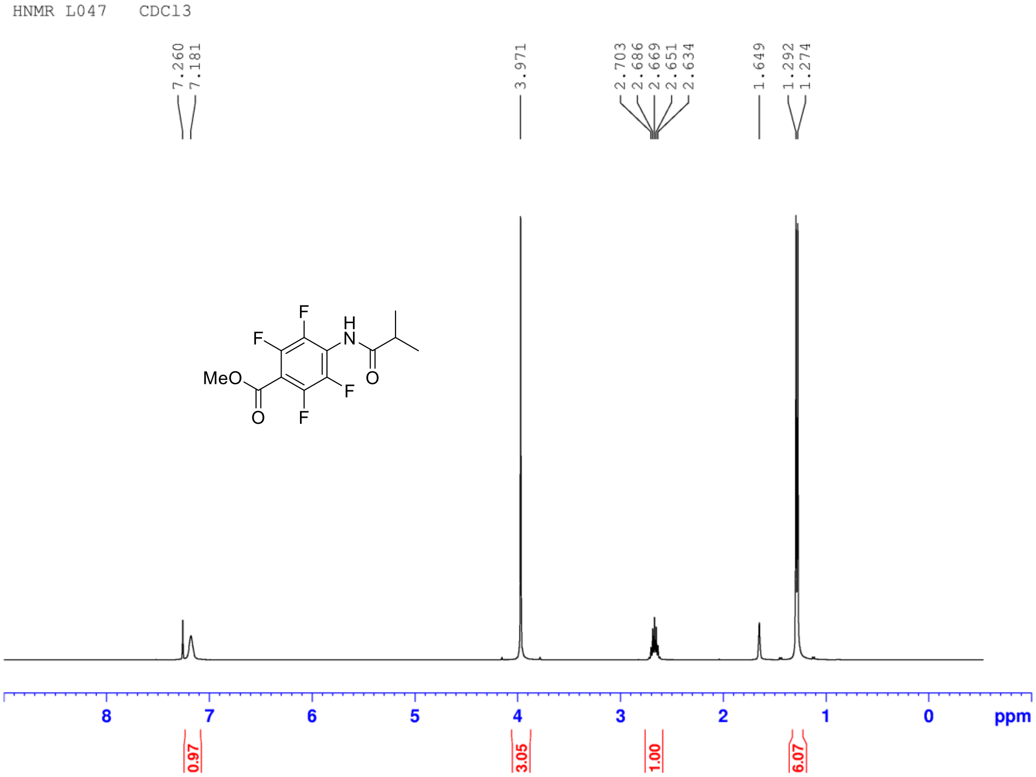
HNMR L047 CDCl3
7.260
7.181
3.971
2.703
2.686
2.669
2.651
2.634
1.649
1.292
1.274
F
F
H
N
MeO
O
F
O
F
8 7 6 5 4 3 2 1 0 ppm
0.97
3.05
1.00
6.07

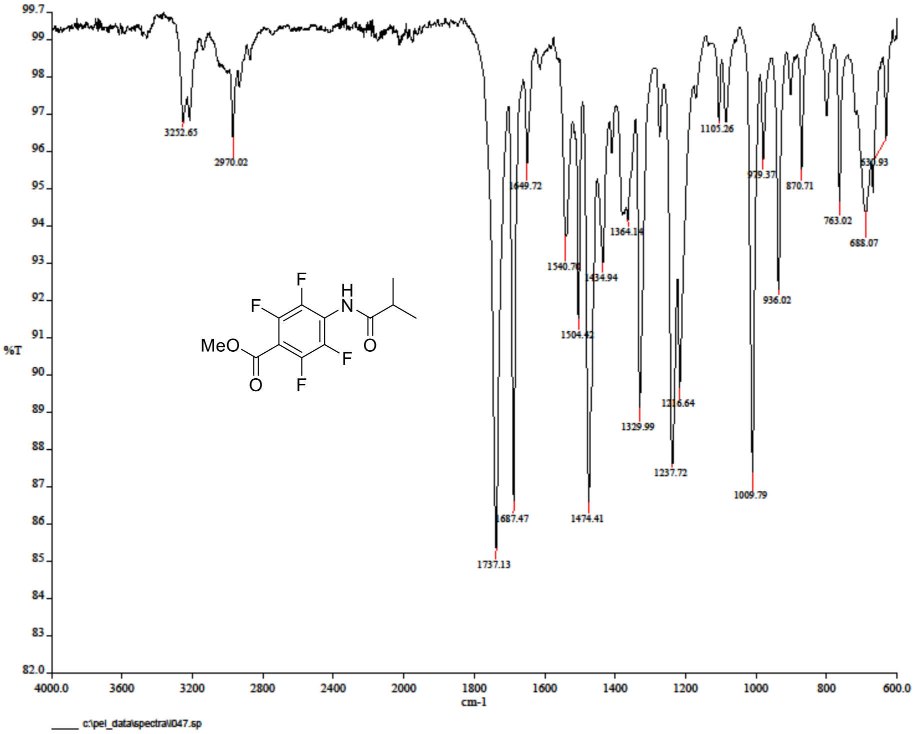
99.7
%T
82.0
4000.0 3600 3200 2800 2400 2000 1800 1600 1400 1200 1000 800 600.0
cm-1
3252.65
2970.02
1649.72
1540.76
1504.42
1434.94
1364.14
1329.99
1216.64
1237.72
1105.26
1009.79
979.37
936.02
870.71
763.02
688.07
1737.13
1687.47
1474.41
MeO
F
F
H
N
O
F
O
F
c:\pel_data\spectra\l047.sp

F-NMR   L057, without phase procession

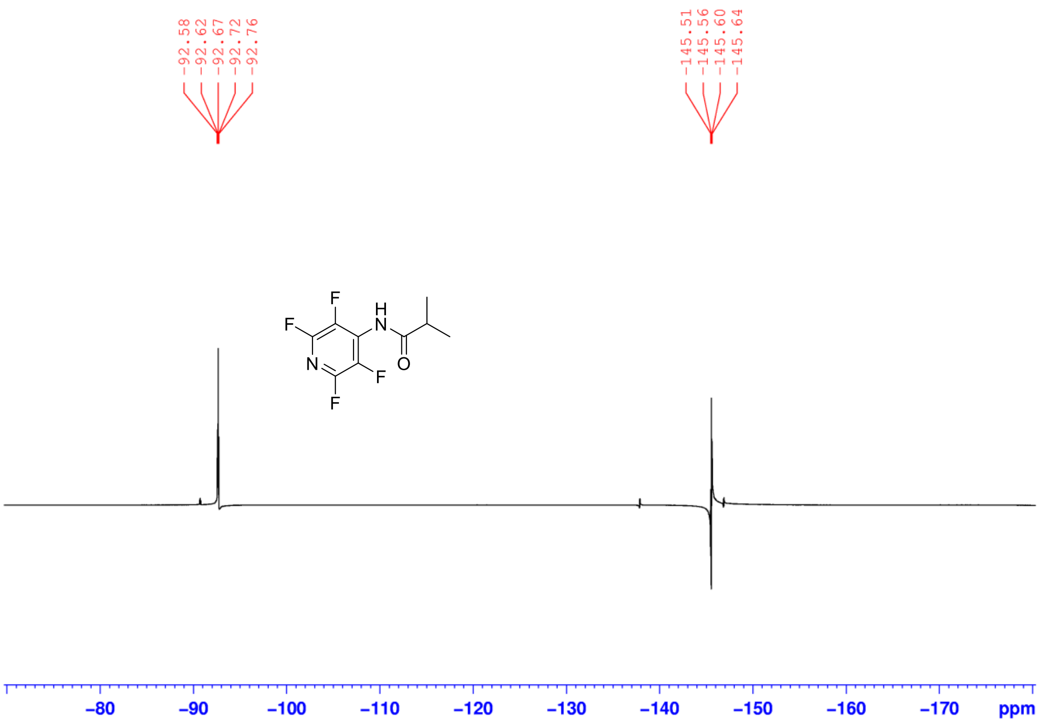


C-NMR   L057

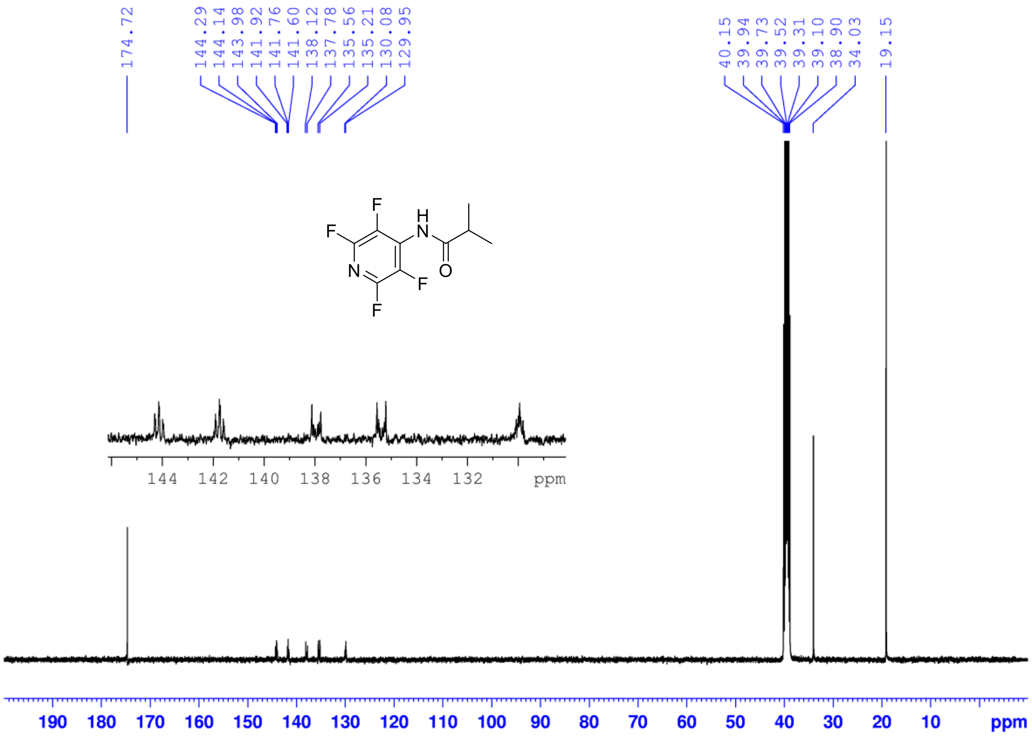

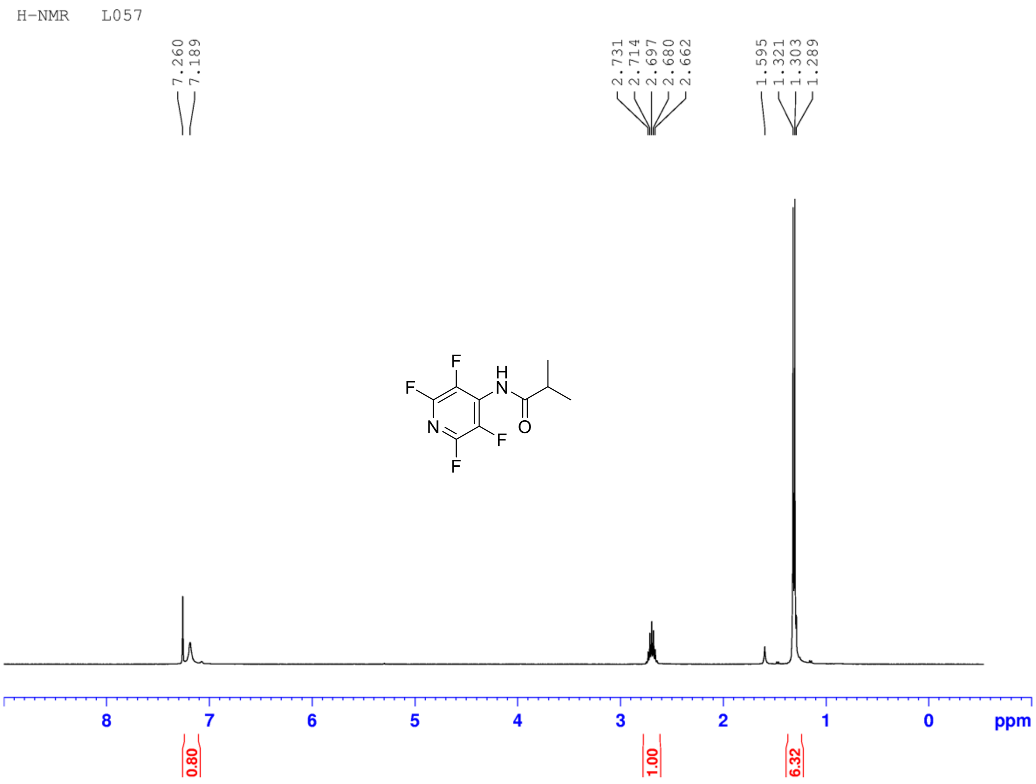
H-NMR L057
7.260
7.189
2.731
2.714
2.697
2.680
2.662
1.595
1.321
1.303
1.289
8
7
6
5
4
3
2
1
0
ppm
0.80
1.00
6.32
F
F
N
F
F
H
N
O

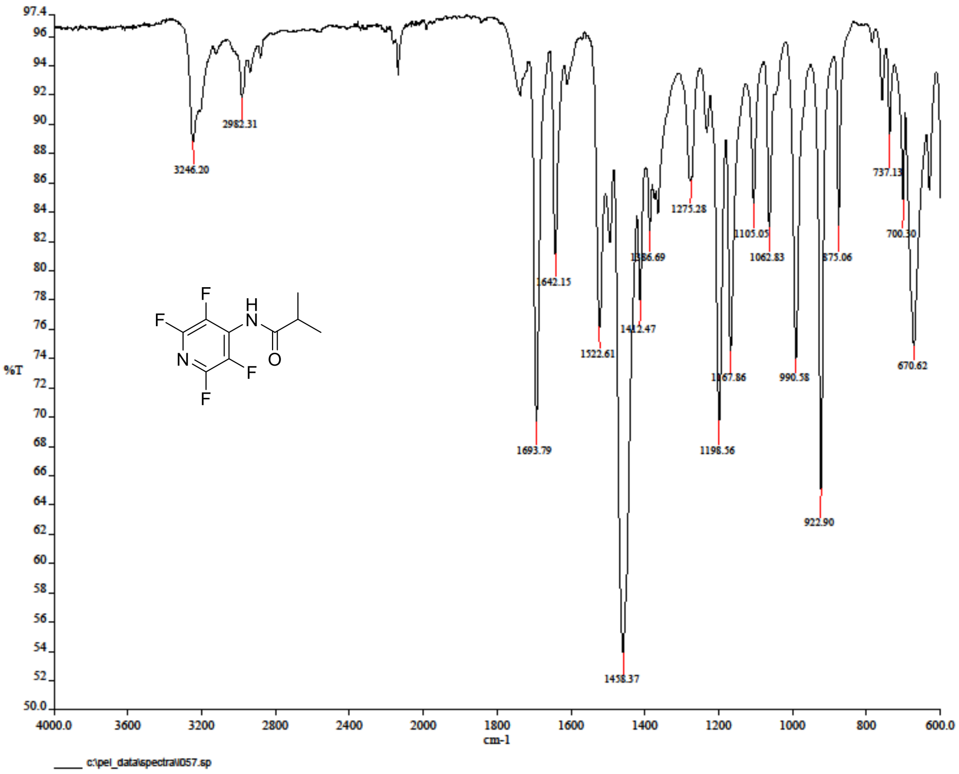
%T
cm-1
3246.20
2982.31
1693.79
1642.15
1522.61
1458.37
1412.47
1386.69
1275.28
1198.56
1167.86
1105.05
1062.83
990.58
922.90
875.06
737.13
700.30
670.62
c:\pel_data\spectra\l057.sp

F-NMR L058

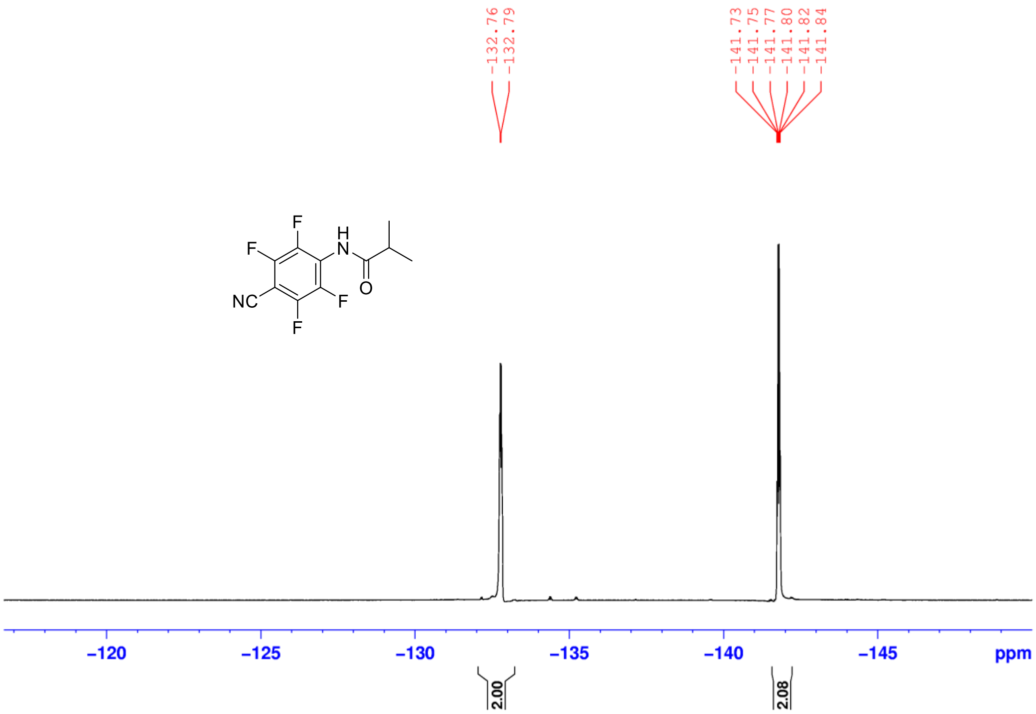


C-NMR L058

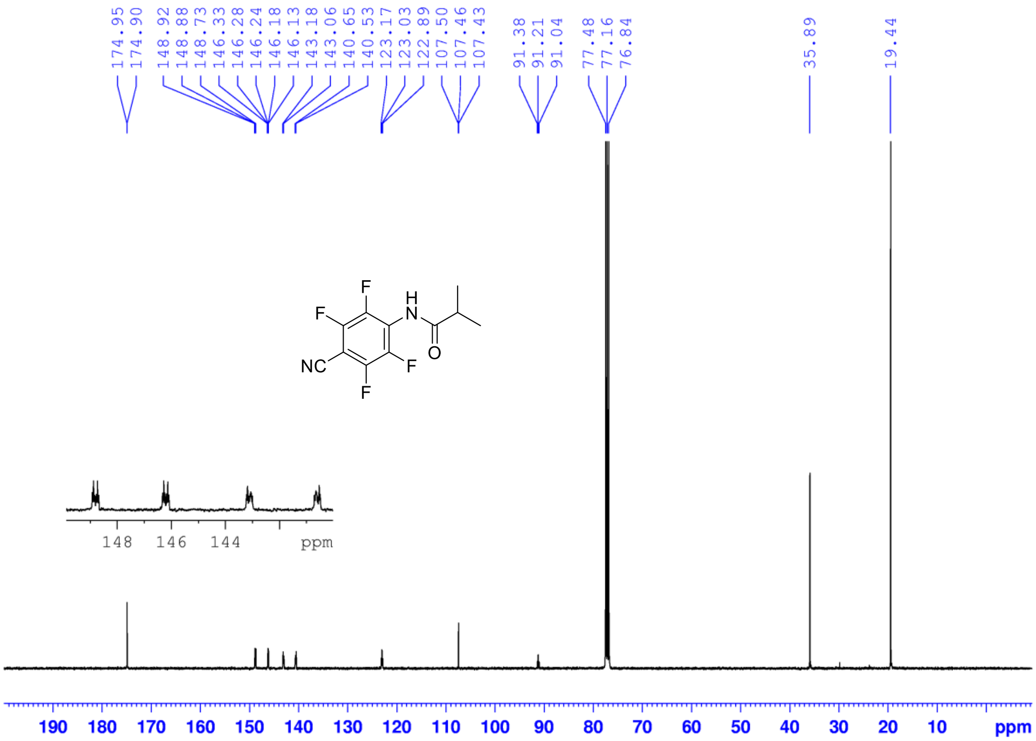

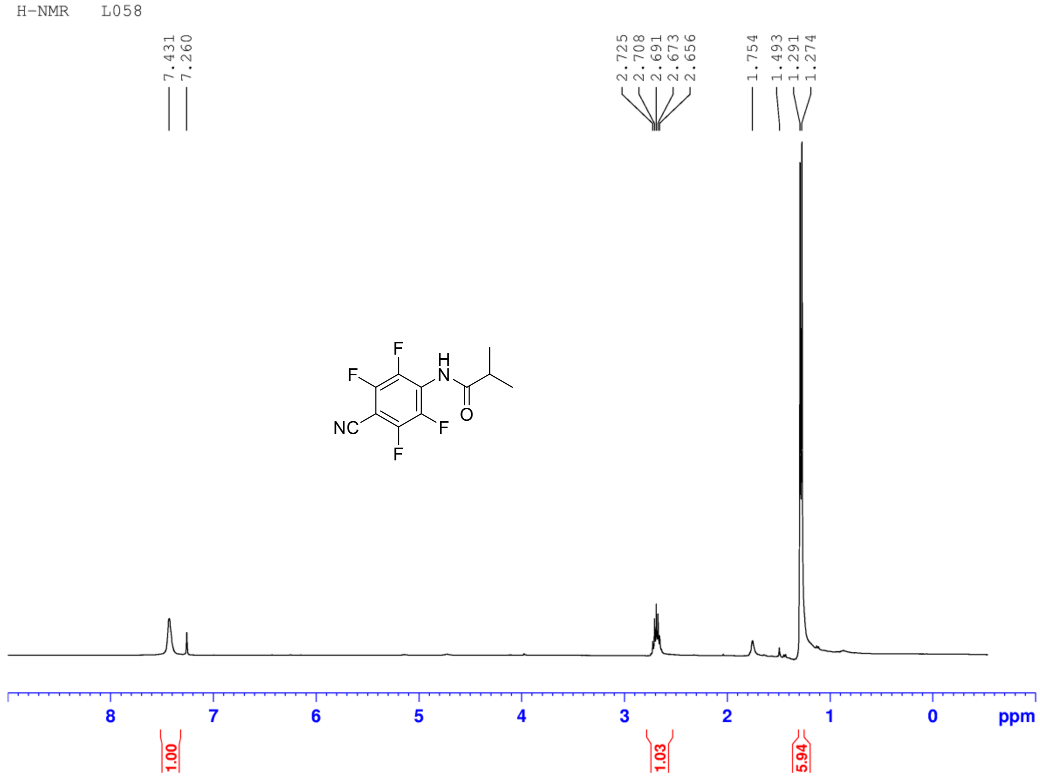
H-NMR L058
7.431
7.260
2.725
2.708
2.691
2.673
2.656
1.754
1.493
1.291
1.274
F
F
H
N
O
NC
F
F
8
7
6
5
4
3
2
1
0
ppm
1.00
1.03
5.94

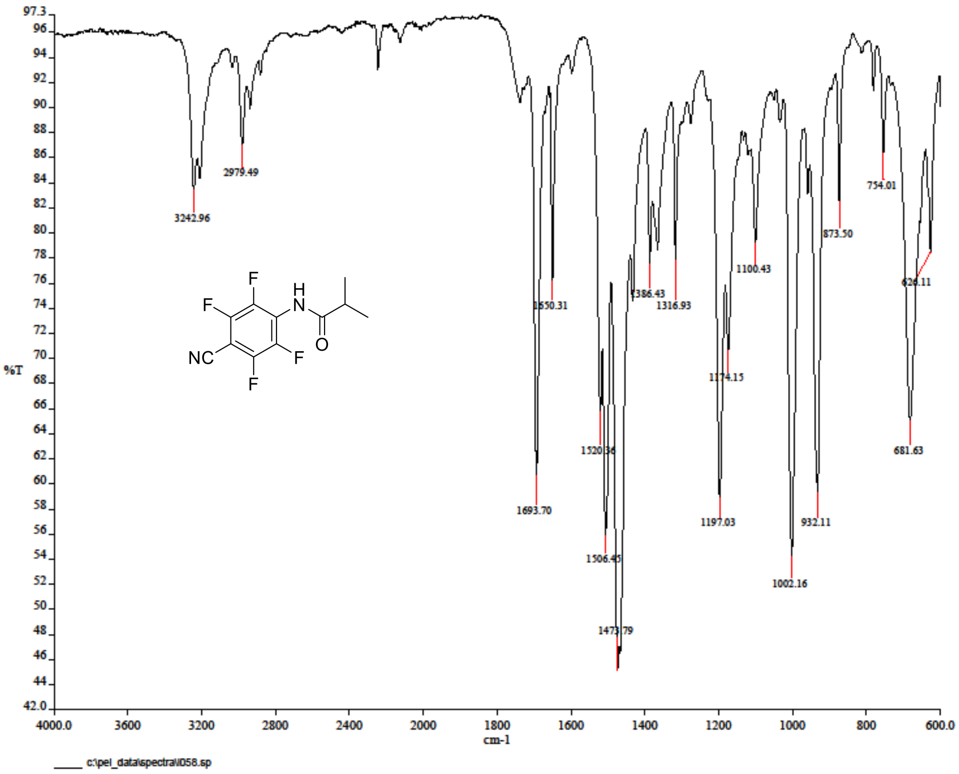
%T
cm-1
3242.96
2979.49
1693.70
1650.31
1520.36
1506.45
1473.79
1386.43
1316.93
1197.03
1174.15
1100.43
1002.16
932.11
873.50
754.01
681.63
626.11
c:\pel_data\spectra\l058.sp

FNMR L082

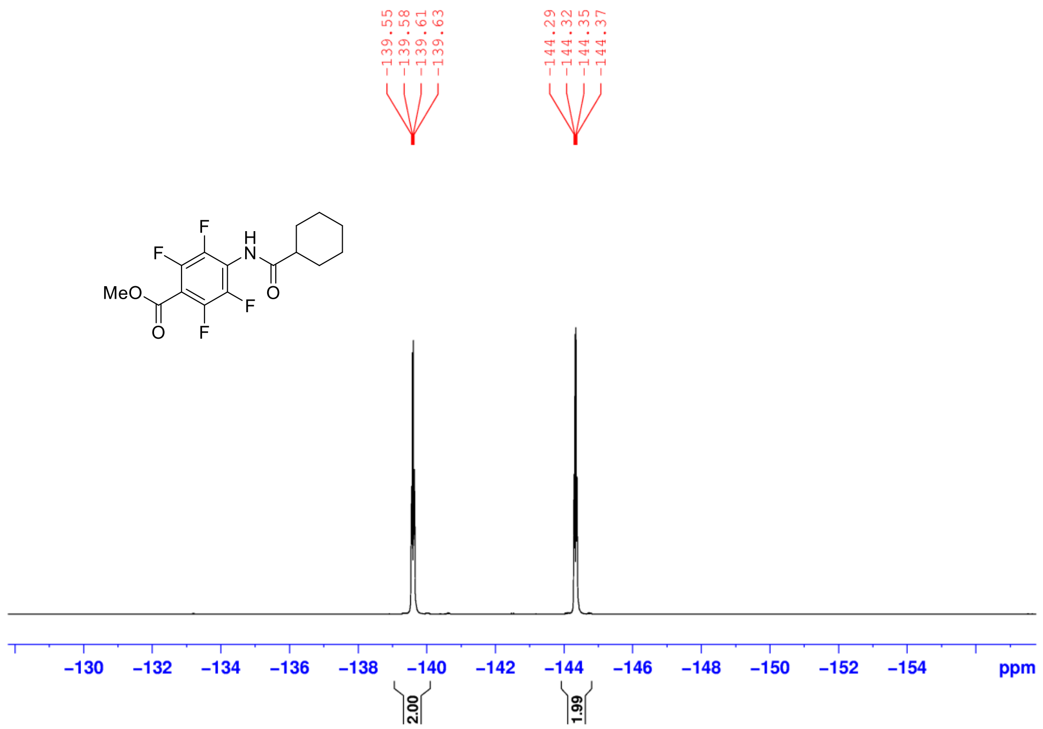


CNMR L081

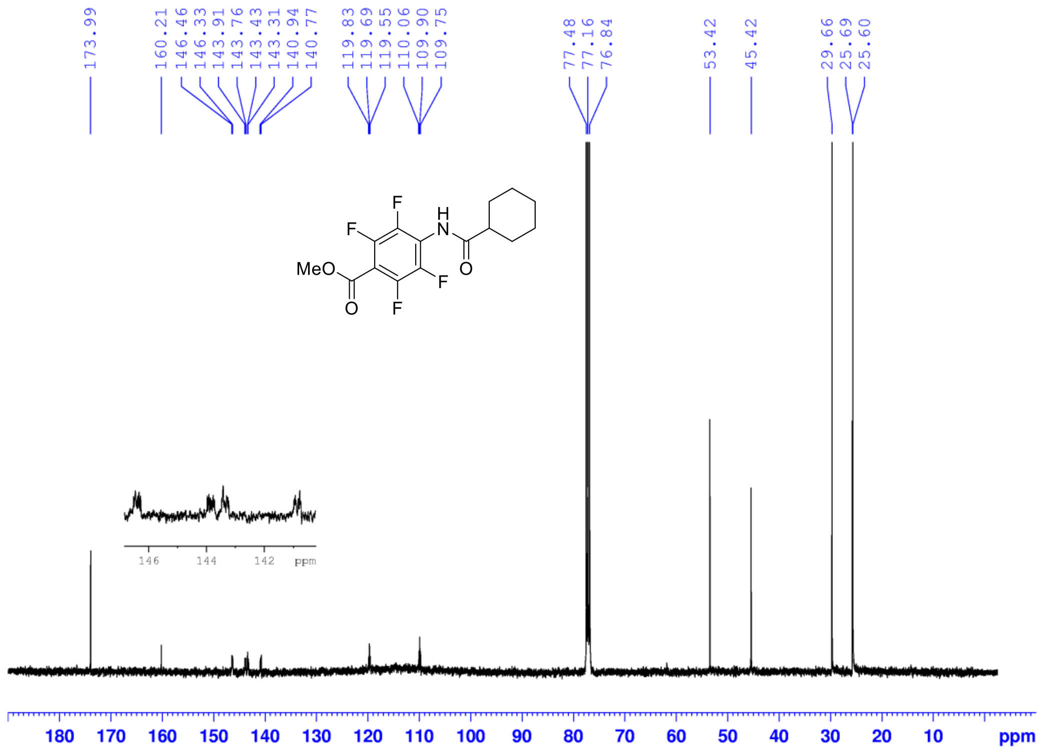

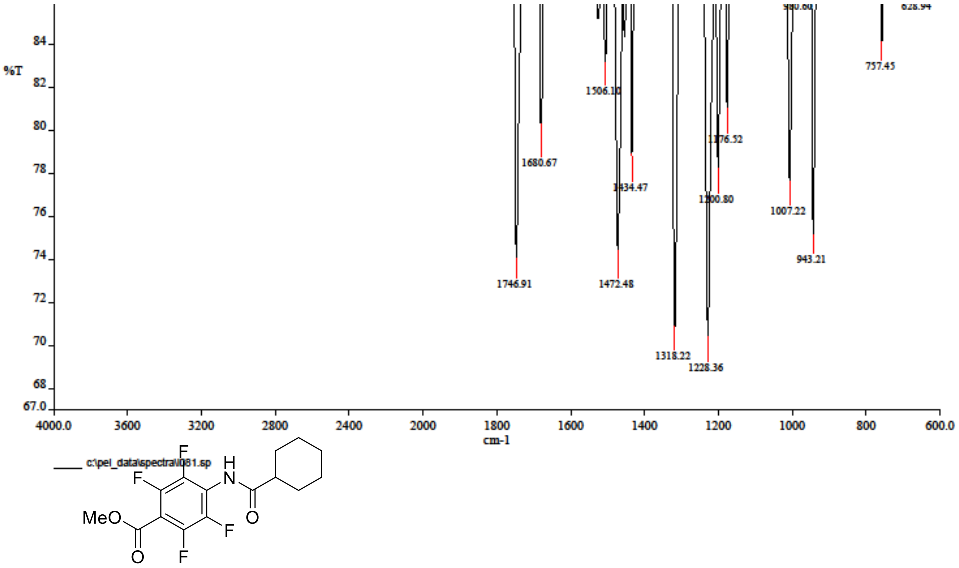
%T
1746.91
1680.67
1506.10
1472.48
1434.47
1318.22
1228.36
1200.80
1176.52
1007.22
943.21
757.45
cm-1
c:\pel_data\spectra\l081.sp
MeO
F
H
N
O

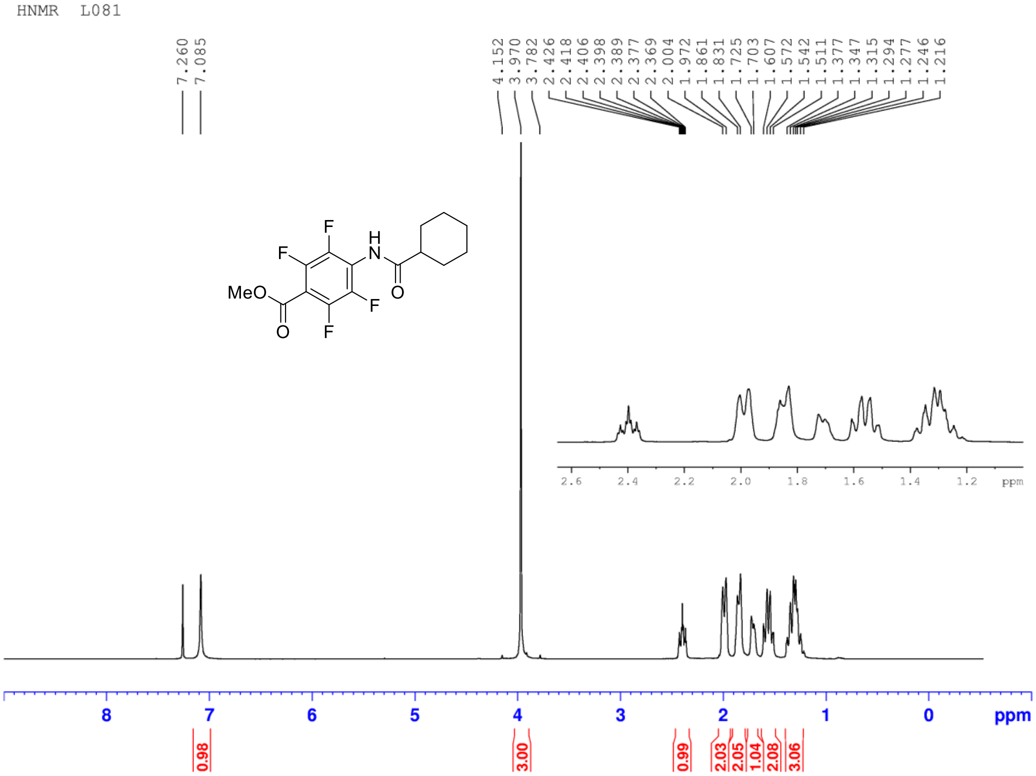
HNMR L081
7.260
7.085
4.152
3.970
3.782
2.426
2.418
2.406
2.398
2.389
2.377
2.369
2.004
1.972
1.861
1.831
1.725
1.703
1.607
1.572
1.542
1.511
1.377
1.347
1.315
1.294
1.277
1.246
1.216
ppm
0.98
3.00
0.99
2.03
2.05
1.04
2.08
3.06
MeO
F
H
N
O

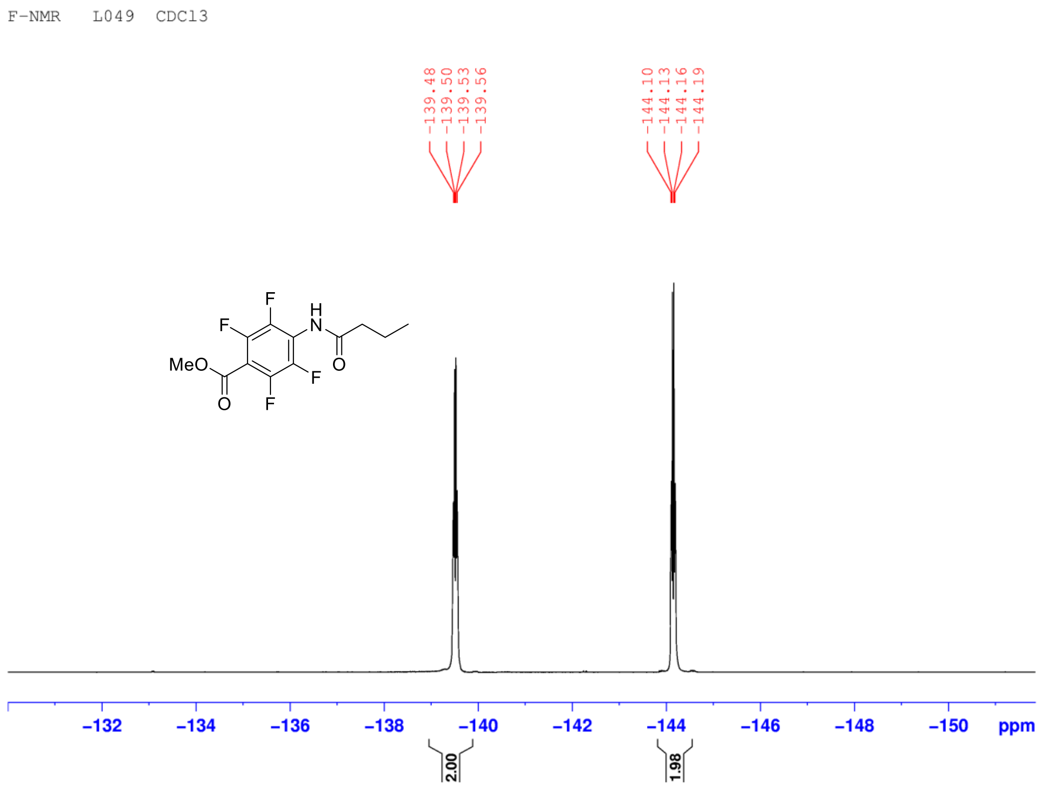

F-NMR L049 CDCl3
-139.48
-139.50
-139.53
-139.56
-144.10
-144.13
-144.16
-144.19
F
F
H
N
MeO
O
O
F
F
-132
-134
-136
-138
-140
-142
-144
-146
-148
-150
ppm
2.00
1.98


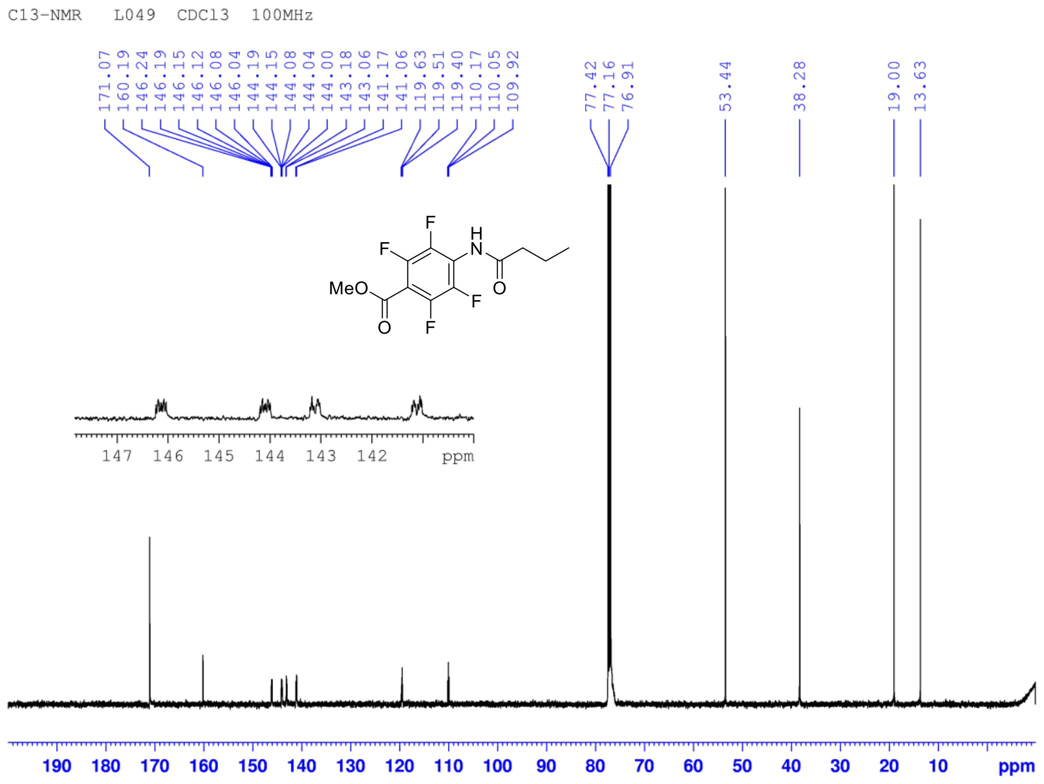

C13-NMR L049 CDCl3 100MHz
171.07
160.19
146.24
146.19
146.15
146.12
146.08
146.04
144.19
144.15
144.08
144.04
144.00
143.18
143.06
141.17
141.06
119.63
119.51
119.40
110.17
110.05
109.92
77.42
77.16
76.91
53.44
38.28
19.00
13.63
F
F
H
N
MeO
O
O
F
F
147
146
145
144
143
142
ppm
190
180
170
160
150
140
130
120
110
100
90
80
70
60
50
40
30
20
10
ppm

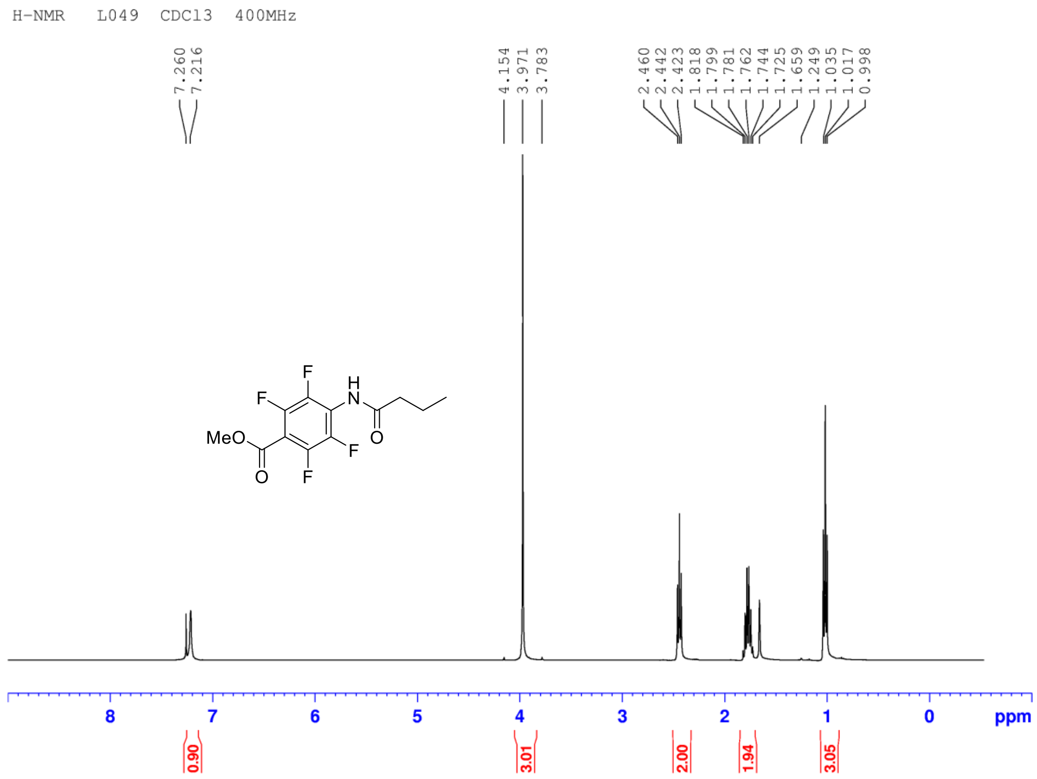
H-NMR L049 CDCl3 400MHz
7.260
7.216
4.154
3.971
3.783
2.460
2.442
2.423
1.818
1.799
1.781
1.762
1.744
1.725
1.659
1.249
1.035
1.017
0.998
F
F
H
N
MeO
O
F
O
F
8
7
6
5
4
3
2
1
0
ppm
0.90
3.01
2.00
1.94
3.05

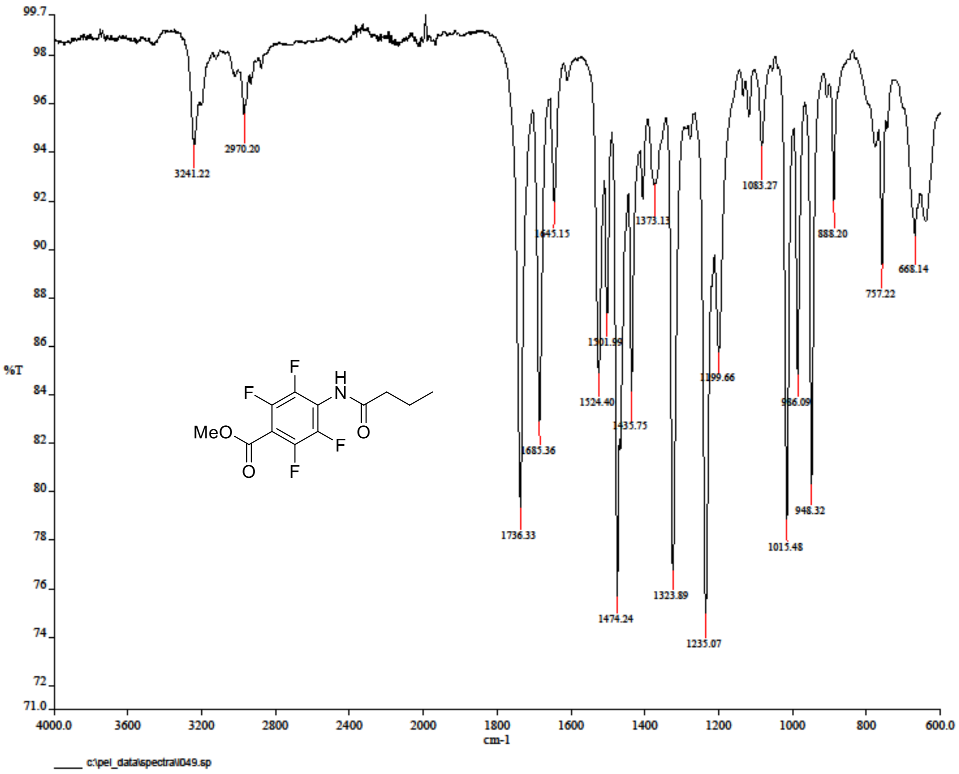
99.7
98
96
94
92
90
88
86
%T
84
82
80
78
76
74
72
71.0
4000.0
3600
3200
2800
2400
2000
1800
cm-1
1600
1400
1200
1000
800
600.0
3241.22
2970.20
1736.33
1685.36
1645.15
1524.40
1501.99
1474.24
1435.75
1373.13
1323.89
1235.07
1199.66
1083.27
1015.48
986.09
948.32
888.20
757.22
668.14
F
F
H
N
MeO
O
F
O
F
c:\pel_data\spectra\l049.sp

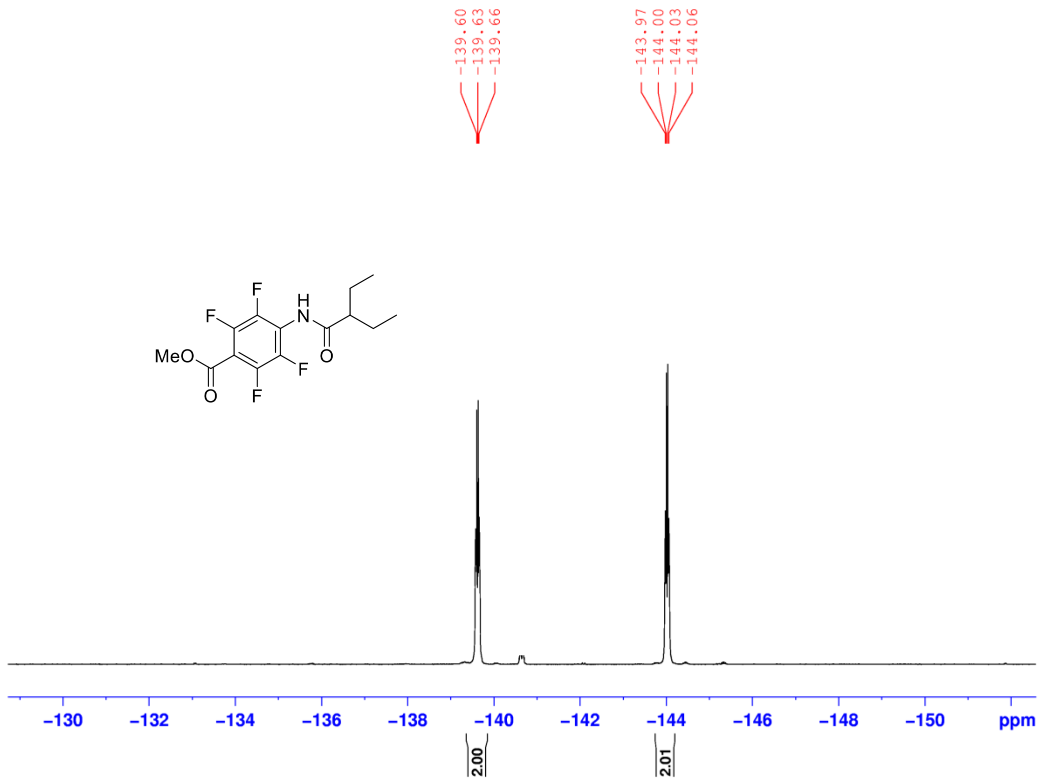
-139.60
-139.63
-139.66
-143.97
-144.00
-144.03
-144.06
F
F
H
N
MeO
F
O
O
F
-130 -132 -134 -136 -138 -140 -142 -144 -146 -148 -150 ppm
2.00
2.01

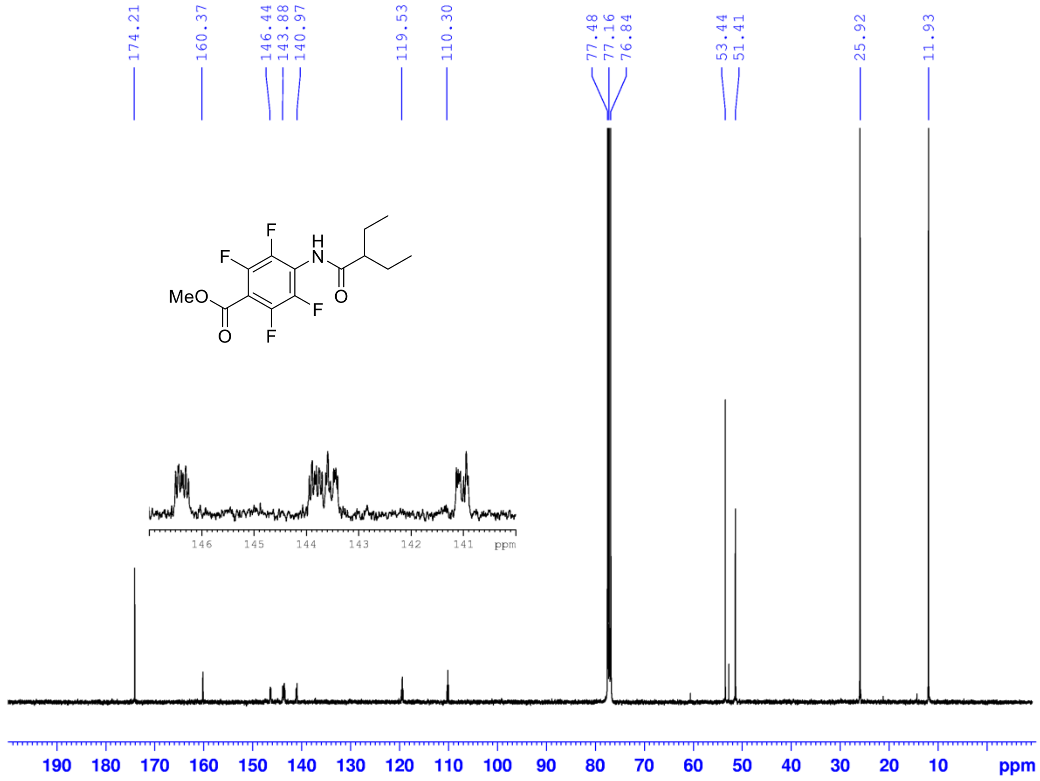
174.21
160.37
146.44
143.88
140.97
119.53
110.30
77.48
77.16
76.84
53.44
51.41
25.92
11.93
F
F
H
N
MeO
F
O
O
F
146 145 144 143 142 141 ppm
190 180 170 160 150 140 130 120 110 100 90 80 70 60 50 40 30 20 10 ppm

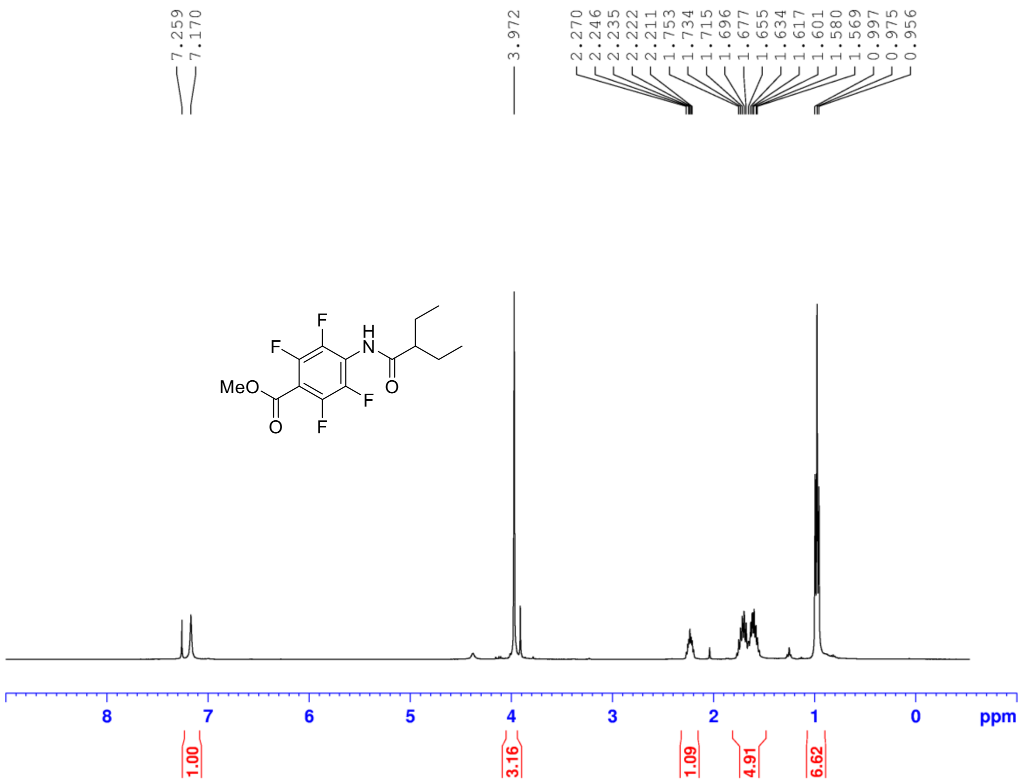

7.259
7.170
3.972
2.270
2.246
2.235
2.222
2.211
1.753
1.734
1.715
1.696
1.677
1.655
1.634
1.617
1.601
1.580
1.569
0.997
0.975
0.956
F
H
N
F
MeO
O
F
O
F
8
7
6
5
4
3
2
1
0
ppm
1.00
3.16
1.09
4.91
6.62

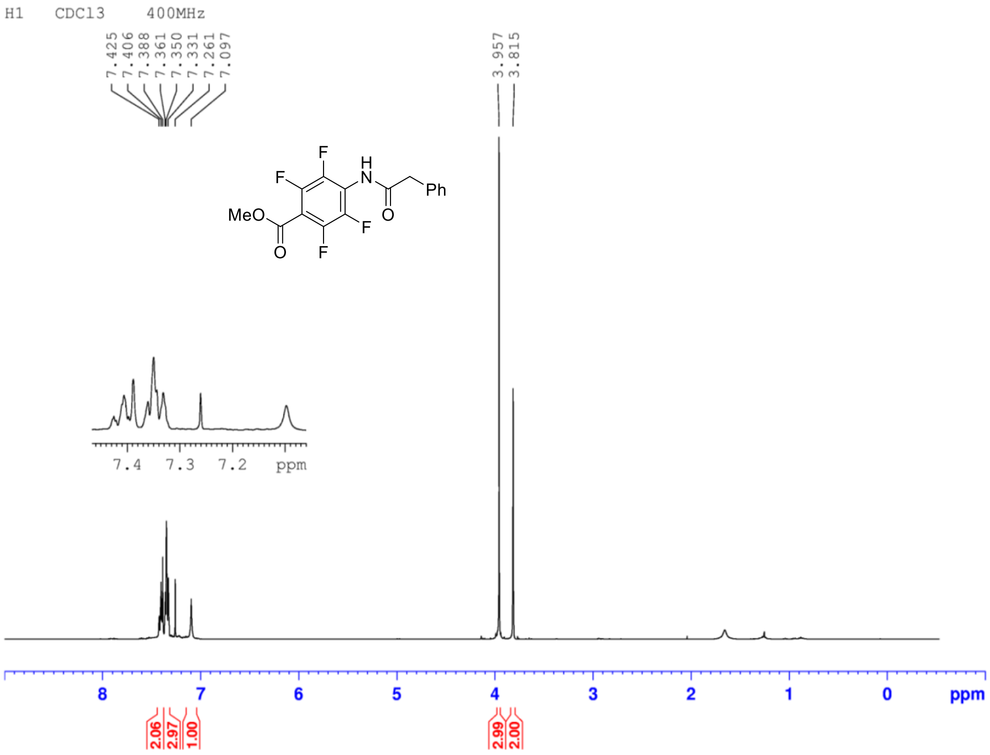
H1 CDCl3 400MHz
7.425
7.406
7.388
7.361
7.350
7.331
7.261
7.097
3.957
3.815
F
F
H
N
Ph
MeO
O
F
F
O
7.4 7.3 7.2 ppm
8 7 6 5 4 3 2 1 0 ppm
2.06
2.97
1.00
2.99
2.00

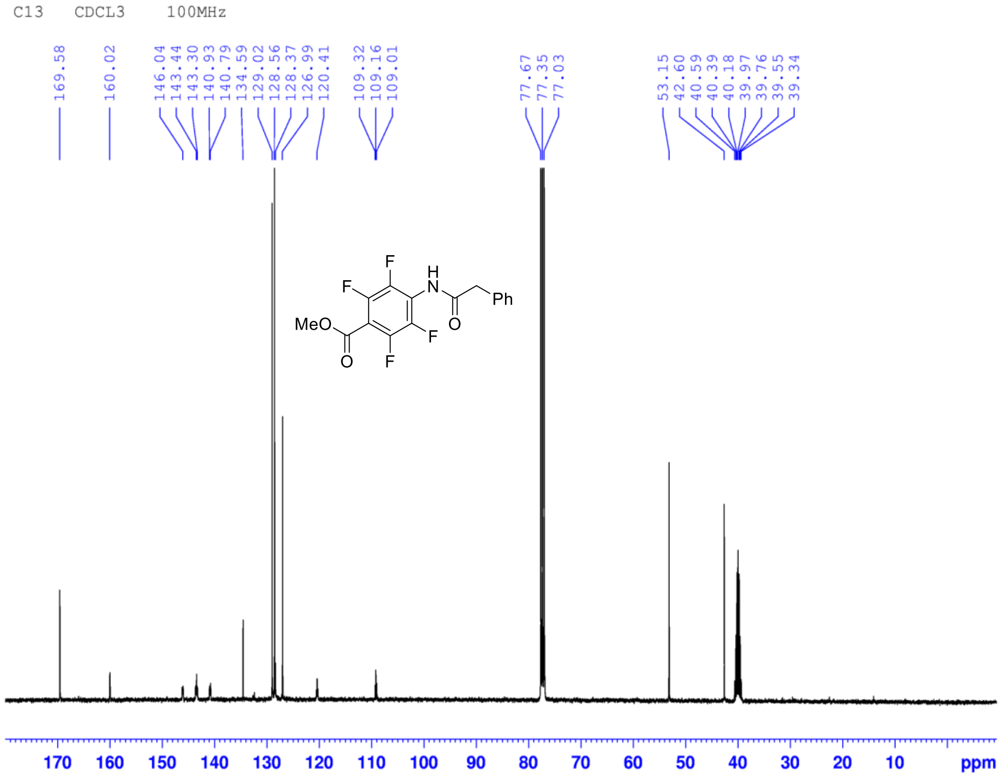
C13 CDCL3 100MHz
169.58
160.02
146.04
143.44
143.30
140.93
140.79
134.59
129.02
128.56
128.37
126.99
120.41
109.32
109.16
109.01
77.67
77.35
77.03
53.15
42.60
40.59
40.39
40.18
39.97
39.76
39.55
39.34
F
F
H
N
Ph
MeO
O
F
F
O
170 160 150 140 130 120 110 100 90 80 70 60 50 40 30 20 10 ppm

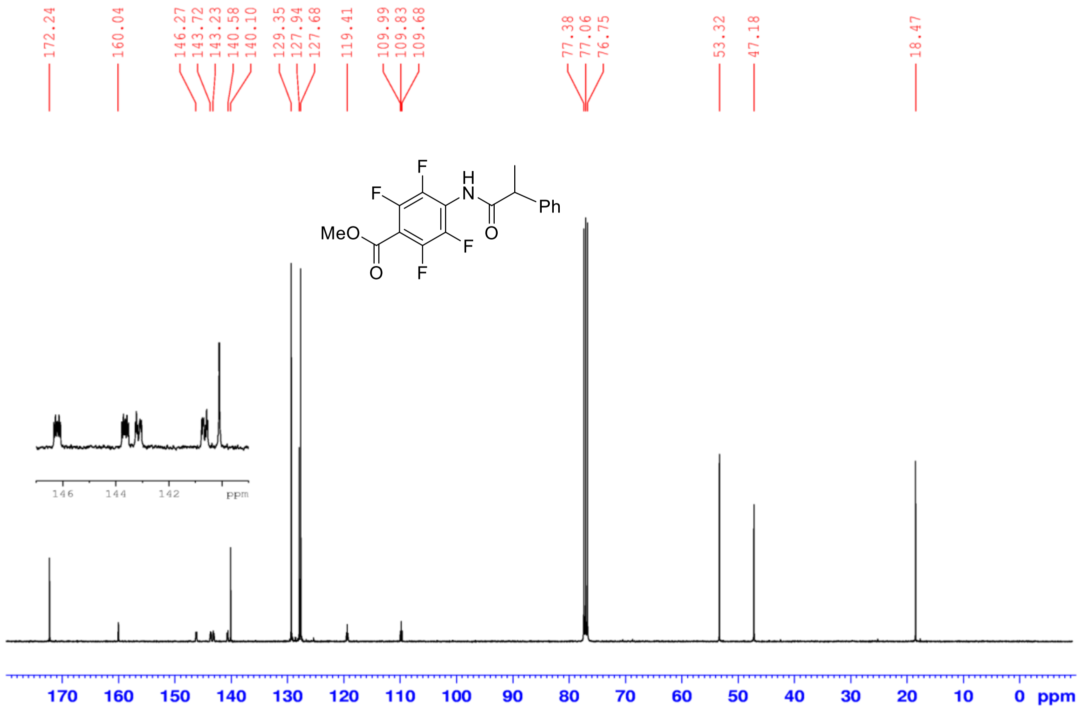
172.24
160.04
146.27
143.72
143.23
140.58
140.10
129.35
127.94
127.68
119.41
109.99
109.83
109.68
77.38
77.06
76.75
53.32
47.18
18.47
F
H
N
Ph
F
MeO
O
F
O
F
146
144
142
ppm
170
160
150
140
130
120
110
100
90
80
70
60
50
40
30
20
10
0
ppm

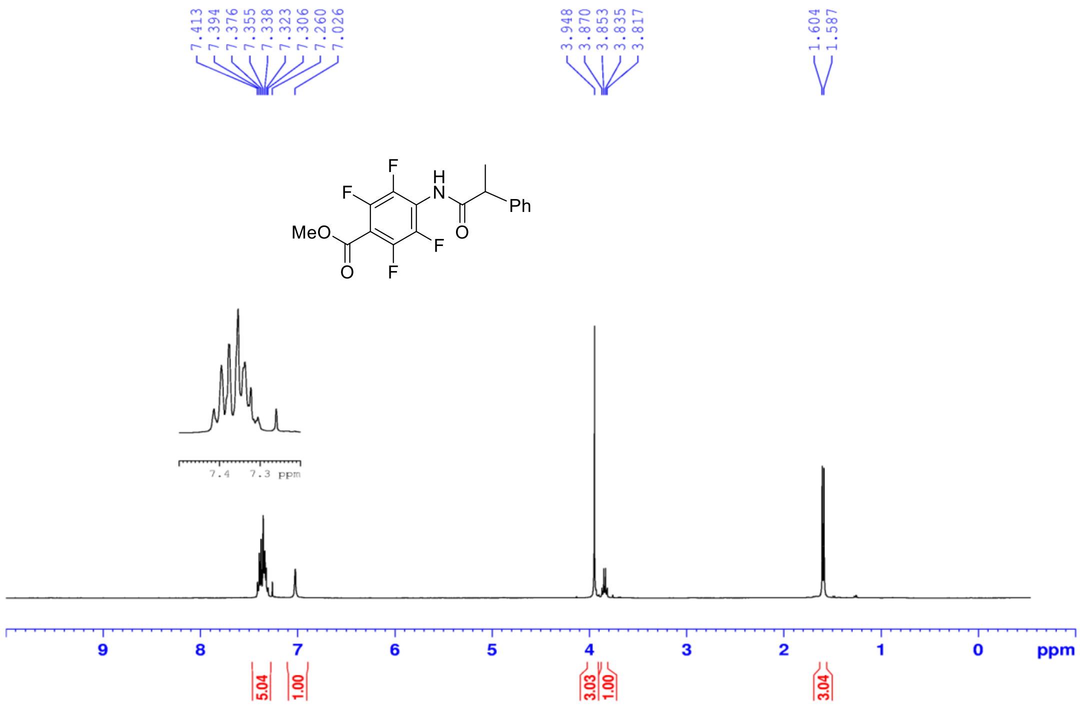
7.413
7.394
7.376
7.355
7.338
7.323
7.306
7.260
7.026
3.948
3.870
3.853
3.835
3.817
1.604
1.587
F
H
N
Ph
F
MeO
O
F
O
F
7.4
7.3
ppm
9
8
7
6
5
4
3
2
1
0
ppm
5.04
1.00
3.03
1.00
3.04

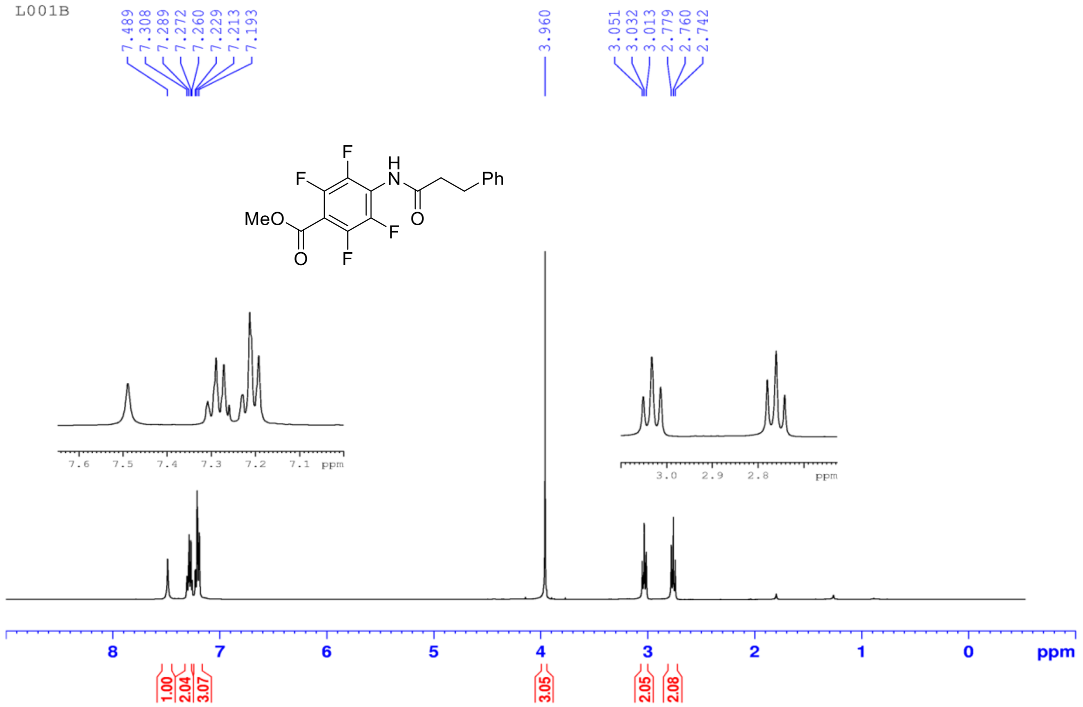
L001B
7.489
7.308
7.289
7.272
7.260
7.229
7.213
7.193
3.960
3.051
3.032
3.013
2.779
2.760
2.742
F
F
H
N
Ph
MeO
O
F
O
F
1.00
2.04
3.07
3.05
2.05
2.08
ppm

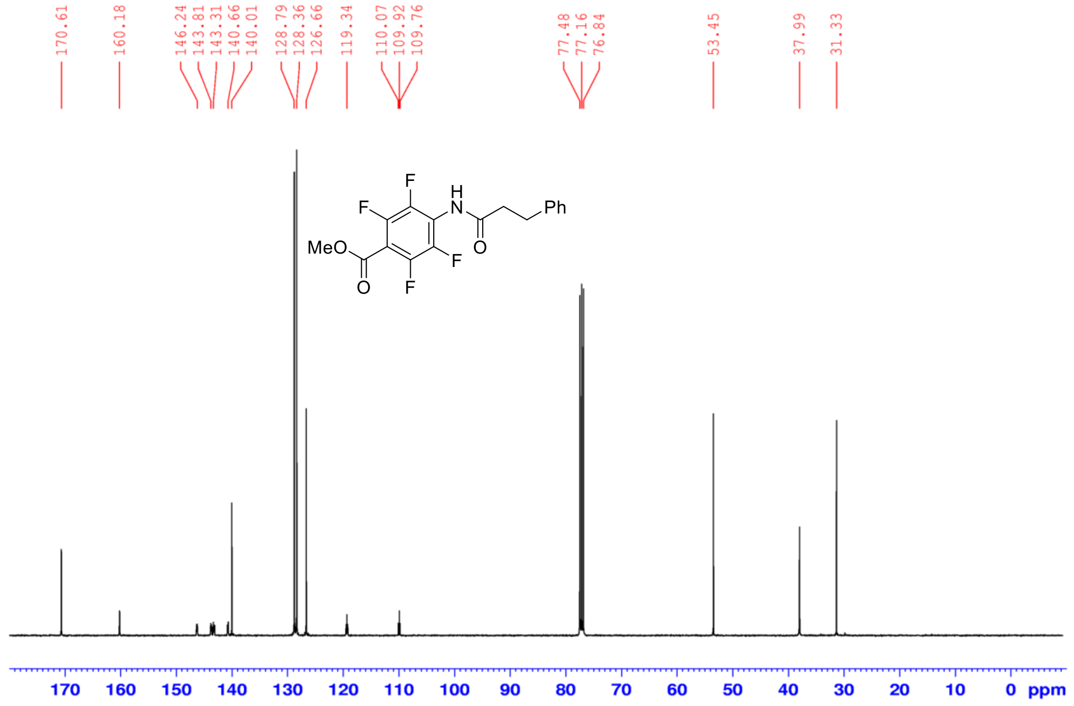
170.61
160.18
146.24
143.81
143.31
140.66
140.01
128.79
128.36
126.66
119.34
110.07
109.92
109.76
77.48
77.16
76.84
53.45
37.99
31.33
F
F
H
N
Ph
MeO
O
F
O
F
ppm

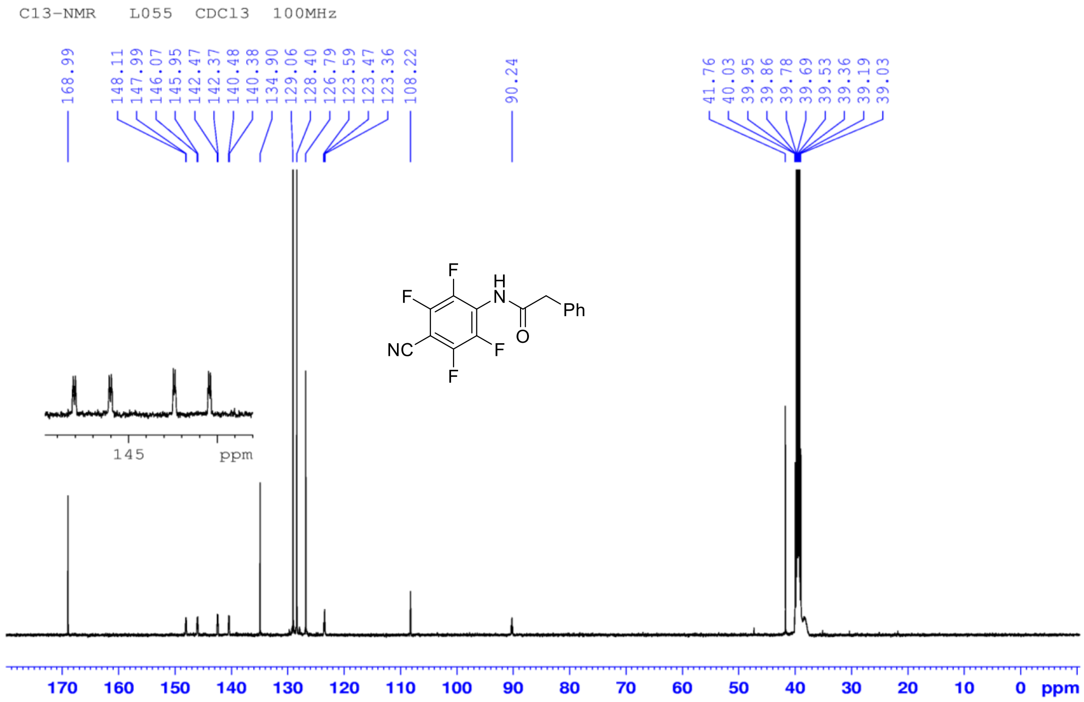
C13-NMR L055 CDCl3 100MHz
168.99
148.11
147.99
146.07
145.95
142.47
142.37
140.48
140.38
134.90
129.06
128.40
126.79
123.59
123.47
123.36
108.22
90.24
41.76
40.03
39.95
39.86
39.78
39.69
39.53
39.36
39.19
39.03
F
H
N
F
Ph
NC
F
O
F
145
ppm
170 160 150 140 130 120 110 100 90 80 70 60 50 40 30 20 10 0 ppm

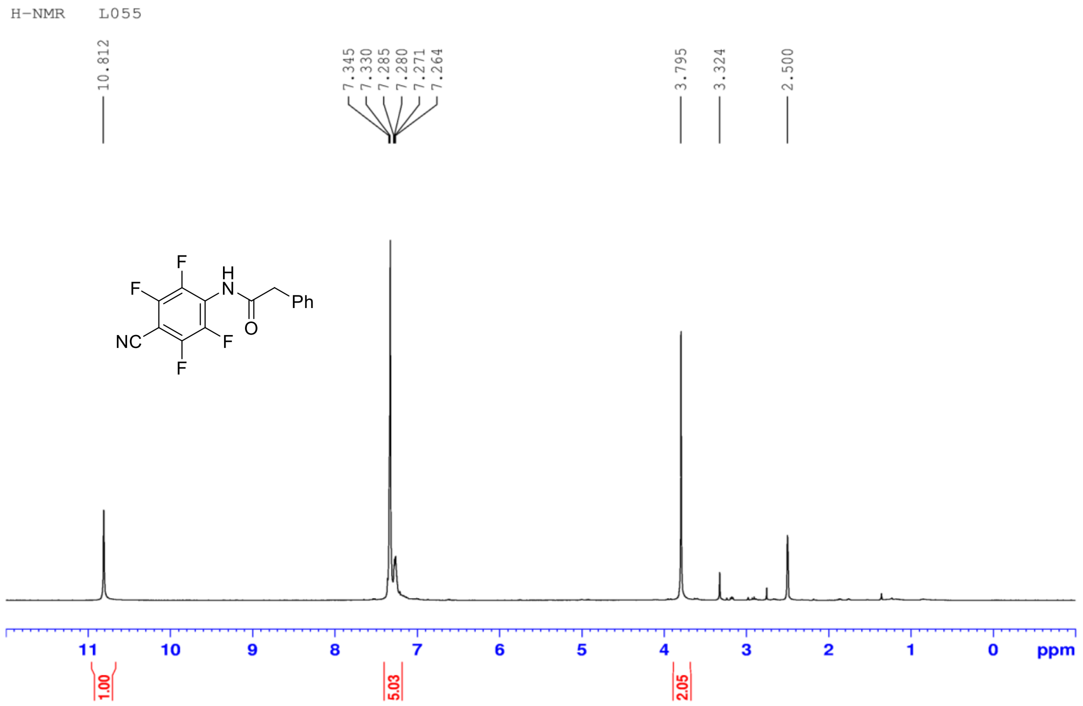
H-NMR L055
10.812
7.345
7.330
7.285
7.280
7.271
7.264
3.795
3.324
2.500
F
H
N
F
Ph
NC
F
O
F
11 10 9 8 7 6 5 4 3 2 1 0 ppm
1.00
5.03
2.05

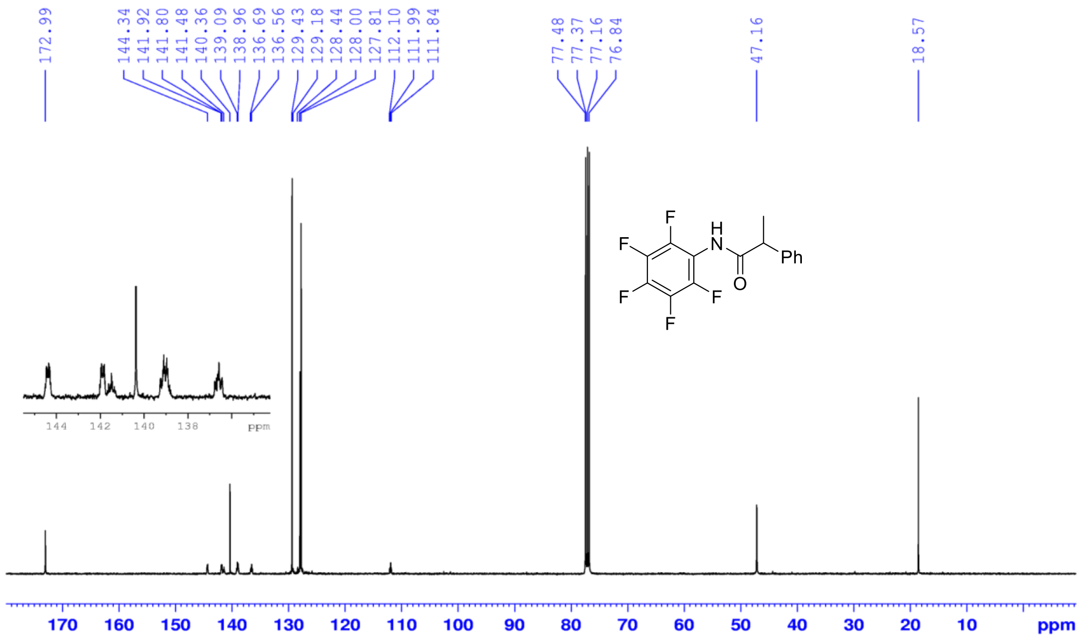
172.99
144.34
141.92
141.80
141.48
140.36
139.09
138.96
136.69
136.56
129.43
129.18
128.44
128.00
127.81
112.10
111.99
111.84
77.48
77.37
77.16
76.84
47.16
18.57
F
H
N
F
Ph
O
F
F
F
144
142
140
138
ppm
170
160
150
140
130
120
110
100
90
80
70
60
50
40
30
20
10
ppm

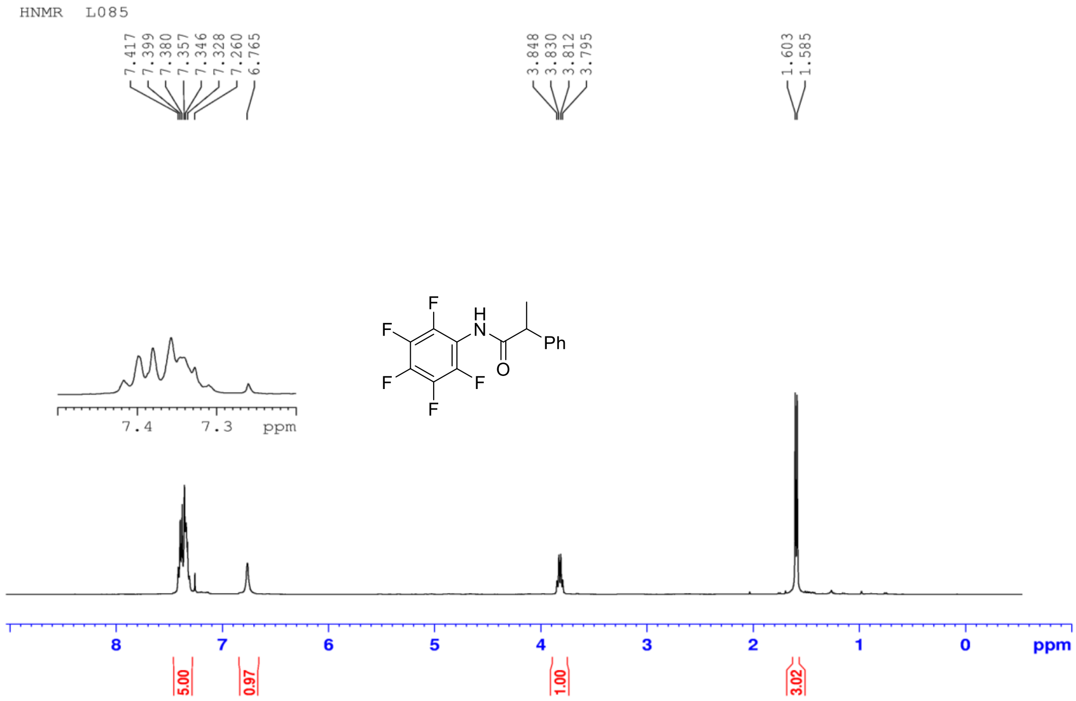
HNMR L085
7.417
7.399
7.380
7.357
7.346
7.328
7.260
6.765
3.848
3.830
3.812
3.795
1.603
1.585
F
H
N
F
Ph
O
F
F
F
7.4
7.3
ppm
8
7
6
5
4
3
2
1
0
ppm
5.00
0.97
1.00
3.02

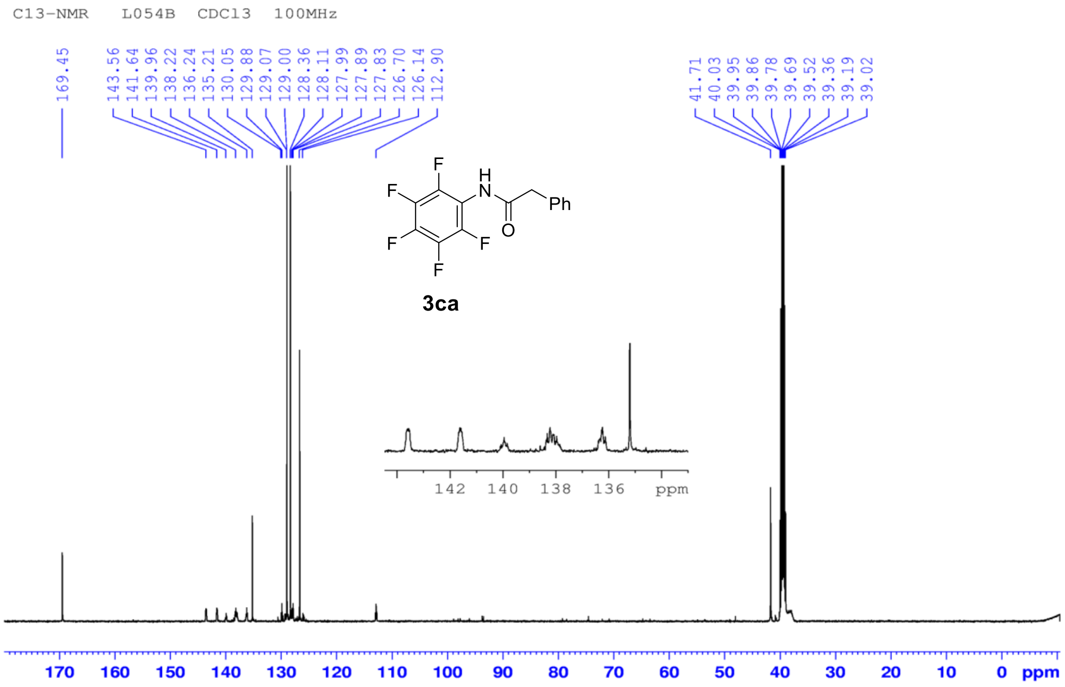
C13-NMR L054B CDCl3 100MHz
169.45
143.56
141.64
139.96
138.22
136.24
135.21
130.05
129.88
129.07
129.00
128.36
128.11
127.99
127.89
127.83
126.70
126.14
112.90
41.71
40.03
39.95
39.86
39.78
39.69
39.52
39.36
39.19
39.02
F
H
N
Ph
O
3ca
142
140
138
136
ppm
170
160
150
140
130
120
110
100
90
80
70
60
50
40
30
20
10
0
ppm

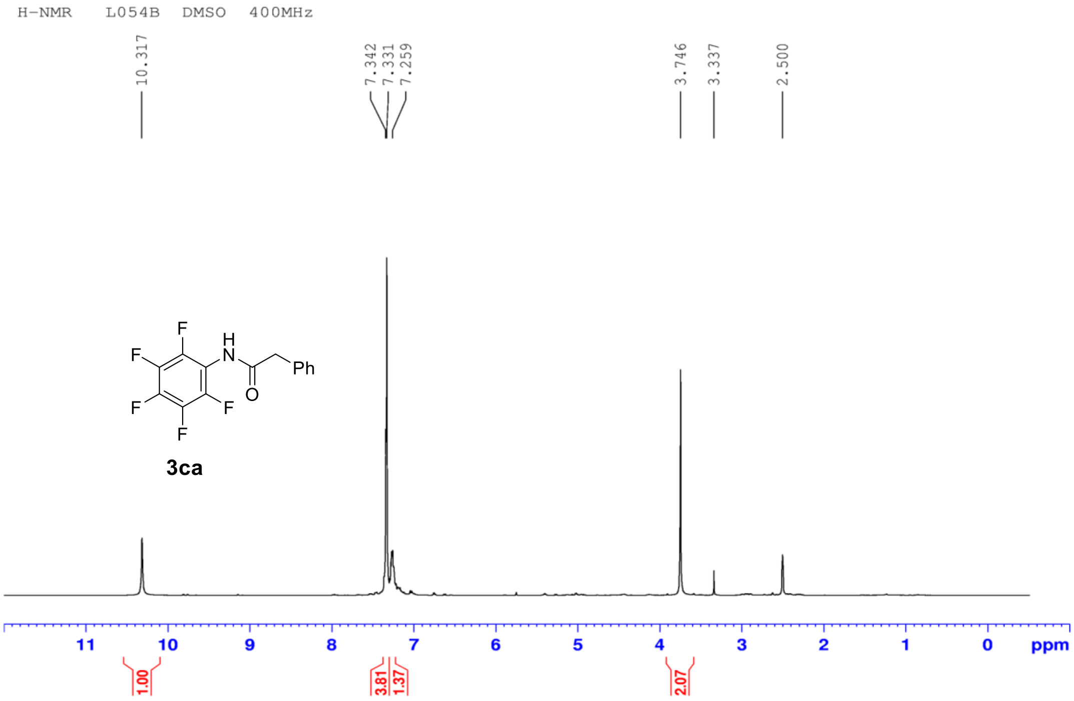
H-NMR L054B DMSO 400MHz
10.317
7.342
7.331
7.259
3.746
3.337
2.500
F
H
N
Ph
O
3ca
11
10
9
8
7
6
5
4
3
2
1
0
ppm
1.00
3.81
1.37
2.07

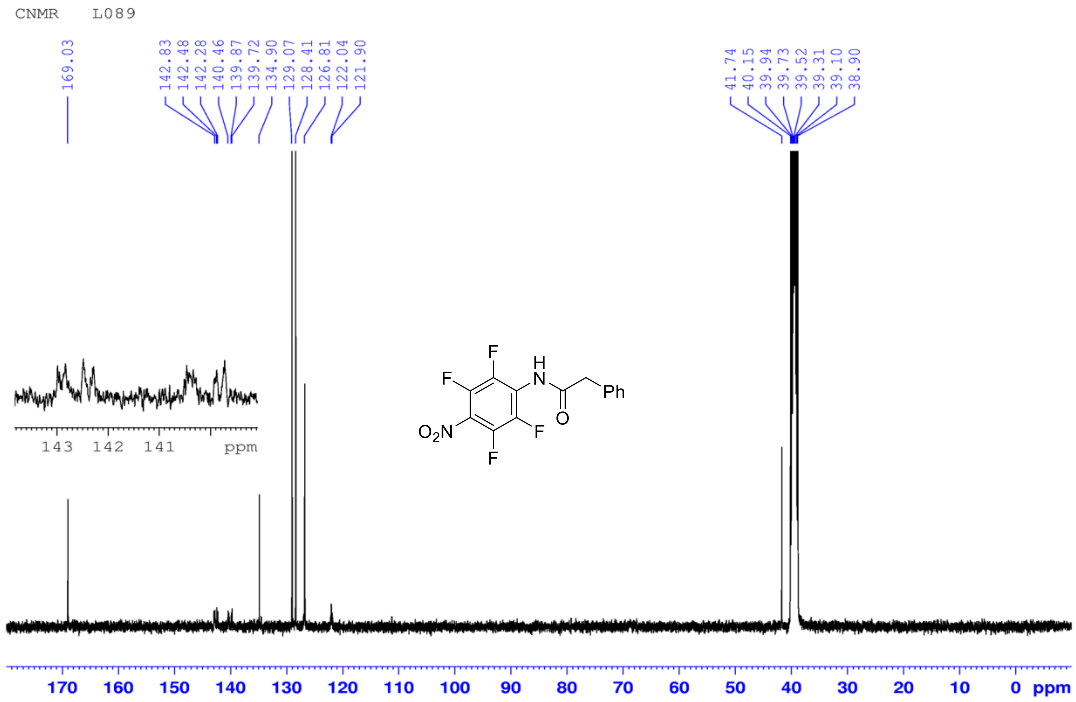

CNMR L089
169.03
142.83
142.48
142.28
140.46
139.87
139.72
134.90
129.07
128.41
126.81
122.04
121.90
41.74
40.15
39.94
39.73
39.52
39.31
39.10
38.90
143 142 141 ppm
F
F
H
N
Ph
O
$O_2N$
F
F
170 160 150 140 130 120 110 100 90 80 70 60 50 40 30 20 10 0 ppm


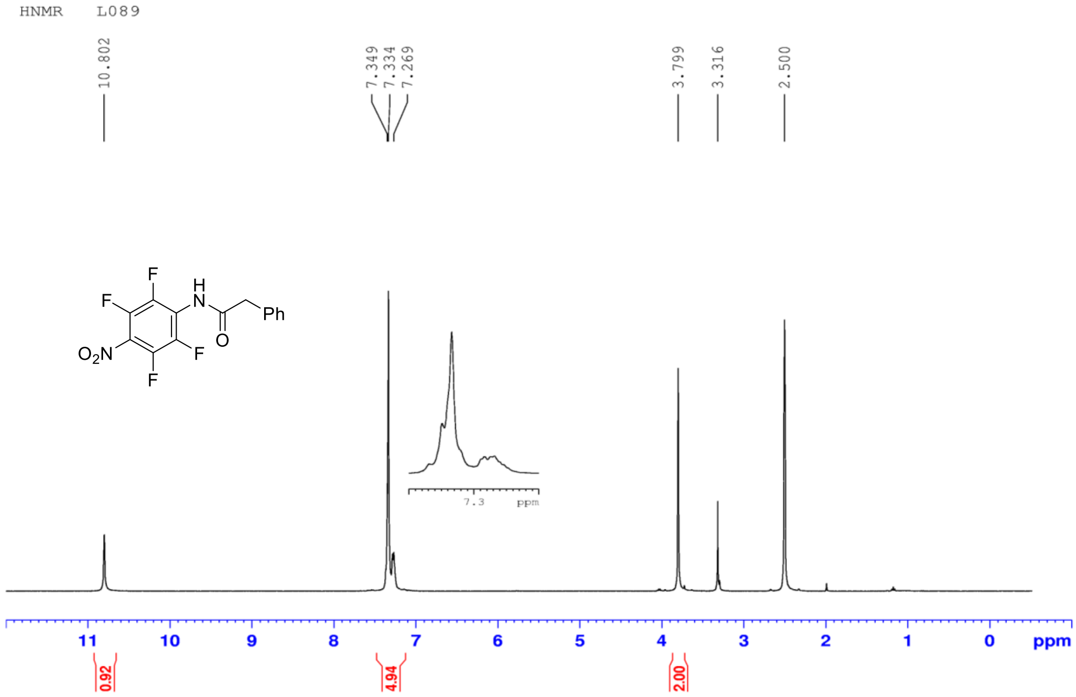

HNMR L089
10.802
7.349
7.334
7.269
3.799
3.316
2.500
F
F
H
N
Ph
O
$O_2N$
F
F
7.3 ppm
11 10 9 8 7 6 5 4 3 2 1 0 ppm
0.92
4.94
2.00

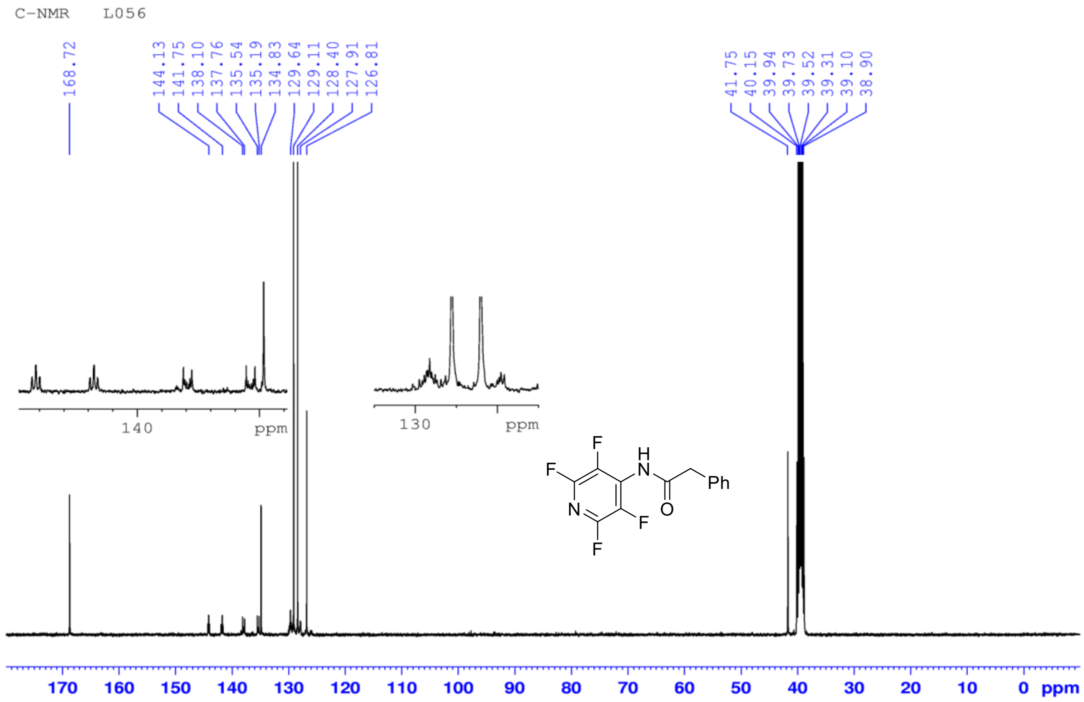
C-NMR L056
168.72
144.13
141.75
138.10
137.76
135.54
135.19
134.83
129.64
129.11
128.40
127.91
126.81
41.75
40.15
39.94
39.73
39.52
39.31
39.10
38.90
140
ppm
130
ppm
F
F
N
F
F
H
N
O
Ph
170 160 150 140 130 120 110 100 90 80 70 60 50 40 30 20 10 0 ppm

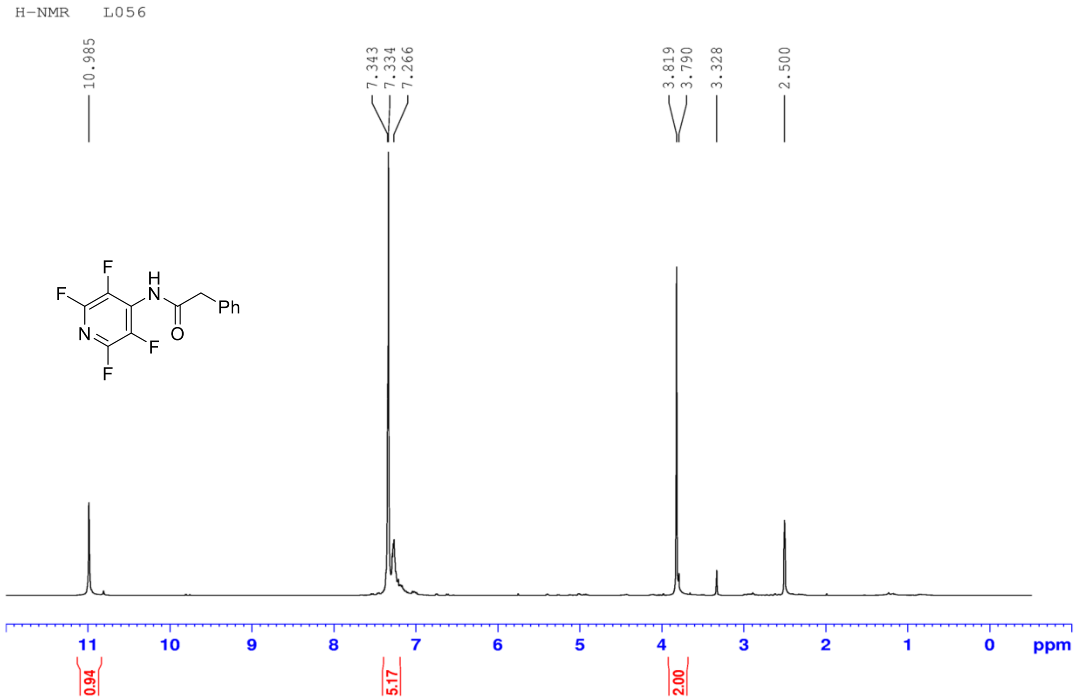
H-NMR L056
10.985
7.343
7.334
7.266
3.819
3.790
3.328
2.500
F
F
N
F
F
H
N
O
Ph
11 10 9 8 7 6 5 4 3 2 1 0 ppm
0.94
5.17
2.00

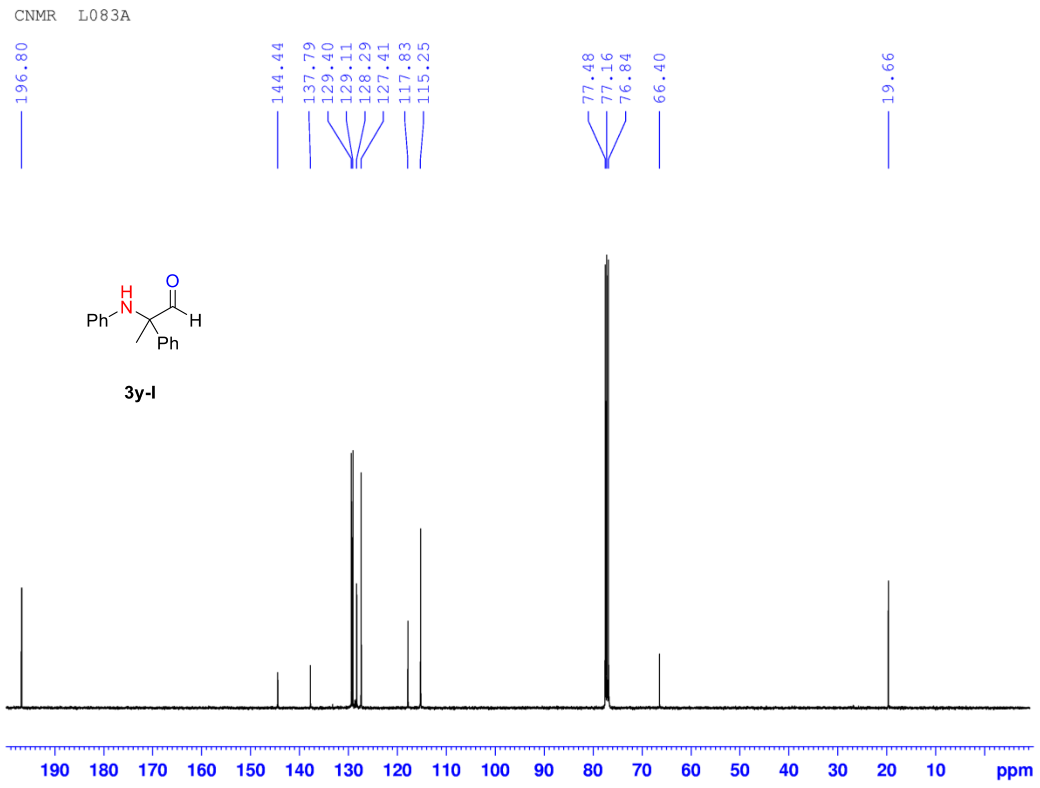
CNMR L083A
196.80
144.44
137.79
129.40
129.11
128.29
127.41
117.83
115.25
77.48
77.16
76.84
66.40
19.66
Ph
N
H
O
H
Ph
3y-l
190 180 170 160 150 140 130 120 110 100 90 80 70 60 50 40 30 20 10 ppm

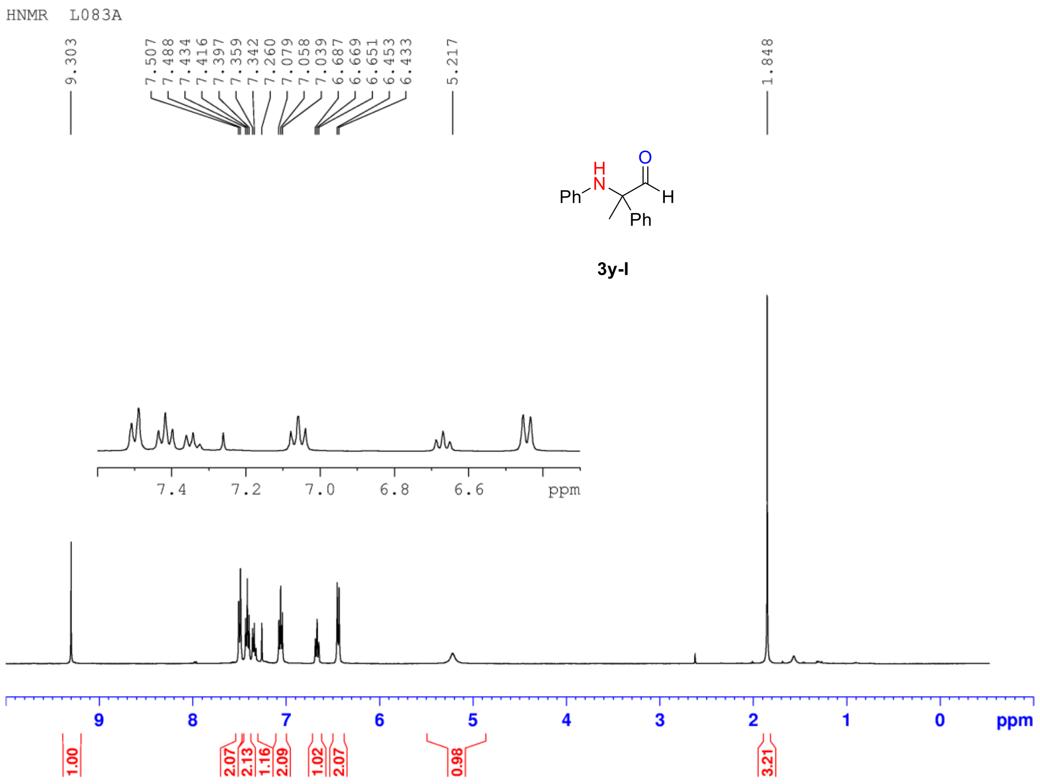
HNMR L083A
9.303
7.507
7.488
7.434
7.416
7.397
7.359
7.342
7.260
7.079
7.058
7.039
6.687
6.669
6.651
6.453
6.433
5.217
1.848
Ph
N
H
O
H
Ph
3y-l
7.4 7.2 7.0 6.8 6.6 ppm
9 8 7 6 5 4 3 2 1 0 ppm
1.00
2.07
2.13
1.16
2.09
1.02
2.07
0.98
3.21

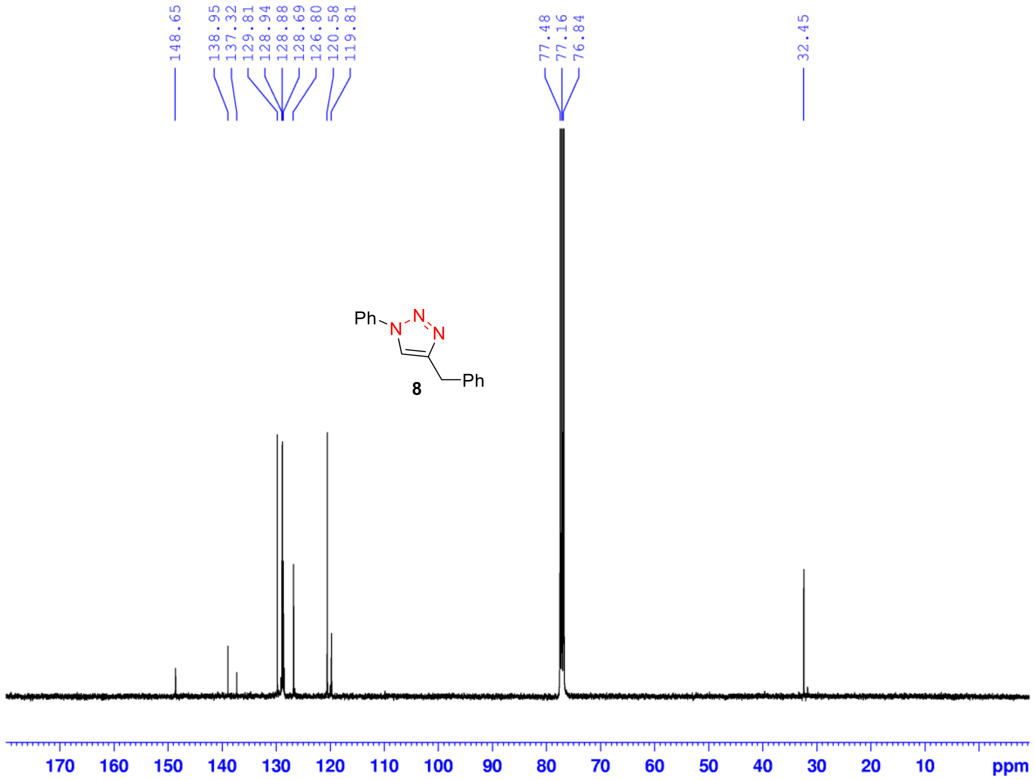
148.65
138.95
137.32
129.81
128.94
128.88
128.69
126.80
120.58
119.81
77.48
77.16
76.84
32.45
Ph
N
N
N
8
Ph
170 160 150 140 130 120 110 100 90 80 70 60 50 40 30 20 10 ppm

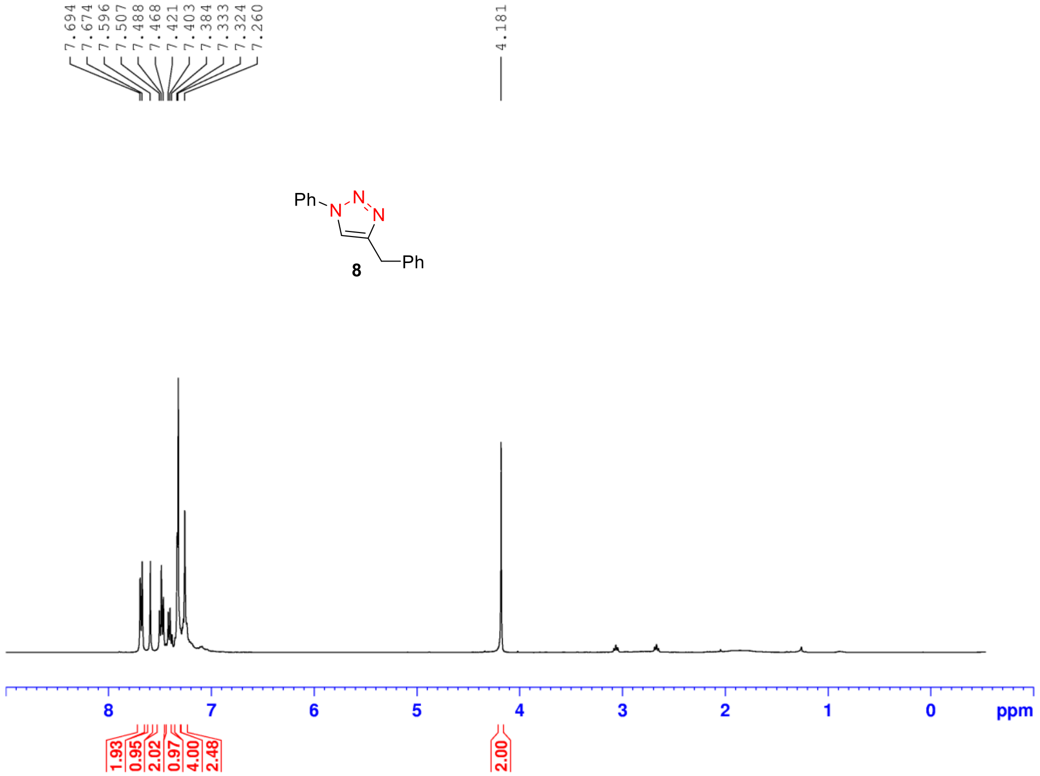
7.694
7.674
7.596
7.507
7.488
7.468
7.421
7.403
7.384
7.333
7.324
7.260
4.181
Ph
N
N
N
8
Ph
8 7 6 5 4 3 2 1 0 ppm
1.93
0.95
2.02
0.97
4.00
2.48
2.00

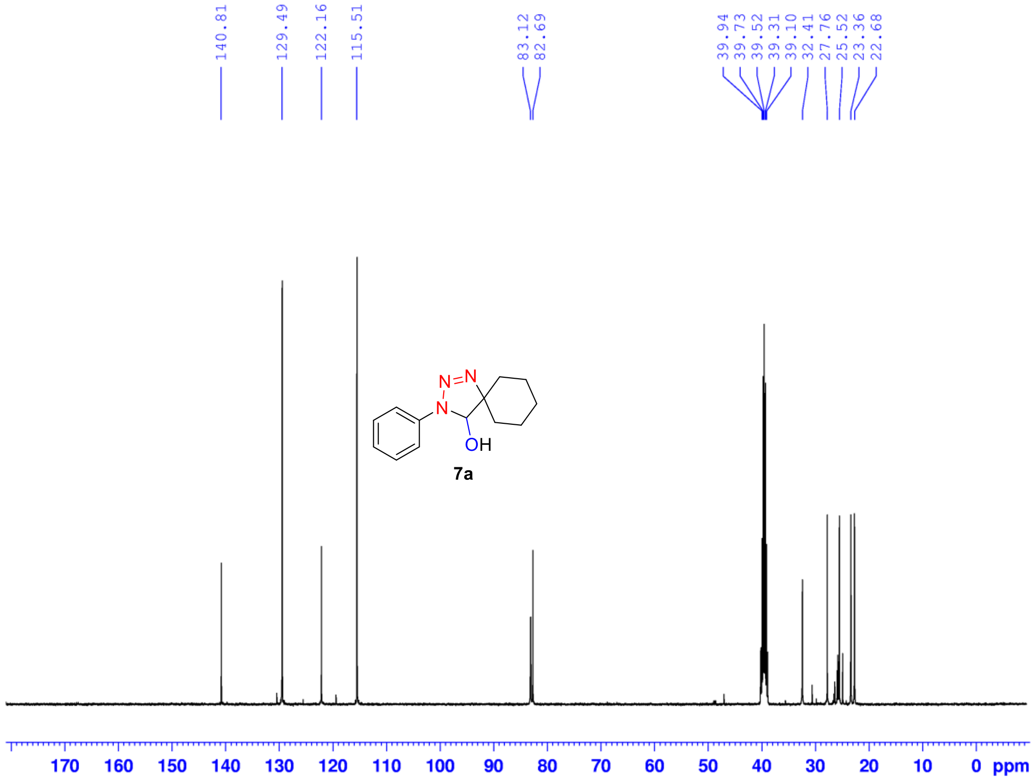
140.81
129.49
122.16
115.51
83.12
82.69
39.94
39.73
39.52
39.31
39.10
32.41
27.76
25.52
23.36
22.68
N
N
N
OH
7a
170 160 150 140 130 120 110 100 90 80 70 60 50 40 30 20 10 0 ppm

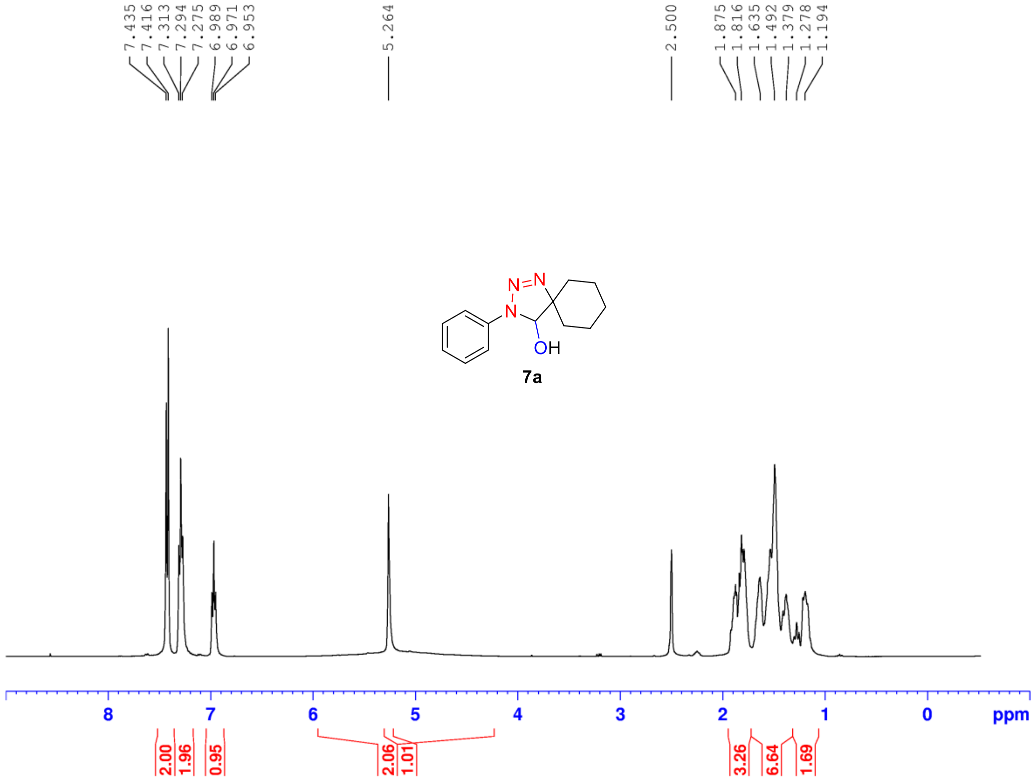
7.435
7.416
7.313
7.294
7.275
6.989
6.971
6.953
5.264
2.500
1.875
1.816
1.635
1.492
1.379
1.278
1.194
N
N
N
OH
7a
8 7 6 5 4 3 2 1 0 ppm
2.00
1.96
0.95
2.06
1.01
3.26
6.64
1.69
